# Synthesis of Stereodefined Polysubstituted Bicyclo[1.1.0]butanes

**DOI:** 10.1021/jacs.4c04438

**Published:** 2024-05-09

**Authors:** Rahul Suresh, Noam Orbach, Ilan Marek

**Affiliations:** Schulich Faculty of Chemistry and The Resnick Sustainability Center for Catalysis, Technion−Israel Institute of Technology, Technion City, Haifa 32000, Israel

## Abstract

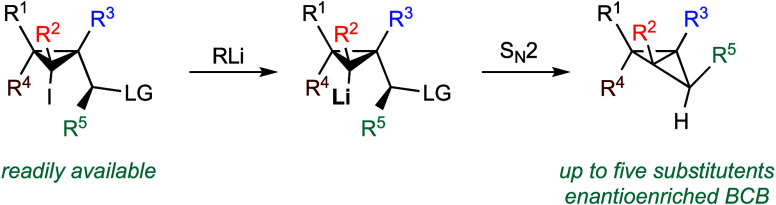

We report a highly diastereoselective synthesis of polysubstituted bicyclobutanes possessing up to three stereodefined quaternary centers and five substituents. Our strategy involves a diastereoselective carbometalation of cyclopropenes followed by a cyclization to furnish the bicyclobutane ring system. This straightforward approach allows for the incorporation of a diverse range of substituents and functional groups, notably without the need for electron-withdrawing functionalities.

Bicyclo[1.1.0]butane (BCB), the smallest fused-ring carbocycle with the highest strain energy (64 kcal/mol),^1^ was first synthesized by Wiberg and Ciula in 1959.^2^ Since then, BCBs have been indispensable synthetic building blocks because of their unusual bonding and the chemistry enabled by strain release,^3−12^ including reaction with nucleophiles,^13−22^ electrophiles,^23−27^ radicals,^28−31^ carbenes,^32,33^ and pericyclic reactions,^34,35^ to generate diverse molecular frameworks. In recent years, we have witnessed a remarkable renaissance of the field with BCBs serving as an entry to stereodefined cyclobutanes^19^ and C(sp^3^)-rich bioisosteres.^36−38^ However, the synthesis of stereodefined polysubstituted BCBs has received limited attention. Following the pioneering independent reports by Moore and Skattebo̷l of the synthesis of bridge-substituted BCBs,^39,40^ several alternative strategies have appeared in the literature for the preparation of BCBs possessing two or more substituents (Scheme 1a).^41−59^ As the number of substituents increases, many of these strategies often struggle to achieve precise stereochemical control, especially stereocontrolled quaternary centers. Furthermore, most of them necessitate electron-withdrawing groups (EWGs) on a bridgehead carbon center.^46−54^ These limitations significantly restrict the diversity of accessible BCBs. Another approach to incorporate substituents is to metalate the bridgehead or the bridge position of simple BCBs followed by electrophile trapping.^60−63^ The latter strategy is facilitated by the presence of EWGs. In this context, Anderson introduced an additional functionalization step involving the metalation of the bridge C–H bond using a lithium base followed by electrophile trapping/cross coupling, which results in the formation of bridge-substituted BCBs (Scheme 1b).^62,63^ However, these approaches are limited in their ability to accommodate stereodefined quaternary centers.

**Scheme 1:**
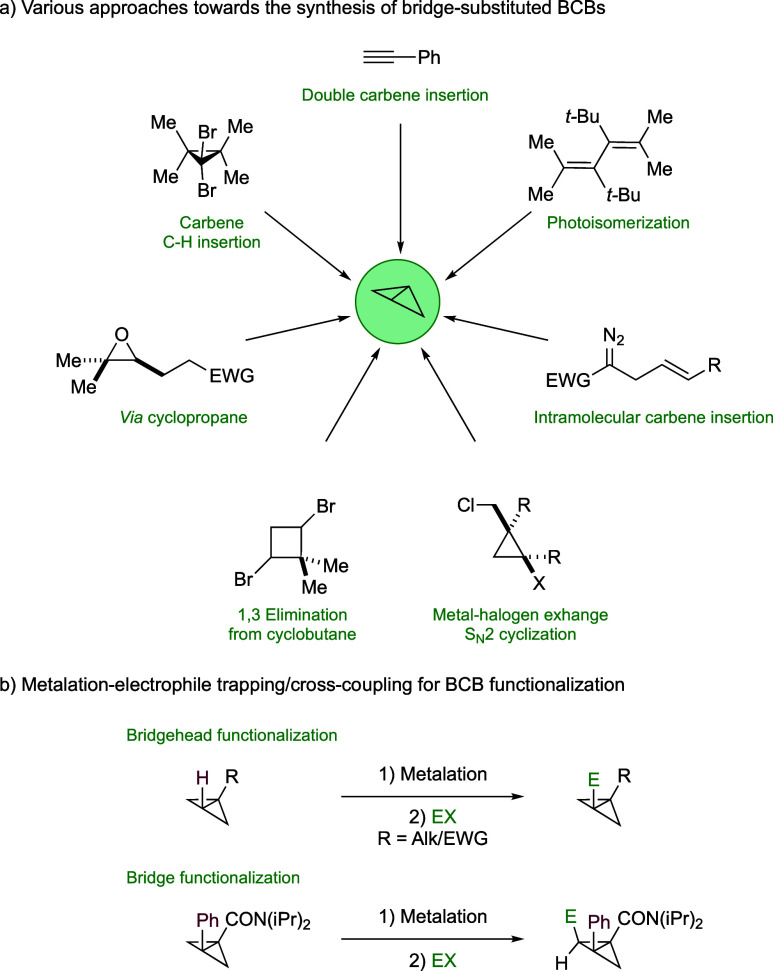
Synthetic Routes to Substituted BCBs

To the best of our knowledge, the stereoselective synthesis of nonbiased BCBs (without EWG) possessing quaternary stereocenters is unprecedented. We, therefore, addressed this synthetic gap and pursued the synthesis of polysubstituted BCBs featuring stereodefined quaternary centers by leveraging the diastereoselective carbometalation of cyclopropenes^64^ followed by further functionalization.

Our group has recently reported an efficient and straightforward synthesis of stereodefined polysubstitued spiropentanes **3**.^65^ The method involves a diastereoselective carbocupration reaction of cyclopropenes **1** to generate cyclopropyl copper species **2**, which subsequently cyclize to produce the expected spiropentanes **3** (Scheme 2a) with excellent diastereoselectivity. Motivated by the success of this approach, we set out to utilize the cyclization of cyclopropyl metal species to stereoselectively produce BCBs (Scheme 2b).

**Scheme 2:**
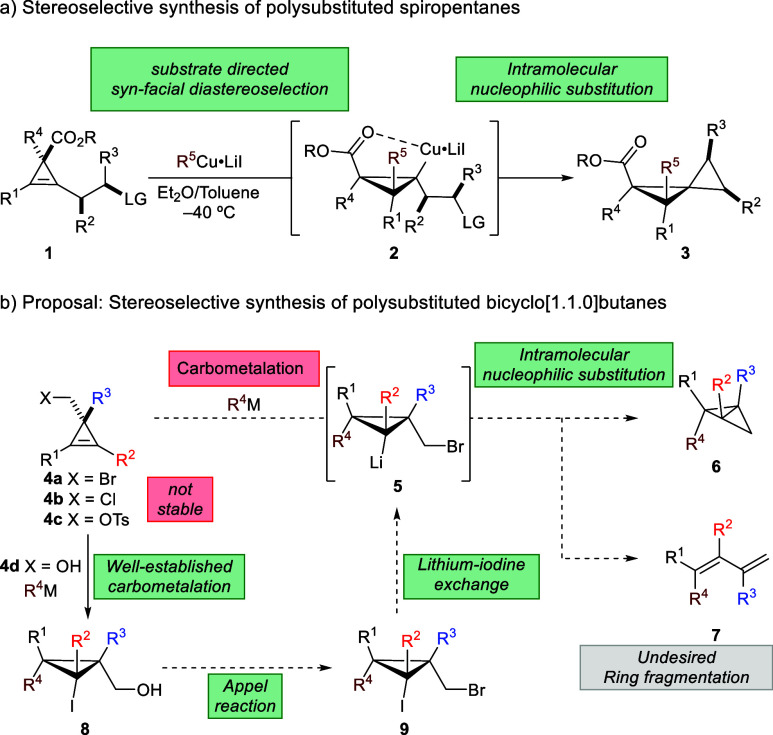
Proposed Strategy

Therefore, our initial proposal involved a *syn*-diastereocontrolled carbometalation reaction of cyclopropenes **4** possessing a leaving group at the cyclopropenyl carbinyl position (X = Br, Cl, OTs; **4a**–**4c**, respectively). Subsequently, the resulting cyclopropylmetals **5** would undergo an intramolecular nucleophilic substitution to produce the desired BCBs **6** in a single-pot operation from **4**. However, we quickly realized that this proposed approach had several pitfalls, such as the stability of the starting material when X is a good leaving group and that the desired cyclization of cyclopropylmetals **5** into **6** could be overshadowed by a ring fragmentation into dienes **7**. As a result of these considerations, we therefore decided to break down the strategy by utilizing metal–halogen exchange followed by cyclization.^42,53−55^ An initial and well-established diastereoselective copper-catalyzed carbomagnesiation reaction of stable cyclopropenyl carbinols **4d**(66−68) followed by electrophile trapping with I_2_ would provide cyclopropyl iodides **8** in excellent yields and diastereomeric ratios. An Appel reaction would convert alcohols **8** into key intermediate bromides **9**. The latter would be poised to undergo lithium–iodine exchange followed by the desired cyclization en route to polysubstituted stereodefined BCBs **6**. It should be, however, emphasized that a selective lithium–halogen exchange^69^ of **9** is required, in addition to an intramolecular nucleophilic substitution, rather than the undesired fragmentation reaction into diene **7** (Scheme 2b).

Cyclopropenyl alcohols **4d** were readily obtained through a Rh-catalyzed decomposition of diazoesters in the presence of alkynes followed by reduction.^70−73^ Subsequently, we used the well-established copper-catalyzed carbomagnesiation reaction^66−68,74−83^ to provide the corresponding products **8a**–**r** as a single diastereomer. It should be noted that for cases where R^1^, R^2^, R^3^ ≠ H, **8o**–**r**, the iodination reaction proceeds in low yields, and therefore, the carbometalation–iodination sequence was performed on cyclopropenyl esters (see the Supporting Information for all details).^84^ An Appel reaction then transformed the alcohols into bench- and column-stable (bromomethyl)–iodocyclopropanes **9a**–**r** (Scheme 3). *gem*-Disubstituted (bromomethyl)–iodocyclopropanes featuring several alkyl groups (**9a**–**c**, Scheme 3) or a vinyl group (**9d**) or possessing a functionality, such as a chlorine atom (**9e**), were easily accessed with very high diastereoselectivity and moderate to high yields. When symmetrical sp^2^-disubstituted cyclopropenyl carbinols were employed (R^1^ = R^2^ = alkyl, R^1^ = R^2^ = Ph), the sequence proceeded similarly to provide trisubstituted (bromomethyl)–iodocyclopropanes **9f**–**i** as single diastereomers. In the case of nonsymmetric disubstituted cyclopropenes, the regioselectivity of the addition is determined by the presence of a π-hybridized carbon (**9f**–**j**, Scheme 3) or by a silicon-stabilized carbanion (**9k**,**l**, Scheme 3) linked to the sp^2^-carbon center of the cyclopropenes, while diastereoselectivity is influenced by the chelation of the organometallic species by the alcoholate. Substituent R^3^ can be an aryl or alkyl group, and this strategy can also efficiently yield challenging *per* (fully)-substituted (bromomethyl)–iodocyclopropanes (**9o**–**r**, Scheme 3).

**Scheme 3:**
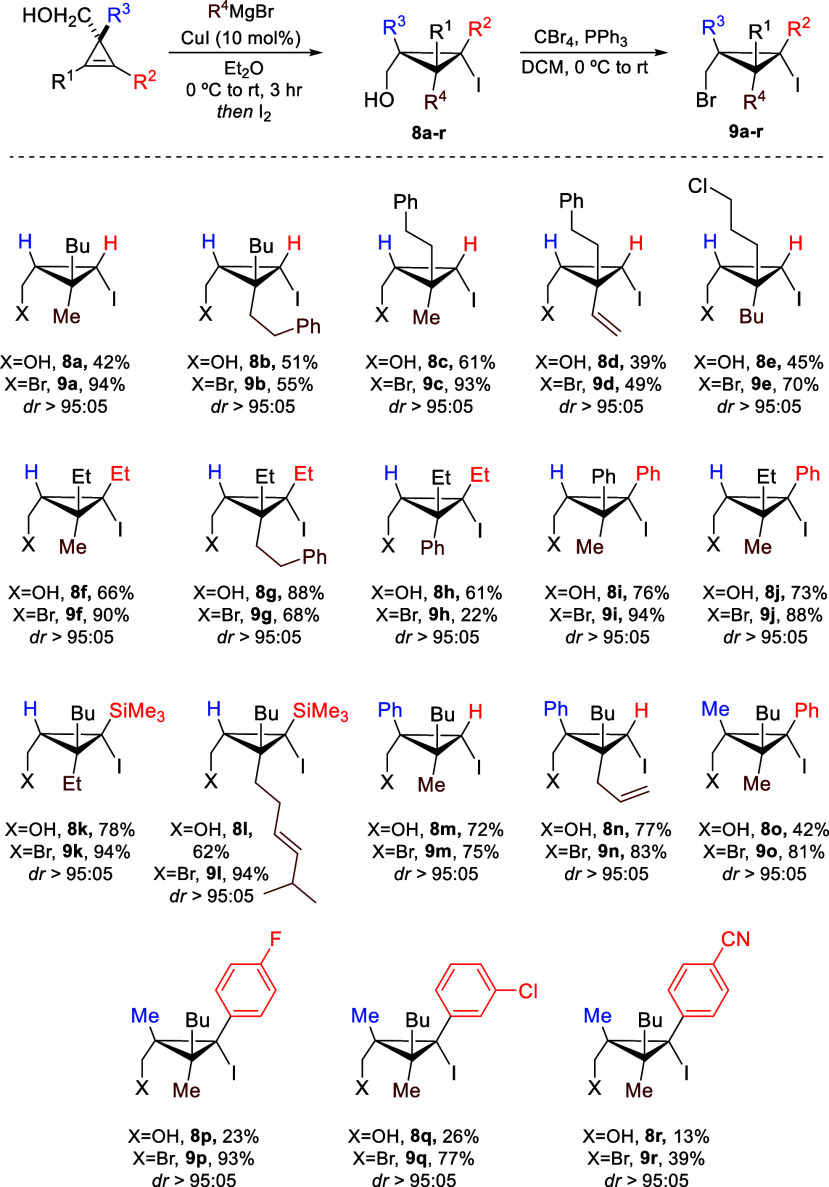
Diastereoselective Preparation of Polysubstituted Bromomethyl–iodocyclopropanes

Treatment of (bromomethyl)–iodocyclopropanes **9** with *n*-BuLi in THF at −78 °C for 15 min, followed by warming up the reaction mixture to 0 °C, provided BCBs **6** in high yields as a single diastereomer (dr > 95:05, Scheme 4).^85^ In such conditions, an exclusive iodine–lithium exchange reaction occurs instead of the bromo–lithium exchange, even if the former is secondary or tertiary. To illustrate the effectiveness of these optimized conditions, the simplest substrates **9a**–**e** gave BCBs **6a**–**e** possessing a single quaternary stereocenter in excellent yields (with the exception of the volatile **6a**). It is worth noting that this reaction is applicable to alkenyl cyclopropane (**9d** into **6d**, Scheme 4) and proceeds selectively in the presence of functional groups such as primary alkyl chloride (**6e**, Scheme 4). When highly substituted **9f**–**l** were engaged in this transformation, BCBs **6f**–**l** possessing two quaternary stereocenters were produced in high yields. Of note, BCB **6h** would otherwise represent a challenge to prepare because of the phenyl group on the axial position but was successfully produced in high yields. To showcase the synthetic utility and practicality of this protocol, the reaction could be performed using cyclopropane **9j** on a 1.5 g scale to furnish BCB **6j** in 82% yield.^86^ Additionally, BCBs with a silyl group positioned at the bridgehead were synthesized in a similar manner (**6k**,**l**, Scheme 4). We later focused on cyclopropanes **9m,n** with phenyl substituent β in the leaving group. They underwent the reaction and yielded BCBs **6m,n** bearing an allyl group and a methyl group, respectively, in the axial position.

**Scheme 4:**
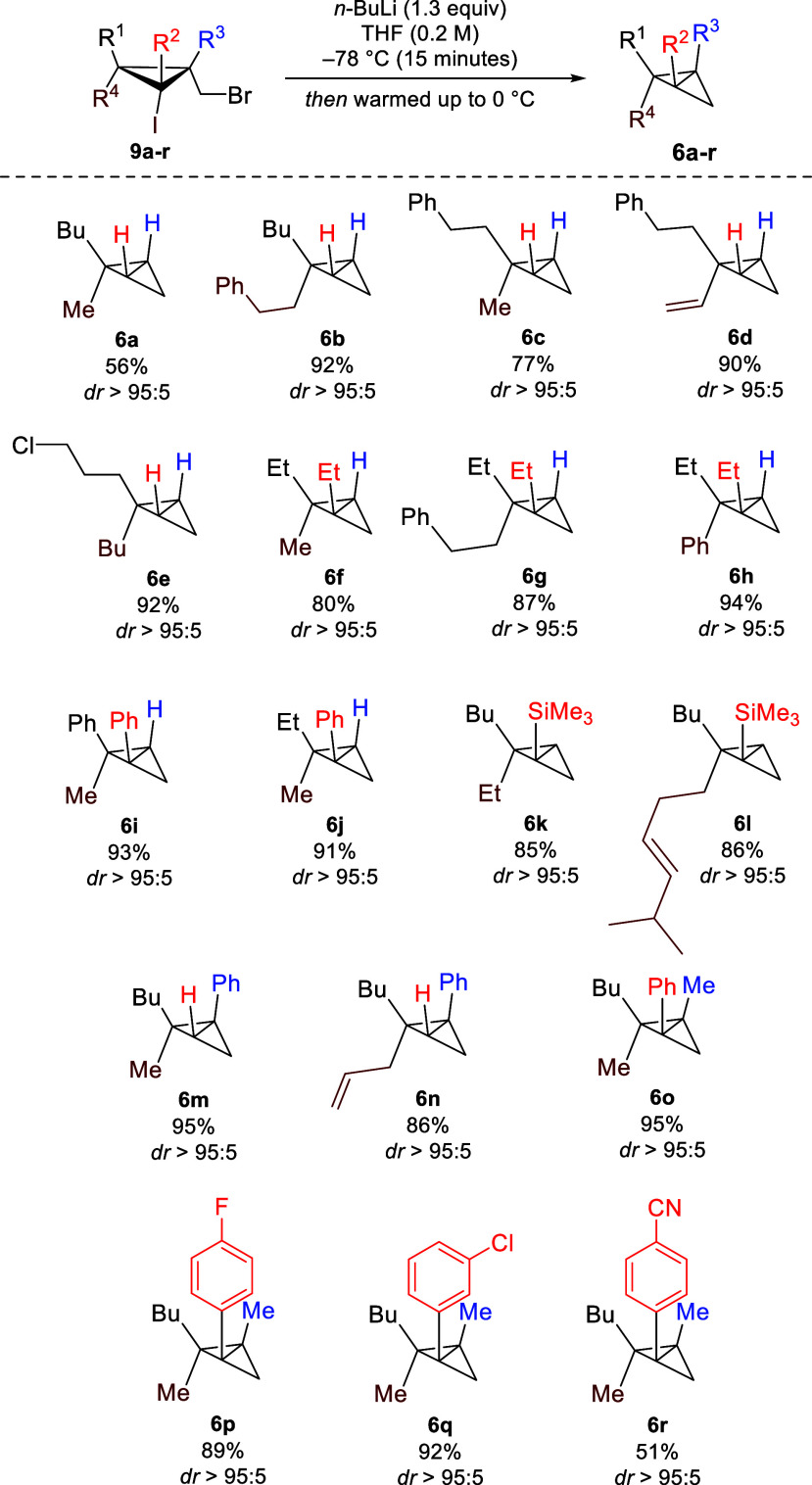
Synthesis of Polysubstituted BCBs Yields were measured using ^1^H NMR with *tert*-butyl methyl ether as the internal standard.

Stability assessment reveals that these BCBs remain stable at room temperature, as evidenced by the absence of visible decomposition in the pentasubstituted BCB **6s** stored at room temperature for three months. Furthermore, at −20 °C, BCBs exhibit prolonged stability with no visible decomposition observed in the NMR spectra of substrate **6l** for nine months or substrate **6m** for several months. Next, we proceeded toward the synthesis of tetrasubstituted BCBs. To achieve this goal, hexasubstituted cyclopropanes **9o**–**r** were subjected to our standard experimental conditions, and to our delight, we successfully obtained sterically demanding tetrasubstituted BCBs **6o**–**6r** featuring three contiguous quaternary stereocenters without any observable ring fragmentation. It is worth noting that functionalized aryl groups on the tetrasubstituted BCBs were compatible with no interference or formation of side products, even when an alkyllithium sensitive nitrile functionality was present (Scheme 4).

Our methodology can also be extended to the synthesis of enantioenriched BCBs. Enantioenriched bromomethyl iodocyclopropane 9m* was obtained with 90:10 er from the enantioenriched cyclopropene prepared using a commercially available chiral rhodium catalyst.^87^ The reaction proceeded smoothly to obtain BCB 6m* with high yield and excellent enantiospecificity (Scheme 5).^88^ To the best of our knowledge, this is the first example of an enantioenriched BCB bearing a quaternary carbon center on the bridge.

**Scheme 5:**
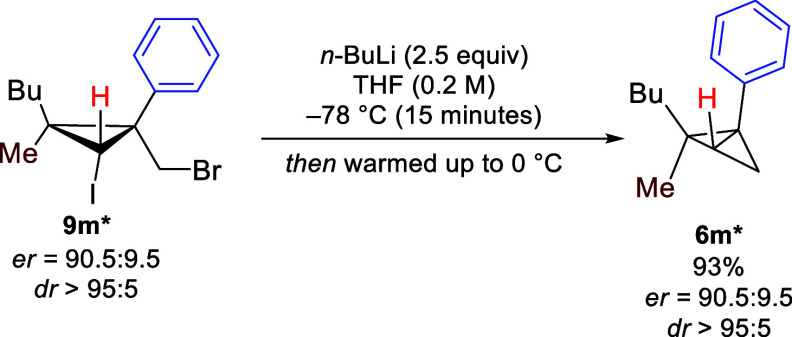
Synthesis of Enantioenriched BCB Yields were measured using ^1^H NMR with *tert*-butyl methyl ether as the internal standard.

Encouraged by these results, we finally targeted the synthesis of challenging pentasubstituted BCBs. We sought to use our methodology to obtain the challenging nonbiased BCB with different substituents in a stereoselective manner. Starting material **8t** was accessed through Grignard addition to a *per*-substituted iodocyclopropyl aldehyde (see the Supporting Information for details). When **8t** was subjected to the Appel reaction, it fragmentated, presumably because of the high stabilization of the corresponding cationic intermediate. For this reason, we used an alternative approach in which alcohol **8t** was converted into a leaving group via mesylation to avoid preparation of a less stable halide to side reactions. The mesylate **9s** under our standard reaction conditions yielded the desired pentasubstituted BCB **6s** as a single diastereomer (Scheme 6). The relative stereochemistry was determined using 2D NOESY spectroscopy (see the Supporting Information for details).

**Scheme 6:**
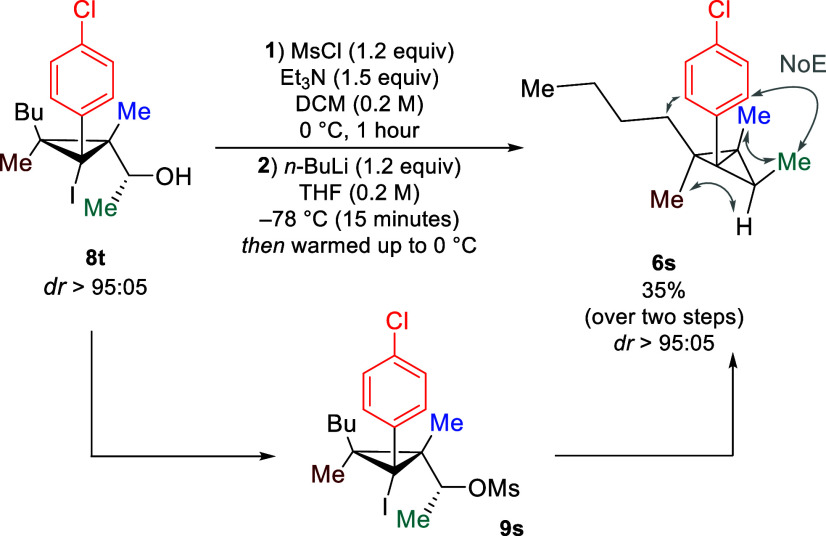
Synthesis of Pentasubstituted BCB Yields were measured using ^1^H NMR with *tert*-butyl methyl ether as the internal standard.

In conclusion, we have developed a simple and straightforward approach to synthesize stereodefined polysubstituted BCBs. This method enables the preparation of BCBs lacking EWGs and bearing up to three quaternary stereocenters with a diverse scope of substituents and substitution pattern. This methodology was used to obtain enantioenriched BCB bearing quaternary centers. Moreover, we synthesized the first nonbiased pentasubstituted BCB with different substituents.

## Supporting Information Available

Experimental procedures; characterization data for all new compounds, along with copies of spectra; computational methods and data; and geometries of computed intermediates and transition states (PDF)

## Abbreviations

BCB: bicyclo[1.1.0]butane
EWG: electron-withdrawing group
