# Addition to “Synthesis of Stereodefined Polysubstituted Bicyclo[1.1.0]butanes”

**DOI:** 10.1021/jacs.4c06987

**Published:** 2024-06-13

**Authors:** Rahul Suresh, Noam Orbach, Ilan Marek

A key reference was omitted for the original publication and should be included as follows.^1^
