# Supplementary material for: Synthesis of Stereodefined Polysubstituted Bicyclo[1.1.0]butanes
Source: J Am Chem Soc. 2024 May 9;146(20):13748–53. doi: 10.1021/jacs.4c04438 (PMC11117409; doi:10.1021/jacs.4c04438)

Synthesis of Stereodefined Polysubstituted Bicyclo[1.1.0]butanes

Rahul Suresh, Noam Orbach, and Ilan Marek*

Schulich Faculty of Chemistry and The Resnick Sustainability Center for Catalysis
Technion–Israel Institute of Technology
Technion City, Haifa 32000, Israel

Contents

1. General information 3

2. Synthesis of starting materials 4

2.1 General synthesis of cyclopropenes. 4

2.2 General procedure for the palladium-catalyzed cross coupling of stannylcyclopropenes. 4

2.3 General procedure for the copper-catalyzed carbometallation of cyclopropenes folllowed by iodine trapping. 5

2.4 General procedure for synthesis of hexasubstituted iodocyclopropane methanol derivatives. 11

2.5 General procedure for synthesis of secondary iodocyclopropyl ethanol derivatives. 14

2.6 General procedure for the synthesis of bromomethyl-iodocyclopropane derivatives. 15

2.7 General procedure for the synthesis of polysubstituted BCBs. 21

2.8 General procedure for the synthesis of Pentasubstituted BCB. 28

3. References. 30

4. Spectrum of compounds 31

1. General information

Unless otherwise stated, reactions were conducted in a flame-dried glassware under a positive pressure of argon. Et_2O and THF were dried from Pure-Solv® Purification System (Innovative Technology©). All organolithium reagents were purchased from Aldrich. All other reagents were purchased from Aldrich, Strem, Acros, and Alfa Aesar, and used as received. Thin-layer chromatography (TLC) was conducted with E. Merck silica gel 60 F254 pre-coated plates, (0.25 mm) and visualized by exposure to UV light (254 nm) or stained with anisaldehyde, CAM (solution of $Mo_7(NH_4)_6O_{24}$) and $(NH_4)_4Ce(SO_4)_4$), phosphomolybdic acid, or potassium permanganate. Column chromatography was performed using Fluka silica gel 60 Å (40- 63 µm, 230-400 mesh). All NMR spectra were recorded on Bruker spectrometers (AVII 300, AVIII 400, AVII500 and AVII 600), and reported relative to deuterated solvent signals and/or $SiMe_4$ as internal standard. Chemical shifts are reported in parts per million (ppm) with respect to the residual solvent signal $CDCl_3$ (^1H NMR: δ = 7.26; ^{13}C NMR: δ = 77.16) or C_6D_6 (^1H NMR: δ = 7.16; ^{13}C NMR: δ = 128.06.) Peak multiplicities are reported as follows: s = singlet, bs = broad singlet, d = doublet, t = triplet, q = quartet, quin = quintet, sext = sextet, sep= septet, dd = doublet of doublets, td = triplet of doublets, ddd = doublet of doublets of doublets, m = multiplet. High-resolution mass spectra (HRMS) were obtained by the mass spectrometry facility at the Technion.

2. Synthesis of starting materials

2.1 General synthesis of cyclopropenes.

All cyclopropenyl esters and cyclopropenyl carbinyl derivatives were prepared according to the previous reported methods.[1,2,3]

2.2 General procedure for the palladium-catalyzed cross coupling of stannylcyclopropenes.

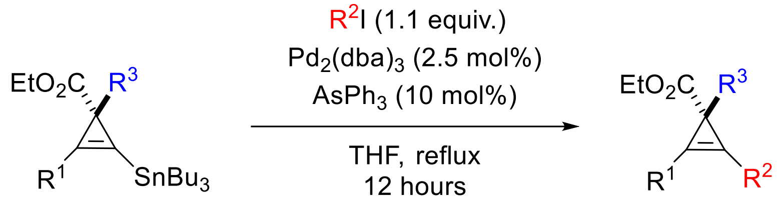

In a flame dried three neck round bottom flask, $Pd_2(dba)_3$ (0.025 equiv.) and Ph_3As (0.1 equiv.) were taken in THF (0.2 M) and were stirred at room temperature for 15 minutes. A solution of stannylcyclopropene (1 equiv.) and aryliodide (1.1 equiv.) in THF (0.1 M) was cannulated into the reaction mixture, heated to 55 °C and stirred at this temperature for 12 hours. The reaction mixture was filtered through silica with Et_2O as the solvent and concentrated in vacuo. The crude product was further purified by silica gel column chromatography to obtain the desired product **S1a-c**.[4]

Ethyl-2-butyl-3-(4-fluorophenyl)-1-methylcycloprop-2-ene-1-carboxylate (S1a)

Scale, physical description, yield, mass: 3.2 mmol, colorless oil, 87% yield, 0.746 gr (2.7 mmol).

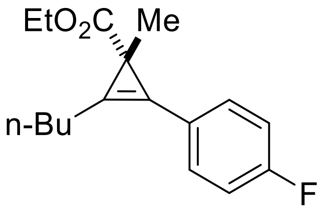

^{1}H-NMR (400 MHz, $CDCl_3$): δ 7.42 – 7.34 (m, 2H), 7.07 (t, J = 8.7 Hz, 2H), 4.09 (dddd, J = 18.1, 11.0, 7.2, 3.8 Hz, 2H), 2.62 (t, J = 7.3 Hz, 2H), 1.73 – 1.62 (m, 2H), 1.46 (s, 3H), 1.45 – 1.32 (m, 2H), 1.17 (t, J = 7.1 Hz, 3H), 0.96 (t, J = 7.4 Hz, 3H).

^{13}C-NMR (101 MHz, $CDCl_3$): δ 177.10, 162.71 (d, J = 248.7 Hz), 130.89 (d, J = 8.1 Hz), 123.73 (d, J = 3.3 Hz), 116.01 (d, J = 22.0 Hz), 115.43 (d, J = 2.5 Hz), 109.32, 60.36, 29.83, 26.73, 24.49, 22.64, 18.67, 14.54, 13.93.

^{19}F NMR (377 MHz, $CDCl_3$): δ -112.14.

HRMS (APCI): m/z calculated for $C_{17}H_{22}FO_2$ $[M+H]^+$: 277.1598, found 277.1591.

Ethyl-2-butyl-3-(4-chlorophenyl)-1-methylcycloprop-2-ene-1-carboxylate (S1b)

Scale, physical description, yield, mass: 14.4 mmol, colorless oil, 50% yield, 2.1 gr (7.1 mmol).

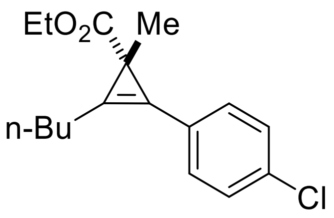

^{1}H-NMR (400 MHz, $CDCl_3$): δ 7.34 (d, J = 3.0 Hz, 4H), 4.08 (dtt, J = 18.0, 11.0, 7.1 Hz, 2H), 2.62 (t, J = 7.3 Hz, 2H), 1.73 – 1.64 (m, 2H), 1.46 (s, 3H), 1.44 (d, J = 8.8 Hz, 2H), 1.17 (t, J = 7.1 Hz, 3H), 0.95 (t, J = 7.4 Hz, 3H).

^{13}C-NMR (101 MHz, $CDCl_3$): δ 176.94, 134.26, 130.30, 129.12, 125.99, 116.88, 109.39, 60.39, 29.77, 26.74, 24.60, 22.63, 18.66, 14.53, 13.92.

HRMS (APCI): m/z calculated for $C_{17}H_{22}ClO_2$ $[M+H]^+$: 293.1303, found 293.1301.

Ethyl -2-butyl-3-(4-cyanophenyl)-1-methylcycloprop-2-ene-1-carboxylate (S2c)

Scale, physical description, yield, mass: 3.2 mmol, colorless oil, 97% yield, 850 mg (3.0 mmol). Diethyl ether is present in the spectrum.

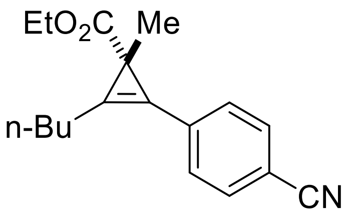

^{1}H-NMR (400 MHz, $CDCl_3$): δ 7.65 (d, J = 8.2 Hz, 2H), 7.47 (d, J = 8.2 Hz, 2H), 4.08 (dddd, J = 12.8, 10.8, 7.1, 3.7 Hz, 2H), 2.67 (t, J = 7.4 Hz, 2H), 1.78 – 1.63 (m, 2H), 1.49 – 1.40 (m, 5H), 1.17 (t, J = 7.1 Hz, 3H), 0.87 – 0.78 (m, 3H).

^{13}C-NMR (101 MHz, $CDCl_3$): δ 176.21, 132.59, 131.93, 129.42, 121.57, 118.80, 111.55, 109.48, 60.56, 29.62, 27.06, 24.81, 22.59, 18.59, 14.46, 13.84.

HRMS (APCI): m/z calculated for $C_{18}H_{22}NO_2$ $[M+H]^+$: 284.1645, found 284.1649.

2.3 General procedure for the copper-catalyzed carbometallation of cyclopropenes folllowed by iodine trapping.

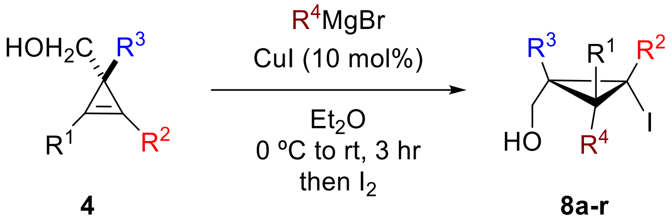

In a flame dried three neck round bottom flask, copper iodide (0.1 equiv.) was taken and purged with argon three times. Et_2O (0.1 M) was added through a septum followed by the addition of cyclopropene (**4**, 1.0 equiv.). This solution was cooled to 0 °C (ice bath), followed by the dropwise addition of a Grignard reagent (3.0 equiv.). The solution was warmed to room temperature over three hours. Upon full consumption of the cyclopropene (followed by TLC $KMnO_4$ or p-anisaldehyde), the reaction mixture was cooled to 0 °C (ice bath), followed by addition of iodine

(3 equiv.). After warming up to room temperature for over three hours, the reaction was quenched with 1:1 saturated solution of NH_4Cl and NH_4OH. This mixture was directly transferred to a separation funnel with a saturated solution of $Na_2S_2O_4$ and extracted with Et_2O (3 × 20 mL/mmol). The combined organic layers were dried with Na_2SO_4, filtered, and concentrated in vacuo. The crude product was further purified by silica gel column chromatography to obtain the desired product **8**. (Product usually comes with 15-20% Et_2O/Petroleum ether).

2-Butyl-3-iodo-2-methylcyclopropyl)methanol (8a)

Scale, physical description, yield, mass: 5.0 mmol, colorless oil, 42% yield, 567 mg (2.1 mmol).

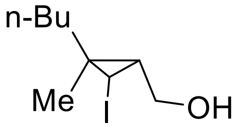

^{1}H-NMR (400 MHz, $CDCl_3$): δ 3.73 (dt, J = 11.6, 5.5 Hz, 1H), 3.66 – 3.54 (m, 1H), 2.68 (d, J = 7.9 Hz, 1H), 1.39 – 1.22 (m, 6H), 1.10 (s, 3H), 0.96 – 0.81 (m, 4H).

^{13}C-NMR (101 MHz, $CDCl_3$): δ 64.85, 40.86, 28.71, 27.68, 23.61, 22.88, 18.64, 14.24, 8.71.

HRMS (APCI): m/z calculated for $C_9H_{16}I$ $[M-OH]^+$: 251.0291, found 251.0304.

2-Butyl-3-iodo-2-phenethylcyclopropyl)methanol (8b)

Scale, physical description, yield, mass: 4.7 mmol, colorless oil, 51% yield, 872 mg (2.4 mmol).

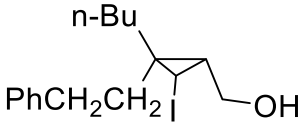

^{1}H-NMR (400 MHz, $CDCl_3$): δ 7.30 (t, J = 7.5 Hz, 2H), 7.25 – 7.16 (m, 3H), 3.70 (dd, J = 11.9, 6.1 Hz, 1H), 3.64 – 3.54 (m, 1H), 2.77 (dd, J = 13.0, 4.7 Hz, 1H), 2.70 (d, J = 7.9 Hz, 1H), 2.60 (td, J = 12.6, 5.7 Hz, 1H), 1.78 (ddd, J = 14.3, 11.8, 5.6 Hz, 1H), 1.71 – 1.63 (m, 1H), 1.56 – 1.49 (m, 1H), 1.35 (dddd, J = 19.6, 10.7, 5.9, 2.6 Hz, 5H), 0.97 – 0.85 (m, 4H).

^{13}C-NMR (101 MHz, $CDCl_3$): δ 142.13, 128.49, 128.40, 126.05, 64.14, 43.97, 36.94, 34.04, 31.90, 28.34, 26.71, 22.86, 14.17, 7.24.

HRMS (APCI): m/z calculated for $C_{16}H_{22}I$ $[M-OH]^+$: 341.0761, found 341.0782.

3-Iodo-2-methyl-2-phenethylcyclopropyl)methanol (8c)

Scale, physical description, yield, mass: 4.6 mmol, colorless oil, 61% yield, 890 mg (2.8 mmol).

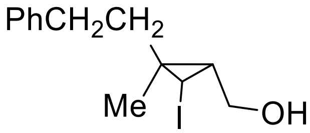

^{1}H-NMR (400 MHz, $CDCl_3$): δ 7.33 – 7.26 (m, 2H), 7.22 – 7.12 (m, 3H), 3.67 (dt, J = 12.6, 6.7 Hz, 1H), 3.63 – 3.52 (m, 1H), 2.77 – 2.67 (m, 2H), 2.65 (d, J = 7.9 Hz, 1H), 1.68 (tt, J = 9.6, 6.6 Hz, 2H), 1.34 (t, J = 6.1 Hz, 1H), 1.20 (s, 3H), 0.89 – 0.79 (m, 1H).

^{13}C-NMR (101 MHz, $CDCl_3$): δ 141.76, 128.62, 128.48, 126.15, 64.77, 43.00, 32.91, 27.83, 23.37, 18.66, 8.20.

HRMS (APCI): m/z calculated for $C_{13}H_{16}I$ $[M-OH]^+$: 299.0291, found 299.0284.

3-Iodo-2-phenethyl-2-vinylcyclopropyl)methanol (8d)

Scale, physical description, yield, mass: 3.0 mmol, colorless oil, 39% yield, 380 mg (1.2 mmol).

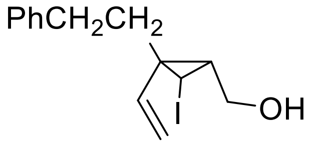

^{1}H-NMR (400 MHz, CDCl$_3$: δ 7.29 (d, J = 7.5 Hz, 1H), 7.21 (dd, J = 7.3, 2.1 Hz, 2H), 7.17 – 7.12 (m, 2H), 5.70 (dd, J = 17.5, 10.9 Hz, 1H), 5.43 (dd, J = 10.9, 1.2 Hz, 1H), 5.38 – 5.25 (m, 1H), 3.78 – 3.66 (m, 1H), 3.58 (ddd, J = 11.9, 8.2, 5.2 Hz, 1H), 2.79 (d, J = 7.8 Hz, 1H), 2.76 – 2.66 (m, 2H), 2.07 (ddd, J = 14.0, 9.5, 6.2 Hz, 1H), 1.68 (ddd, J = 14.0, 9.7, 6.7 Hz, 1H), 1.51 (t, J = 6.2 Hz, 1H), 1.11 (td, J = 8.0, 6.4 Hz, 1H).

^{13}C-NMR (101 MHzz, CDCl$_3$): δ 141.76, 138.10, 128.63, 128.46, 126.14, 118.88, 65.05, 40.46, 32.53, 30.21, 28.77, 7.48.

HRMS (APCI): m/z calculated for $C_{14}H_{16}I$ [M-OH]$^+$: 311.0291, found 311.0312.

2-Butyl-2-(3-chloropropyl)-3-iodocyclopropyl)methanol (8e)

Scale, physical description, yield, mass: 3.7 mmol, colorless oil, 45% yield, 560 mg (1.7 mmol).

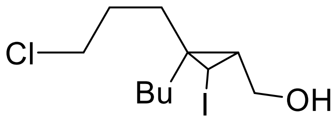

^{1}H-NMR (400 MHz, CDCl$_3$): δ 3.78 (dd, J = 11.9, 6.6 Hz, 1H), 3.66 – 3.47 (m, 3H), 2.68 (d, J = 7.9 Hz, 1H), 1.90 – 1.78 (m, 2H), 1.59 (dd, J = 7.6, 4.1 Hz, 1H), 1.51 – 1.18 (m, 8H), 0.96 – 0.82 (m, 4H).

^{13}C-NMR (101 MHz, CDCl$_3$): δ 64.14, 44.90, 34.31, 31.04, 29.27, 28.25, 27.84, 26.24, 23.15, 14.25, 7.13.

HRMS (APCI): m/z calculated for $C_{11}H_{19}ClIO$ [M-H]$^+$: 329.0164, found 329.0193.

2,3-Diethyl-2-iodo-3-methylcyclopropyl)methanol (8f)

Scale, physical description, yield, mass: 5.0 mmol, colorless oil, 66% yield, 888 mg (3.3 mmol).

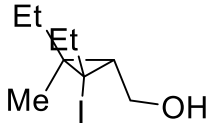

^{1}H-NMR (400 MHz, CDCl$_3$): δ 3.79 (ddd, J = 11.8, 7.8, 6.2 Hz, 1H), 3.59 (ddd, J = 12.1, 8.3, 4.0 Hz, 1H), 1.98 (dq, J = 14.2, 7.1 Hz, 1H), 1.84 (dq, J = 14.5, 7.1 Hz, 1H), 1.54 (dd, J = 7.9, 4.5 Hz, 1H), 1.51 – 1.42 (m, 2H), 1.23 (s, 3H), 1.09 (t, J = 7.1 Hz, 3H), 0.97 (t, J = 7.4 Hz, 3H), 0.33 (dd, J = 8.3, 6.2 Hz, 1H).

^{13}C-NMR (101 MHz, CDCl$_3$): δ 66.42, 41.83, 35.87, 34.99, 30.65, 28.73, 23.57, 14.87, 11.38.

HRMS (APCI): m/z calculated for $C_9H_{16}I$ [M-OH]$^+$: 251.0291, found 251.0284.

2,3-Diethyl-2-iodo-3- phenethylcyclopropyl)methanol (8g)

Scale, physical description, yield, mass: 2.4 mmol, colorless oil, 88% yield, 750 mg (2.1 mmol).

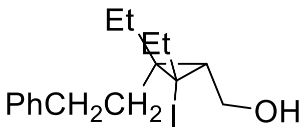

^{1}H-NMR (400 MHz, CDCl$_3$): δ 7.33 – 7.26 (m, 3H), 7.26 – 7.22 (m, 1H), 7.23 – 7.16 (m, 1H), 3.85 – 3.73 (m, 1H), 3.64 (ddd, J = 12.1, 8.3, 3.9 Hz, 1H), 2.73 (td, J = 12.8, 4.4 Hz, 1H), 2.61 (td, J = 12.8, 5.3 Hz, 1H), 2.09 – 1.88 (m, 3H), 1.83 (dq, J = 14.5, 7.3 Hz, 1H), 1.73 – 1.62 (m, 1H), 1.51 (dd, J = 7.9, 4.4 Hz, 1H), 1.33 (dq, J = 14.6, 7.4 Hz, 1H), 1.13 (t, J = 7.1 Hz, 3H), 1.04 (t, J = 7.3 Hz, 3H), 0.41 (dd, J = 8.3, 6.2 Hz, 1H).

^{13}C-NMR (101 MHz, CDCl$_3$): δ 142.31, 128.54, 128.50, 126.09, 65.87, 40.92, 37.62, 36.11, 35.91, 33.77, 31.91, 24.14, 14.65, 11.33.

HRMS (APCI): m/z calculated for $C_{16}H_{22}I$ $[M-OH]^+$: 341.0761, found 341.0782.

2,3-Diethyl-2-iodo-3-phenylcyclopropyl)methanol (8h)

Scale, physical description, yield, mass: 5.0 mmol, colorless oil, 61% yield, 1.0 gr (3.0 mmol).

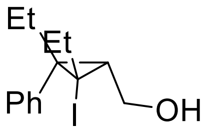

^{1}H-NMR (400 MHz, CDCl$_3$): δ 7.40 – 7.28 (m, 2H), 7.23 (d, J = 7.4 Hz, 1H), 7.12 (d, J = 7.3 Hz, 2H), 4.05 (ddd, J = 11.7, 8.8, 5.8 Hz, 1H), 3.55 – 3.38 (m, 1H), 2.12 (ddt, J = 31.2, 15.0, 7.5 Hz, 2H), 1.77 (dt, J = 14.4, 7.2 Hz, 2H), 1.59 (dq, J = 14.3, 7.3 Hz, 1H), 1.24 (t, J = 7.1 Hz, 3H), 0.83 (t, J = 7.3 Hz, 3H), 0.73 (dd, J = 8.6, 5.8 Hz, 1H).

^{13}C-NMR (101 MHz, CDCl$_3$): δ 142.20, 131.43, 127.86, 126.72, 68.04, 42.33, 38.40, 36.36, 36.09, 31.00, 14.71, 11.77.

HRMS (APCI): m/z calculated for $C_{14}H_{18}I$ $[M-OH]^+$: 313.0448, found 313.0438.

2-Iodo-3-methyl-2,3-diphenylcyclopropyl)methanolbenzene (8i)

Scale, physical description, yield, mass: 4.0 mmol, colorless oil, 76% yield, 1.1 gr (3.0 mmol).

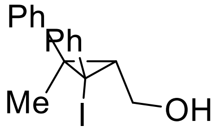

^{1}H-NMR (400 MHz, CDCl$_3$): δ 7.34 – 7.28 (m, 2H), 7.21 (d, J = 8.3 Hz, 2H), 7.16 – 6.92 (m, 6H), 4.25 (q, J = 5.6, 5.1 Hz, 1H), 4.10 (dd, J = 11.7, 7.9 Hz, 1H), 2.87 (s, 1H), 2.12 (dd, J = 7.8, 6.0 Hz, 1H), 1.86 (s, 3H).

^{13}C-NMR (101 MHz, CDCl$_3$): δ 144.78, 140.65, 129.83, 128.02, 127.91, 127.73, 127.00, 126.30, 66.20, 34.91, 31.57, 31.17, 26.69.

HRMS (APCI): m/z calculated for $C_{17}H_{16}I$ $[M-OH]^+$: 347.0291, found 347.0278.

2-Ethyl-3-iodo-2-methyl-3-phenylcyclopropyl)methanol (8j)

Scale, physical description, yield, mass: 5.7 mmol, colorless oil, 73% yield, 1.33 mg (4.2 mmol).

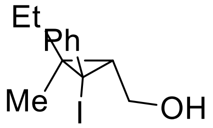

^{1}H-NMR (400 MHz, CDCl$_3$) : δ 7.36 (d, J = 7.2 Hz, 2H), 7.29 (d, J = 7.4 Hz, 2H), 7.21 – 7.14 (m, 1H), 3.98 (ddd, J = 11.9, 8.2, 6.0 Hz, 1H), 3.77 (ddd, J = 11.9, 8.5, 4.6 Hz, 1H), 1.70 (dd, J = 8.2, 4.6 Hz, 1H), 1.40 (s, 3H), 1.39 – 1.32 (m, 1H), 0.98 (dd, J = 8.5, 6.1 Hzzz, 1H), 0.88 (t, J = 7.3 Hz, 3H), 0.68 (dq, J = 14.8, 7.4 Hz, 1H).

^{13}C-NMR (101 MHz, CDCl$_3$): δ 145.97, 129.64, 128.48, 127.47, 66.55, 33.45, 33.08, 31.01, 30.35, 21.96, 11.58.

HRMS (APCI): m/z calculated for $C_{13}H_{16}I$ $[M-OH]^+$: 299.0291, found 299.0273.

2-Butyl-2-ethyl-3-iodo-3-(trimethylsilyl)cyclopropyl)methanol (8k)

Scale, physical description, yield, mass: 2.5 mmol, colorless oil, 78% yield, 700 mg (1.97 mmol).

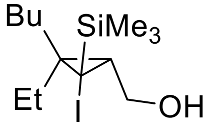

^{1}H-NMR (400 MHz, CDCl$_3$): δ 3.68 (dd, J = 10.3, 7.1 Hz, 1H), 3.46 (dd, J = 10.2, 8.1 Hz, 1H), 1.83 (dq, J = 14.7, 7.4 Hz, 1H), 1.63 (ddd, J = 13.1, 9.4, 5.2 Hz, 1H), 1.48 (dt, J = 14.5, 7.3 Hz, 1H), 1.38 – 1.17 (m, 5H), 0.91 (dt, J = 13.6, 7.0 Hz, 6H), 0.80 (t, J = 7.6 Hz, 1H), 0.23 (s, 9H).

^{13}C-NMR (101 MHz, CDCl$_3$): δ 65.80, 33.85, 32.59, 32.41, 29.36, 28.80, 28.60, 23.05, 14.29, 10.35, 1.55.

HRMS (APCI): m/z calculated for $C_{13}H_{28}IOSi$ $[M+H]^+$: 355.0949, found 355.0947.

2-Butyl-3-iodo-2-((E)-5-methylhex-3-en-1-yl)-3-(trimethylsilyl)cyclopropyl)methanol (8l)

Scale, physical description, yield, mass: 2.5 mmol, colorless oil, 62% yield, 660 mg (1.56 mmol).

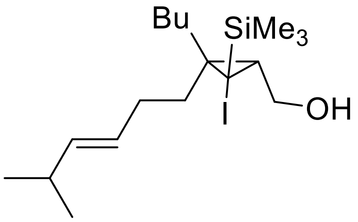

^{1}H-NMR (400 MHz, CDCl$_3$): δ 5.41 (q, J = 6.1 Hz, 2H), 3.92 (dt, J = 12.6, 6.4 Hz, 1H), 3.66 (t, J = 10.2 Hz, 1H), 2.12 – 1.93 (m, 2H), 1.86 (t, J = 6.3 Hz, 2H), 1.75 (ddd, J = 14.0, 11.2, 5.6 Hz, 1H), 1.58 (d, J = 5.9 Hz, 1H), 1.54 – 1.48 (m, 1H), 1.34 (d, J = 14.5 Hz, 1H), 1.28 – 1.16 (m, 3H), 0.88 (dd, J = 14.2, 6.7 Hz, 9H), 0.60 (dd, J = 8.5, 5.7 Hz, 1H), 0.23 (s, 9H).

^{13}C-NMR (101 MHz, CDCl$_3$): δ 130.66, 129.77, 65.83, 42.09, 35.69, 33.03, 32.93, 32.61, 29.41, 29.13, 28.59, 23.02, 22.46, 22.43, 14.28, 1.56.

HRMS (APCI): m/z calculated for $C_{18}H_{34}IOSi$ $[M-H]^+$: 421.1418, found 421.1414.

(2-butyl-3-iodo-2-methyl-1-phenylcyclopropyl)methanol (8m)

Scale, physical description, yield, mass: 2.96 mmol, colorless oil, 75% yield, 730 mg (2.21 mmol).

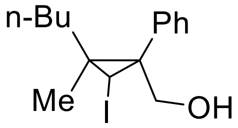

Racemic sample. Product matched the previously reported spectra.[5]

((1R,2S,3S)-2-butyl-3-iodo-2-methyl-1-phenylcyclopropyl)methanol (8m*)

Scale, physical description, yield, mass: 1.97 mmol, colorless oil, 75% yield, 513 mg (1.49 mmol).

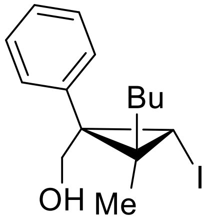

^{1}H-NMR (400 MHz, $CDCl_3$): δ 7.36 – 7.29 (m, 2H), 7.25 (tq, J = 4.8, 1.9, 1.5 Hz, 3H), 3.98 (d, J = 11.9 Hz, 1H), 3.80 (d, J = 11.9 Hz, 1H), 3.08 (s, 1H), 1.44 – 1.30 (m, 2H), 1.27 (s, 3H), 1.26 – 1.22 (m, 1H), 1.16 – 0.97 (m, 2H), 0.75 (t, J = 7.3 Hz, 3H), 0.70 – 0.58 (m, 1H).

^{13}C-NMR (101 MHz, $CDCl_3$): δ 139.42, 129.87, 128.67, 127.24, 69.72, 39.08, 37.49, 28.96, 28.40, 22.89, 19.23, 14.18, 13.45.

The enantiomeric ratio was determined by HPLC analysis using CHIRACEL OX-H, column size: 0.46cmI.D x 25 cmL (99:1 n-Hexane/IPA, 1 mL/min, 20 °C, 210 nm, τ minor = 25.85 min, τ major = 26.89 min).

2-Allyl-2-butyl-3-iodo-1-phenylcyclopropyl)methanol (8n)

Scale, physical description, yield, mass: 2.96 mmol, colorless oil, 78% yield, 850 mg (2.29 mmol).

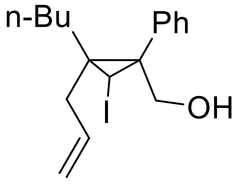

^{1}H-NMR (400 MHz, $CDCl_3$): δ 7.34 (t, J = 7.4 Hz, 2H), 7.27 (d, J = 6.9 Hz, 1H), 7.25 – 7.17 (m, 2H), 5.94 (ddt, J = 17.2, 10.1, 7.0 Hz, 1H), 5.28 – 5.11 (m, 2H), 4.05 (d, J = 11.9 Hz, 1H), 3.86 (d, J = 11.9 Hz, 1H), 3.10 (s, 1H), 2.44 (dd, J = 15.2, 7.6 Hz, 1H), 2.29 (dd, J = 15.3, 6.3 Hz, 1H), 1.64 (ddd, J = 14.4, 10.3, 4.4 Hz, 1H), 1.42 – 1.01 (m, 5H), 0.77 (t, J = 7.2 Hz, 3H), 0.39 (ddd, J = 15.4, 10.8, 5.6 Hz, 1H).

^{13}C-NMR (101 MHz, $CDCl_3$): δ 139.10, 135.59, 129.93, 128.66, 127.26, 117.64, 69.42, 39.51, 37.23, 34.04, 32.06, 27.90, 22.82, 14.22, 11.70.

HRMS (APCI): m/z calculated for $C_{17}H_{22}I$ $[M-OH]^+$: 353.0761, found 353.0744.

2.4 General procedure for synthesis of hexasubstituted iodocyclopropane methanol derivatives.

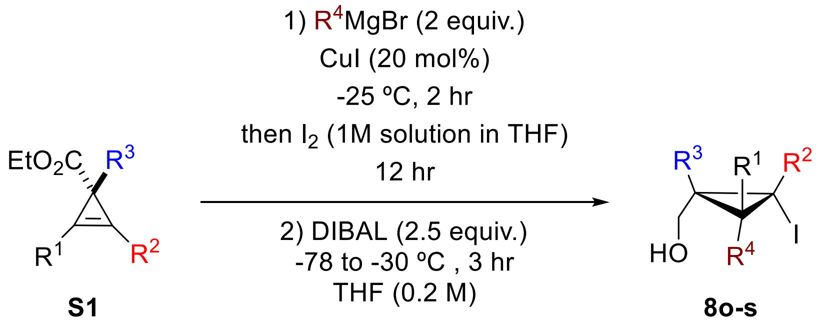

S1 **8o-s**

Step A: In a flame dried three neck round bottom flask, copper iodide (0.2 equiv.) was taken and purged with argon three times. Et_2O (0.1 M) was added through a septum followed by the addition of cyclopropene (**4**, 1.0 equiv.). The solution was cooled to -30 °C, followed by the dropwise addition of a Grignard reagent (2.0 equiv.). The solution was stirred at -25 °C for two hours. Upon full consumption of the cyclopropene (followed by TLC $KMnO_4$ or p-anisaldehyde), the reaction mixture was cooled to -30 °C, followed by slow canulation of iodine solution (1 M) in THF (5 equiv.). This reaction mixture was stirred at -25 °C for 12 hours and the reaction was quenched with 1:1 saturated solution of NH_4Cl and NH_4OH. This mixture was directly transferred to a separation funnel with a saturated solution of $Na_2S_2O_4$ and extracted with Et_2O (3 × 20 mL/mmol). The combined organic layers were dried with Na_2SO_4, filtered, and concentrated in vacuo. The crude product was taken to the next step after a quick column. The product is often accompanied by hydrolysis product which is separated at the next step.

Step B: In a flame dried three neck round bottom flask, the product from step A (1 equiv.) was taken in THF (0.5 M) and cooled to -78 °C. To this solution DIBAL-H (2.5 equiv. 1 M in hexane) was added dropwise. The solution was stirred at -30 °C until full consumption of the cyclopropyl ester (followed by TLC $KMnO_4$ or p-anisaldehyde). The reaction mixture was slowly diluted with Et_2O and was quenched with 1 M HCl solution. The resulting solution was stirred until two separated phases appeared. The organic phase was separated, and the aqueous phase was extracted with Et_2O (3 × 20 mL/mmol). The combined organic layers were dried with Na_2SO_4, filtered, and concentrated in vacuo. The crude product was further purified by silica gel column chromatography to obtain the desired product **8o-s**.

2-Butyl-3-iodo-1,2-dimethyl-3-phenylcyclopropyl)methanol (8o)

Scale, physical description, yield, mass: 1.6 mmol, colorless oil, 42% yield, 240 mg (0.67 mmol).

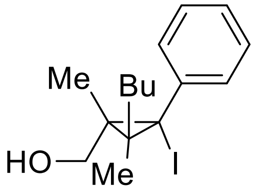

^{1}H-NMR (400 MHz, CDCl$_3$): δ 7.33 – 7.27 (m, 3H), 7.25 (d, J = 2.0 Hz, 1H), 7.17 (tt, J = 7.3, 2.0 Hz, 1H), 3.94 – 3.82 (m, 2H), 1.60 (s, 1H), 1.53 (s, 3H), 1.45 (s, 1H), 1.20 (s, 3H), 1.18 – 1.03 (m, 3H), 0.90 – 0.85 (m, 1H), 0.83 (t, J = 7.2 Hz, 3H).

^{13}C-NMR (101 MHz, CDCl$_3$): δ 144.43, 130.59, 128.44, 127.09, 72.83, 38.41, 34.27, 32.96, 31.54, 30.16, 24.77, 23.47, 14.85, 14.21.

HRMS (APCI): m/z calculated for $C_{16}H_{22}I$ [M-OH]$^+$: 341.0761, found 341.0762.

2-Butyl-3-(4-fluorophenyl)-3-iodo-1,2-dimethylcyclopropyl)methanol (8p)

Scale, physical description, yield, mass: 2.7 mmol, colorless oil, 55% yield, 230 mg (0.61 mmol).

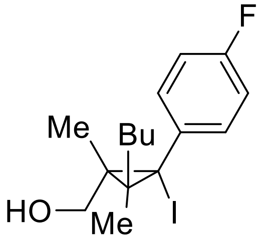

^{1}H-NMR (400 MHz, CDCl$_3$): δ 7.29 (d, J = 3.8 Hz, 1H), 7.25 (s, 1H), 7.04 – 6.95 (m, 2H), 3.90 (d, J = 2.3 Hz, 2H), 3.13 – 2.97 (m, 1H), 1.58 (d, J = 4.9 Hz, 1H), 1.54 (s, 3H), 1.52 – 1.44 (m, 1H), 1.22 (s, 3H), 1.20 – 0.98 (m, 3H), 0.90 (ddd, J = 7.9, 4.8, 2.2 Hz, 1H), 0.85 (t, J = 7.1 Hz, 3H).

^{13}C-NMR (101 MHz, CDCl$_3$): δ 161.48 (d, J = 247.3 Hz), 140.41 (d, J = 3.6 Hz), 132.27, 115.43 (d, J = 21.7 Hz), 72.70, 37.07, 34.16, 32.85, 31.56, 30.05, 24.66, 23.42, 14.77, 14.15.

^{19}F- NMR (377 MHz, CDCl3): δ -114.42.

HRMS (APCI): m/z calculated for $C_{16}H_{22}FO$ [M-I]$^+$: 249.1649, found 249.1645.

2-Butyl-3-(3-chlorophenyl)-3-iodo-1,2-dimethylcyclopropyl)methanolmethanol (8q)

Scale, physical description, yield, mass: 2.05 mmol, colorless oil, 26% yield, 212 mg (0.54 mmol).

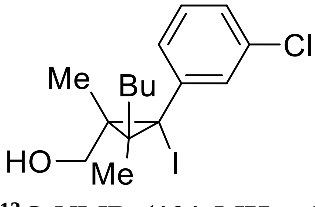

^{1}H-NMR (400 MHz, CDCl$_3$): δ 7.33 – 7.25 (m, 2H), 7.20 (d, J = 7.9 Hz, 2H), 3.87 (d, J = 10.4 Hz, 1H), 3.74 (d, J = 10.3 Hz, 1H), 1.62 (d, J = 11.8 Hz, 1H), 1.59 (d, J = 1.7 Hz, 3H), 1.47 (d, J = 1.8 Hz, 1H), 1.30 (s, 3H), 1.28 – 1.22 (m, 3H), 1.14 (t, J = 12.9 Hz, 1H), 0.89 (t, J = 6.8 Hz, 3H).

^{13}C-NMR (101 MHz, CDCl$_3$): δ 146.14, 133.86, 129.73, 127.34, 72.58, 36.21, 34.12, 32.97, 31.58, 30.12, 24.53, 23.44, 14.76, 14.20.

HRMS (APCI): m/z calculated for $C_{16}H_{21}ClI$ [M-OH]$^+$: 375.0371, found 375.1354.

2-Butyl-3-(hydroxymethyl)-1-iodo-2,3-dimethylcyclopropyl)benzonitrile (8r)

Scale, physical description, yield, mass: 3.00 mmol, colorless oil, 13% yield, 155 mg (0.4 mmol).

The reaction was a mixture of two products (1:2.2) = (**8r:8r***).

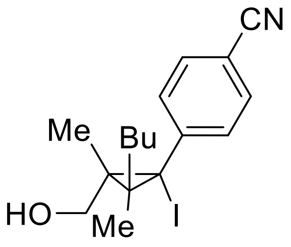

8r

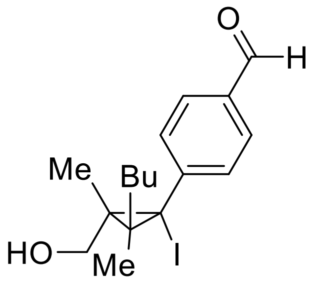

8r*

^{1}H-NMR (400 MHz, CDCl$_3$): δ 9.97 (s, 1H), 7.81 (d, J = 8.1 Hz, 1H), 7.58 (d, J = 8.1 Hz, 1H), 7.41 (d, J = 8.0 Hz, 1H), 7.34 (d, J = 8.2 Hz, 1H), 3.92 – 3.80 (m, 2H), 1.56 – 1.45 (m, 5H), 1.18 (d, J = 9.3 Hz, 7H), 1.04 – 0.93 (m, 1H), 0.81 (td, J = 7.1, 2.0 Hz, 4H).

^{13}C-NMR (101 MHz, CDCl$_3$): δ 191.82, 150.86, 149.31, 134.75, 132.30, 131.21, 129.92, 110.62, 72.39, 72.24, 65.96, 35.95, 35.03, 34.22, 34.18, 32.84, 31.54, 31.53, 30.02, 29.99, 24.41, 24.33, 23.36, 15.36, 14.75, 14.69, 14.11.

HRMS (APCI): m/z calculated for $C_{17}H_{23}INO$ [M+H]$^+$: 384.0819, found 384.0817.

2-Butyl-3-(4-chlorophenyl)-3-iodo-1,2-dimethylcyclopropyl)methanol (8s)

Scale, physical description, yield, mass: 17.04 mmol, colorless oil, 59% yield, 3.97 gr (10.12 mmol).

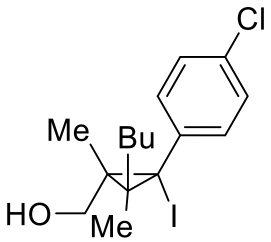

^{1}H-NMR (400 MHz, CDCl$_3$): δ 7.33 – 7.25 (m, 2H), 7.19 (d, J = 8.2 Hz, 2H), 3.85 (d, J = 5.8 Hz, 2H), 1.88 (bs, 1H), 1.77 – 1.64 (m, 1H), 1.51 (s, 3H), 1.41 (s, 1H), 1.18 (m, 5H), 1.09 – 0.96 (m, 2H), 0.83 (t, J = 7.1 Hz, 3H).

^{13}C-NMR (101 MHz, CDCl$_3$): δ 142.97, 132.86, 131.90, 128.74, 72.61, 36.64, 34.20, 32.98, 31.59, 30.10, 24.60, 23.44, 14.73, 14.19.

HRMS (APCI): m/z calculated for $C_{16}H_{20}ClO$ [M-IH$_2$]$^+$: 263.1197, found 263.1199.

2.5 General procedure for synthesis of secondary iodocyclopropyl ethanol derivatives.

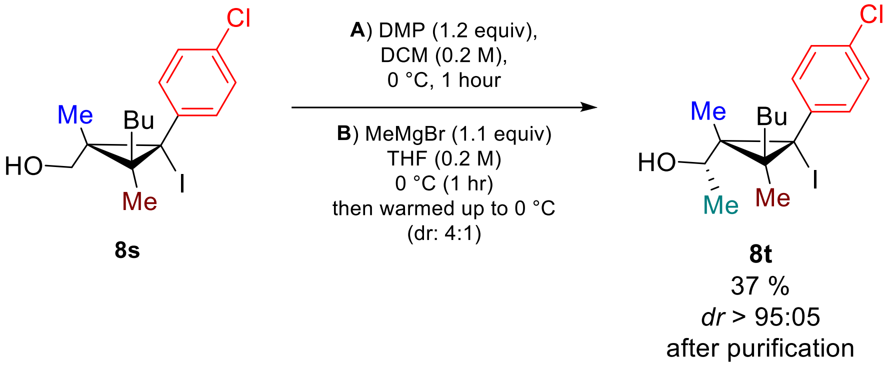

Step A: In a oven dried round bottom flask, cyclopropane (**8s**, 1.0 equiv.) was dissolved in DCM (0.1 M) and cooled to 0 °C. Dess Martin periodinane (DMP) (1.2 equiv.) was added to this solution, in one portion. The reaction mixture was stirred at 0 °C for one hour. Upon completion, the reaction mixture was directly poured into a separation funnel and washed with (1:1) saturated solution of $NaHCO_3$ and $Na_2S_2O_4$ (3 × 10mL/mmol). The organic layer was dried with Na_2SO_4, filtered, and concentrated in vacuo. The crude product was taken to the next step.

Step B: In a flame dried three neck round bottom flask, the product from step A (1 equiv.) was taken in THF (0.2 M) and cooled to 0 °C. To this solution, MeMgBr (1.1 equiv. 2.1 M in Et_2O) was added dropwise. This solution was stirred at 0 °C until the full consumption of the aldehyde (followed by TLC $KMnO_4$ or p-anisaldehyde). The reaction mixture was slowly diluted with Et_2O and was quenched with 1 M HCl solution. The organic phase was separated, and the aqueous phase was extracted with Et_2O (3 × 20 mL/mmol). The combined organic layer was dried with Na_2SO_4, filtered, and concentrated in vacuo. The crude product was further purified on silica gel column chromatography to obtain the desired product **8t.**

1-(-2-butyl-3-(4-chlorophenyl)-3-iodo-1,2-dimethylcyclopropyl)ethan-1-ol (8t)

Scale, physical description, yield, mass: 5.00 mmol, colorless oil, 37% yield, 719 mg (1.84 mmol).

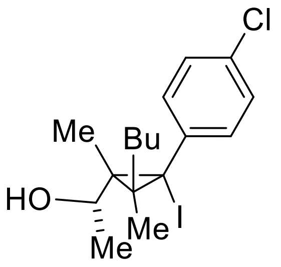

^{1}H-NMR (400 MHz, $CDCl_3$) : δ 7.20 (d, J = 1.9 Hz, 1H), 7.17 (d, J = 10.3 Hz, 1H), 7.06 (d, J = 8.1 Hz, 2H), 3.92 (q, J = 6.2 Hz, 1H), 1.52 (s, 3H), 1.45 (tdd, J = 12.4, 5.9, 1.9 Hz, 2H), 1.37 (d, J = 6.4 Hz, 3H), 1.31 – 1.22 (m, 1H), 1.17 – 1.03 (m, 3H), 0.95 (s, 3H), 0.77 (t, J = 7.1 Hz, 3H).

^{13}C-NMR (101 MHz, $CDCl_3$): δ 143.16, 132.73, 131.68, 128.72, 75.49, 35.71, 34.43, 34.20, 31.78, 30.03, 24.82, 23.46, 18.62, 14.19, 10.05.

HRMS (APCI): m/z calculated for $C_{17}H_{24}ClO$ $[M-IH_2]^+$: 279.1510, found 279.1527.

2.6 General procedure for the synthesis of bromomethyl-iodocyclopropane derivatives.

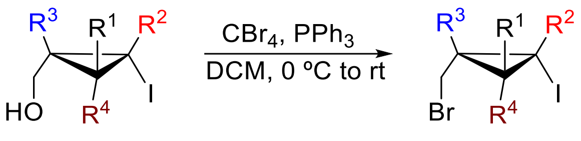

In an oven dried round bottom flask, a stirred solution of iodocyclopropanes (**8,** 1.0 equiv.) in DCM (0.2 M) was cooled to 0 °C (ice bath). After 5 minutes, CBr_4 (1.5 equiv.) and $P(Ph)_3$ (1.5 equiv) were added in one batch. After 15 minutes, the reaction mixture was slowly warmed up to rt over three hours. Petroleum ether (50 mL/mmol) was added to the flask, followed by filtration of the resulting white suspension over silica. The resulting filtrate was concentrated in vacuo and was further purified by silica gel chromatography to obtain pure bromomethyl-iodocyclopropane derivatives **9**. (Product usually comes with 1-2% Et_2O/Petroleum ether, it usually turns to pale brown over time)

2-(Bromomethyl)-1-butyl-3-iodo-1-methylcyclopropane (9a)

Scale, physical description, yield, mass: 2.11 mmol, colorless oil, 94% yield, 655 mg (1.98 mmol).

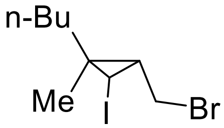

^{1}H-NMR (400 MHz, $CDCl_3$): δ 3.45 – 3.31 (m, 2H), 2.76 (d, J = 7.9 Hz, 1H), 1.40 – 1.24 (m, 6H), 1.12 (s, 3H), 1.11 – 1.05 (m, 1H), 0.89 (t, J = 7.1 Hz, 3H).

^{13}C-NMR (101 MHz, $CDCl_3$): δ 40.74, 36.46, 28.65, 27.91, 26.16, 22.92, 18.11, 14.21, 10.63.

HRMS (APCI): m/z calculated for $C_9H_{16}Br$ $[M-I]^+$: 203.0430, found 203.0414.

2-(Bromomethyl)-1-butyl-3-iodocyclopropyl)ethyl)benzene (9b)

Scale, physical description, yield, mass: 0.56 mmol, colorless oil, 55% yield, 129 mg (0.3 mmol).

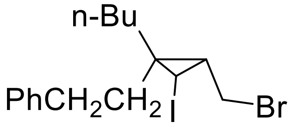

^{1}H-NMR (400 MHz, $CDCl_3$): δ 7.31 (t, J = 7.5 Hz, 2H), 7.26 – 7.18 (m, 3H), 3.44 – 3.30 (m, 2H), 2.77 (d, J = 7.8 Hz, 1H), 2.76 – 2.61 (m, 2H), 1.87 (ddd, J = 14.3, 12.1, 5.6 Hz, 1H), 1.66 (ddd, J = 13.8, 9.6, 6.4 Hz, 1H), 1.61 – 1.52 (m, 1H), 1.42 (dt, J = 8.4, 3.2 Hz, 2H), 1.38 – 1.29 (m, 2H), 1.21 (ddd, J = 13.9, 9.6, 5.9 Hz, 1H), 1.12 (q, J = 7.8 Hz, 1H), 0.93 (t, J = 7.2 Hz, 3H).

^{13}C-NMR (101 MHz, $CDCl_3$): δ 142.11, 128.60, 128.52, 126.20, 36.96, 35.74, 33.53, 31.69, 29.23, 28.85, 28.41, 23.00, 14.22, 9.21.

HRMS (APCI): m/z calculated for $C_{16}H_{22}Br$ $[M-I]^+$: 293.0899, found 293.0913.

(2-((Bromomethyl)-3-iodo-1-methylcyclopropyl)ethyl)benzene (9c)

Scale, physical description, yield, mass: 2.75 mmol, colorless oil, 93% yield, 966 mg (2.55 mmol).

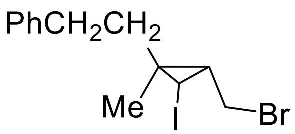

^{1}H-NMR (400 MHz, CDCl$_3$): δ 7.28 (dd, J = 8.1, 6.6 Hz, 2H), 7.23 – 7.13 (m, 3H), 3.45 – 3.28 (m, 2H), 2.79 – 2.67 (m, 3H), 1.79 – 1.60 (m, 2H), 1.22 (s, 3H), 1.10 (q, J = 8.0 Hz, 1H).

^{13}C-NMR (101 MHz, CDCl$_3$): δ 141.73, 128.62, 128.41, 126.13, 43.01, 36.20, 32.89, 28.09, 25.99, 18.13, 10.20.

HRMS (APCI): m/z calculated for $C_{13}H_{16}Br$ [M-I]$^+$: 251.0430, found 251.0438.

(2-(2-(Bromomethyl)-3-iodo-1-vinylcyclopropyl)ethyl)benzene (9d)

Scale, physical description, yield, mass: 1.16 mmol, colorless oil, 49% yield, 224 mg (0.57 mmol).

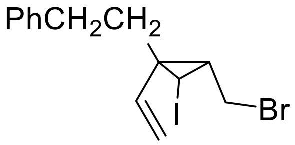

^{1}H-NMR (400 MHz, CDCl$_3$): δ 7.32 – 7.26 (m, 2H), 7.23 – 7.17 (m, 1H), 7.17 – 7.11 (m, 2H), 5.66 (dd, J = 17.4, 10.8 Hz, 1H), 5.48 (dd, J = 10.9, 1.3 Hz, 1H), 5.38 (dd, J = 17.4, 1.3 Hz, 1H), 3.43 (dd, J = 10.5, 6.9 Hz, 1H), 3.29 (dd, J = 10.5, 8.3 Hz, 1H), 2.86 (d, J = 7.9 Hz, 1H), 2.73 (dd, J = 9.3, 6.8 Hz, 2H), 2.06 – 1.94 (m, 1H), 1.81 – 1.68 (m, 1H), 1.32 (td, J = 8.2, 7.0 Hz, 1H).

^{13}C-NMR (101 MHz, CDCl$_3$): δ 141.65, 136.90, 128.65, 128.42, 126.17, 119.96, 40.86, 36.12, 32.61, 31.67, 30.28, 9.48.

HRMS (APCI): m/z calculated for $C_{14}H_{18}Br$ [M-I+H$_2$]$^+$: 265.0586, found 265.0564.

2-(bromomethyl)-1-butyl-1-(3-chloropropyl)-3-iodocyclopropane (9e)

Scale, physical description, yield, mass: 1.69 mmol, colorless oil, 70% yield, 470 mg (1.19 mmol).

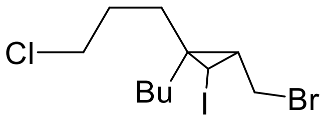

^{1}H-NMR (400 MHz, CDCl$_3$): δ 3.54 (h, J = 4.6 Hz, 2H), 3.49 – 3.33 (m, 2H), 2.75 (d, J = 8.0 Hz, 1H), 1.89 – 1.80 (m, 2H), 1.68 (dt, J = 13.9, 7.9 Hz, 1H), 1.55 – 1.50 (m, 1H), 1.43 – 1.20 (m, 6H), 1.11 (q, J = 7.9 Hz, 1H), 0.94 (t, J = 6.8 Hz, 3H).

^{13}C-NMR (101 MHz, CDCl$_3$): δ 44.96, 35.67, 34.24, 30.29, 29.30, 28.62, 28.59, 27.48, 23.19, 14.27, 8.98

HRMS (APCI): m/z calculated for $C_{11}H_{19}BrCl$ [M-I]$^+$: 265.0353, found 265.0349.

3-(Bromomethyl)-1,2-diethyl-1-iodo-2-methylcyclopropane (9f)

Scale, physical description, yield, mass: 3.31 mmol, colorless oil, 90% yield, 990 mg (2.99 mmol).

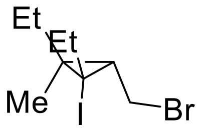

^{1}H-NMR (400 MHz, CDCl$_3$): δ 3.45 (d, J = 7.8 Hz, 2H), 1.97 (dq, J = 14.3, 7.1 Hz, 1H), 1.84 (dq, J = 14.5, 7.1 Hz, 1H), 1.50 (qd, J = 7.2, 3.8 Hz, 2H), 1.25 (s, 3H), 1.10 (t, J = 7.1 Hz, 3H), 1.01 (t, J = 7.4 Hz, 3H), 0.55 (t, J = 7.8 Hz, 1H).

^{13}C-NMR (101 MHz, CDCl$_3$): δ 42.95, 38.16, 35.72, 35.16, 32.86, 28.39, 23.15, 14.78, 11.36.

HRMS (APCI): m/z calculated for $C_9H_{16}Br$ [M-I]$^+$: 203.0430, found 203.0439.

(2-(3-(Bromomethyl)-1,2-diethyl-2-iodocyclopropyl)ethyl)benzene (9g)

Scale, physical description, yield, mass: 2.09 mmol, colorless oil, 68% yield, 600 mg (1.42 mmol).

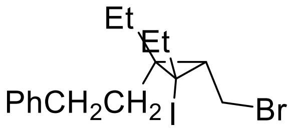

^{1}H-NMR (400 MHz, CDCl$_3$): δ 7.29 (d, J = 8.8 Hz, 4H), 7.24 – 7.16 (m, 1H), 3.46 (dd, J = 7.9, 1.7 Hz, 2H), 2.68 (ddq, J = 25.4, 13.1, 6.8, 5.4 Hz, 2H), 2.20 – 1.98 (m, 2H), 1.90 (dp, J = 13.8, 6.7 Hz, 2H), 1.60 (dd, J = 12.7, 4.4 Hz, 1H), 1.36 – 1.19 (m, 1H), 1.14 (td, J = 7.2, 1.7 Hz, 3H), 1.08 (td, J = 7.4, 1.7 Hz, 3H), 0.64 (td, J = 7.8, 1.7 Hz, 1H).

^{13}C-NMR (101 MHz, CDCl$_3$): δ 142.21, 128.56, 128.53, 126.14, 41.91, 37.41, 36.95, 36.25, 35.96, 35.55, 31.53, 23.84, 14.55, 11.29.

HRMS (APCI): m/z calculated for $C_{16}H_{22}Br$ [M-I]$^+$: 293.0899 found 293.0919.

(3-(Bromomethyl)-1,2-diethyl-2-iodocyclopropyl)benzene (9h):

Scale, physical description, yield, mass: 3.03 mmol, colorless oil, 22% yield, 270 mg (0.68 mmol). Minor amount of BHT is present.

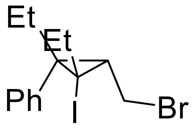

^{1}H-NMR (400 MHz, CDCl$_3$): δ 7.33 (dd, J = 7.9, 6.5 Hz, 2H), 7.25 – 7.23 (m, 1H), 7.23 – 7.17 (m, 2H), 3.77 (dd, J = 10.3, 6.4 Hz, 1H), 3.28 (dd, J = 10.3, 8.2 Hz, 1H), 2.15 (dt, J = 14.6, 7.3 Hz, 1H), 2.11 – 2.01 (m, 1H), 1.87 – 1.75 (m, 1H), 1.66 – 1.55 (m, 1H), 1.25 (t, J = 7.1 Hz, 3H), 0.95 (dd, J = 8.2, 6.4 Hz, 1H), 0.85 (t, J = 7.3 Hz, 3H).

^{13}C-NMR (101 MHz, CDCl$_3$): δ 141.89, 131.30, 128.04, 126.95, 39.97, 39.80, 36.98, 35.83, 30.98, 30.47, 14.67, 11.86.

HRMS (APCI): m/z calculated for $C_{14}H_{18}Br$ [M-I]$^+$: 265.0586, found 265.0616.

3-(Bromomethyl)-1-iodo-2-methylcyclopropane-1,2-diyl)dibenzene (9i)

Scale, physical description, yield, mass: 3.02 mmol, white solid, 94% yield, 1.21 gr (2.83 mmol).

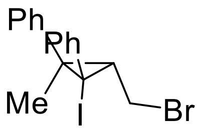

^{1}H-NMR (400 MHz, CDCl$_3$): δ 7.31 – 7.24 (m, 2H), 7.20 (d, J = 7.2 Hz, 2H), 7.14 (t, J = 7.6 Hz, 2H), 7.07 (dt, J = 9.2, 7.3 Hz, 3H), 6.99 (t, J = 7.3 Hz, 1H), 3.83 (qd, J = 10.5, 7.7 Hz, 2H), 2.27 (t, J = 7.7 Hz, 1H), 1.83 (s, 3H).

^{13}C-NMR (101 MHz, CDCl$_3$): δ 144.53, 140.42, 129.76, 128.19, 128.12, 127.94, 127.32, 126.64, 37.45, 37.11, 32.50, 32.09, 26.63.

HRMS (APCI): m/z calculated for $C_{17}H_{16}Br$ [M-I]$^+$: 299.0430, found 299.0450.

3-(Bromomethyl)-2-ethyl-1-iodo-2-methylcyclopropyl)benzene (9j)

Scale, physical description, yield, mass: 4.2 mmol, colorless oil, 88% yield, 1.4 gr (3.69 mmol).

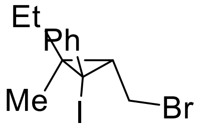

^{1}H-NMR (400 MHz, CDCl$_3$): δ 7.38 – 7.33 (m, 2H), 7.29 (d, J = 7.3 Hz, 2H), 7.22 – 7.15 (m, 1H), 3.66 (dd, J = 10.4, 7.3 Hz, 1H), 3.57 (dd, J = 10.4, 8.3 Hz, 1H), 1.47 – 1.42 (m, 1H), 1.41 (s, 3H), 1.17 (dd, J = 8.2, 7.3 Hz, 1H), 0.91 (t, J = 7.3 Hz, 3H), 0.70 (dq, J = 14.7, 7.4 Hz, 1H).

^{13}C-NMR (101 MHz, CDCl$_3$): δ 145.60, 129.37, 128.53, 127.57, 38.06, 34.35, 33.95, 33.34, 30.21, 21.58, 11.52.

HRMS (APCI): m/z calculated for $C_{13}H_{16}Br$ [M-I]$^+$: 251.0430, found 251.0422.

3-(Bromomethyl)-2-butyl-2-ethyl-1-iodocyclopropyl)trimethylsilane (9k)

Scale, physical description, yield, mass: 1.97 mmol, colorless oil, 94% yield, 776 mg (1.86 mmol).

^{1}H-NMR (400 MHz, CDCl$_3$): δ 3.68 (dd, J = 10.3, 7.1 Hz, 1H), 3.46 (dd, J = 10.2, 8.1 Hz, 1H), 1.83 (dq, J = 14.7, 7.4 Hz, 1H), 1.63 (ddd, J = 13.1, 9.4, 5.2 Hz, 1H), 1.49 (dq, J = 14.5, 7.4 Hz, 1H), 1.37 – 1.16 (m, 5H), 0.91 (dt, J = 13.6, 7.0 Hz, 6H), 0.80 (t, J = 7.6 Hz, 1H), 0.23 (s, 9H).

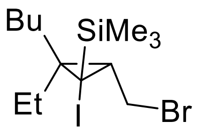

^{13}C-NMR (101 MHz, CDCl$_3$): δ 38.54, 35.85, 32.71, 32.33, 29.73, 29.30, 28.01, 23.14, 14.24, 9.87, 1.62.

^{29}Si-NMR (80 MHz, CDCl3): δ 7.35.

HRMS (APCI): m/z calculated for $C_{13}H_{26}BrSi$ [M-I]$^+$: 289.0982, found 289.0974.

3-(Bromomethyl)-2-butyl-1-iodo-2-((E)-5-methylhex-3-en-1-yl)cyclopropyl)trimethylsilane (9l)

Scale, physical description, yield, mass: 1.56 mmol, colorless oil, 94% yield, 709 mg (1.46 mmol).

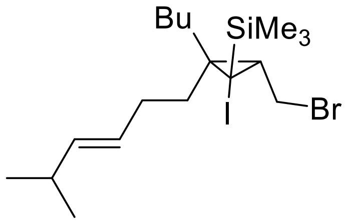

^{1}H-NMR (400 MHz, $CDCl_3$): δ 5.42 (q, J = 6.2 Hz, 2H), 3.68 (dd, J = 10.3, 7.1 Hz, 1H), 3.46 (dd, J = 10.3, 8.2 Hz, 1H), 2.11 – 2.00 (m, 2H), 1.91 – 1.77 (m, 3H), 1.67 – 1.58 (m, 1H), 1.47 (ddd, J = 15.2, 10.7, 5.5 Hz, 1H), 1.33 (dt, J = 10.7, 6.2 Hz, 2H), 1.19 (ddd, J = 15.2, 10.5, 6.5 Hz, 2H), 0.89 (dd, J = 14.7, 7.0 Hz, 9H), 0.79 (t, J = 7.6 Hz, 1H), 0.23 (s, 9H).

^{13}C-NMR (101 MHz, $CDCl_3$): δ 130.55, 129.83, 42.10, 38.49, 35.13, 35.05, 32.99, 32.74, 29.59, 29.35, 28.70, 28.59, 23.13, 22.48, 22.45, 14.24, 1.63.

^{29}Si-NMR (80 MHz, CDCl3): δ 7.42.

HRMS (APCI): m/z calculated for $C_{18}H_{34}BrSi$ $[M-I]^+$: 357.1608, found 357.1615.

1-(Bromomethyl)-2-butyl-3-iodo-2-methylcyclopropyl)benzene (9m)

Scale, physical description, yield, mass: 2.21 mmol, colorless oil, 75% yield, 650 mg (1.59 mmol).

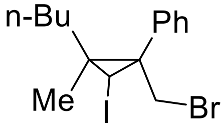

Racemic sample.

((1R,2S,3S)-1-(Bromomethyl)-2-butyl-3-iodo-2-methylcyclopropyl)benzene (9m*)

Scale, physical description, yield, mass: 1.49 mmol, colorless oil, 92% yield, 560 mg (1.37mmol).

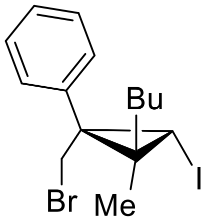

^{1}H-NMR (400 MHz, $CDCl_3$: δ 7.36 – 7.30 (m, 2H), 7.30 – 7.26 (m, 3H), 3.87 (d, J = 10.6 Hz, 1H), 3.63 (d, J = 10.6 Hz, 1H), 3.21 (s, 1H), 1.53 – 1.43 (m, 1H), 1.38 (dtt, J = 11.3, 6.7, 2.3 Hz, 1H), 1.32 (s, 3H), 1.28 – 1.18 (m, 1H), 1.10 (dddd, J = 22.1, 13.6, 7.0, 3.0 Hz, 2H), 0.77 (t, J = 7.3 Hz, 3H), 0.74 – 0.65 (m, 1H).

^{13}C-NMR (101 MHz, $CDCl_3$): δ 139.46, 130.00, 128.28, 127.39, 44.97, 37.91, 37.47, 31.98, 28.48, 22.86, 19.55, 15.90, 14.16.

HRMS (APCI): m/z calculated for $C_{15}H_{20}Br$ $[M-I]^+$: 279.0743, found 279.0744.

The enantiomeric ratio was determined by HPLC analysis using CHIRACEL OD column, size: 1cmI.D x 25 cmL (n-Hexane, 1.5 mL/min, 5 °C, 210 nm, τ major = 32.83 min, τ minor = 35.55 min).

2-Allyl-1-(bromomethyl)-2-butyl-3-iodocyclopropyl)benzene (9n)

Scale, physical description, yield, mass: 2.29 mmol, colorless oil, 83% yield, 830mg (1.91 mmol).

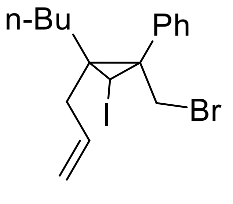

^{1}H-NMR (400 MHz, $CDCl_3$): δ 7.39 – 7.26 (m, 3H), 7.22 – 7.16 (m, 2H), 5.92 (ddt, J = 17.1, 10.4, 7.1 Hz, 1H), 5.34 – 5.13 (m, 2H), 3.98 (d, J = 10.4 Hz, 1H), 3.71 (d, J = 10.3 Hz, 1H), 3.24 (s, 1H), 2.44 (dd, J = 15.4, 7.5 Hz, 1H), 2.38 – 2.27 (m, 1H), 1.58 (ddd, J = 17.2, 8.7, 3.6 Hz, 1H), 1.33 (dddq, J = 11.0, 6.6, 4.8, 2.2 Hz, 1H), 1.27 – 0.93 (m, 4H), 0.76 (t, J = 7.2 Hz, 3H), 0.45 (ddd, J = 14.1, 11.0, 5.7 Hz, 1H).

^{13}C-NMR (101 MHz, $CDCl_3$): δ 139.03, 134.94, 129.92, 128.34, 127.39, 118.02, 44.35, 38.18, 37.50, 35.33, 34.70, 27.85, 22.78, 14.15, 13.82.

HRMS (APCI): m/z calculated for $C_{17}H_{22}Br$ $[M\text{-}I]^+$: 305.0899, found 305.0872.

2-(Bromomethyl)-3-butyl-1-iodo-2,3-dimethylcyclopropyl)benzene (9o)

Scale, physical description, yield, mass: 0.67 mmol, colorless oil, 81% yield, 230 mg (0.546 mmol).

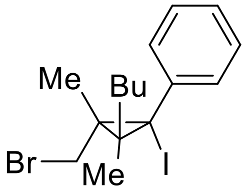

^{1}H-NMR (400 MHz, $CDCl_3$): δ 7.29 (dt, J = 11.0, 7.3 Hz, 4H), 7.18 (tt, J = 8.6, 2.1 Hz, 1H), 3.87 (dd, J = 10.4, 3.3 Hz, 1H), 3.75 (dd, J = 10.4, 3.5 Hz, 1H), 1.69 – 1.60 (m, 1H), 1.60 – 1.56 (m, 3H), 1.56 – 1.42 (m, 1H), 1.27 (s, 3H), 1.25 – 1.06 (m, 4H), 0.85 (dt, J = 7.7, 3.9 Hz, 3H).

^{13}C-NMR (101 MHz, $CDCl_3$): δ 144.28, 130.29, 128.44, 127.17, 49.19, 39.07, 34.23, 33.73, 31.45, 29.88, 25.19, 23.49, 16.81, 14.22.

HRMS (APCI): m/z calculated for $C_{16}H_{22}Br$ $[M\text{-}I]^+$: 293.0899, found 293.0875.

2-(Bromomethyl)-3-butyl-1-iodo-2,3-dimethylcyclopropyl)-4-fluorobenzene (9p)

Scale, physical description, yield, mass: 0.61 mmol, colorless oil, 93% yield, 250 mg (0.569 mmol).

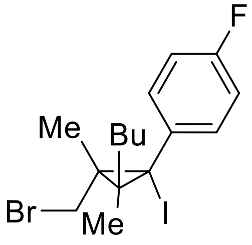

^{1}H-NMR (400 MHz, $CDCl_3$): δ 7.23 (t, J = 6.8 Hz, 2H), 6.98 (t, J = 8.5 Hz, 2H), 3.84 (d, J = 10.3 Hz, 1H), 3.71 (d, J = 10.3 Hz, 1H), 1.60 (dd, J = 10.0, 3.3 Hz, 1H), 1.55 (s, 3H), 1.53 – 1.43 (m, 1H), 1.24 (s, 3H), 1.23 – 1.15 (m, 3H), 1.09 (td, J = 12.7, 12.0, 3.5 Hz, 1H), 0.84 (t, J = 7.0 Hz, 3H).

^{13}C-NMR (101 MHz, $CDCl_3$): δ 161.57 (d, J = 247.6 Hz), 140.32 (d, J = 3.6 Hz), 132.33, 115.51 (d, J = 21.3 Hz), 48.91, 37.75, 34.23, 33.90, 31.58, 29.83, 25.09, 23.46, 16.70, 14.15.

^{19}F NMR (377 MHz, $CDCl_3$): δ -114.17.

HRMS (APCI): m/z calculated for $C_{16}H_{21}BrF$ $[M+I]^+$: 311.0805, found 311.0815.

2-(Bromomethyl)-3-butyl-1-iodo-2,3-dimethylcyclopropyl)-3-chlorobenzene (9q)

Scale, physical description, yield, mass: 0.54 mmol, colorless oil, 77% yield, 190 mg (0.41 mmol). Diethyl ether and grease is present.

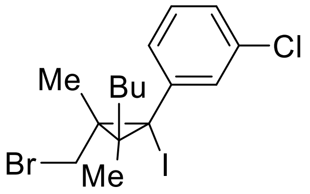

^{1}H-NMR (400 MHz, CDCl$_3$): δ 7.33 – 7.25 (m, 2H), 7.20 (d, J = 7.9 Hz, 2H), 3.87 (d, J = 10.4 Hz, 1H), 3.74 (d, J = 10.3 Hz, 1H), 1.62 (d, J = 11.8 Hz, 1H), 1.59 (d, J = 1.7 Hz, 3H), 1.47 (d, J = 1.8 Hz, 1H), 1.30 (s, 3H), 1.28 – 1.22 (m, 3H), 1.14 (t, J = 12.9 Hz, 1H), 0.89 (t, J = 6.8 Hz, 3H).

^{13}C-NMR (101 MHz, CDCl$_3$): δ 145.97, 133.88, 129.75, 127.45, 48.58, 36.94, 34.11, 33.80, 31.53, 29.84, 24.94, 23.44, 16.68, 14.16.

HRMS (APCI): m/z calculated for $C_{16}H_{22}BrClI$ [M+H]$^+$: 454.9633, found 454.9610.

2-(Bromomethyl)-3-butyl-1-iodo-2,3-dimethylcyclopropyl)benzonitrile (9r)

Scale, physical description, yield, mass: 0.404 mmol, colorless oil, 39% yield, 70 mg (0.157 mmol). Diethyl ether is present.

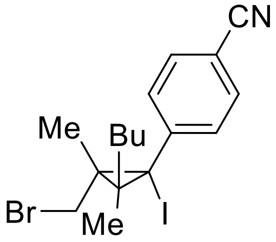

^{1}H-NMR (400 MHz, CDCl$_3$): δ 7.65 – 7.56 (m, 2H), 7.34 (d, J = 7.8 Hz, 2H), 3.82 (d, J =Z 10.4 Hz, 1H), 3.69 (d, J = 10.4 Hz, 1H), 1.61 (d, J = 10.9 Hz, 1H), 1.56 (s, 3H), 1.50 (tdt, J = 9.2, 6.5, 2.9 Hz, 1H), 1.24 (s, 3H), 1.20 – 1.16 (m, 2H), 1.03 (td, J = 12.8, 12.1, 3.4 Hz, 1H), 0.85 (q, J = 7.1 Hz, 4H).

^{13}C-NMR (101 MHz, CDCl$_3$): δ 149.07, 132.36, 130.90, 118.72, 110.86, 48.02, 35.89, 34.28, 33.94, 31.63, 29.79, 24.80, 23.40, 16.62, 14.11.

HRMS (APCI): m/z calculated for $C_{17}H_{19}BrIN$ [M-H$_2$]$^+$: 442.9740, found 442.9741.

2.7 General procedure for the synthesis of polysubstituted BCBs.

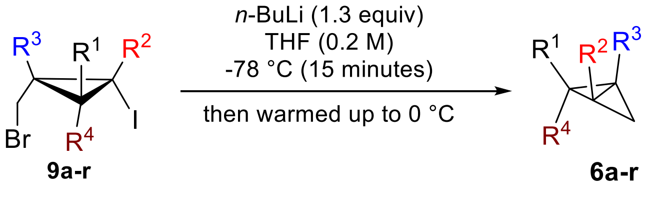

In a flame dried Schlenk tube, a stirred solution of bromomethyl iodocyclopropanes (**9,** 1.0 equiv.) in THF (0.2 M) was cooled to -78 °C (dry ice bath). After 15 minutes, n-BuLi (1.2 equiv.) was added dropwise. After 15 minutes, the reaction mixture was slowly warmed up to 0 °C over 3 hours. Water was added to hydrolyze the reaction. The mixture was directly poured into a separation funnel and was extracted with Et_2O (3 × 50 mL/mmol). The combined organic layer was dried with Na_2SO_4, filtered, and concentrated in vacuo. The NMR yields were calculated using tertbutyl methyl ether as the internal standard and crude product was further purified by Kugelrohr distillation to obtain **6**.

Note: During the recovery process of all compounds, there was occasionally grease coming from the bulb, hence the reported yield being preferentially the NMR yield. It is primordial to note that the grease did not affect subsequent transformations.

The distillation of the BCBs was conducted using a Kugelrohr apparatus. To estimate the oven temperature and pressure, we utilized the "Quest Predict™ Boiling Point Predictor" by AAT Bioquest, Inc. Link (accessed April 25, 2024). The predicted boiling point was then used to estimate the expected boiling point at reduced pressure employing a nomograph.

Below, we outline the general trends of boiling points and pressures for different types of BCBs:

The general trend of the boiling point and pressure different types of BCBs are given below:

- For BCBs weighing from 125-150 g·mol^{-1}, the oven temperature was maintained between 40 to 60 °C, the product distilled at 20 mbar of pressure and was collected by cooling the receiving flask with dry ice-acetone bath (-78 °C).
- For BCBs weighing from 150-200 g·mol^{-1}, the oven temperature was maintained between 60 to 80 °C, the product distilled at 3-4 mbar of pressure and was collected by cooling the receiving flask with ice cold water bath.
- For BCBs weighing from 200-250 g·mol^{-1}, the oven temperature was maintained between 60 to 80 °C, the product distilled at 3-4 mbar of pressure and was collected by cooling the receiving flask with ice cold water bath.
- For the pentasubstituted BCB, the oven temperature was maintained between 50 to 60 °C, the product distilled at 0.1 mbar of pressure and was collected by cooling the receiving flask with ice cold water bath.

(1R*,2R*)-2-Butyl-2-methylbicyclo[1.1.0]butane (6a)

Scale, physical description, NMR yield: 0.5 mmol, colorless oil, 56% yield.

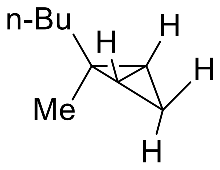

^{1}H-NMR (400 MHz, CDCl$_3$): δ 1.68 (td, J = 3.4, 1.8 Hz, 1H), 1.33 (t, J = 3.8 Hz, 5H), 1.27 (t, J = 4.7 Hz, 2H), 1.12 (t, J = 2.6 Hz, 2H), 0.89 (t, J = 7.0 Hz, 3H), 0.80 (s, 3H).

^{13}C-NMR (101 MHz, CDCl$_3$): 51.09, 38.30, 29.74, 29.25, 23.13, 14.23, 11.81, 7.21.

HRMS (APCI): m/z calculated for C_9H_{17} $[M+H]^+$: 125.1325, found 125.1315.

(1R*,2S*)-2-Butyl-2-phenethylbicyclo[1.1.0]butane (6b)

Scale, physical description, NMR yield: 0.321 mmol, colorless oil, 92% yield.

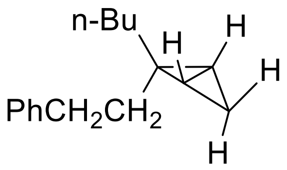

^{1}H-NMR (400 MHz, CDCl$_3$):δ 7.30 – 7.26 (m, 1H), 7.25 (s, 1H), 7.23 – 7.12 (m, 3H), 2.59 – 2.51 (m, 2H), 1.73 (td, J = 3.4, 1.9 Hz, 1H), 1.50 – 1.44 (m, 2H), 1.43 – 1.39 (m, 2H), 1.38 – 1.32 (m, 3H), 1.31 (d, J = 4.7 Hz, 1H), 1.27 (d, J = 3.9 Hz, 1H), 1.21 (dd, J = 3.4, 1.8 Hz, 2H), 0.91 (t, J = 7.0 Hz, 3H).

^{13}C-NMR (101 MHz, CDCl$_3$): δ 143.32, 128.41, 128.35, 125.63, 55.65, 35.03, 33.94, 29.79, 29.14, 26.68, 23.20, 14.24, 7.69.

HRMS (APCI): m/z calculated for $C_{16}H_{23}$ [M+H]$^+$: 215.1800, found 215.1823.

(1R*,2R*)-2-Methyl-2-phenethylbicyclo[1.1.0]butane (6c)

Scale, physical description, NMR yield: 0.3 mmol, colorless oil, 77% yield.

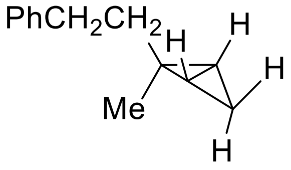

^{1}H-NMR (400 MHz, CDCl$_3$): δ 7.34 – 7.26 (m, 2H), 7.23 – 7.14 (m, 3H), 2.76 – 2.60 (m, 2H), 1.73 – 1.62 (m, 3H), 1.30 (q, J = 1.9 Hz, 1H), 1.15 (dd, J = 3.3, 1.8 Hz, 2H), 0.91 (s, 3H).

^{13}C-NMR (101 MHz, CDCl$_3$): δ 142.76, 128.49, 128.44, 125.85, 50.56, 40.68, 33.49, 29.63, 11.86, 7.26.

HRMS (APCI): m/z calculated for $C_{13}H_{17}$ [M+H]$^+$: 173.1330, found 173.1346.

(1R*,2S*)-2-Phenethyl-2-vinylbicyclo[1.1.0]butane (6d)

Scale, physical description, NMR yield: 0.250 mmol, colorless oil, 90% yield.

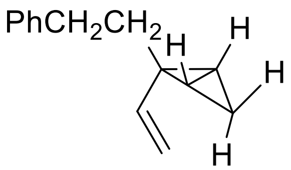

^{1}H-NMR (400 MHz, CDCl$_3$): δ 7.34 – 7.26 (m, 2H), 7.23 – 7.14 (m, 3H), 5.57 (dd, J = 17.0, 10.0 Hz, 1H), 5.14 (dd, J = 10.0, 2.5 Hz, 1H), 4.99 (dd, J = 16.9, 2.5 Hz, 1H), 2.71 – 2.60 (m, 2H), 1.83 – 1.75 (m, 2H), 1.65 (td, J = 3.3, 1.3 Hz, 1H), 1.36 (t, J = 2.5 Hz, 2H), 1.16 (q, J = 1.7 Hz, 1H).
^{13}C-NMR (101 MHz, CDCl$_3$): δ 142.63, 133.41, 128.55, 128.41, 125.86, 116.70, 58.54, 38.23, 33.52, 29.85, 7.48.

HRMS (APCI): m/z calculated for $C_{14}H_{17}$ [M+H]$^+$: 185.1325, found 185.1344.

(1R*,2R*)-2-Butyl-2-(3-choropropyl)bicyclo[1.1.0]butane (6e)

Scale, physical description, NMR yield: 0.3 mmol, colorless oil, 92% yield.

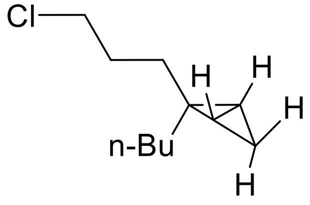

^{1}H-NMR (400 MHz, CDCl$_3$): δ 3.53 (t, J = 6.6 Hz, 2H), 1.86 – 1.76 (m, 2H), 1.71 (td, J = 3.4, 1.9 Hz, 1H), 1.52 – 1.45 (m, 2H), 1.35 – 1.23 (m, 4H), 1.23 – 1.17 (m, 3H), 1.12 (dd, J = 8.7, 5.3 Hz, 2H), 0.88 (t, J = 7.1 Hz, 3H).

^{13}C-NMR (101 MHz, CDCl$_3$): δ 54.34, 45.39, 32.44, 29.96, 29.82, 29.48, 23.69, 23.02, 14.33, 7.43.

HRMS (APCI): m/z calculated for $C_{11}H_{18}Cl$ [M-H]$^+$: 185.1092, found 185.1099

(2S*,3S*)-1,2-Diethyl-2-methylbicyclo[1.1.0]butane (6f)

Scale, physical description, NMR yield: 0.5 mmol, colorless oil, 90% yield. n-octane present along with product.

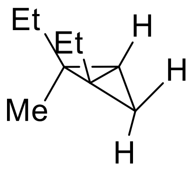

^{1}H-NMR (400 MHz, CDCl$_3$): δ 2.06 (dq, J = 14.6, 7.3 Hz, 1H), 1.62 (dq, J = 14.9, 7.5 Hz, 1H), 1.45 (dd, J = 3.2, 1.2 Hz, 1H), 1.37 (qd, J = 7.5, 5.2 Hz, 2H), 1.13 (d, J = 1.3 Hz, 1H), 0.94 (m, 7H), 0.79 (s, 3H).

^{13}C-NMR (101 MHz, CDCl$_3$): δ 50.77, 28.70, 28.53, 21.43, 18.86, 13.39, 11.33, 11.31, 11.13. **n-octane** peaks: 32.09, 29.48, 22.85, 14.27.

HRMS (APCI): m/z calculated for C_9H_{17} [M+H]$^+$: 125.1325, found 125.1324.

(2R*,3R*)-1,2-Diethyl-2-phenethylbicyclo[1.1.0]butane (6g)

Scale, physical description, NMR yield: 0.5 mmol, colorless oil, 87% yield.

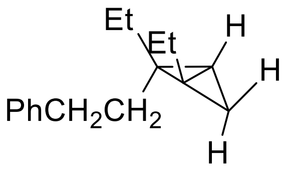

^{1}H-NMR (400 MHz, CDCl$_3$: δ 7.29 (d, J = 6.3 Hz, 1H), 7.27 – 7.24 (m, 1H), 7.22 – 7.14 (m, 3H), 2.62 – 2.43 (m, 2H), 2.06 (dq, J = 15.0, 7.6 Hz, 1H), 1.64 (dqd, J = 15.0, 7.6, 1.6 Hz, 1H), 1.52 (d, J = 7.5 Hz, 2H), 1.49 – 1.41 (m, 3H), 1.17 (s, 1H), 1.08 (s, 1H), 0.99 (t, J = 7.5 Hz, 6H).

^{13}C-NMR (101 MHz, CDCl$_3$): δ 143.45, 128.38, 128.33, 125.58, 55.31, 34.27, 28.72, 26.02, 25.45, 21.97, 18.95, 13.61, 11.92, 11.44.

HRMS (APCI): m/z calculated for $C_{16}H_{23}$ [M+H]$^+$: 215.1794, found 215.1768.

(2R*,3R*)-1,2-Diethyl-2-phenylbicyclo[1.1.0]butane (6h)

Scale, physical description, NMR yield: 0.25 mmol, colorless oil, 94% yield. (BHT is present)

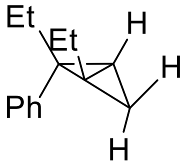

^{1}H-NMR (400 MHz, CDCl$_3$: δ 7.33 – 7.26 (m, 2H), 7.24 – 7.17 (m, 3H), 2.34 – 2.23 (m, 1H), 1.84 (dq, J = 14.8, 7.5 Hz, 1H), 1.71 (qd, J = 7.1, 2.9 Hz, 2H), 1.52 (d, J = 3.0 Hz, 1H), 1.43 (d, J = 3.0 Hz, 1H), 1.17 (t, J = 7.4 Hz, 3H), 0.80 (t, J = 7.4 Hz, 3H), 0.54 (s, 1H).

^{13}C-NMR (101 MHz, CDCl$_3$): δ 140.70, 130.18, 127.64, 126.09, 60.79, 29.77, 28.31, 22.27, 18.94, 14.05, 12.09, 11.36.

HRMS (APCI): m/z calculated for $C_{14}H_{19}$ [M+H]$^+$: 187.1481, found 187.1497.

(2R*,3S*)-2-Methyl-1,2-diphenylbicyclo[1.1.0]butane (6i)

Scale, physical description, NMR yield: 0.5 mmol, colorless oil, 93% yield.

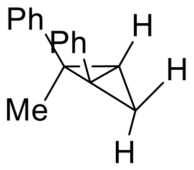

^{1}H-NMR (400 MHz, CDCl$_3$: δ 7.21 (qd, J = 8.5, 7.6, 2.1 Hz, 5H), 7.16 – 7.03 (m, 3H), 7.00 – 6.86 (m, 2H), 2.41 (d, J = 3.3 Hz, 1H), 2.15 (dd, J = 3.4, 1.3 Hz, 1H), 1.88 (d, J = 4.4 Hz, 1H), 1.42 (s, 3H).

^{13}C-NMR (101 MHz, CDCl$_3$): δ 142.15, 137.34, 129.03, 128.07, 127.85, 127.50, 126.38, 125.63, 53.18, 31.35, 25.24, 18.84, 13.55.

HRMS (APCI): m/z calculated for $C_{17}H_{17}$ $[M+H]^+$: 221.1325, found 221.1341.

(2S*,3S*)-2-Ethyl-2-methyl-1-phenylbicyclo[1.1.0]butane (6j)

Scale, physical description, NMR yield: 0.20 mmol, colorless oil, 91% yield.

Scale, physical description, NMR yield: 3.69 mmol, colorless oil, 82% yield.

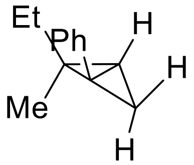

^{1}H-NMR (400 MHz, CDCl$_3$: δ 7.28 (d, J = 1.6 Hz, 1H), 7.25 (d, J = 2.9 Hz, 3H), 7.16 (tt, J = 5.5, 2.6 Hz, 1H), 2.07 (dd, J = 3.4, 1.5 Hz, 1H), 1.75 – 1.68 (m, 2H), 1.60 (dq, J = 13.9, 7.5 Hz, 1H), 1.37 – 1.26 (m, 1H), 1.01 (s, 3H), 0.90 (t, J = 7.5 Hz, 3H).

^{13}C-NMR (101 MHz, CDCl$_3$): δ 138.90, 128.61, 128.23, 125.69, 52.75, 34.19, 29.54, 23.36, 19.10, 11.54, 11.42.

HRMS (APCI): m/z calculated for $C_{13}H_{17}$ $[M+H]^+$: 173.1325, found 173.1315.

(2S*,3S*)-(2-Butyl-2-ethylbicyclo[1.1.0]butan-1-yl)trimethylsilane (6k)

Scale, physical description, NMR yield: 0.30 mmol, colorless oil, 85% yield.

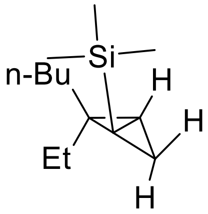

^{1}H-NMR (400 MHz, CDCl$_3$: δ 1.59 (d, J = 2.7 Hz, 1H), 1.54 – 1.46 (m, 1H), 1.40 – 1.22 (m, 5H), 1.16 (td, J = 7.3, 4.8 Hz, 2H), 1.09 (s, 1H), 1.08 – 0.99 (m, 1H), 0.89 (t, J = 6.8 Hz, 3H), 0.78 (t, J = 7.4 Hz, 3H), 0.06 (s, 9H).z

^{13}C-NMR (101 MHz, CDCl$_3$): δ 56.87, 34.81, 29.92, 29.68, 23.29, 18.09, 15.64, 14.26, 12.01, 3.96, 0.21.

^{29}Si-NMR (80 MHz, CDCl$_3$) δ -0.67.

HRMS (APCI): m/z calculated for $C_{13}H_{27}Si$ $[M+H]^+$: 211.1887, found 211.1870.

(2R*,3S*)-((E)-(2-butyl-2-(5-methylhex-3-en-1-yl)bicyclo[1.1.0]butan-1-yl)trimethylsilane (6l)

(**Scale, physical description, NMR yield**: 0.30 mmol, colorless oil, 85% yield.

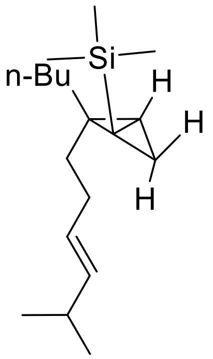

^{1}H-NMR (400 MHz, $CDCl_3$: δ 5.47 – 5.29 (m, 2H), 2.03 – 1.89 (m, 1H), 1.84 (q, J = 8.1, 7.1 Hz, 3H), 1.60 – 1.56 (m, 1H), 1.54 – 1.45 (m, 1H), 1.37 – 1.26 (m, 4H), 1.24 – 1.16 (m, 2H), 1.10 (s, 1H), 1.08 – 1.01 (m, 1H), 0.88 (dd, J = 13.7, 6.7 Hz, 9H), 0.06 (s, 9H).

^{13}C-NMR (101 MHz, $CDCl_3$): δ 131.99, 128.57, 55.42, 42.20, 35.45, 31.13, 29.99, 28.64, 23.45, 23.27, 22.45, 17.87, 14.26, 4.11, 0.20.

^{29}Si-NMR (80 MHz, $CDCl_3$) δ -0.61.

HRMS (APCI): m/z calculated for $C_{18}H_{35}Si$ $[M+H]^+$: 279.2503, found 279.2496.

(1S*,2R*)-2-Butyl-2-methyl-1-phenylbicyclo[1.1.0]butane (6m)

Scale, physical description, NMR yield: 0.50 mmol, colorless oil, 95% yield.

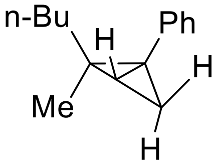

Racemic sample.

(1S,2R)-2-Butyl-2-methyl-1-phenylbicyclo[1.1.0]butane (6m*)

Scale, physical description, NMR yield: 0.10 mmol, colorless oil, 93% yield. 2.5 equivalent of BuLi was used instead of 1.3 equivalent like the standard condition.

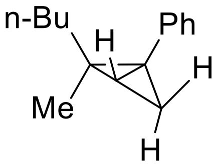

^{1}H-NMR (400 MHz, C_6D_6): δ 7.21 – 7.17 (m, 2H), 7.15 – 7.11 (m, 2H), 7.08 – 7.01 (m, 1H), 1.95 (dd, J = 3.4, 1.6 Hz, 1H), 1.74 (d, J = 1.7 Hz, 1H), 1.60 (dd, J = 3.7, 1.7 Hz, 1H), 1.59 – 1.50 (m, 1H), 1.38 – 1.29 (m, 1H), 1.29 – 1.18 (m, 2H), 1.17 – 1.05 (m, 2H), 1.02 (s, 3H), 0.77 (t, J = 7.3 Hz, 3H).

^{13}C-NMR (101 MHz, C_6D_6): δ 138.88, 129.05, 128.45, 125.99, 51.78, 36.75, 34.47, 29.45, 23.35, 23.33, 19.48, 14.24, 12.04.

HRMS (APCI): m/z calculated for $C_{15}H_{21}$ $[M+H]^+$: 201.1638, found 201.1633.

The enantiomeric ratio was determined by HPLC analysis using CHIRACEL OD column, 1cmI.D x 25 cmL, (n-Hexane, 1.5 mL/min, 20 °C, 254.4 nm, τ major = 16.78 min, τ minor = 22.11 min).

(1S*,2S*)-2-Allyl-2-butyl-1-phenylbicyclo[1.1.0]butane (6n)

Scale, physical description, NMR yield: 0.30 mmol, colorless oil, 86% yield.

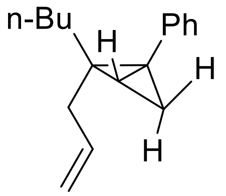

^{1}H-NMR (400 MHz, CDCl$_3$: δ 7.29 – 7.26 (m, 1H), 7.26 – 7.21 (m, 3H), 7.20 – 7.13 (m, 1H), 5.87 (ddt, J = 17.2, 10.1, 7.1 Hz, 1H), 5.19 – 5.03 (m, 2H), 2.15 (dd, J = 3.5, 1.9 Hz, 1H), 2.10 (d, J = 7.2 Hz, 2H), 1.82 (dd, J = 3.5, 1.6 Hz, 1H), 1.77 (d, J = 1.8 Hz, 1H), 1.70 (ddd, J = 14.1, 10.4, 4.5 Hz, 1H), 1.39 (dddd, J = 11.1, 9.0, 6.3, 4.0 Hz, 1H), 1.30 – 1.13 (m, 3H), 1.06 (ddd, J = 13.6, 11.2, 5.0 Hz, 1H), 0.82 (t, J = 7.1 Hz, 3H).

^{13}C-NMR (101 MHz, CDCl$_3$): δ 138.42, 137.98, 128.62, 128.26, 125.79, 116.03, 56.36, 34.64, 33.10, 29.07, 28.87, 23.11, 23.02, 19.80, 14.19.

HRMS (APCI): m/z calculated for $C_{17}H_{23}$ [M+H]$^+$: 227.1794, found 227.1811.

(2R*,3S*)-2-Butyl-1,2-dimethyl-3-phenylbicyclo[1.1.0]butane (6o)

Scale, physical description, NMR yield: 0.161 mmol, colorless oil, 95% yield.

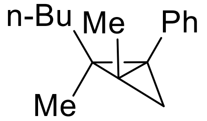

^{1}H-NMR (400 MHz, CDCl$_3$): δ 7.27 (d, J = 7.0 Hz, 1H), δ 7.25 (d, J = 7.0 Hz, 1H), 7.20 – 7.08 (m, 3H), 1.85 (d, J = 1.3 Hz, 1H), 1.69 (s, 1H), 1.68 – 1.60 (m, 1H), 1.55 – 1.47 (m, 1H), 1.46 (s, 3H), 1.35 – 1.20 (m, 4H), 0.99 (s, 3H), 0.87 (t, J = 7.1 Hz, 3H).

^{13}C-NMR (101 MHz, CDCl$_3$): δ 138.60, 128.25, 127.68, 124.90, 49.82, 35.59, 34.34, 28.66, 27.39, 23.89, 23.50, 14.27, 12.20, 9.75.

HRMS (APCI): m/z calculated for $C_{16}H_{23}$ [M+H]$^+$: 215.1794, found 215.1776.

(1S*,2R*)-2-Butyl-1-(4-fluorophenyl)-2,3-dimethylbicyclo[1.1.0]butane (6p)

Scale, physical description, NMR yield: 0.20 mmol, colorless oil, 89% yield.

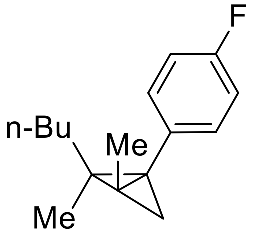

^{1}H-NMR (400 MHz, CDCl$_3$: Zδ 7.14 – 7.06 (m, 2H), 7.00 – 6.91 (m, 2H), 1.76 (d, J = 1.4 Hz, 1H), 1.68 (d, J = 1.4 Hz, 1H), 1.63 – 1.58 (m, 1H), 1.54 – 1.46 (m, 1H), 1.45 (s, 3H), 1.25 (pq, J = 7.0, 4.3, 3.2 Hz, 4H), 0.97 (s, 3H), 0.87 (t, J = 7.0 Hz, 3H).

^{13}C-NMR (101 MHz, CDCl$_3$): δ 160.91 (d, J = 243.0 Hz), 134.05 (d, J = 3.0 Hz), 128.99 (d, J = 7.7 Hz), 115.18 (d, J = 21.2 Hz), 49.68, 35.61, 34.26, 28.56, 26.55, 23.49, 23.21, 14.24, 12.09, 9.60.

^{19}F-NMR (377 MHz, CDCl$_3$): δ -118.28.

HRMS (APCI): m/z calculated for $C_{16}H_{22}F$ [M+H]$^+$: 233.1700, found 233.1721.

(1S*,2R*)-2-Butyl-1-(3-chlorophenyl)-2,3-dimethylbicyclo[1.1.0]butane (6q)

Scale, physical description, NMR yield: 0.25 mmol, colorless oil, 92% yield.

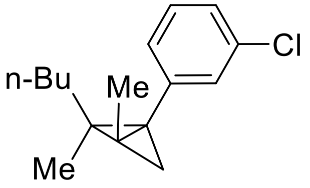

^{1}H-NMR (400 MHz, $CDCl_3$): δ 7.17 (t, J = 7.8 Hz, 1H), 7.13 – 7.07 (m, 2H), 7.03 (dt, J = 7.7, 1.4 Hz, 1H), 1.85 (d, J = 1.6 Hz, 1H), 1.72 (d, J = 1.6 Hz, 1H), 1.70 – 1.60 (m, 1H), 1.56 – 1.46 (m, 1H), 1.45 (s, 3H), 1.35 – 1.27 (m, 3H), 1.23 (d, J = 2.7 Hz, 1H), 0.99 (s, 3H), 0.89 (t, J = 7.0 Hz, 3H).

^{13}C-NMR (101 MHz, $CDCl_3$): δ 141.08, 134.11, 129.43, 127.49, 125.77, 124.99, 50.22, 35.86, 34.24, 30.46, 28.64, 23.78, 23.47, 14.25, 12.22, 9.68.

HRMS (APCI): m/z calculated for $C_{16}H_{22}Cl$ $[M+H]^+$: 249.1405, found 249.1387.

4-((1S*,2R*)-2-Butyl-2,3-dimethylbicyclo[1.1.0]butan-1-yl)benzonitrile (6r)

Scale, physical description, NMR yield: 0.15 mmol, colorless oil, 51% yield.

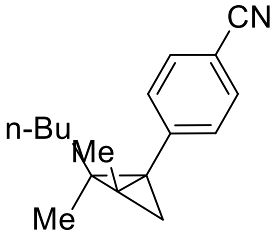

^{1}H-NMR (400 MHz, $CDCl_3$): δ 7.50 (d, J = 8.3 Hz, 2H), 7.19 (d, J = 8.3 Hz, 2H), 1.97 (d, J = 2.0 Hz, 1H), 1.82 (d, J = 2.0 Hz, 1H), 1.51 (td, J = 6.4, 6.0, 4.3 Hz, 2H), 1.42 (s, 3H), 1.31 – 1.26 (m, 4H), 1.01 (s, 3H), 0.89 (d, J = 7.0 Hz, 3H).

^{13}C-NMR (101 MHz, $CDCl_3$): δ 145.72, 131.94, 127.74, 107.74, 51.23, 36.44, 34.36, 32.28, 28.60, 24.76, 23.43, 14.19, 12.52, 9.81.

HRMS (APCI): m/z calculated for $C_{17}H_{21}N$ $[M+H]^+$: 240.1747, found 240.1753.

2.8 General procedure for the synthesis of Pentasubstituted BCB.

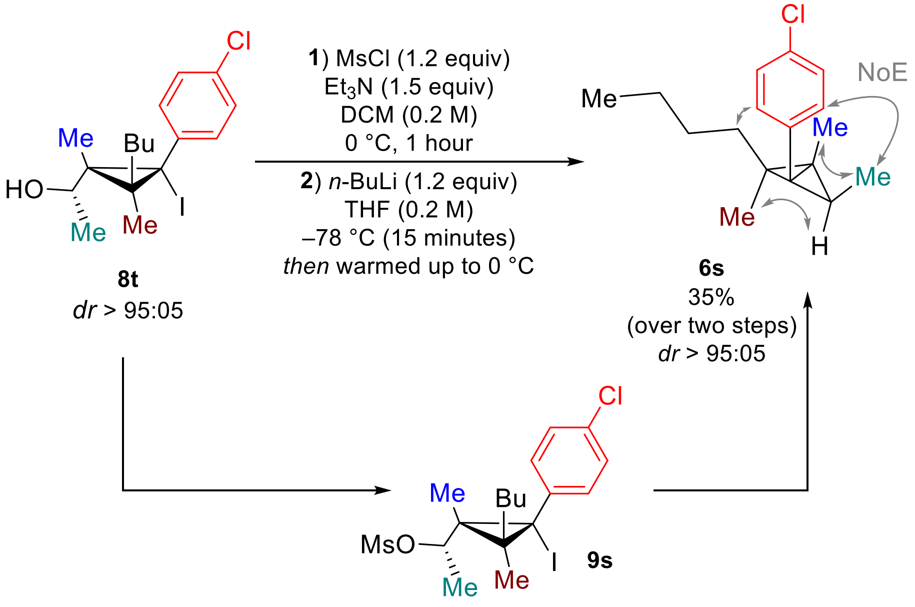

Step A: In a flame dried Schlenk tube, a stirred solution of iodocyclopropanol (**8t,** 1.0 equiv.) in DCM (0.2 M) was cooled to 0 °C (ice bath). After 5 minutes, Et_3N (1.3 equiv.) and MsCl (1.3 equiv.) were added. After one hour, water was added to the flask. The mixture was directly poured into a separation funnel and was extracted with DCM (50 mL/mmol, 3 times). The combined organic layer was dried with Na_2SO_4, filtered, and concentrated in vacuo. This reaction mixture was taken for the next step without any purification.

Step B: In a flame dried Schlenk tube, a stirred solution of iodocyclopropane (**9s**, 1.0 equiv.) in THF (0.2 M) was cooled to -78 °C (dry ice bath). After 15 minutes, n-BuLi (1.2 equiv.) was added dropwise. After 15 minutes, the reaction mixture was slowly warmed up 0 °C over three hours. Water was added to hydrolyze the reaction. The mixture was directly poured into a separation funnel and was extracted with Et_2O (50 mL/mmol, 3 times). The combined organic layers were dried with Na_2SO_4, filtered, and concentrated in vacuo. The NMR yields were calculated using tertbutyl methyl ether as the internal standard and crude product was further purified further purified by Kugelrohr distillation to obtain **6s**.

For distillation, the oven temperature was maintained between 50 to 60 °C, the product distilled at 0.1 mbar of pressure and was collected by cooling the receiving flask with ice cold water bath.

((1R*,2R*,4S*)-2-Butyl-1-(4-chlorophenyl)-2,3,4-trimethylbicyclo[1.1.0]butane (6s)

Scale, physical description, NMR yield: 0.20 mmol, colorless oil, 35% yield.

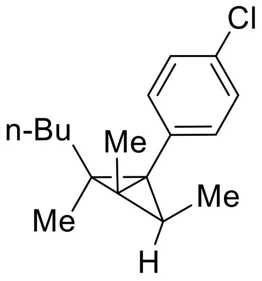

^{1}H-NMR (400 MHz, CDCl$_3$): δ 7.23 (d, J = 8.2 Hz, 2H), 7.14 (d, J = 8.3 Hz, 2H), 1.75 (q, J = 5.7 Hz, 1H), 1.51 (s, 3H), 1.35 – 1.29 (m, 2H), 1.22 – 1.12 (m, 4H), 0.93 (s, 3H), 0.89 (d, J = 5.8 Hz, 3H), 0.80 (t, J = 7.1 Hz, 3H).

^{13}C-NMR (101 MHz, CDCl$_3$): δ 134.77, 132.15, 131.81, 128.13, 46.84, 36.71, 34.02, 28.28, 27.75, 26.04, 23.42, 14.17, 11.17, 10.93, 5.06.

HRMS (APCI): m/z calculated for $C_{17}H_{24}Cl$ $[M+H]^+$: 263.1561, found 263.1560.

3. References.

1) Cohen, Y.; Marek, I. Regio– And Diastereoselective Copper–Catalyzed Carbometalation of Cyclopropenylsilanes. *Org. Lett.* **2019**, *21*, 9162–9165.
2) Cohen, Y.; Augustin, A. U.; Levy, L.; Jones, P. G.; Werz, D. B.; Marek, I. Regio– and Diastereoselective Copper–Catalyzed Carbomagnesiation for the Synthesis of Penta– and Hexa–Substituted Cyclopropanes. *Angew. Chem. Int. Ed*. **2021**, *60*, 11804–11808.
3) Davies, H. M.; Lee, G. H. Dirhodium(II)Tetra(N-(dodecylbenzenesulfonyl)prolinate) catalyzed enantioselective cyclopropenation of alkynes. *Org. Lett*. **2004**, 6, 1233-1236.
4) Fordyce, E. A.; Luebbers, T.; Lam, H. W. Synthesis and application of alkenylstannanes derived from base-sensitive cyclopropenes. *Org. lett*. **2008**, *10*, 3993-3996.
5) Liao, L. A.; Fox, J. M. A Copper–Catalyzed Method for the Facially Selective Addition of Grignard Reagents to Cyclopropenes. *J. Am. Chem. Soc.* **2002**, *124*, 14322–14323.

4. Spectrum of compounds

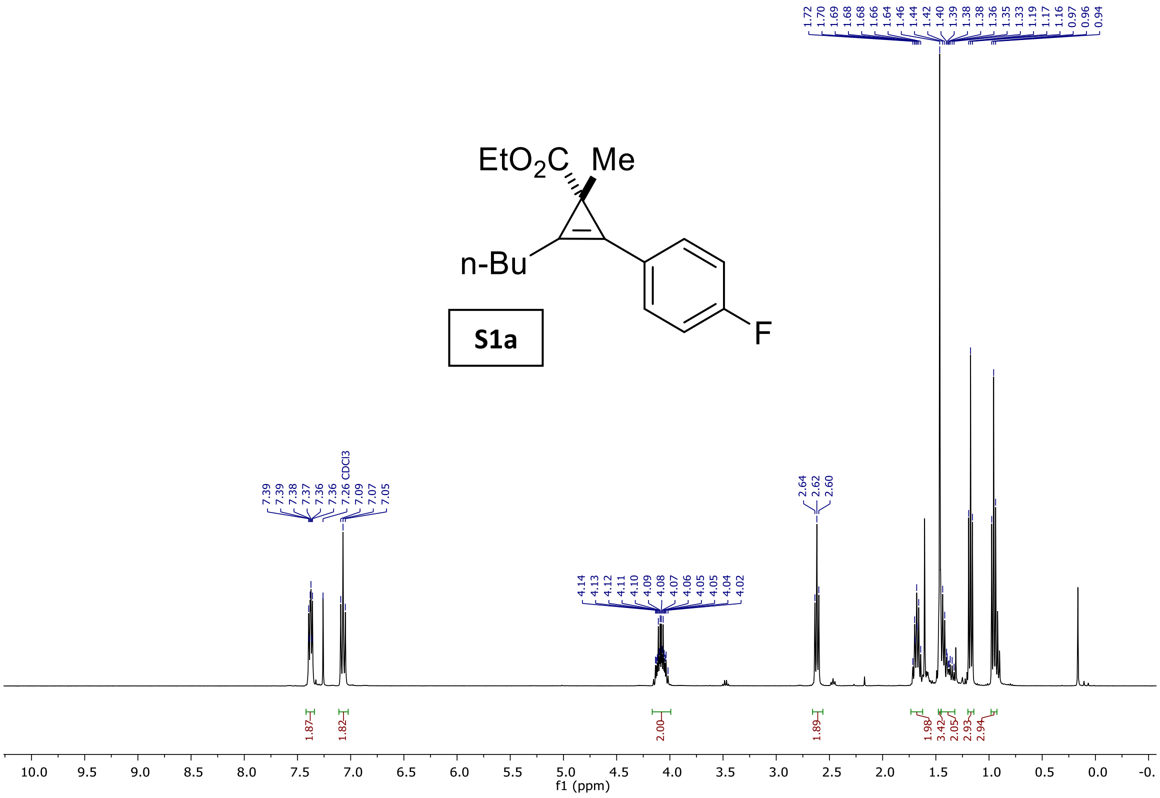

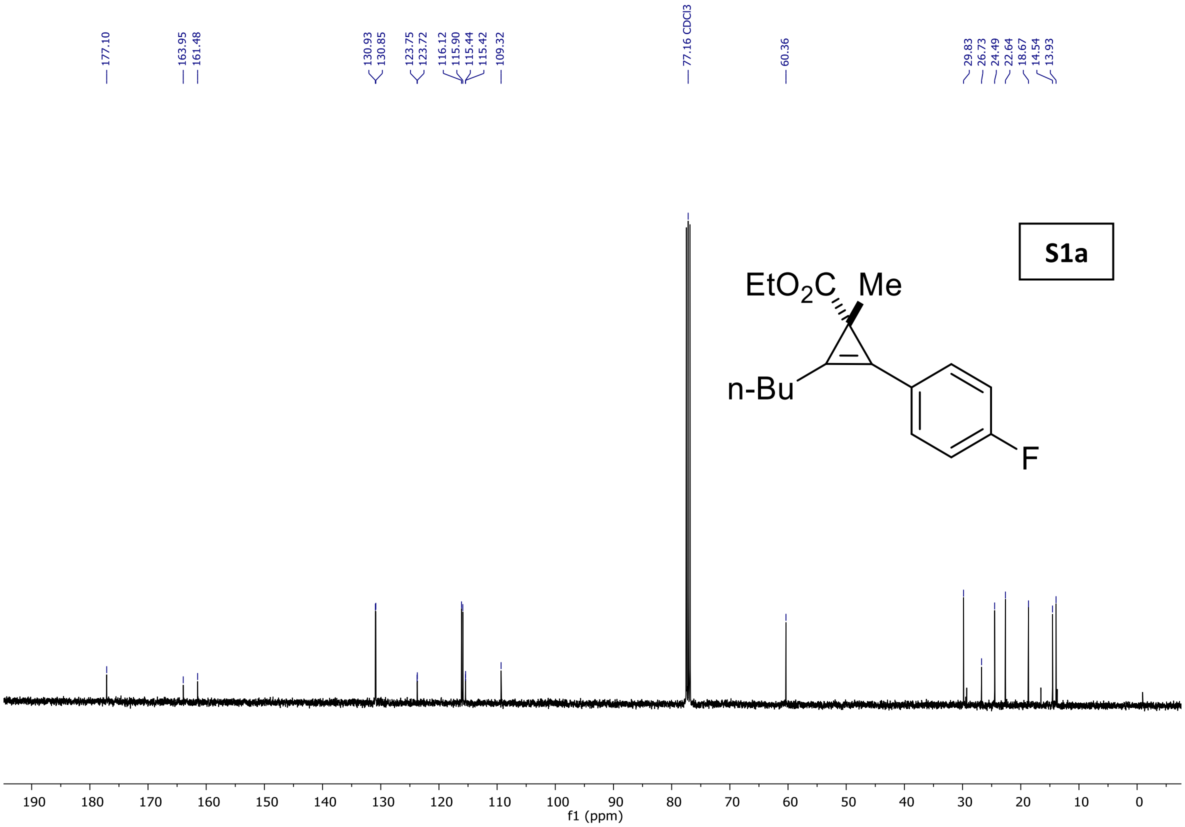
177.10
163.95
161.48
130.93
130.85
123.75
123.72
116.12
115.90
115.44
115.42
109.32
77.16 CDCl3
60.36
29.83
26.73
24.49
22.64
18.67
14.54
13.93
EtO2C
Me
n-Bu
F
S1a
f1 (ppm)

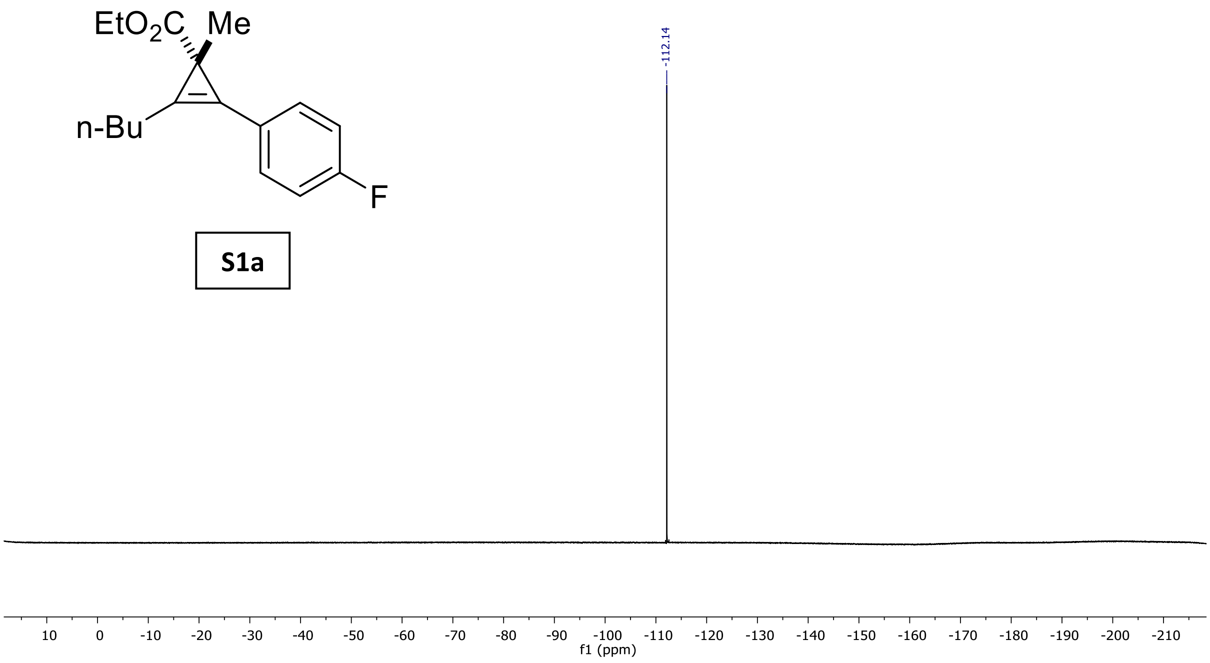
EtO2C
Me
n-Bu
F
S1a
-112.14
f1 (ppm)

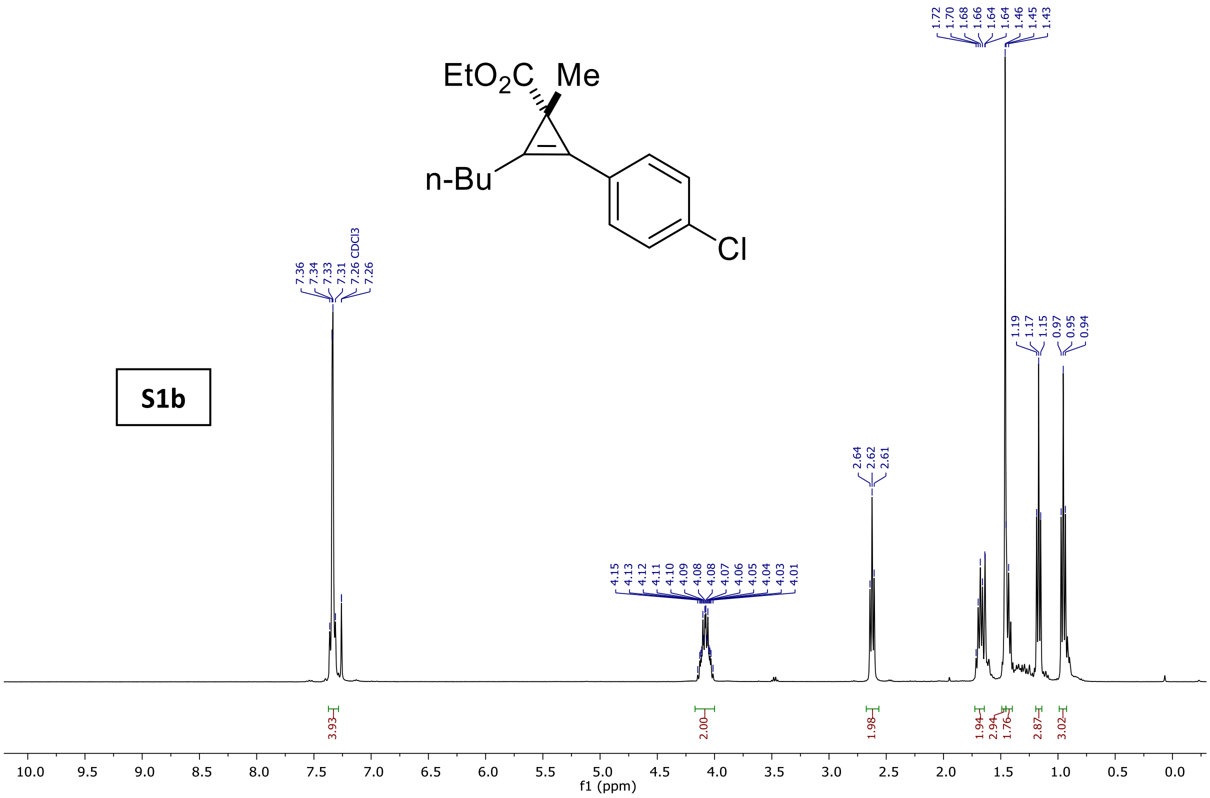

EtO2C Me
n-Bu
Cl
S1b
7.36 7.34 7.33 7.31 7.26 CDCl3 7.26
4.15 4.13 4.12 4.11 4.10 4.09 4.08 4.08 4.07 4.06 4.05 4.04 4.03 4.01
2.64 2.62 2.61
1.72 1.70 1.68 1.66 1.64 1.64 1.46 1.45 1.43
1.19 1.17 1.15 0.97 0.95 0.94
3.93
2.00
1.98
1.94 2.94 1.76
2.87
3.02
f1 (ppm)

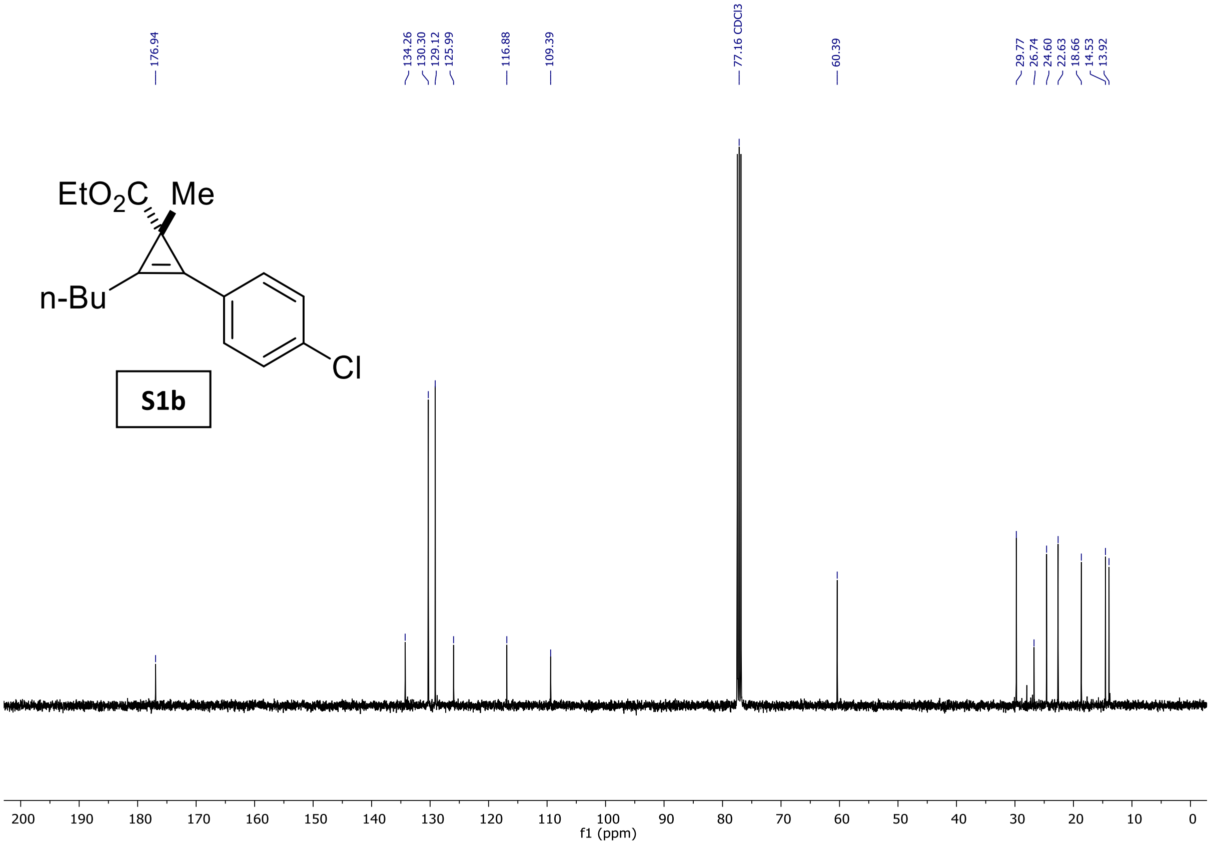

176.94
134.26 130.30 129.12 125.99
116.88
109.39
77.16 CDCl3
60.39
29.77 26.74 24.60 22.63 18.66 14.53 13.92
EtO2C Me
n-Bu
Cl
S1b
f1 (ppm)

S1c

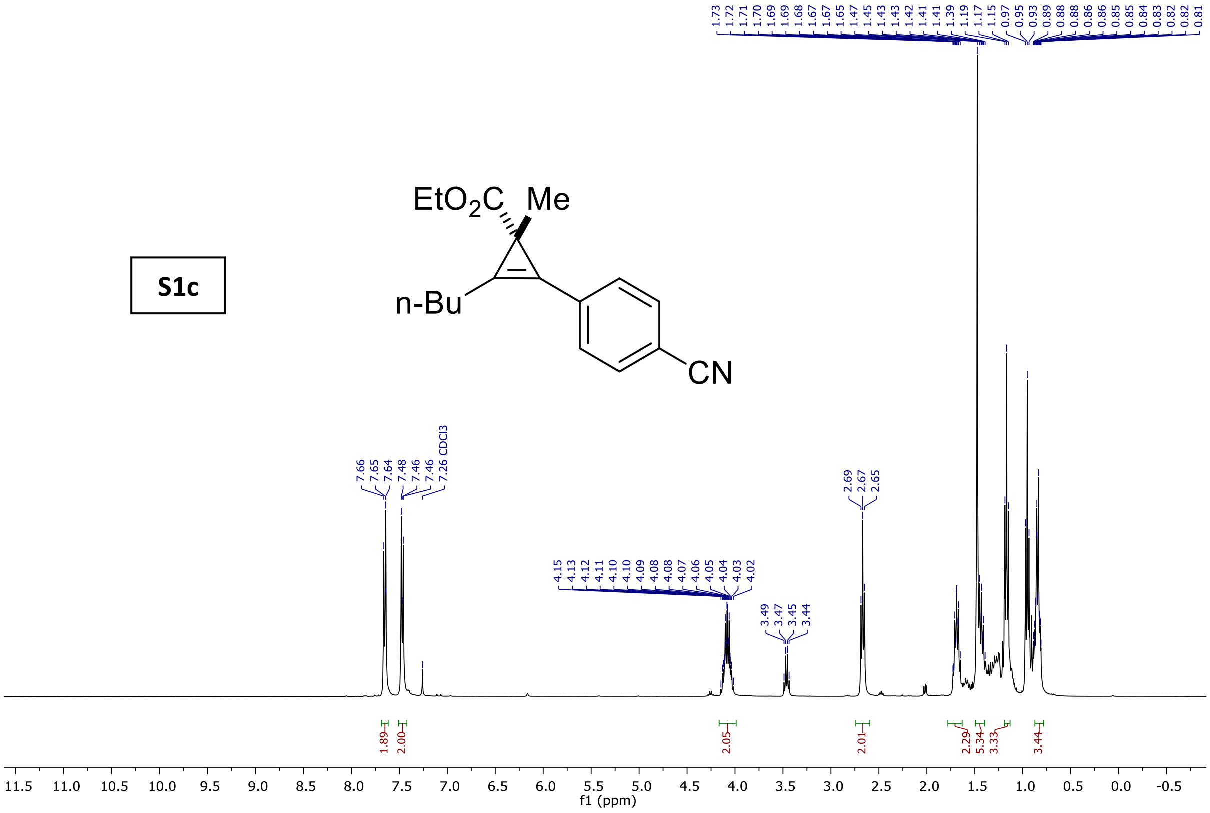
EtO2C Me
n-Bu
CN
7.66 7.65 7.64 7.48 7.46 7.46 7.26 CDCl3
4.15 4.13 4.12 4.11 4.10 4.10 4.09 4.08 4.08 4.07 4.06 4.05 4.04 4.03 4.02
3.49 3.47 3.45 3.44
2.69 2.67 2.65
1.73 1.72 1.71 1.70 1.69 1.68 1.67 1.67 1.65 1.47 1.45 1.43 1.43 1.42 1.41 1.41 1.39 1.19 1.17 1.15 0.97 0.95 0.93 0.89 0.88 0.88 0.86 0.86 0.85 0.85 0.84 0.83 0.82 0.81
1.89 2.00 2.05 2.01 2.29 5.34 3.33 3.44
f1 (ppm)

S1c

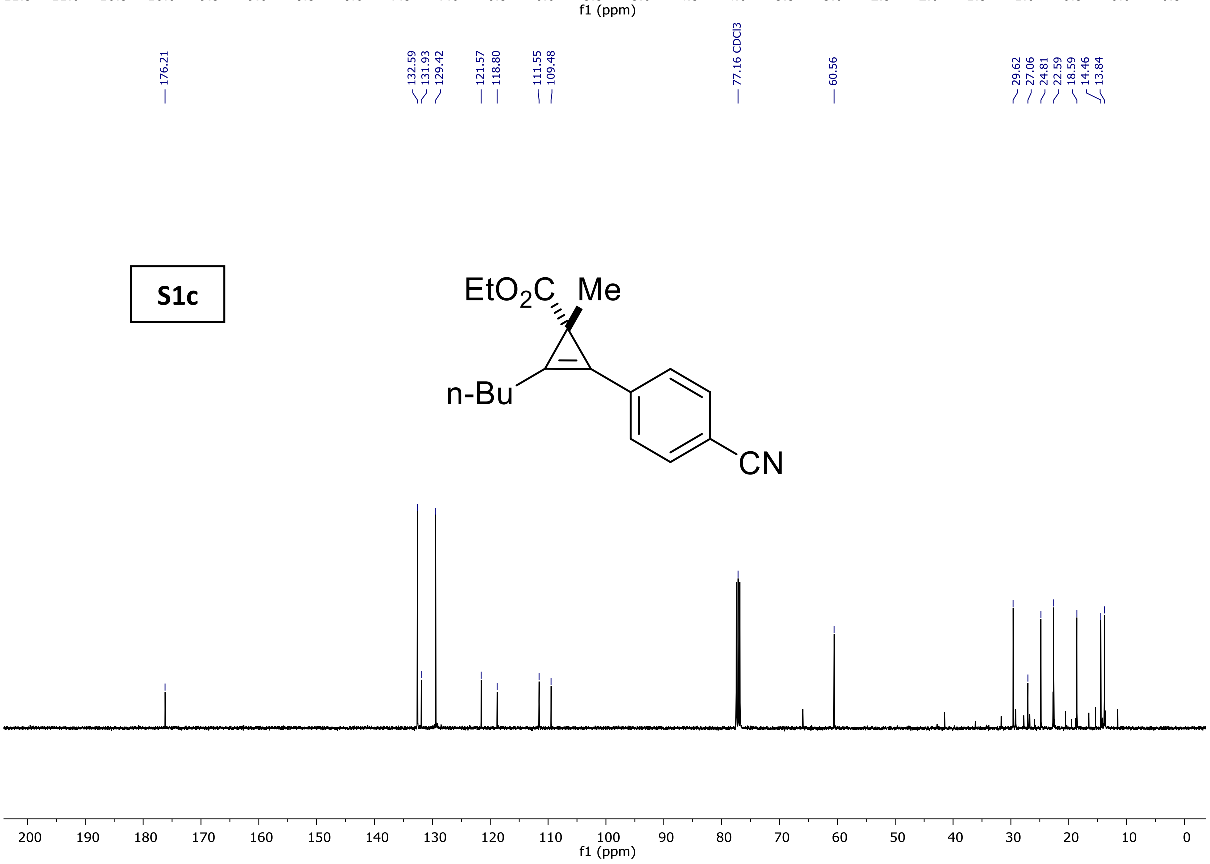
EtO2C Me
n-Bu
CN
176.21
132.59 131.93 129.42
121.57 118.80
111.55 109.48
77.16 CDCl3
60.56
29.62 27.06 24.81 22.59 18.59 14.46 13.84
f1 (ppm)

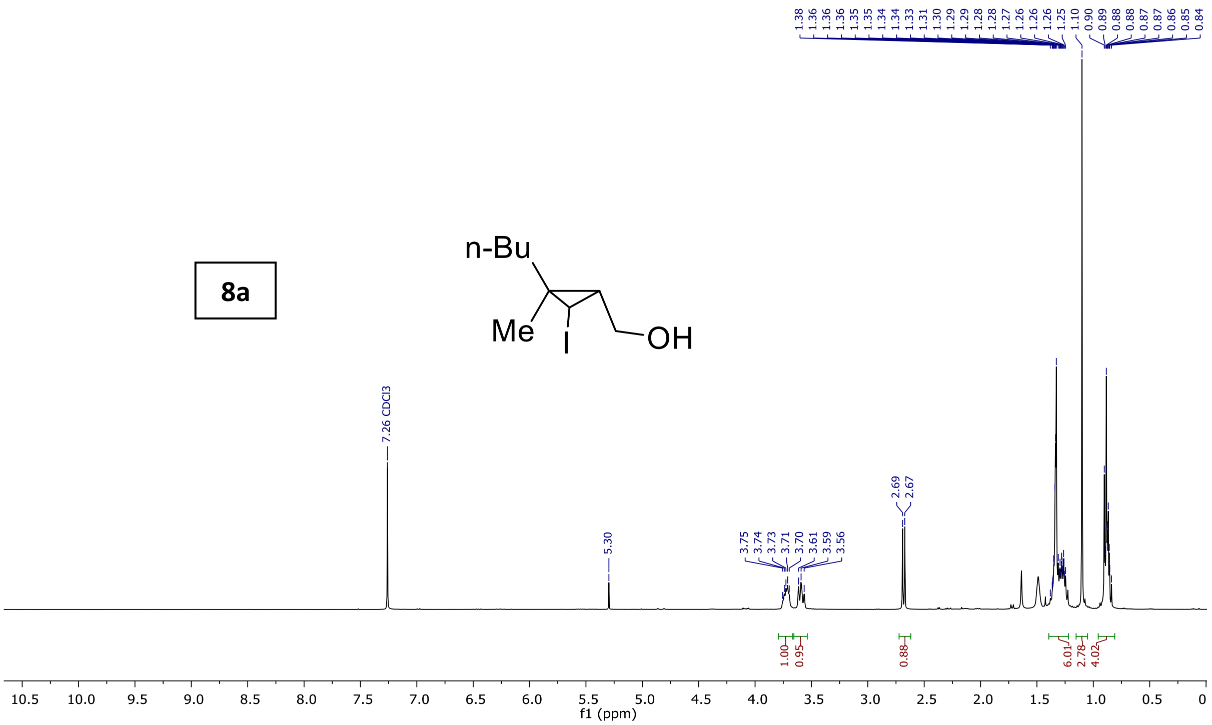
8a
n-Bu
Me
I
OH
7.26 CDCl3
5.30
3.75
3.74
3.73
3.71
3.70
3.61
3.59
3.56
2.69
2.67
1.38
1.36
1.36
1.36
1.35
1.35
1.34
1.34
1.33
1.31
1.30
1.29
1.29
1.28
1.28
1.27
1.26
1.26
1.26
1.25
1.10
0.90
0.89
0.88
0.88
0.87
0.87
0.86
0.85
0.84
1.00
0.95
0.88
6.01
2.78
4.02
f1 (ppm)

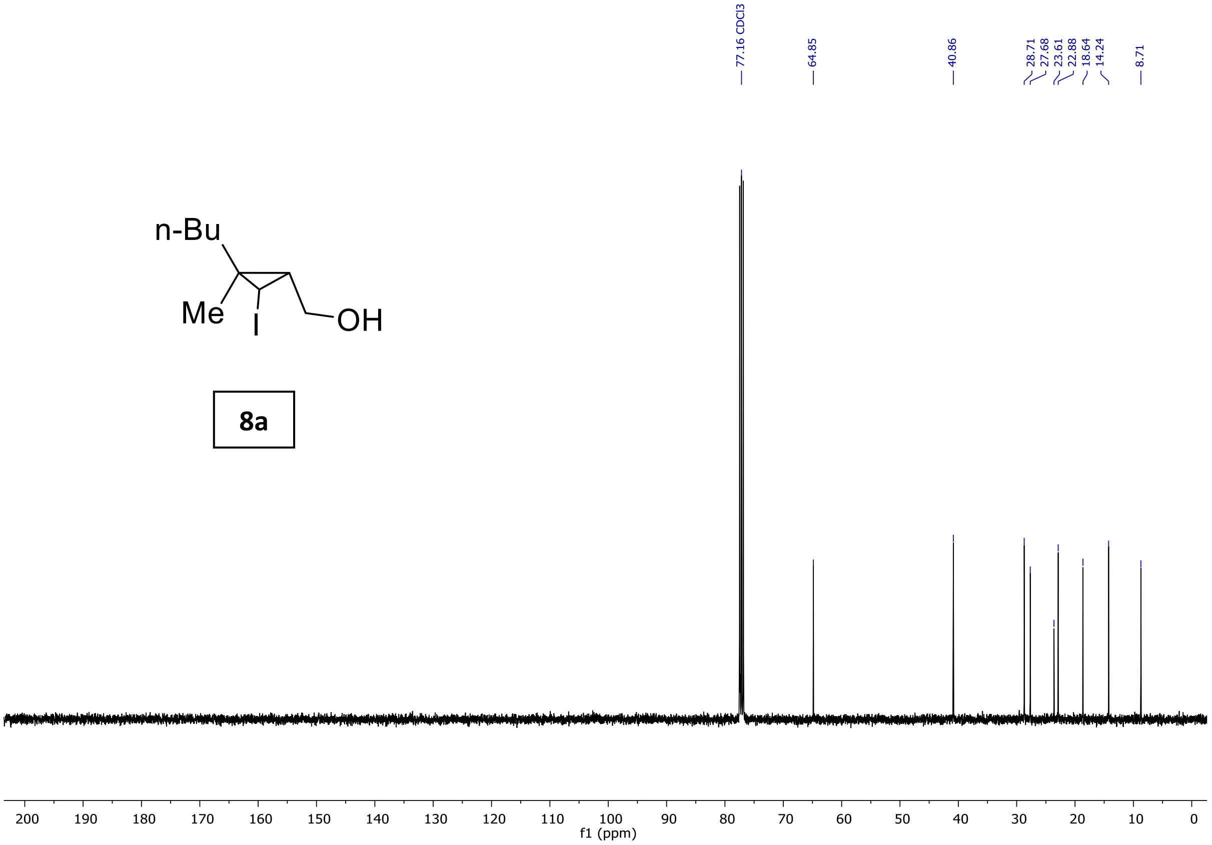
77.16 CDCl3
64.85
40.86
28.71
27.68
23.61
22.88
18.64
14.24
8.71
n-Bu
Me
I
OH
8a
f1 (ppm)

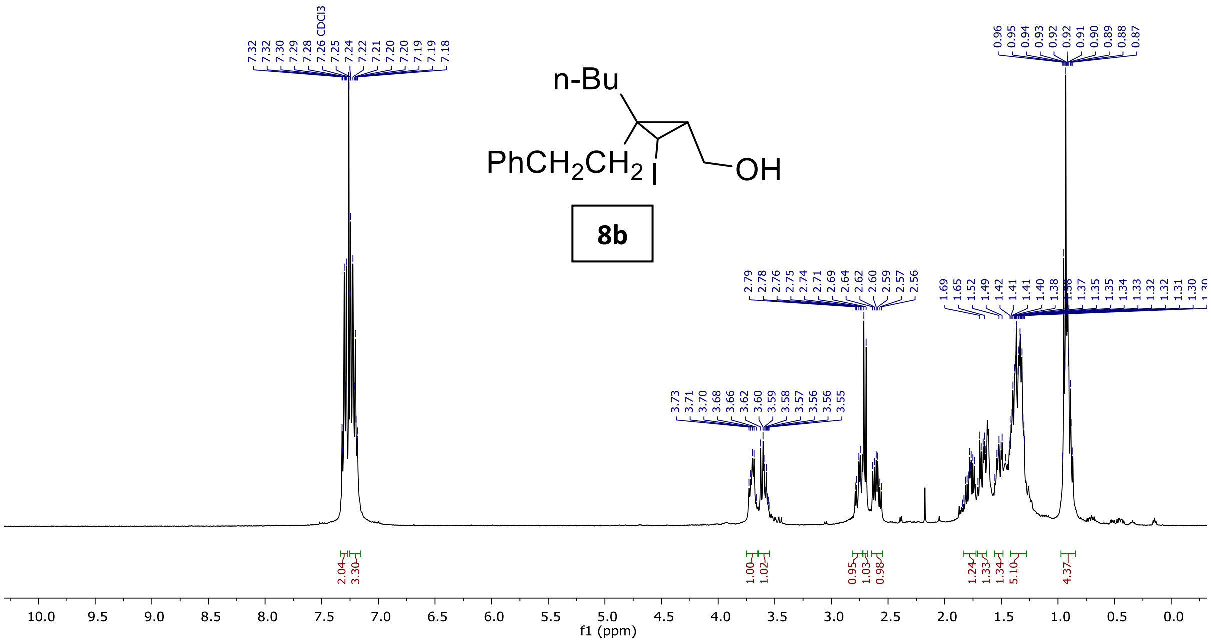
n-Bu
PhCH2CH2
I
OH
8b
f1 (ppm)

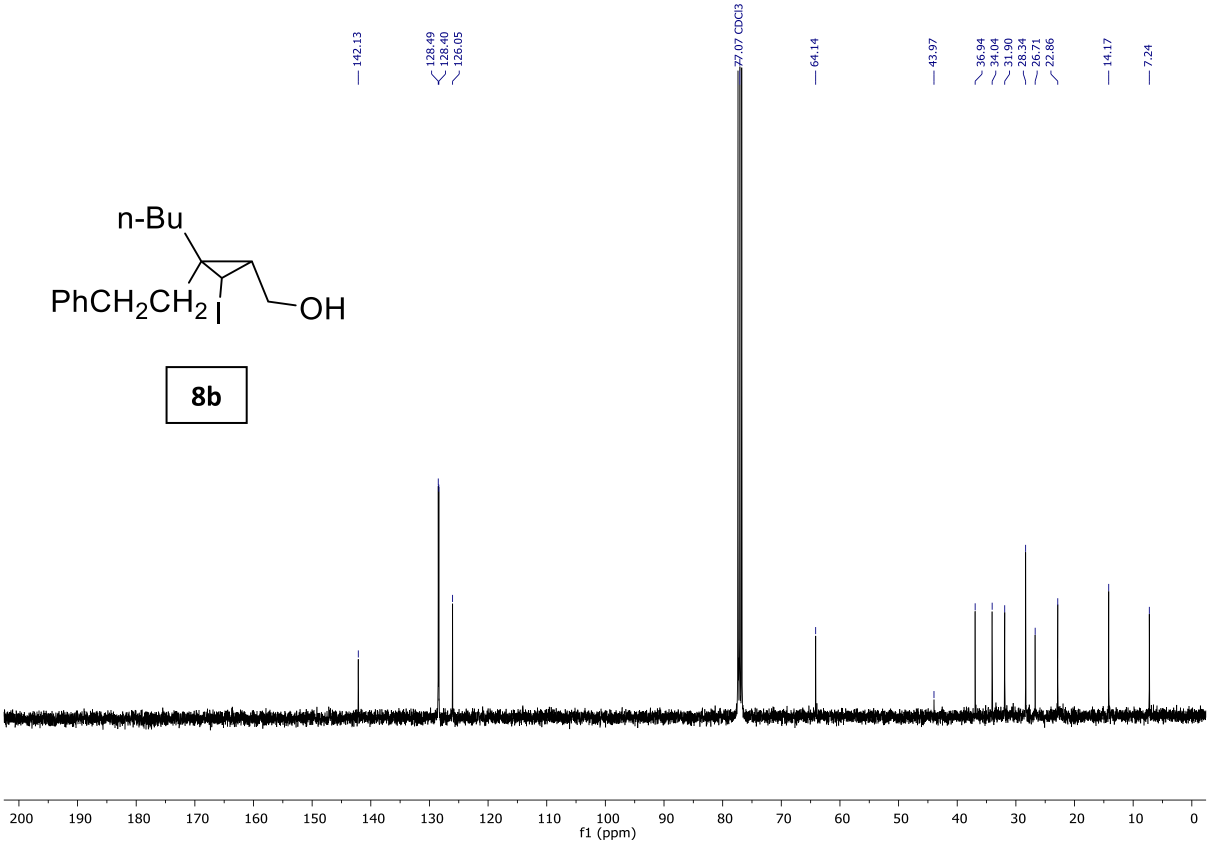
n-Bu
PhCH2CH2
I
OH
8b
f1 (ppm)

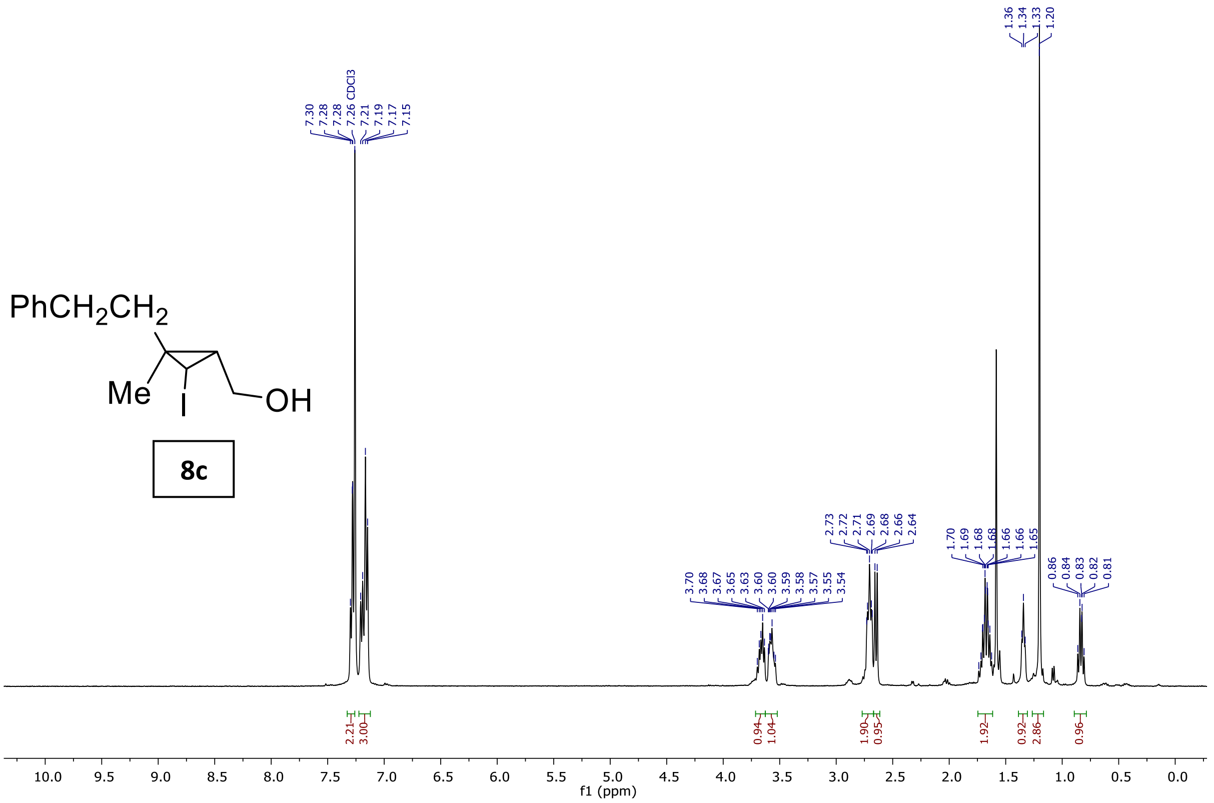
PhCH2CH2
Me
I
OH
8c
7.30
7.28
7.28
7.26 CDCl3
7.21
7.19
7.17
7.15
3.70
3.68
3.67
3.65
3.63
3.60
3.60
3.59
3.58
3.57
3.55
3.54
2.73
2.72
2.71
2.69
2.68
2.66
2.64
1.70
1.69
1.68
1.68
1.66
1.66
1.65
1.36
1.34
1.33
1.20
0.86
0.84
0.83
0.82
0.81
2.21
3.00
0.94
1.04
1.90
0.95
1.92
0.92
2.86
0.96
f1 (ppm)

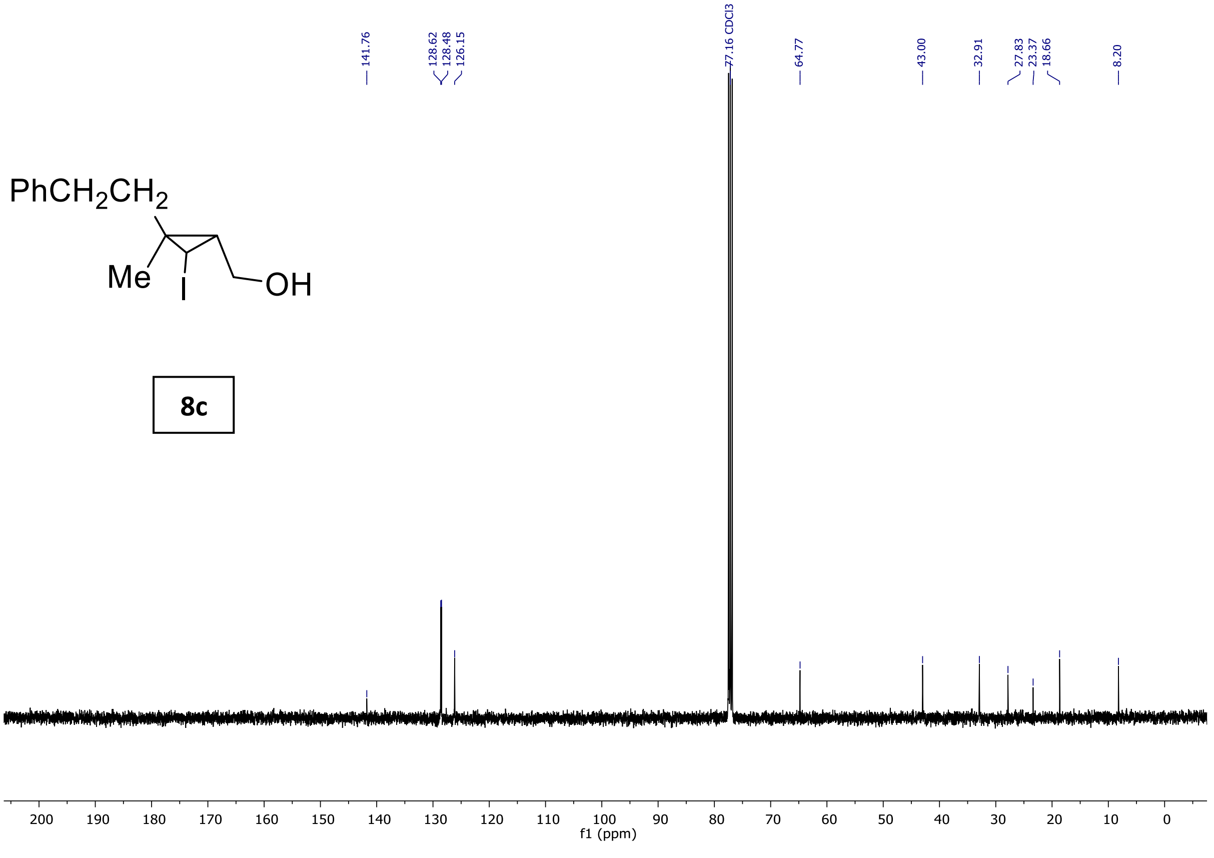
PhCH2CH2
Me
I
OH
8c
141.76
128.62
128.48
126.15
77.16 CDCl3
64.77
43.00
32.91
27.83
23.37
18.66
8.20
f1 (ppm)

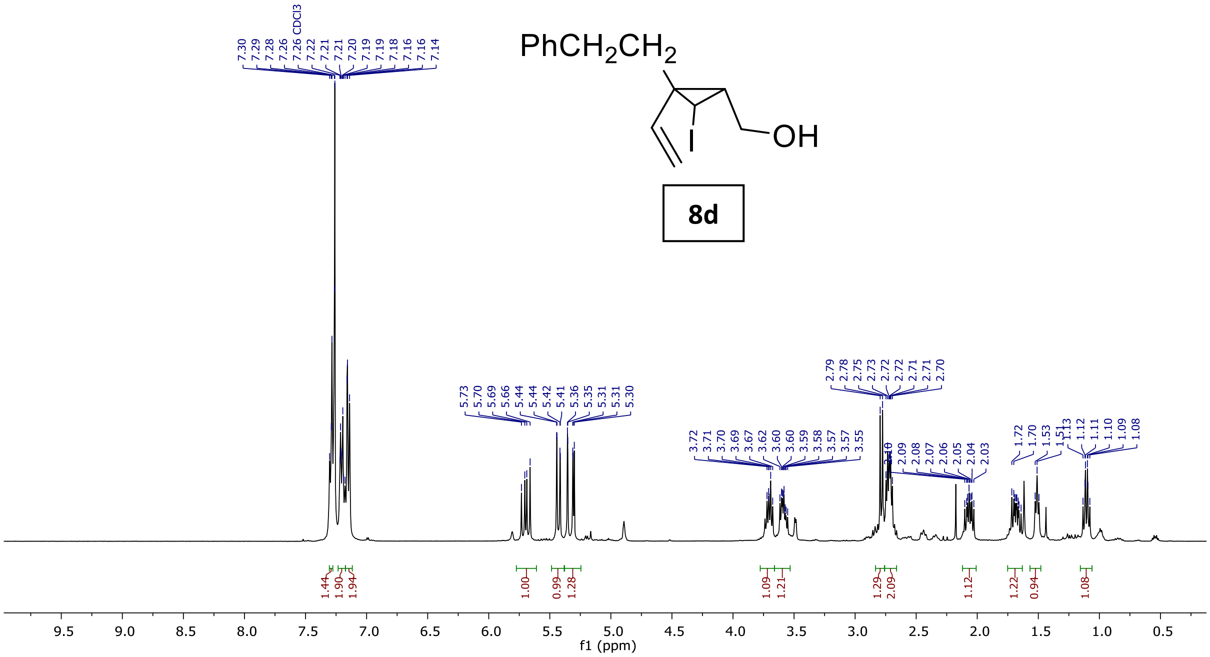
PhCH2CH2
I
OH
8d
7.30 7.29 7.28 7.26 7.26 CDCl3 7.22 7.21 7.21 7.20 7.19 7.19 7.18 7.16 7.16 7.14
5.73 5.70 5.69 5.66 5.44 5.44 5.42 5.41 5.36 5.35 5.31 5.31 5.30
3.72 3.71 3.70 3.69 3.67 3.62 3.60 3.60 3.59 3.58 3.57 3.57 3.55
2.79 2.78 2.75 2.73 2.72 2.72 2.71 2.71 2.70
2.10 2.09 2.08 2.07 2.06 2.05 2.04 2.03
1.72 1.70 1.53 1.51 1.13 1.12 1.11 1.10 1.09 1.08
1.44 1.90 1.94 1.00 0.99 1.28 1.09 1.21 1.29 2.09 1.12 1.22 0.94 1.08
9.5 9.0 8.5 8.0 7.5 7.0 6.5 6.0 5.5 5.0 4.5 4.0 3.5 3.0 2.5 2.0 1.5 1.0 0.5
f1 (ppm)

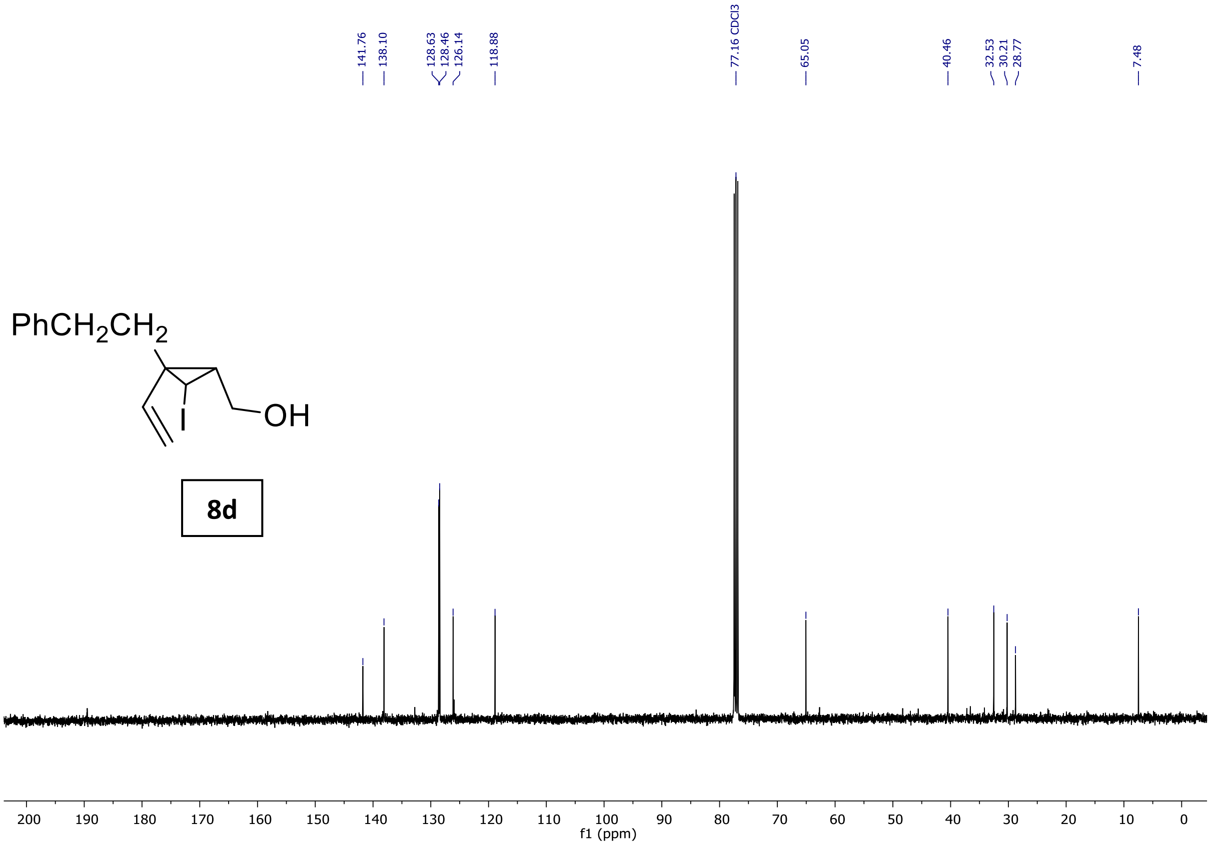
141.76 138.10 128.63 128.46 126.14 118.88 77.16 CDCl3 65.05 40.46 32.53 30.21 28.77 7.48
PhCH2CH2
I
OH
8d
200 190 180 170 160 150 140 130 120 110 100 90 80 70 60 50 40 30 20 10 0
f1 (ppm)

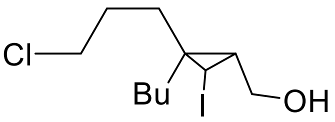
Cl
Bu
I
OH

8e

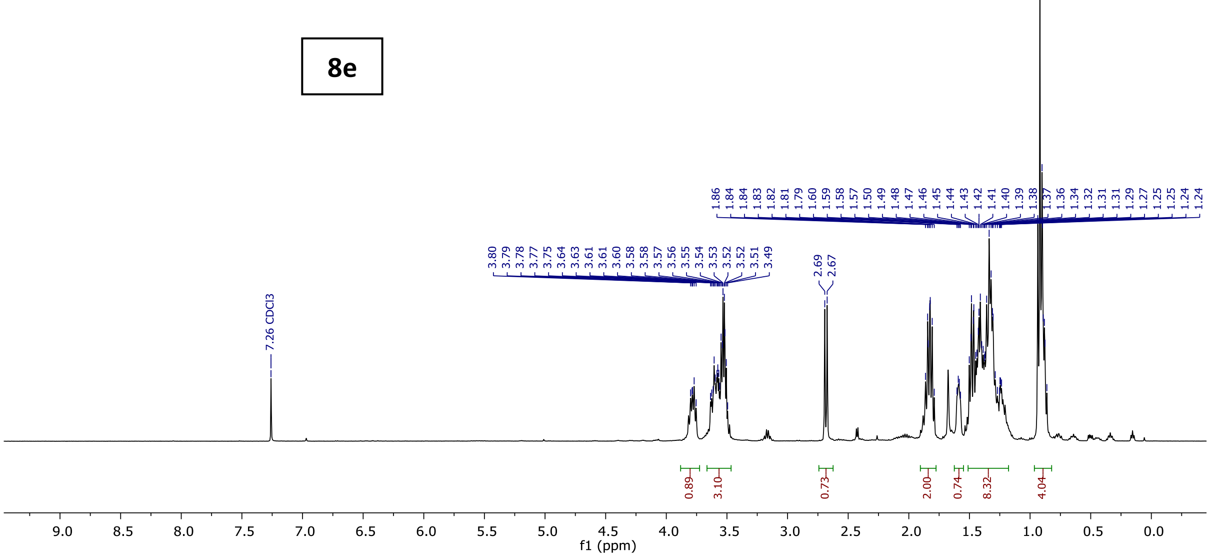
0.94
0.92
0.90
0.90
0.89
0.88
0.88
0.86
1.86
1.84
1.84
1.83
1.82
1.81
1.79
1.60
1.59
1.58
1.57
1.50
1.49
1.48
1.47
1.46
1.45
1.44
1.43
1.42
1.41
1.40
1.39
1.38
1.37
1.36
1.34
1.32
1.31
1.31
1.29
1.27
1.25
1.25
1.24
1.24
3.80
3.79
3.78
3.77
3.75
3.64
3.63
3.61
3.61
3.60
3.58
3.58
3.57
3.56
3.55
3.54
3.53
3.52
3.52
3.51
3.49
2.69
2.67
7.26 CDCl3
0.89
3.10
0.73
2.00
0.74
8.32
4.04
9.0
8.5
8.0
7.5
7.0
6.5
6.0
5.5
5.0
4.5
4.0
3.5
3.0
2.5
2.0
1.5
1.0
0.5
0.0
f1 (ppm)

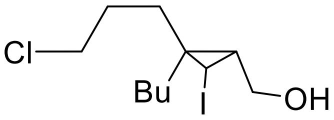
Cl
Bu
I
OH

8e

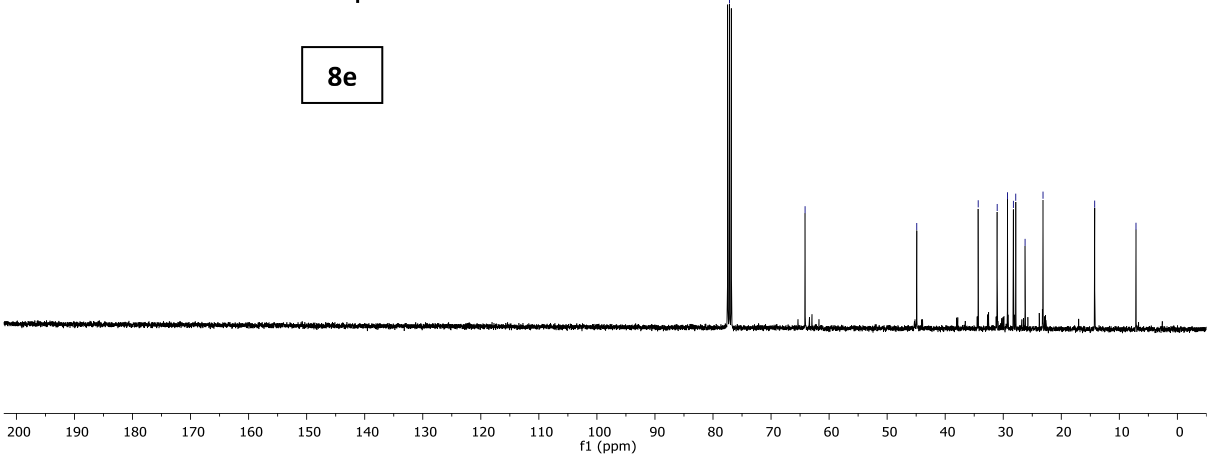
77.16 CDCl3
64.14
44.90
34.31
31.04
29.27
28.25
27.84
26.24
23.15
14.25
7.13
200
190
180
170
160
150
140
130
120
110
100
90
80
70
60
50
40
30
20
10
0
f1 (ppm)

8f

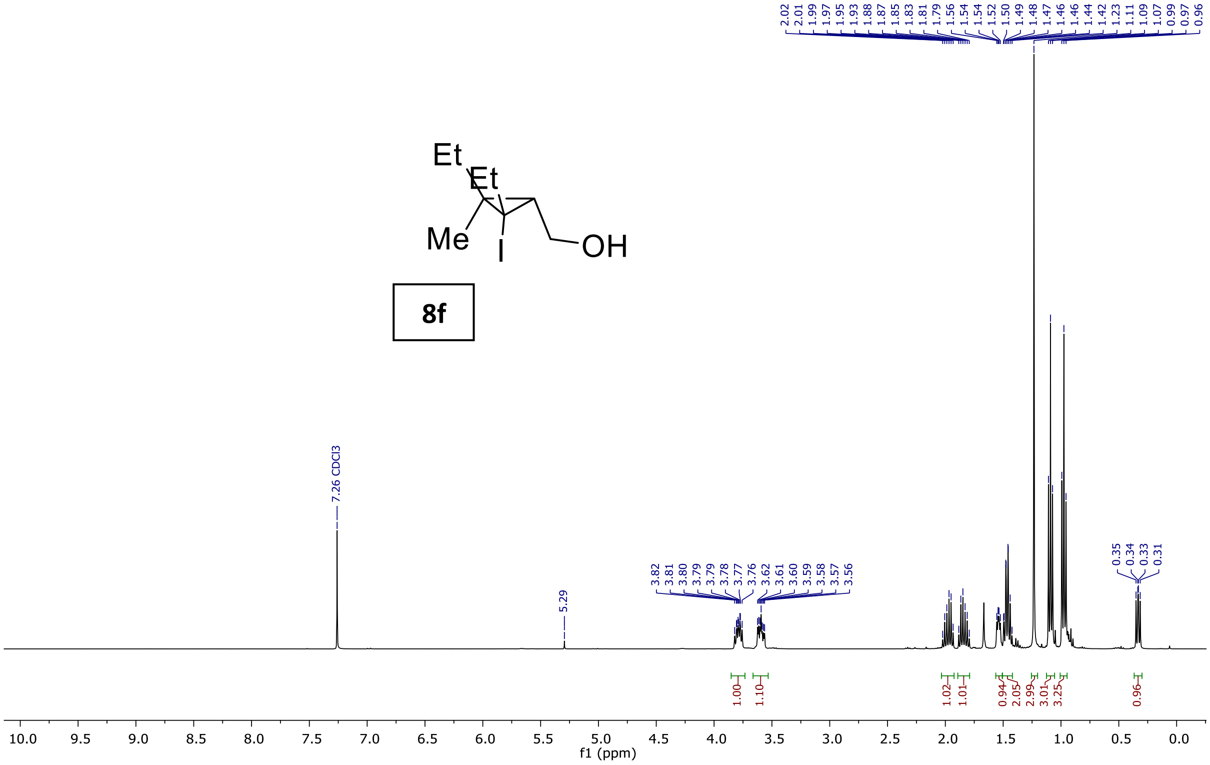
2.02 2.01 1.99 1.97 1.95 1.93 1.88 1.87 1.85 1.83 1.81 1.79 1.56 1.54 1.54 1.52 1.50 1.49 1.48 1.47 1.46 1.46 1.44 1.42 1.23 1.11 1.09 1.07 0.99 0.97 0.96
7.26 CDCl3
5.29
3.82 3.81 3.80 3.79 3.79 3.78 3.77 3.76 3.62 3.61 3.60 3.59 3.58 3.57 3.56
0.35 0.34 0.33 0.31
1.00 1.10 1.02 1.01 0.94 2.05 2.99 3.01 3.25 0.96
10.0 9.5 9.0 8.5 8.0 7.5 7.0 6.5 6.0 5.5 5.0 4.5 4.0 3.5 3.0 2.5 2.0 1.5 1.0 0.5 0.0
f1 (ppm)

8f

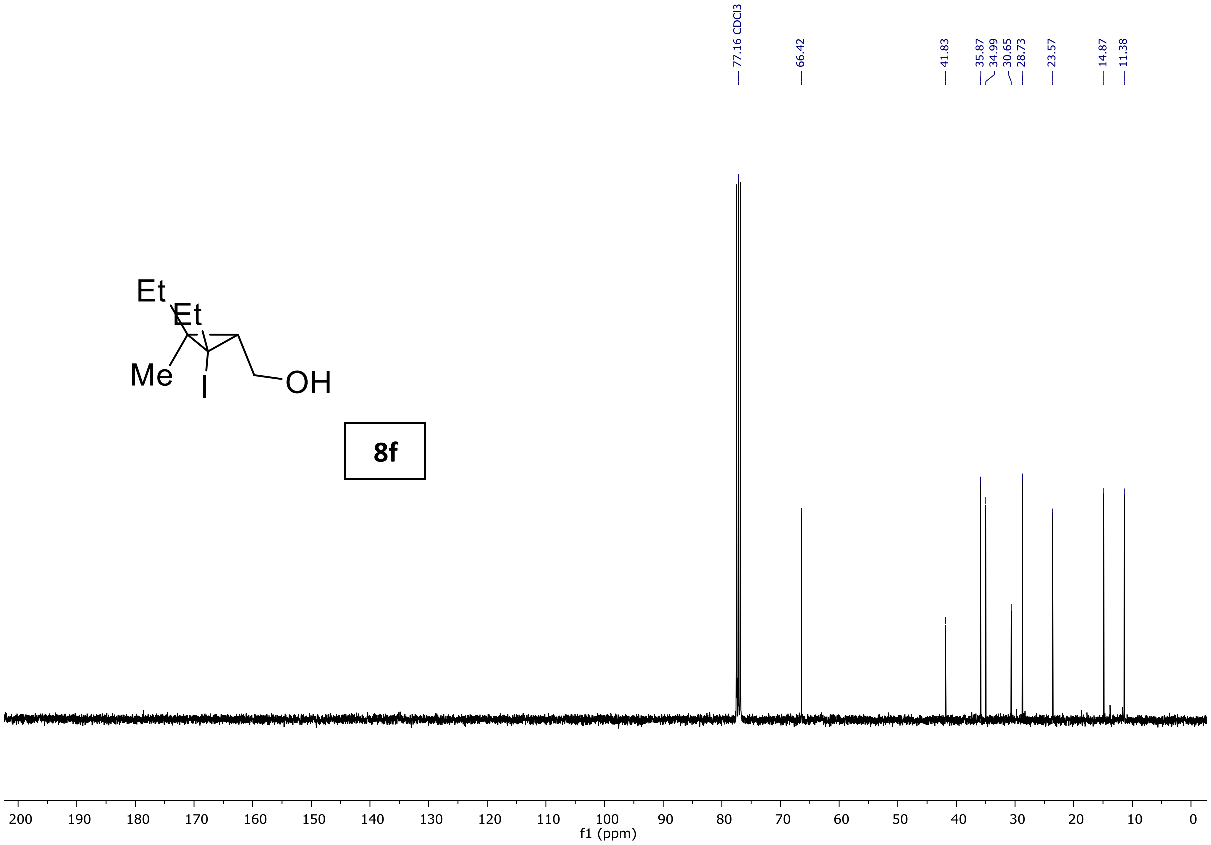
77.16 CDCl3
66.42
41.83
35.87 34.99 30.65 28.73
23.57
14.87
11.38
200 190 180 170 160 150 140 130 120 110 100 90 80 70 60 50 40 30 20 10 0
f1 (ppm)

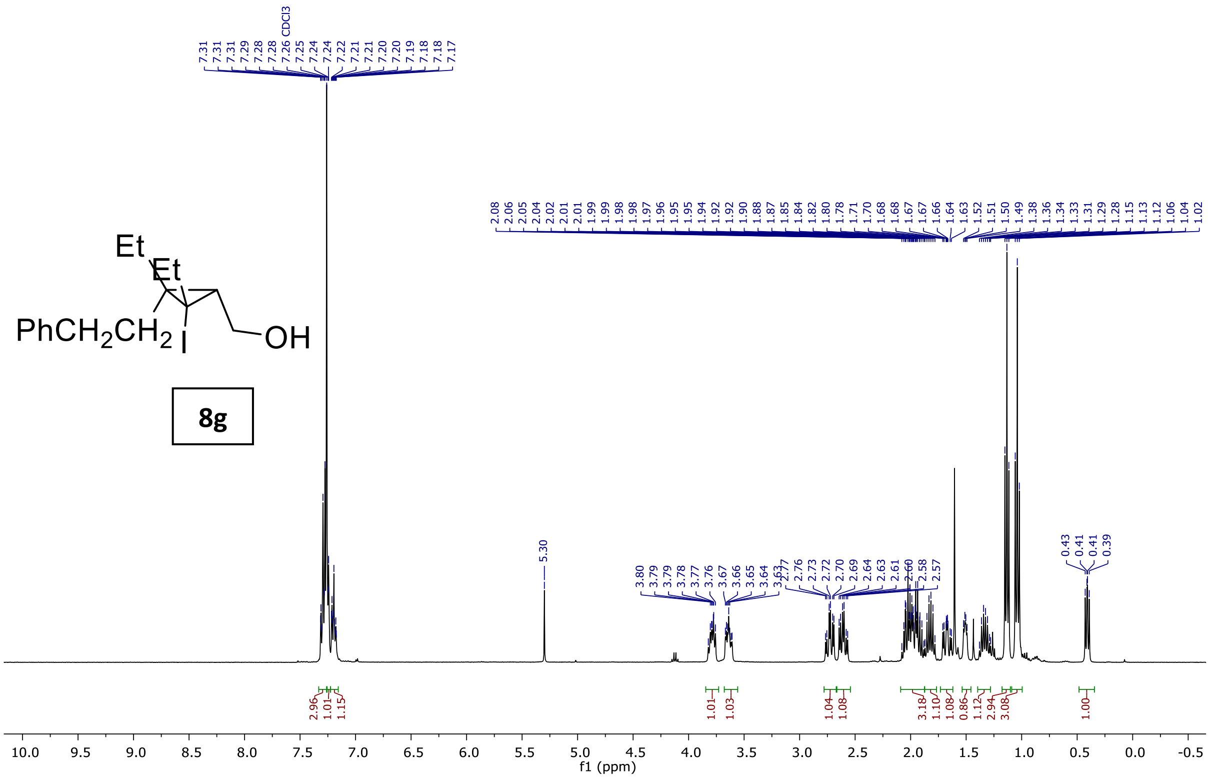
Et
Et
PhCH2CH2
I
OH
8g
7.31 7.31 7.31 7.29 7.28 7.28 7.26 CDCl3 7.25 7.24 7.24 7.22 7.21 7.21 7.20 7.20 7.19 7.18 7.18 7.17
2.08 2.06 2.05 2.04 2.02 2.01 1.99 1.99 1.98 1.98 1.97 1.96 1.95 1.95 1.94 1.92 1.92 1.90 1.88 1.87 1.85 1.84 1.82 1.80 1.78 1.71 1.70 1.68 1.68 1.67 1.67 1.66 1.64 1.63 1.52 1.51 1.50 1.49 1.38 1.36 1.34 1.33 1.31 1.29 1.28 1.15 1.13 1.12 1.06 1.04 1.02
5.30
3.80 3.79 3.79 3.78 3.77 3.76 3.67 3.66 3.65 3.64 3.63
2.77 2.76 2.73 2.72 2.70 2.69 2.64 2.63 2.61 2.60 2.58 2.57
0.43 0.41 0.41 0.39
2.96 1.01 1.15 1.01 1.03 1.04 1.08 3.18 1.10 1.08 0.86 1.12 2.94 3.08 1.00
10.0 9.5 9.0 8.5 8.0 7.5 7.0 6.5 6.0 5.5 5.0 4.5 4.0 3.5 3.0 2.5 2.0 1.5 1.0 0.5 0.0 -0.5
f1 (ppm)

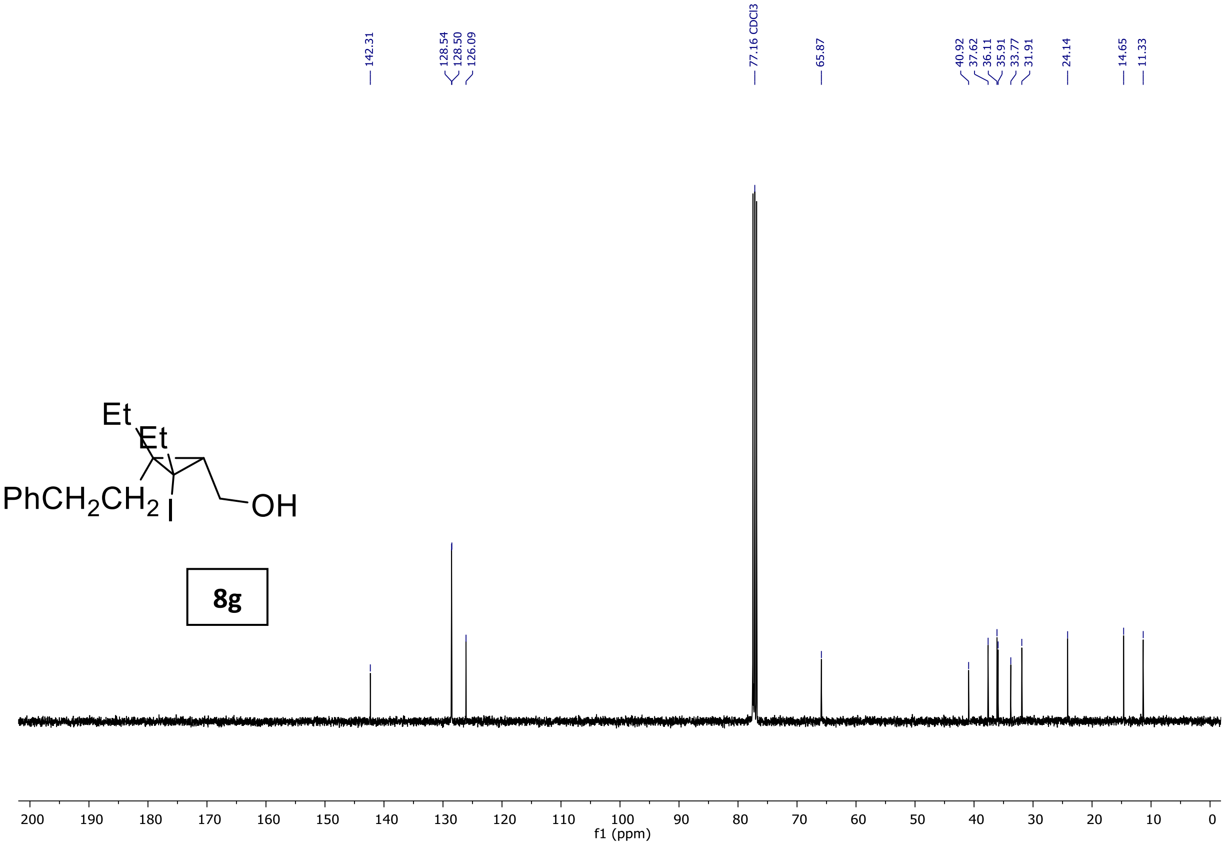
142.31
128.54 128.50 126.09
77.16 CDCl3
65.87
40.92 37.62 36.11 35.91 33.77 31.91
24.14
14.65 11.33
Et
Et
PhCH2CH2
I
OH
8g
200 190 180 170 160 150 140 130 120 110 100 90 80 70 60 50 40 30 20 10 0
f1 (ppm)

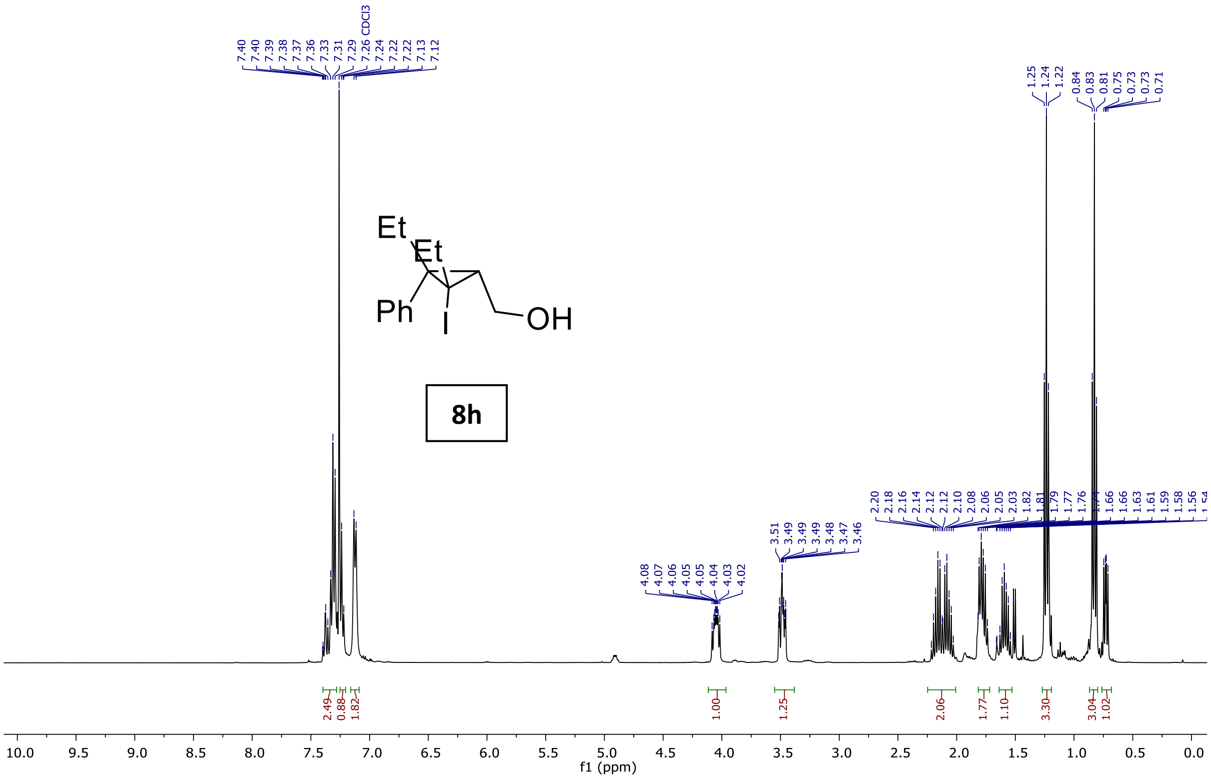
7.40 7.40 7.39 7.38 7.37 7.36 7.33 7.31 7.29 7.26 CDCl3 7.24 7.22 7.22 7.13 7.12
1.25 1.24 1.22 0.84 0.83 0.81 0.75 0.73 0.73 0.71
Et
Et
Ph
I
OH
8h
4.08 4.07 4.06 4.05 4.05 4.04 4.03 4.02
3.51 3.49 3.49 3.48 3.47 3.46
2.49 0.88 1.82 1.00 1.25 2.06 1.77 1.10 3.30 3.04 1.02
10.0 9.5 9.0 8.5 8.0 7.5 7.0 6.5 6.0 5.5 5.0 4.5 4.0 3.5 3.0 2.5 2.0 1.5 1.0 0.5 0.0
f1 (ppm)

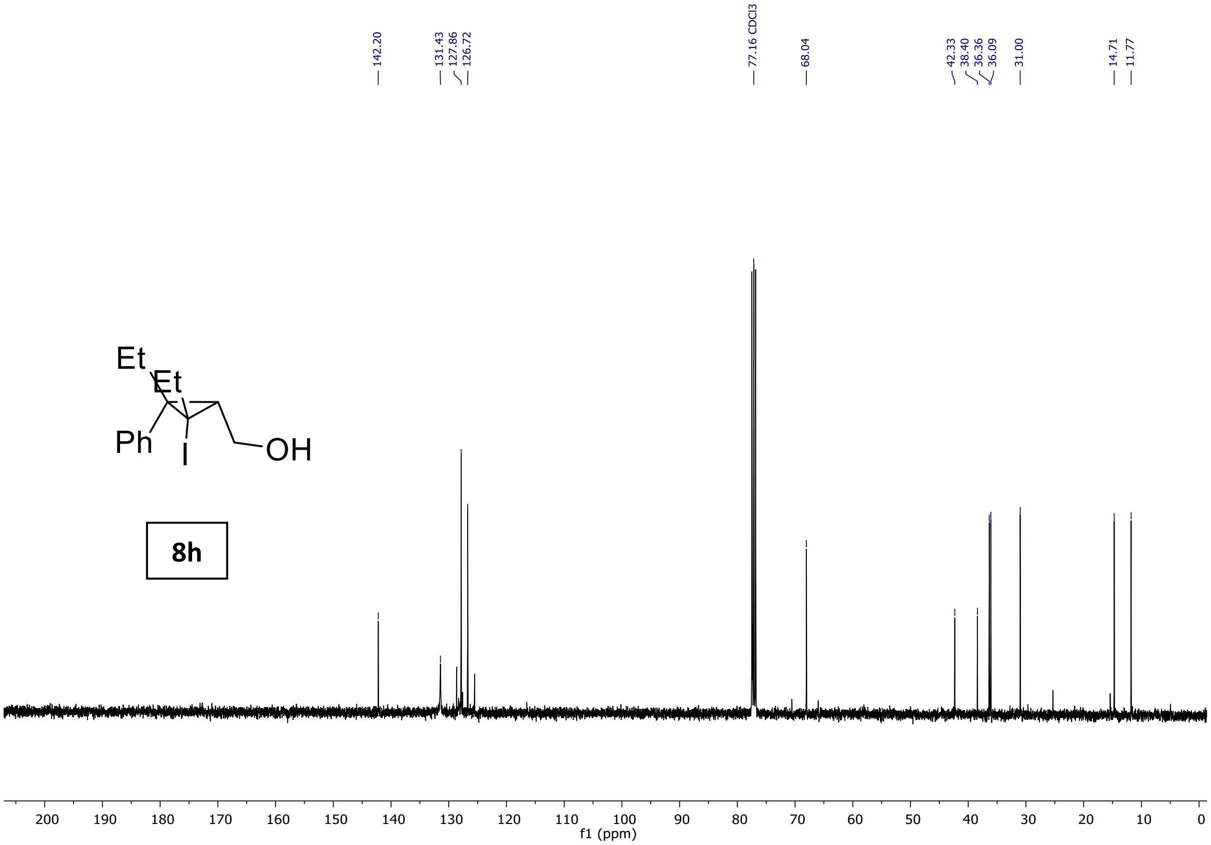
142.20
131.43 127.86 126.72
77.16 CDCl3
68.04
42.33 38.40 36.36 36.09
31.00
14.71 11.77
Et
Et
Ph
I
OH
8h
200 190 180 170 160 150 140 130 120 110 100 90 80 70 60 50 40 30 20 10 0
f1 (ppm)

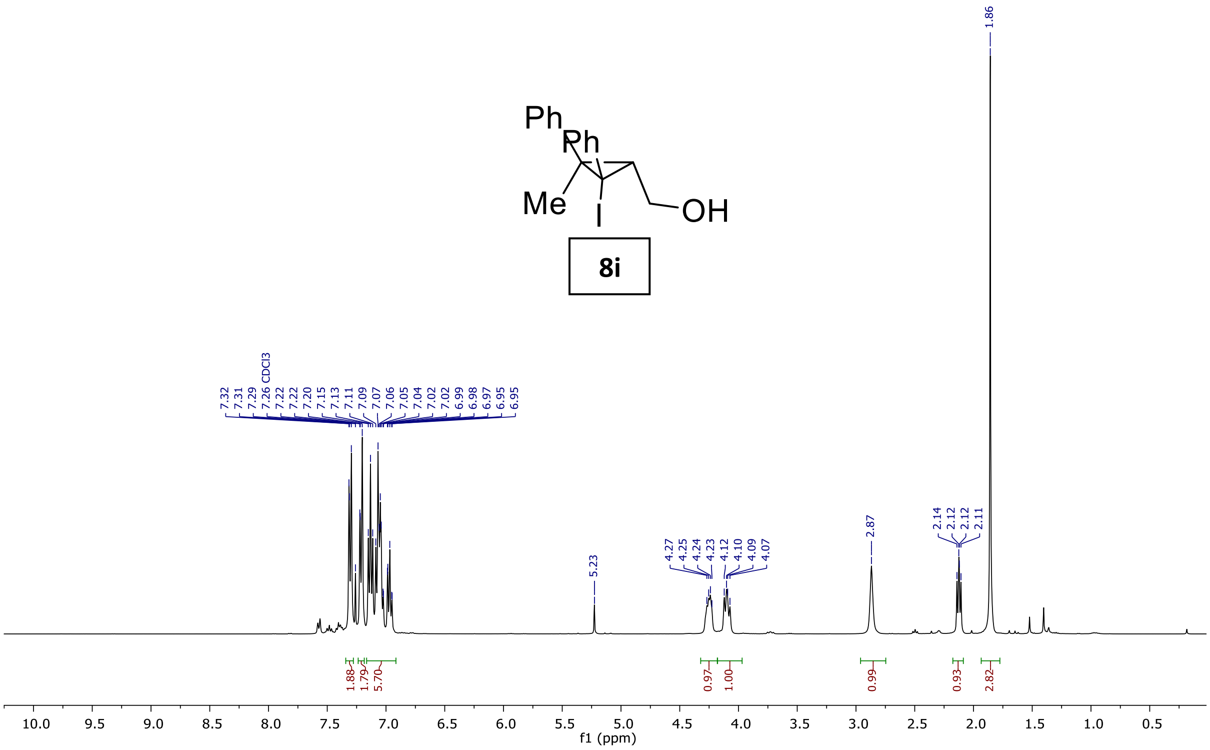

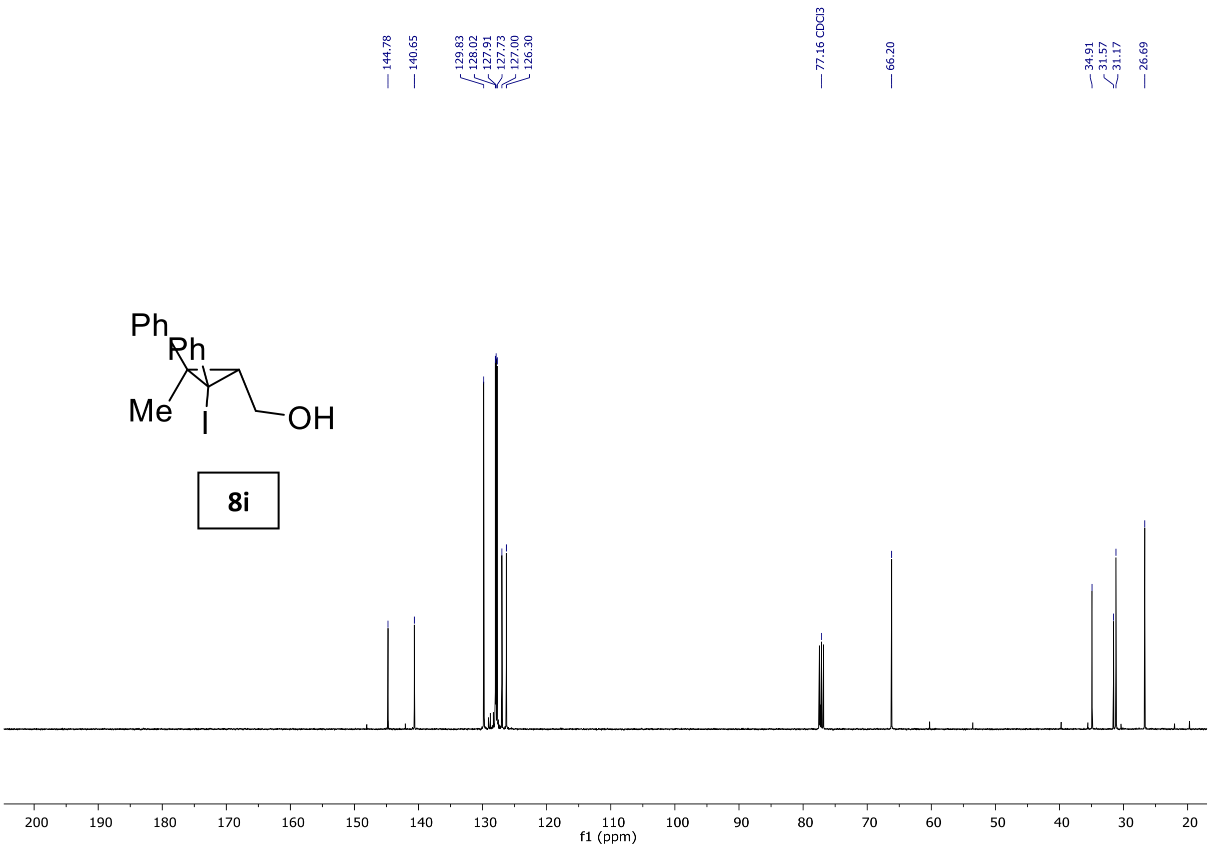

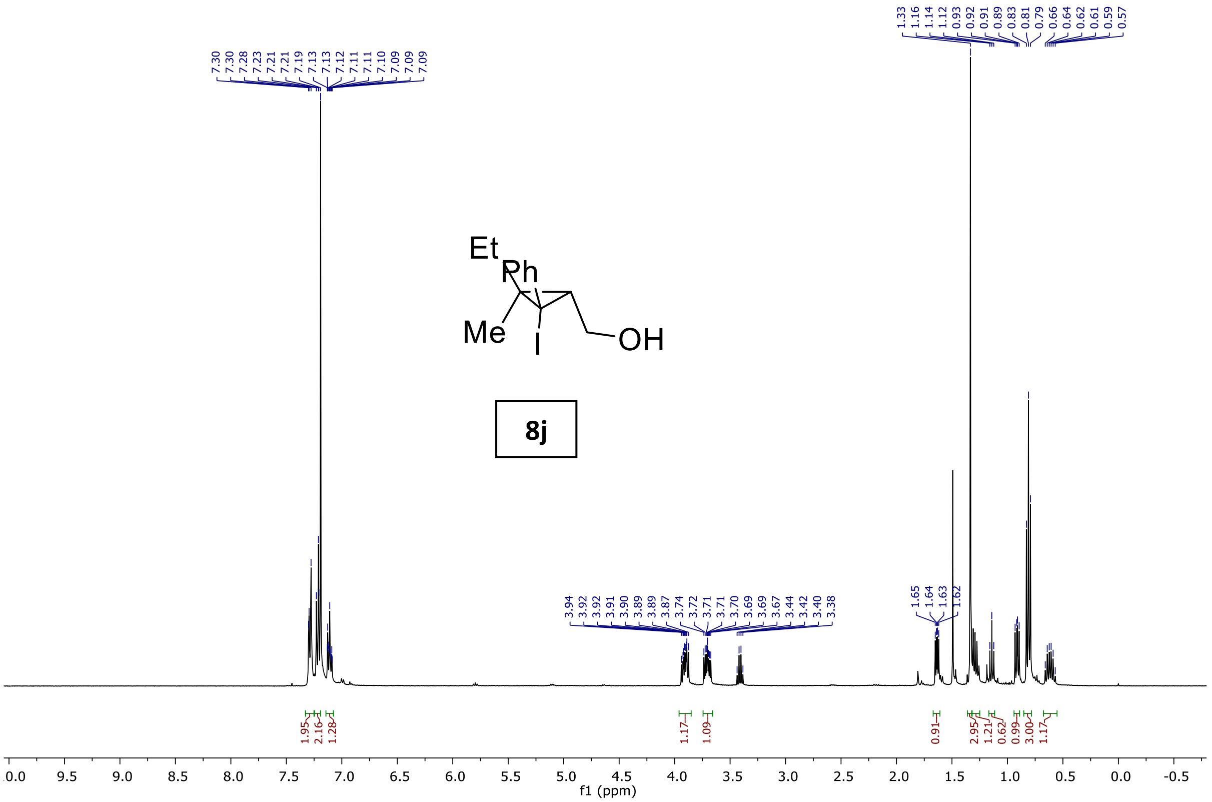

8j

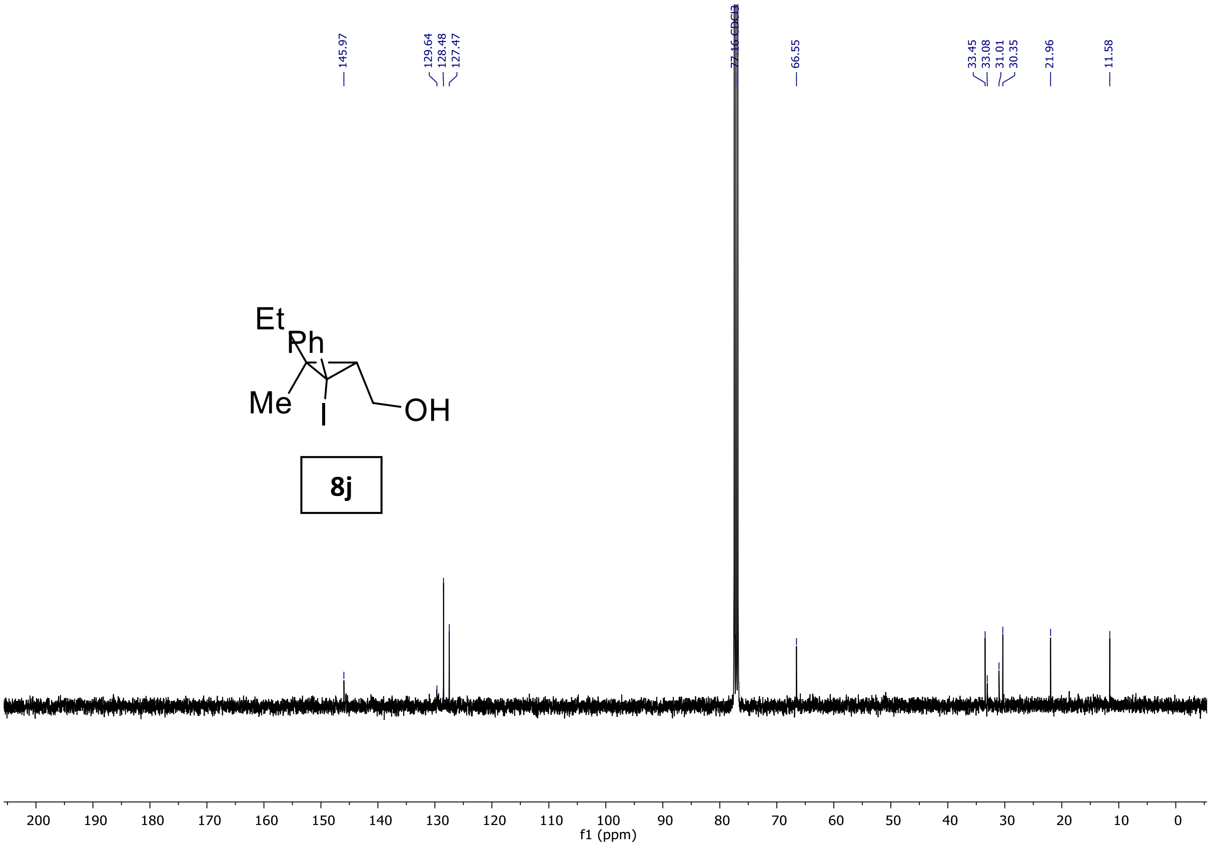

8j

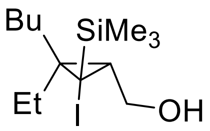

8k

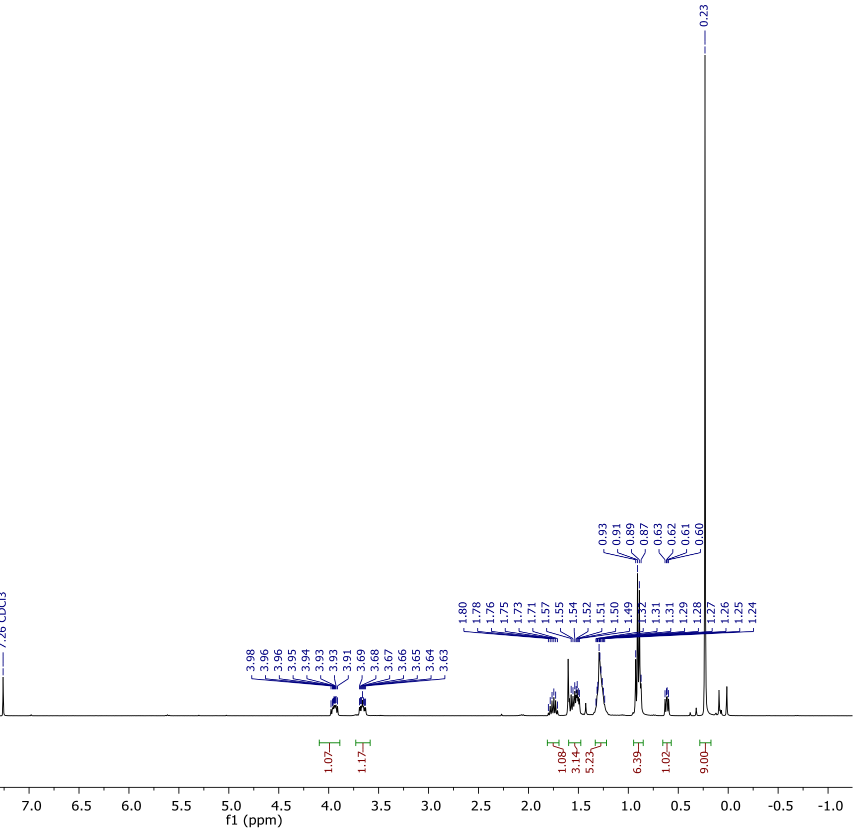
7.26 CDCl3
3.98 3.96 3.96 3.95 3.94 3.93 3.93 3.91 3.69 3.68 3.67 3.66 3.65 3.64 3.63
1.80 1.78 1.76 1.75 1.73 1.71 1.57 1.55 1.54 1.52 1.51 1.50 1.49 1.32 1.31 1.31 1.29 1.28 1.27 1.26 1.25 1.24
0.93 0.91 0.89 0.87 0.63 0.62 0.61 0.60
0.23
f1 (ppm)

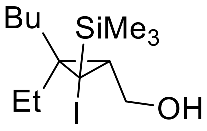

8k

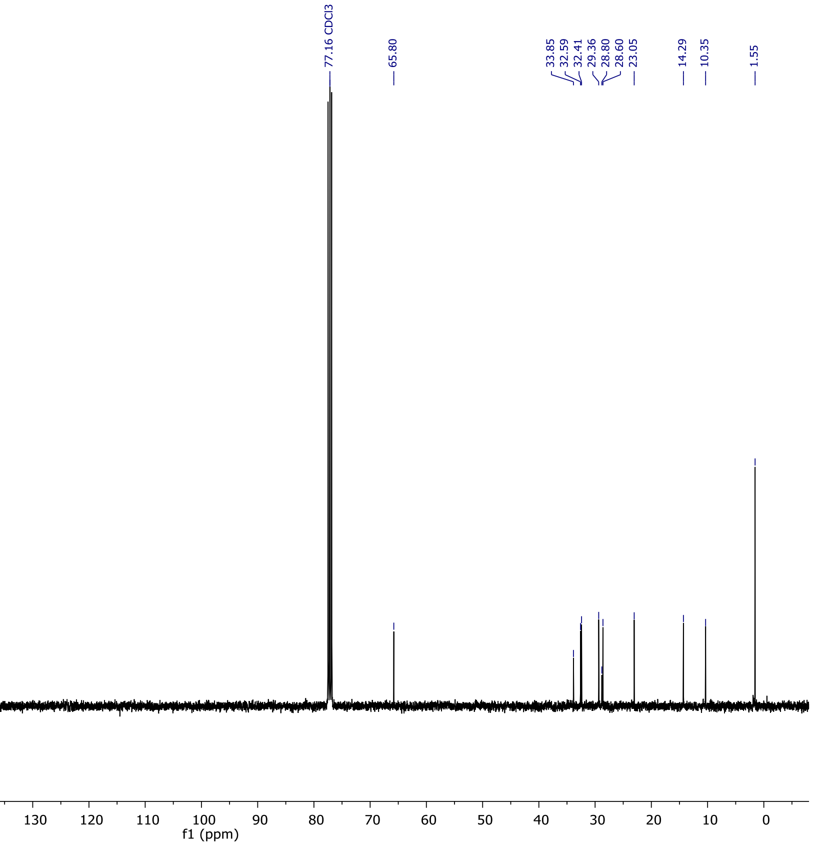
77.16 CDCl3
65.80
33.85 32.59 32.41 29.36 28.80 28.60 23.05
14.29
10.35
1.55
f1 (ppm)

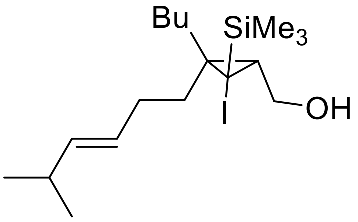
Bu
SiMe3
I
OH

8l

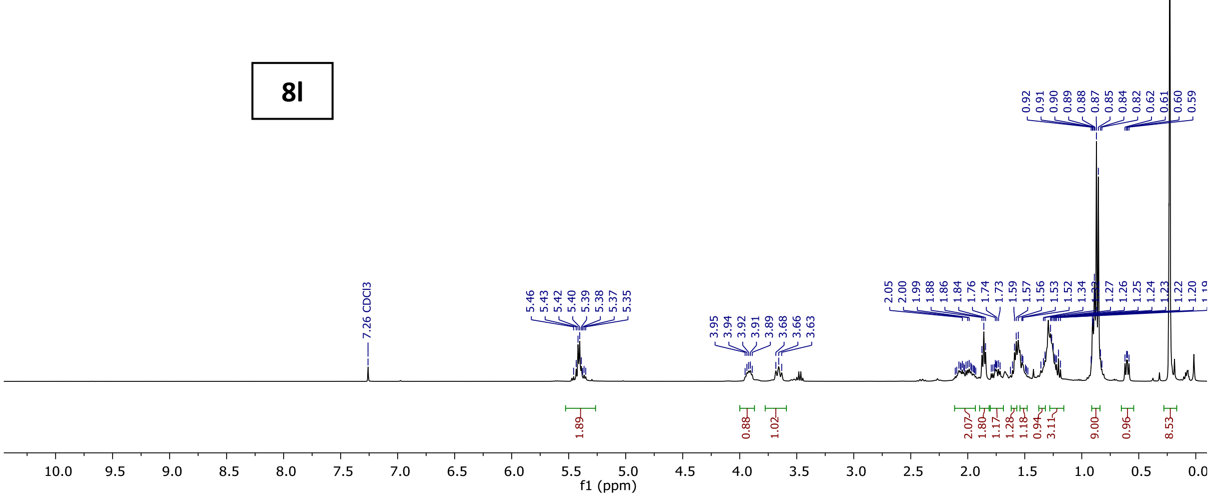
7.26 CDCl3
5.46 5.43 5.42 5.40 5.39 5.38 5.37 5.35
3.95 3.94 3.92 3.91 3.89 3.68 3.66 3.63
2.05 2.00 1.99 1.88 1.86 1.84 1.76 1.74 1.73 1.59 1.57 1.56 1.53 1.52 1.34 1.27 1.26 1.25 1.24 1.23 1.22 1.20
0.92 0.91 0.90 0.89 0.88 0.87 0.85 0.84 0.82 0.62 0.61 0.60 0.59
0.23
1.89
0.88
1.02
2.07 1.80 1.17 1.28 1.18 0.94 3.11
9.00
0.96
8.53
f1 (ppm)

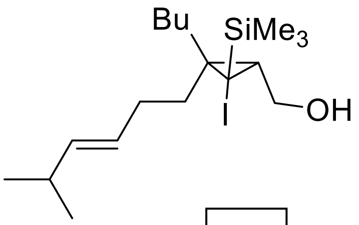
Bu
SiMe3
I
OH

8l

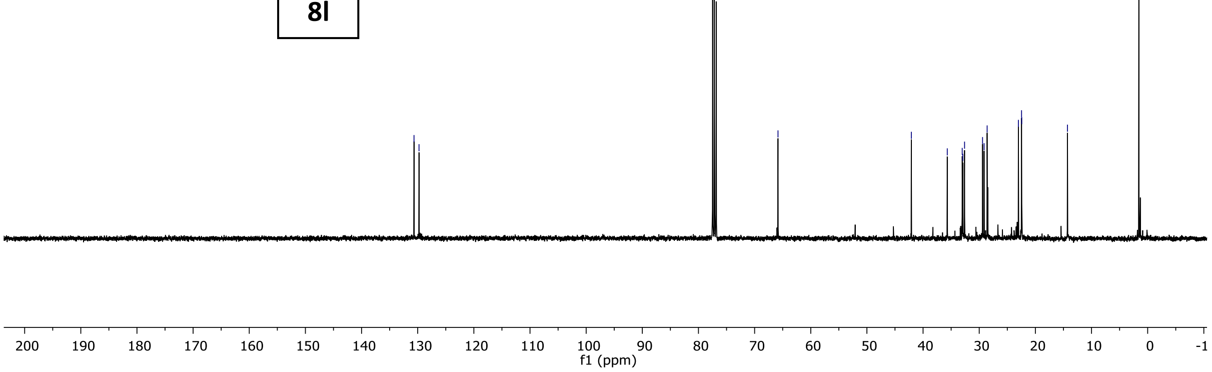
130.66
129.77
77.16 CDCl3
65.83
42.09 35.69 33.03 32.93 32.61 29.41 29.13 28.59 23.02 22.46 22.43
14.28
1.56
f1 (ppm)

8m*

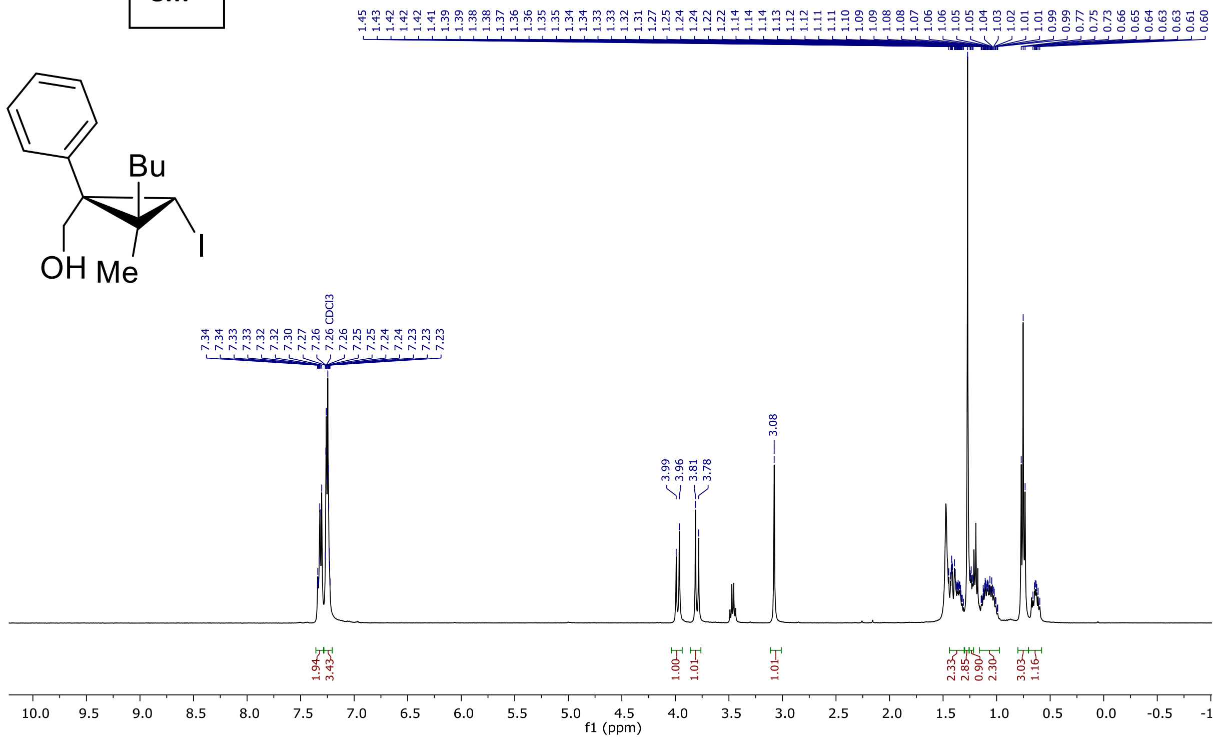

Bu
OH
Me
I
3.99
3.96
3.81
3.78
3.08
1.94
3.43
1.00
1.01
1.01
2.33
2.85
0.90
2.30
3.03
1.16
f1 (ppm)

8m*

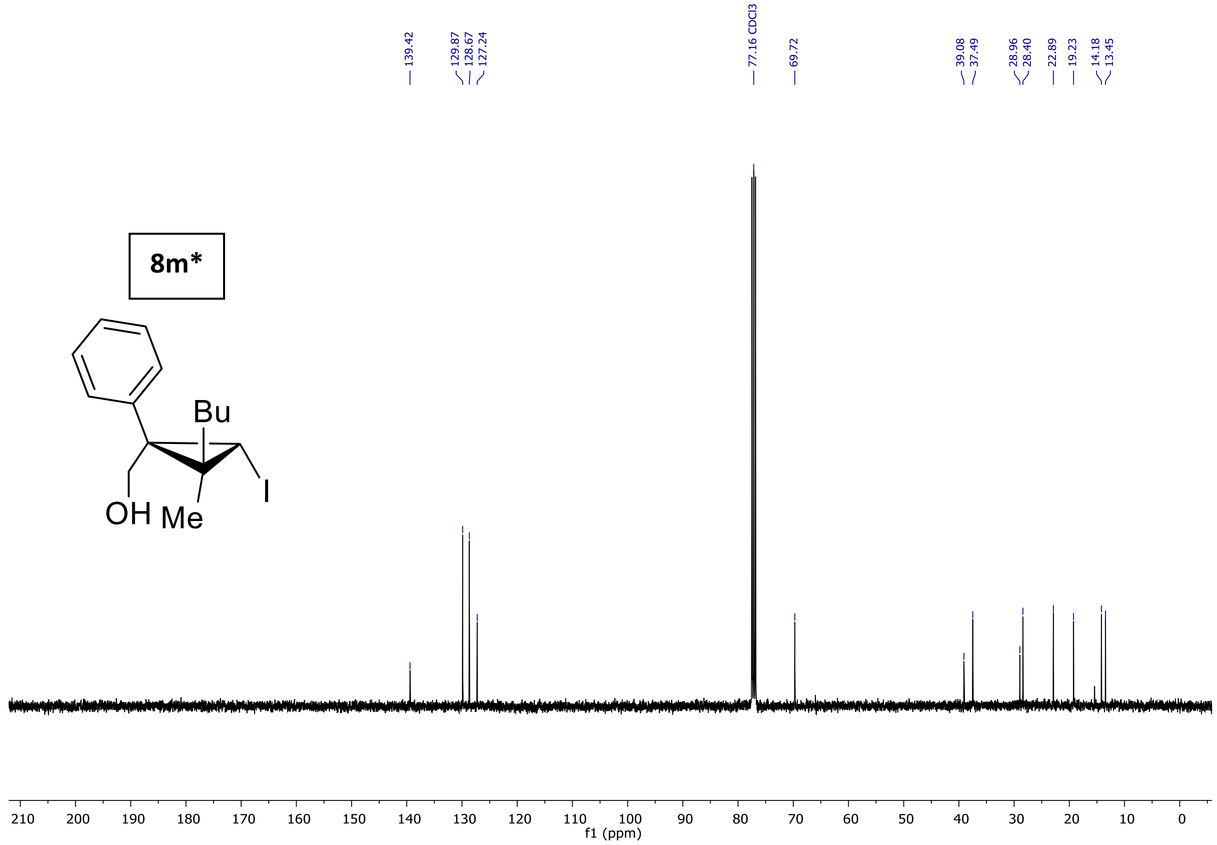

Bu
OH
Me
I
139.42
129.87
128.67
127.24
77.16 CDCl3
69.72
39.08
37.49
28.96
28.40
22.89
19.23
14.18
13.45
f1 (ppm)

HPLC chromatogram of racemic compound (8m)

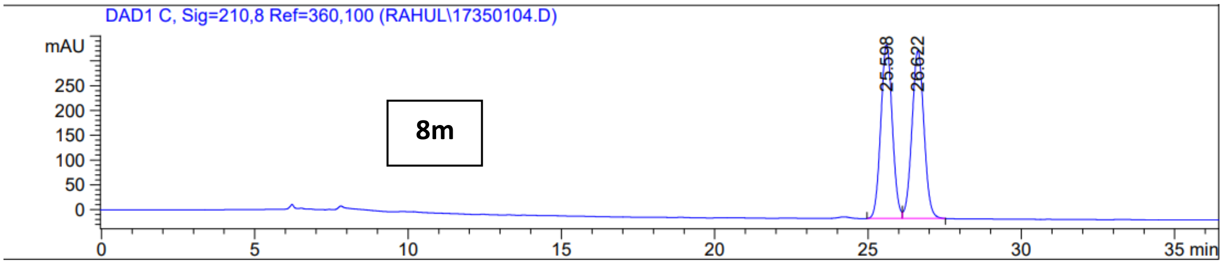

Signal 3: DAD1 C, Sig=210,8 Ref=360,100

Peak #	RetTime [min]	Type	Width [min]	Area [mAU*s]	Height [mAU]	Area %
1	25.598	BV	0.4061	9145.24121	350.89844	49.7259
2	26.622	VB	0.4246	9246.06738	338.63052	50.2741
Totals :				1.83913e4	689.52896	

CHIRACEL OX-H, column size: 0.46cmI.D x 25 cmL (99:1 n-Hexane/IPA, 1 mL/min, 20 °C)

HPLC chromatogram of enantioenriched compound (8m*)

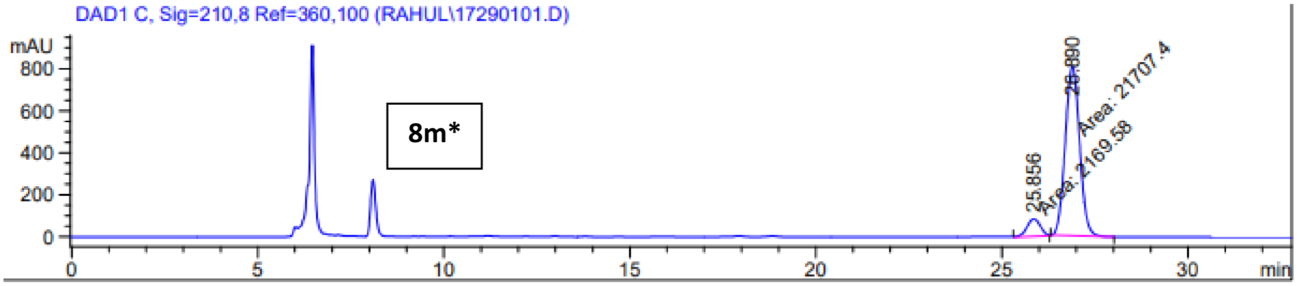

Signal 3: DAD1 C, Sig=210,8 Ref=360,100

Peak #	RetTime [min]	Type	Width [min]	Area [mAU*s]	Height [mAU]	Area %
1	25.856	MM	0.4265	2169.58032	84.78465	9.0865
2	26.890	MM	0.4475	2.17074e4	808.41235	90.9135
Totals :				2.38770e4	893.19700	

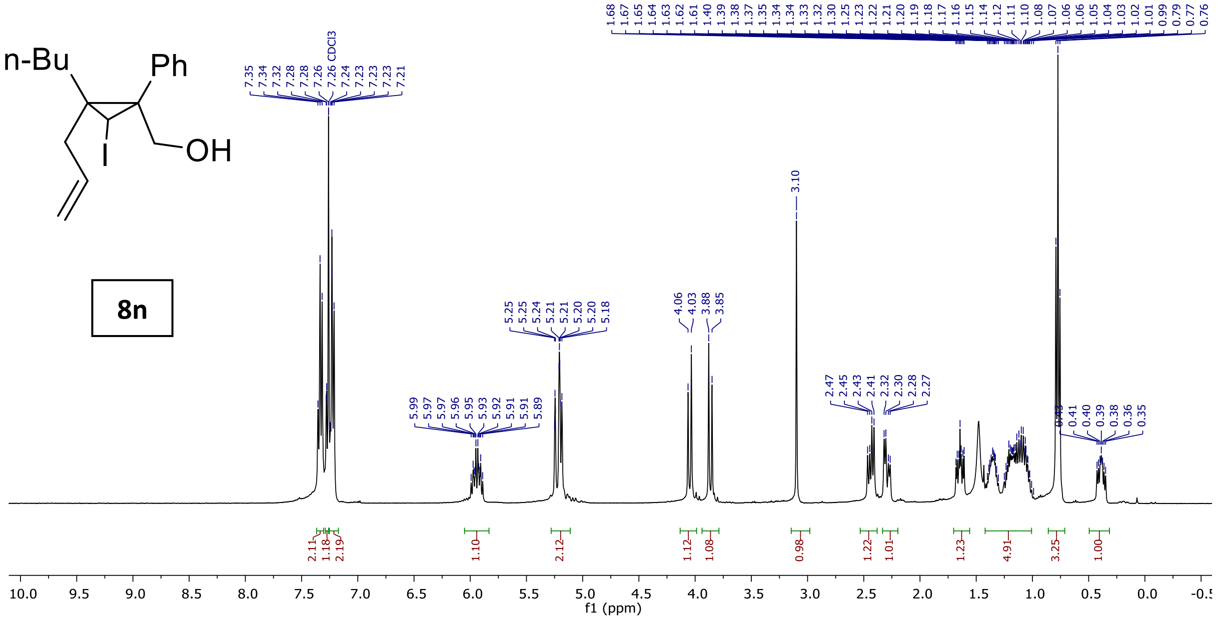
n-Bu
Ph
I
OH
8n
f1 (ppm)

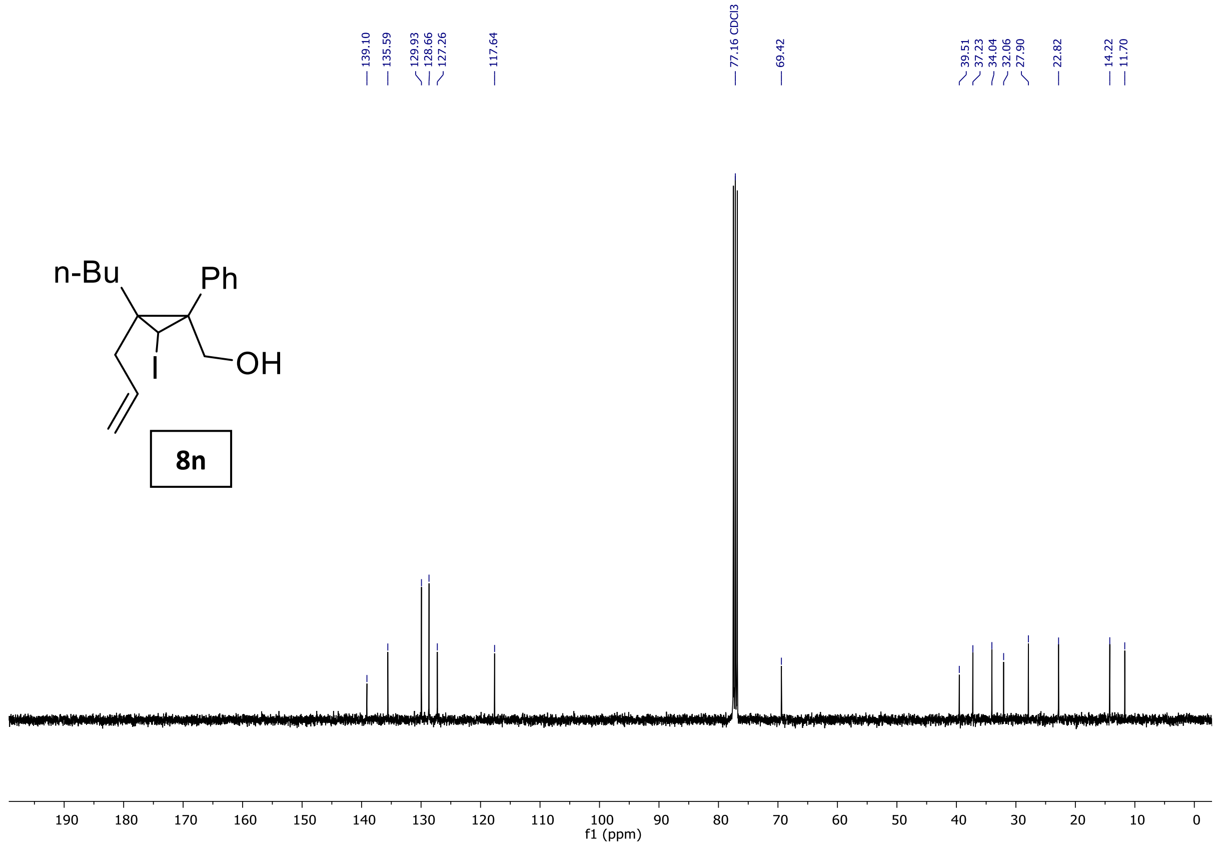
139.10
135.59
129.93
128.66
127.26
117.64
77.16 CDCl3
69.42
39.51
37.23
34.04
32.06
27.90
22.82
14.22
11.70
n-Bu
Ph
I
OH
8n
f1 (ppm)

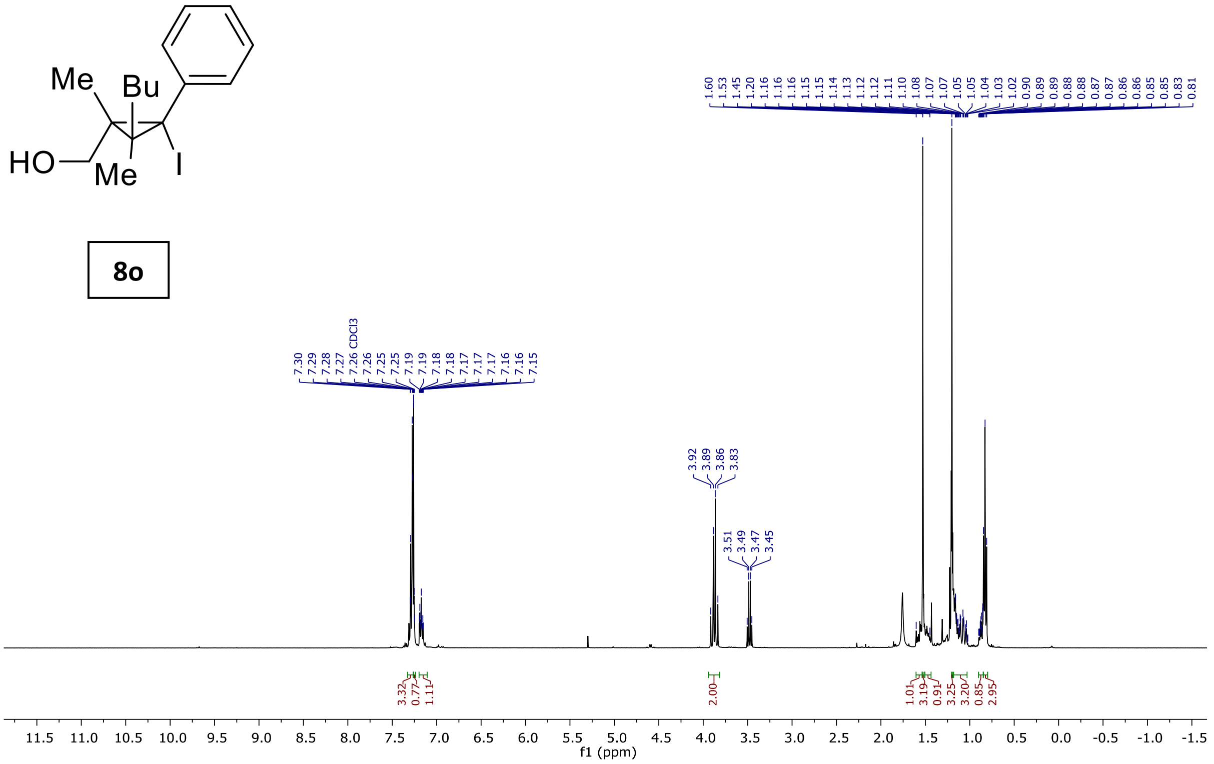
Me
Bu
HO
Me
I
8o
1.60 1.53 1.45 1.20 1.16 1.16 1.16 1.15 1.15 1.14 1.13 1.12 1.12 1.11 1.10 1.08 1.07 1.07 1.05 1.05 1.04 1.03 1.02 0.90 0.89 0.89 0.88 0.88 0.87 0.87 0.86 0.85 0.85 0.83 0.81
7.30 7.29 7.28 7.27 7.26 CDCl3 7.26 7.25 7.25 7.19 7.19 7.18 7.18 7.17 7.17 7.17 7.16 7.16 7.15
3.92 3.89 3.86 3.83
3.51 3.49 3.47 3.45
3.32 0.77 1.11
2.00
1.01 3.19 0.91 3.25 3.20 0.85 2.95
11.5 11.0 10.5 10.0 9.5 9.0 8.5 8.0 7.5 7.0 6.5 6.0 5.5 5.0 4.5 4.0 3.5 3.0 2.5 2.0 1.5 1.0 0.5 0.0 -0.5 -1.0 -1.5
f1 (ppm)

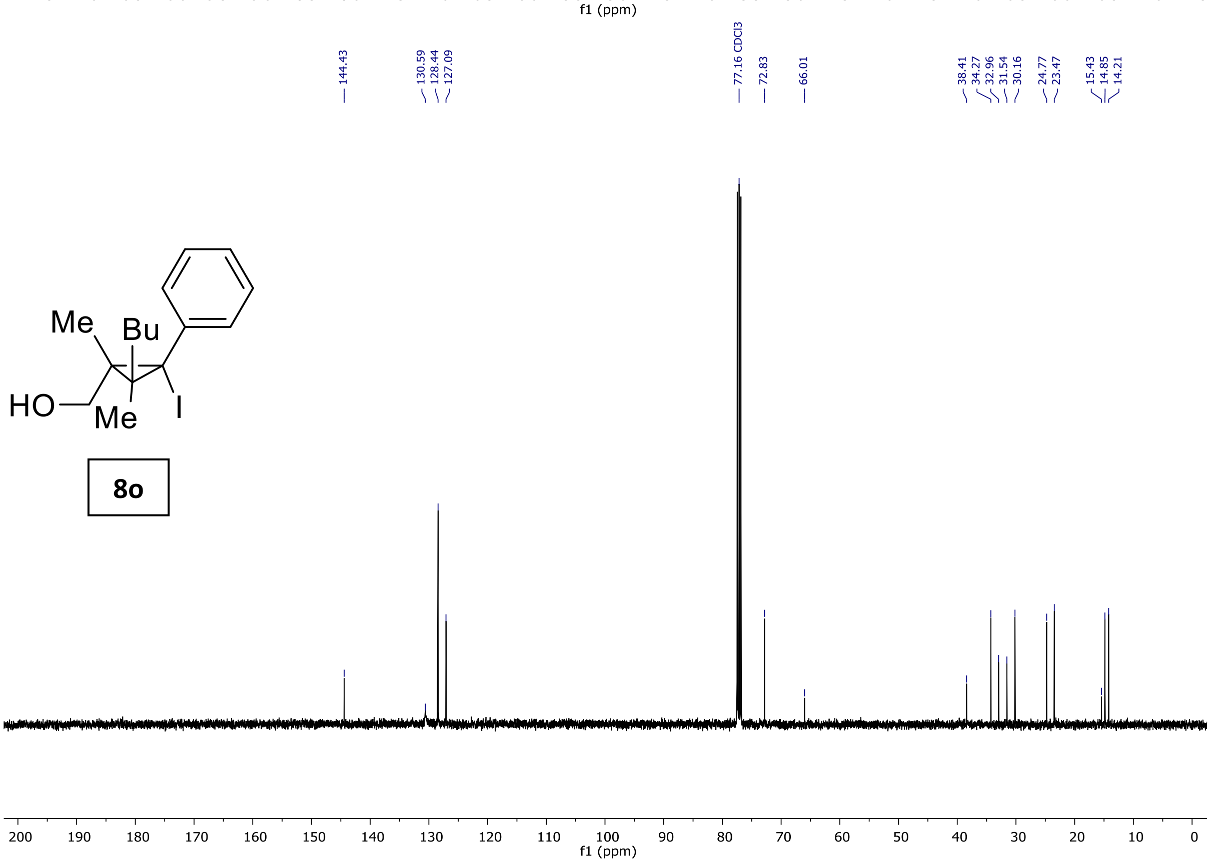
144.43
130.59 128.44 127.09
77.16 CDCl3
72.83
66.01
38.41 34.27 32.96 31.54 30.16
24.77 23.47
15.43 14.85 14.21
Me
Bu
HO
Me
I
8o
200 190 180 170 160 150 140 130 120 110 100 90 80 70 60 50 40 30 20 10 0
f1 (ppm)

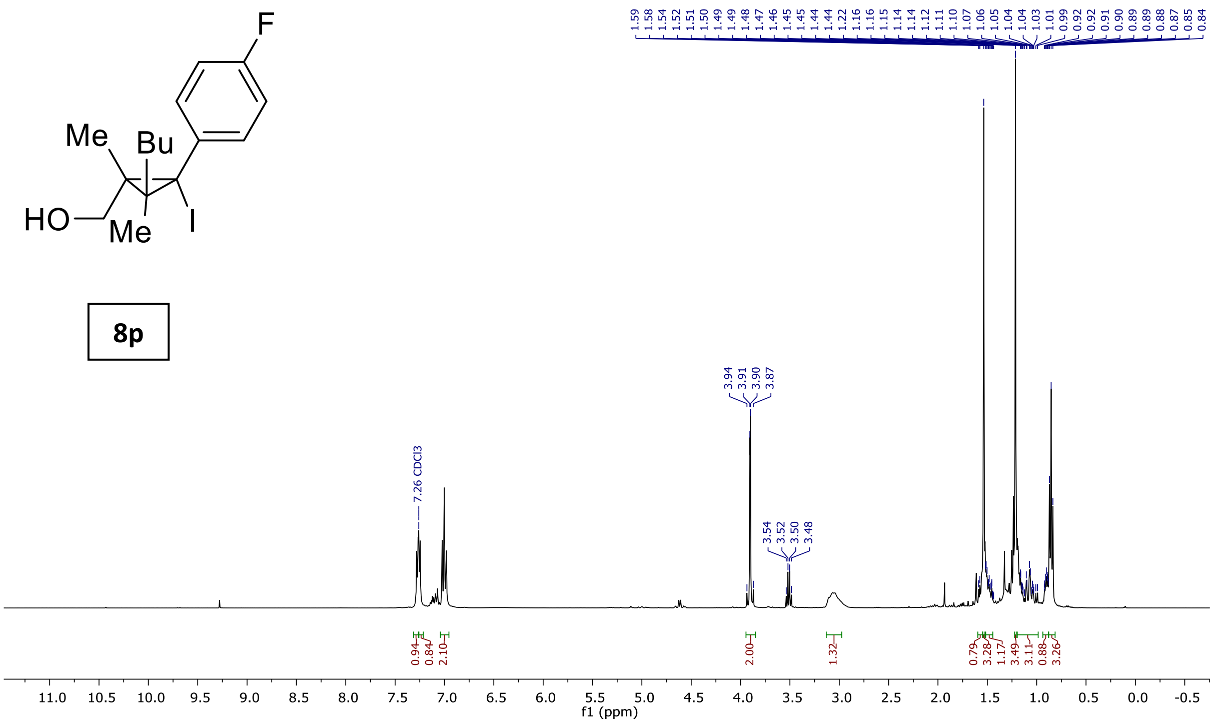

F
Me
Bu
HO
Me
I
8p
7.26 CDCl3
3.94
3.91
3.90
3.87
3.54
3.52
3.50
3.48
f1 (ppm)

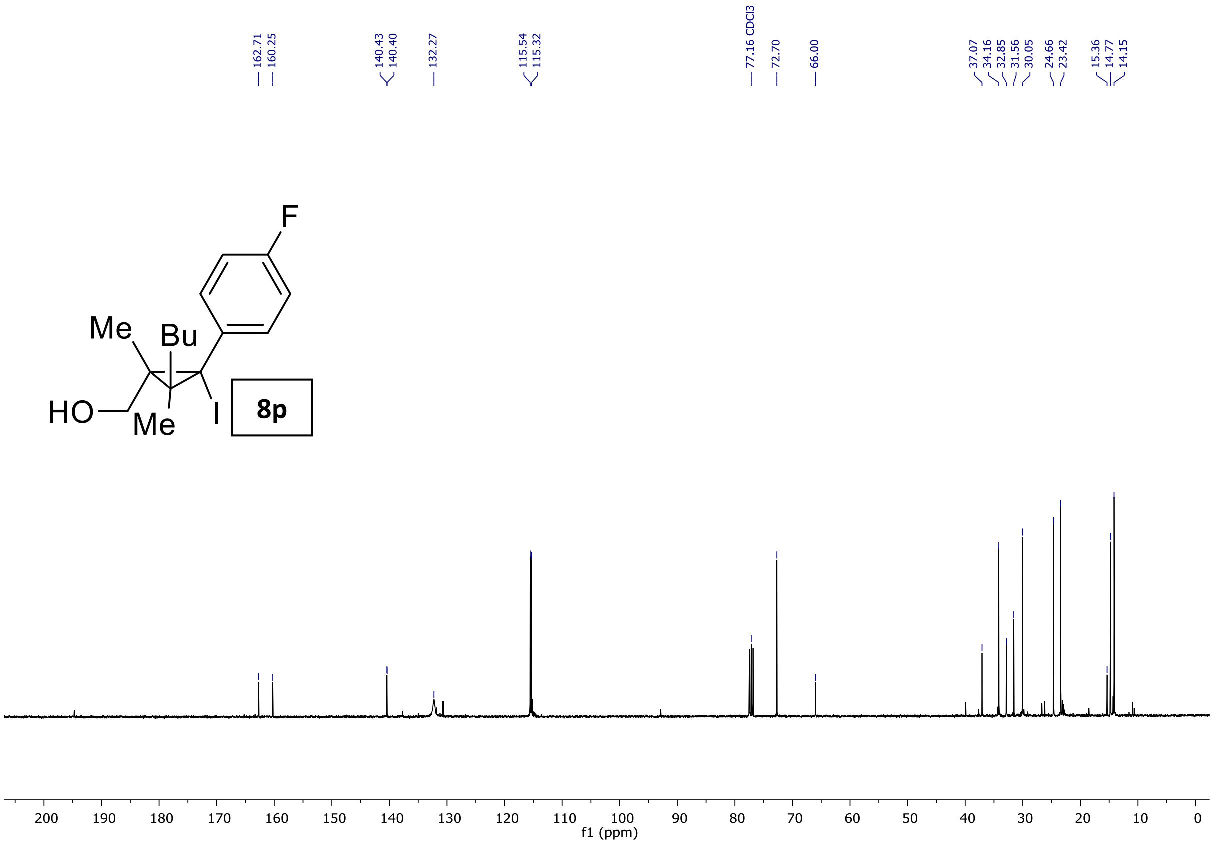

162.71
160.25
140.43
140.40
132.27
115.54
115.32
77.16 CDCl3
72.70
66.00
37.07
34.16
32.85
31.56
30.05
24.66
23.42
15.36
14.77
14.15
F
Me
Bu
HO
Me
I
8p
f1 (ppm)

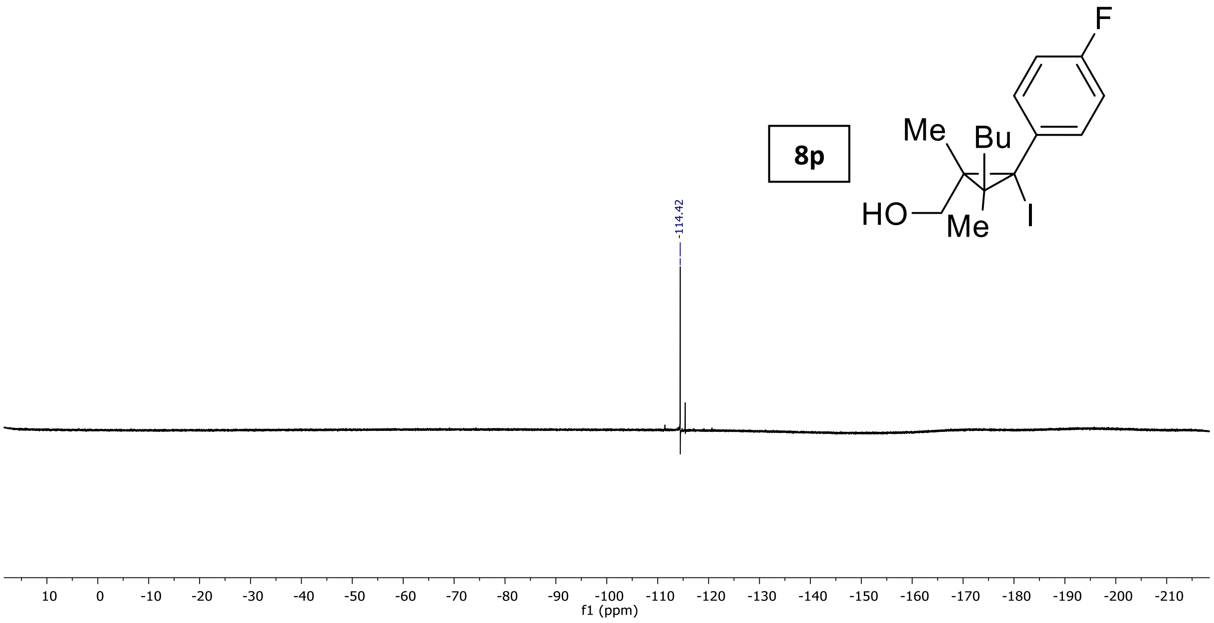
8p
F
Me
Bu
HO
Me
I
-114.42
10 0 -10 -20 -30 -40 -50 -60 -70 -80 -90 -100 -110 -120 -130 -140 -150 -160 -170 -180 -190 -200 -210
f1 (ppm)

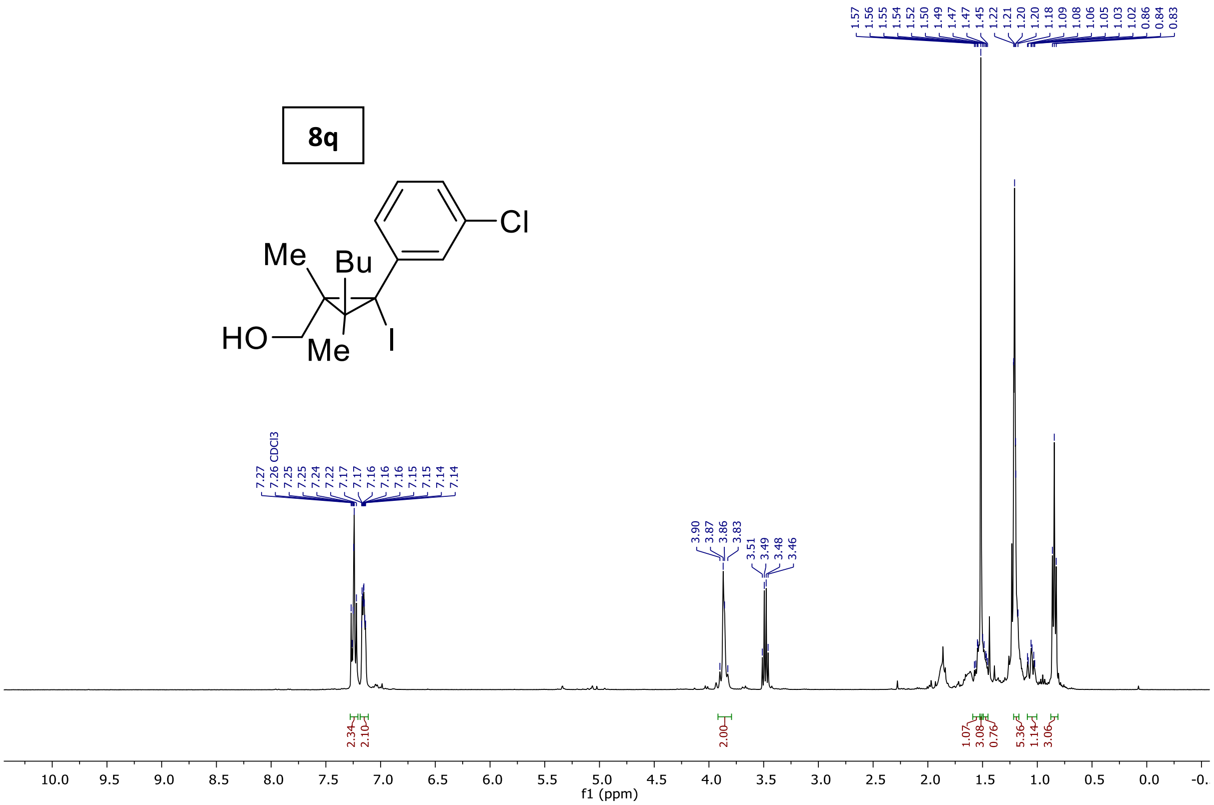
8q
Cl
Me
Bu
HO
Me
I
1.57 1.56 1.55 1.54 1.52 1.50 1.49 1.47 1.47 1.45 1.22 1.21 1.20 1.20 1.18 1.09 1.08 1.06 1.05 1.03 1.02 0.86 0.84 0.83
7.27 7.26 CDCl3 7.25 7.25 7.24 7.22 7.17 7.17 7.16 7.16 7.16 7.15 7.15 7.14 7.14
3.90 3.87 3.86 3.83
3.51 3.49 3.48 3.46
2.34 2.10 2.00 1.07 3.08 0.76 5.36 1.14 3.06
f1 (ppm)

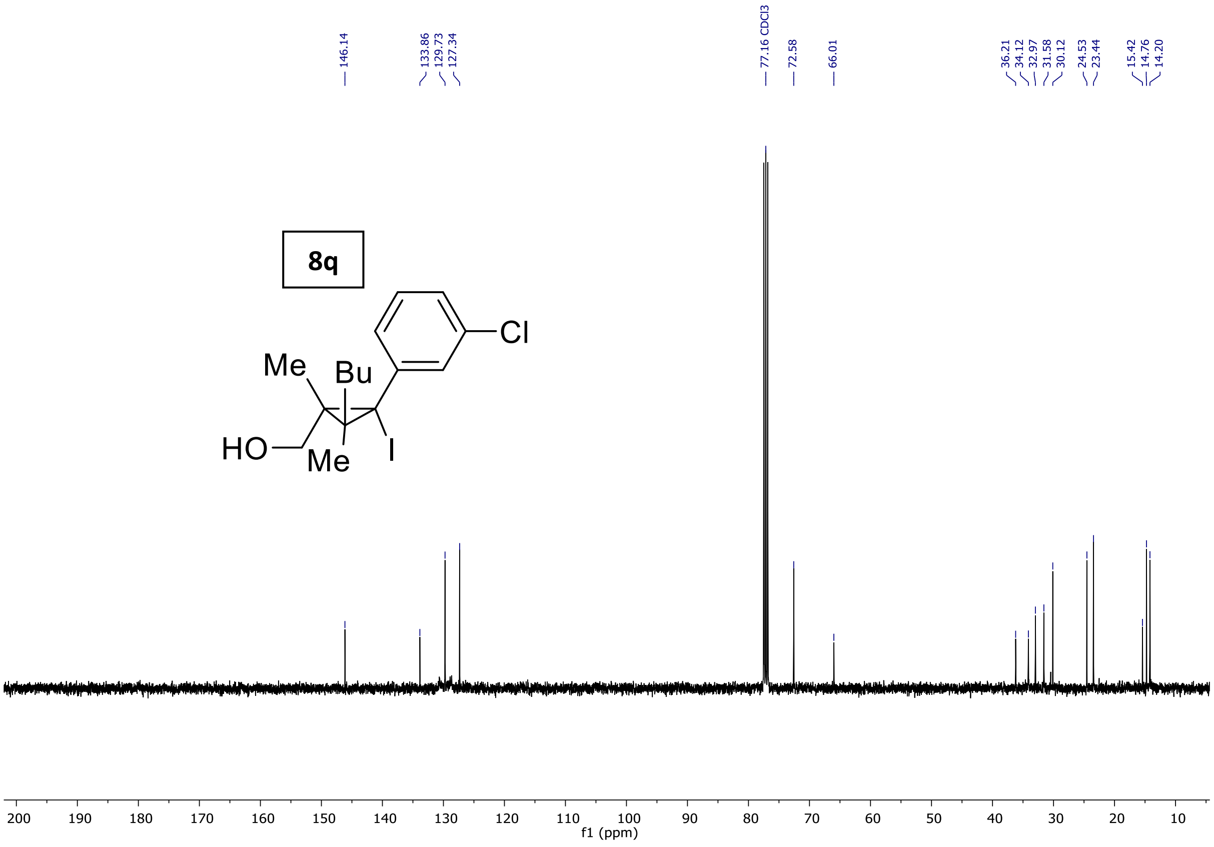
146.14
133.86 129.73 127.34
77.16 CDCl3
72.58
66.01
36.21 34.12 32.97 31.58 30.12 24.53 23.44
15.42 14.76 14.20
8q
Cl
Me
Bu
HO
Me
I
f1 (ppm)

8r

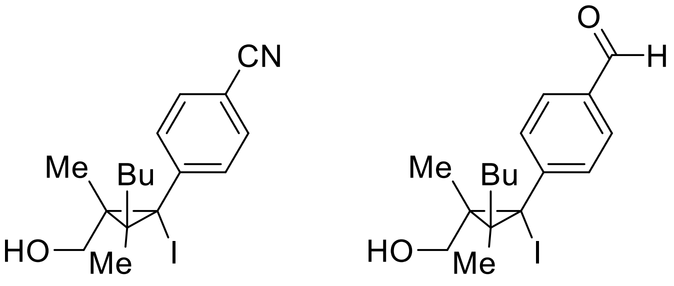

CN
Me
Bu
HO
Me
I
O
H
Me
Bu
HO
Me
I

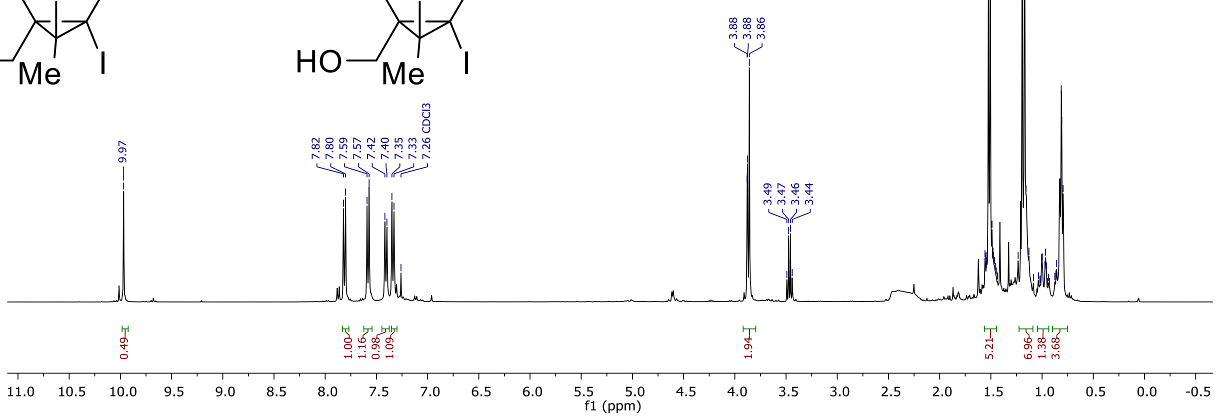

9.97
7.82 7.80 7.59 7.57 7.42 7.40 7.35 7.33 7.26 CDCl3
3.88 3.88 3.86
3.49 3.47 3.46 3.44
1.56 1.55 1.54 1.54 1.52 1.51 1.49 1.48 1.47 1.46 1.46 1.45 1.44 1.23 1.19 1.17 1.16 1.13 1.08 1.03 1.02 1.02 1.00 1.00 0.97 0.97 0.96 0.95 0.94 0.93 0.88 0.87 0.86 0.83 0.83 0.81 0.81 0.79 0.79
0.49
1.00
1.16
0.98
1.09
1.94
5.21
6.96
1.38
3.68
f1 (ppm)

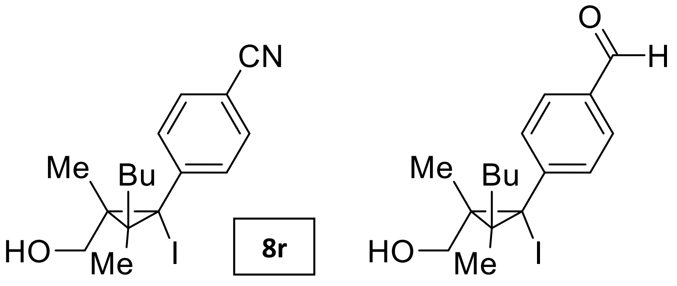

CN
Me
Bu
HO
Me
I
O
H
Me
Bu
HO
Me
I

8r

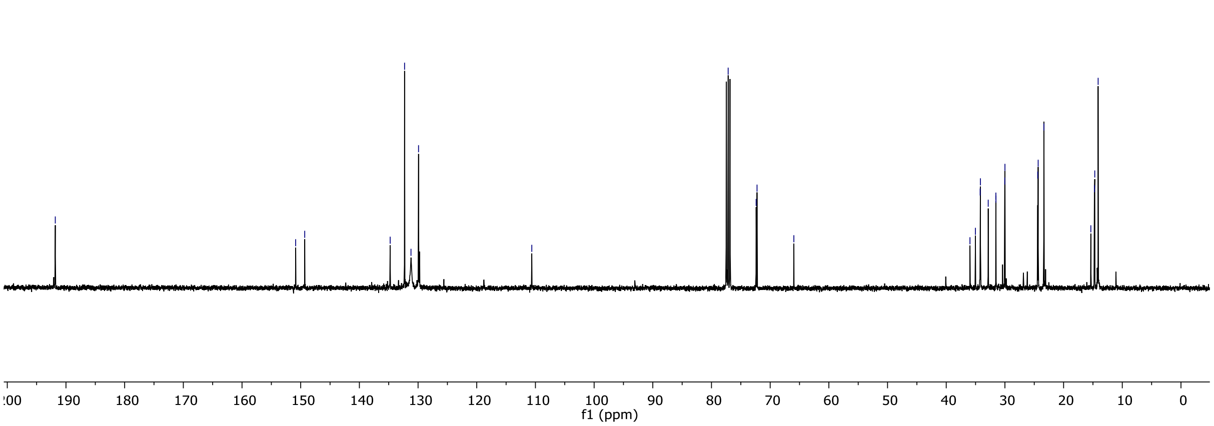

191.82
150.86
149.31
134.75
132.30
131.21
129.92
110.62
77.16 CDCl3
72.39
72.24
65.96
35.95 35.03 34.22 34.18 32.84 31.54 31.53 30.02 29.99 24.41 24.33 23.36 15.36 14.75 14.69 14.11
f1 (ppm)

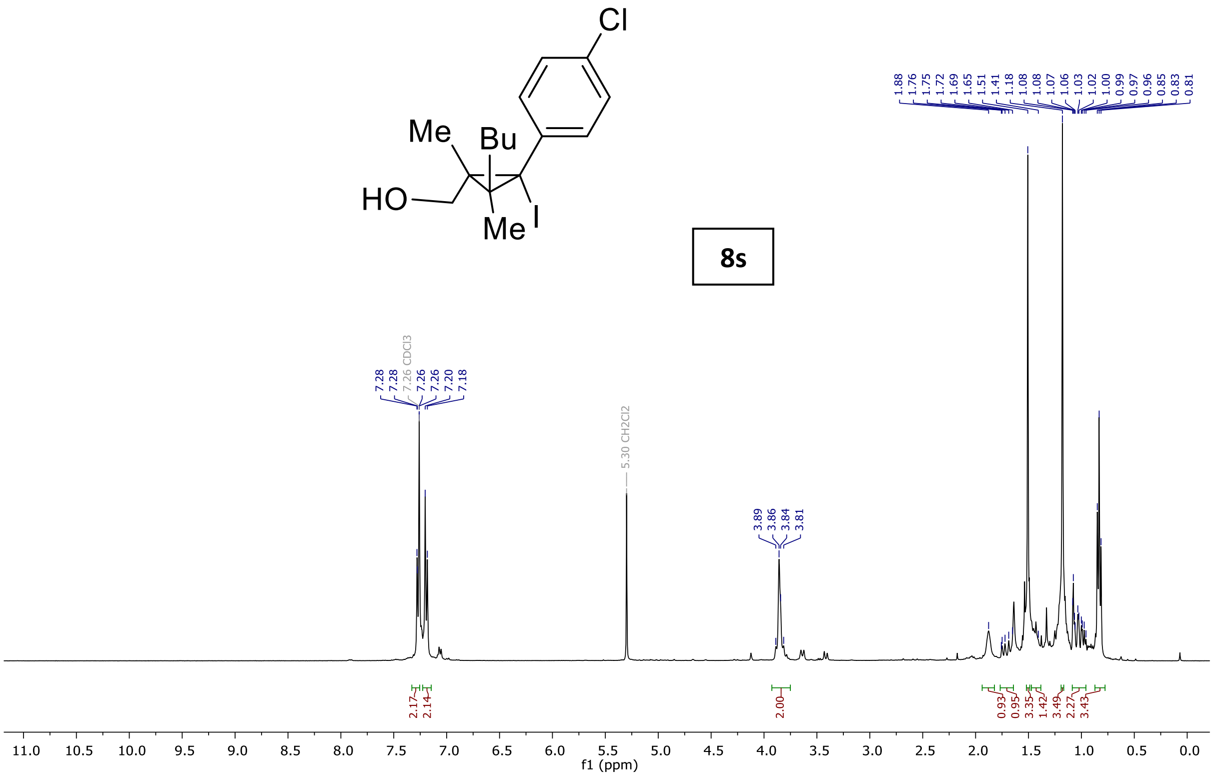
Cl
Me
Bu
HO
Me
I
8s
1.88 1.76 1.75 1.72 1.69 1.65 1.51 1.41 1.18 1.08 1.08 1.07 1.06 1.03 1.02 1.00 0.99 0.97 0.96 0.85 0.83 0.81
7.28 7.28 7.26 CDCl3 7.26 7.26 7.20 7.18
5.30 CH2Cl2
3.89 3.86 3.84 3.81
2.17 2.14
2.00
0.93 0.95 3.35 1.42 3.49 2.27 3.43
f1 (ppm)

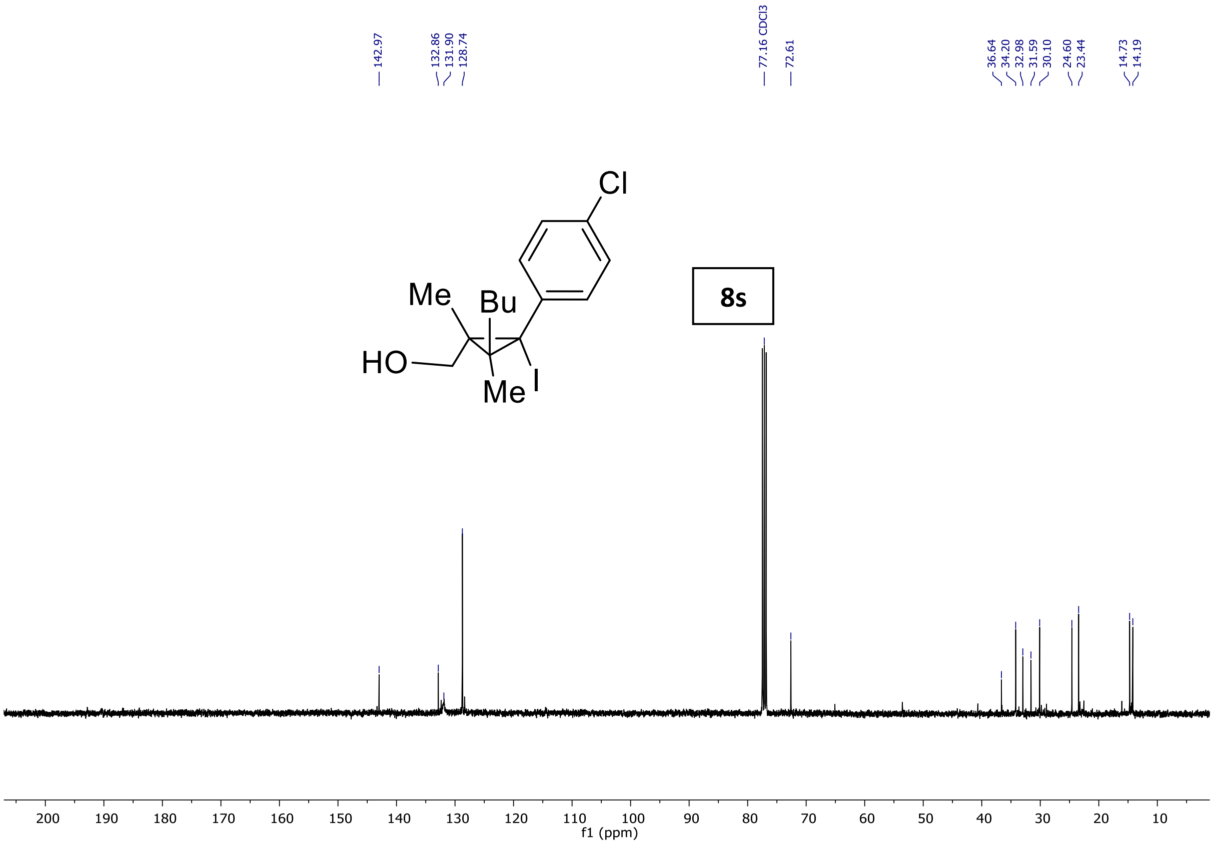
142.97
132.86 131.90 128.74
77.16 CDCl3
72.61
36.64 34.20 32.98 31.59 30.10 24.60 23.44
14.73 14.19
Cl
Me
Bu
HO
Me
I
8s
f1 (ppm)

8t

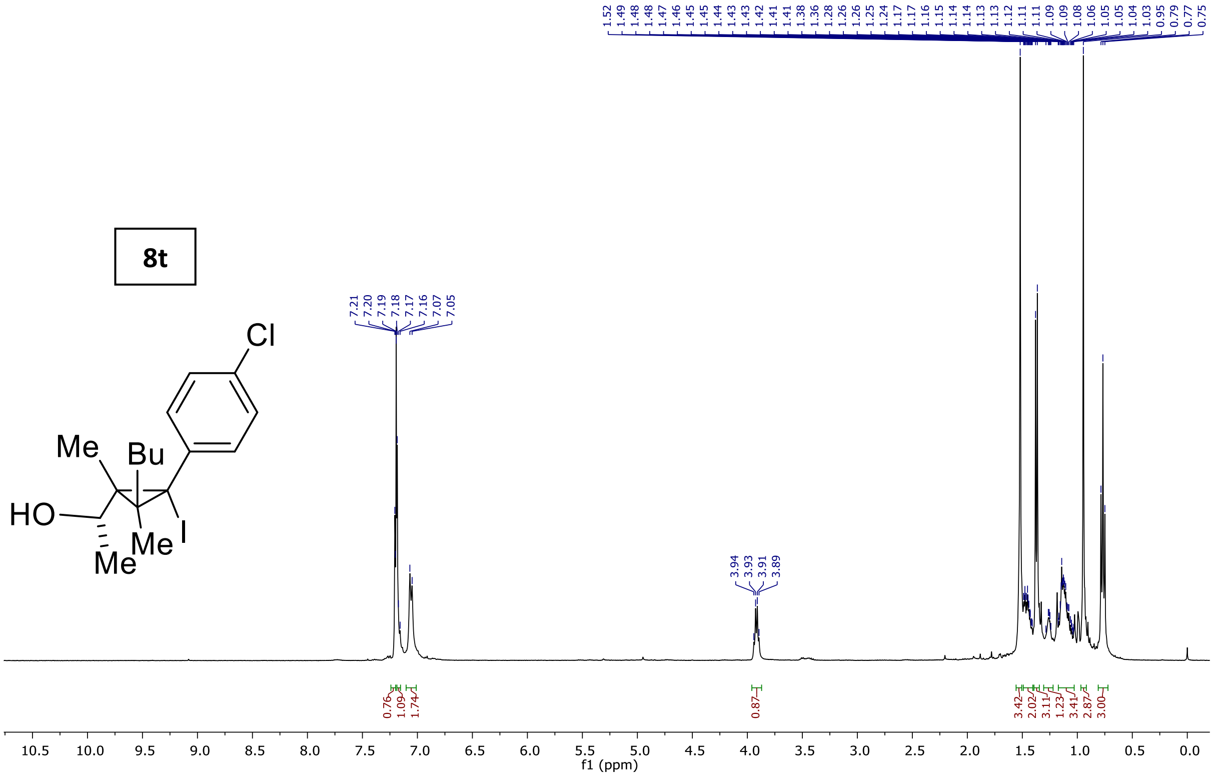
Cl
Me
Bu
HO
Me
I
Me
1.52 1.49 1.48 1.47 1.46 1.45 1.45 1.44 1.43 1.43 1.42 1.41 1.41 1.38 1.36 1.28 1.26 1.26 1.25 1.24 1.17 1.17 1.16 1.15 1.14 1.14 1.13 1.13 1.12 1.11 1.11 1.09 1.09 1.08 1.06 1.05 1.05 1.04 1.03 0.95 0.79 0.77 0.75
7.21 7.20 7.19 7.18 7.17 7.16 7.07 7.05
3.94 3.93 3.91 3.89
0.76 1.09 1.74
0.87
3.42 2.02 3.11 1.23 3.41 2.87 3.00
10.5 10.0 9.5 9.0 8.5 8.0 7.5 7.0 6.5 6.0 5.5 5.0 4.5 4.0 3.5 3.0 2.5 2.0 1.5 1.0 0.5 0.0
f1 (ppm)

8t

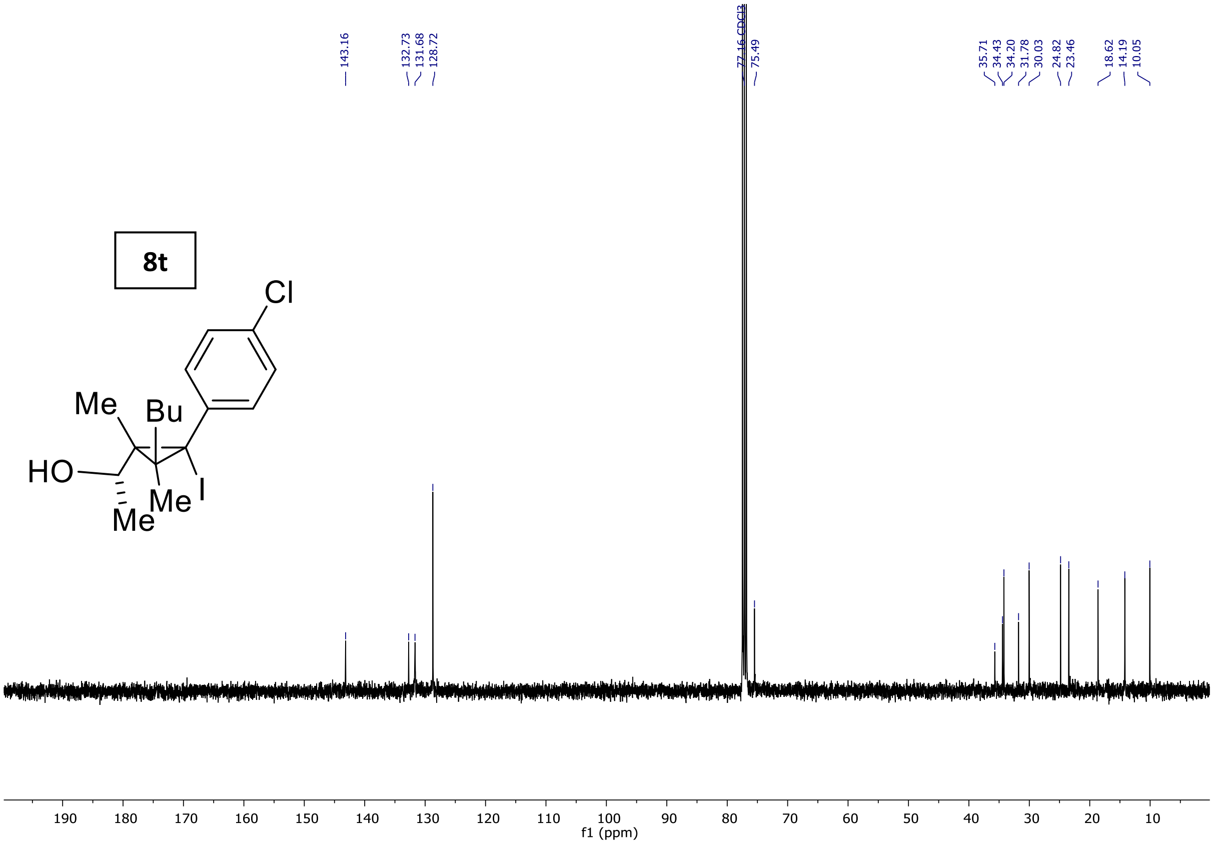
Cl
Me
Bu
HO
Me
I
Me
143.16
132.73 131.68 128.72
77.16 CDCl3
75.49
35.71 34.43 34.20 31.78 30.03 24.82 23.46
18.62 14.19 10.05
190 180 170 160 150 140 130 120 110 100 90 80 70 60 50 40 30 20 10
f1 (ppm)

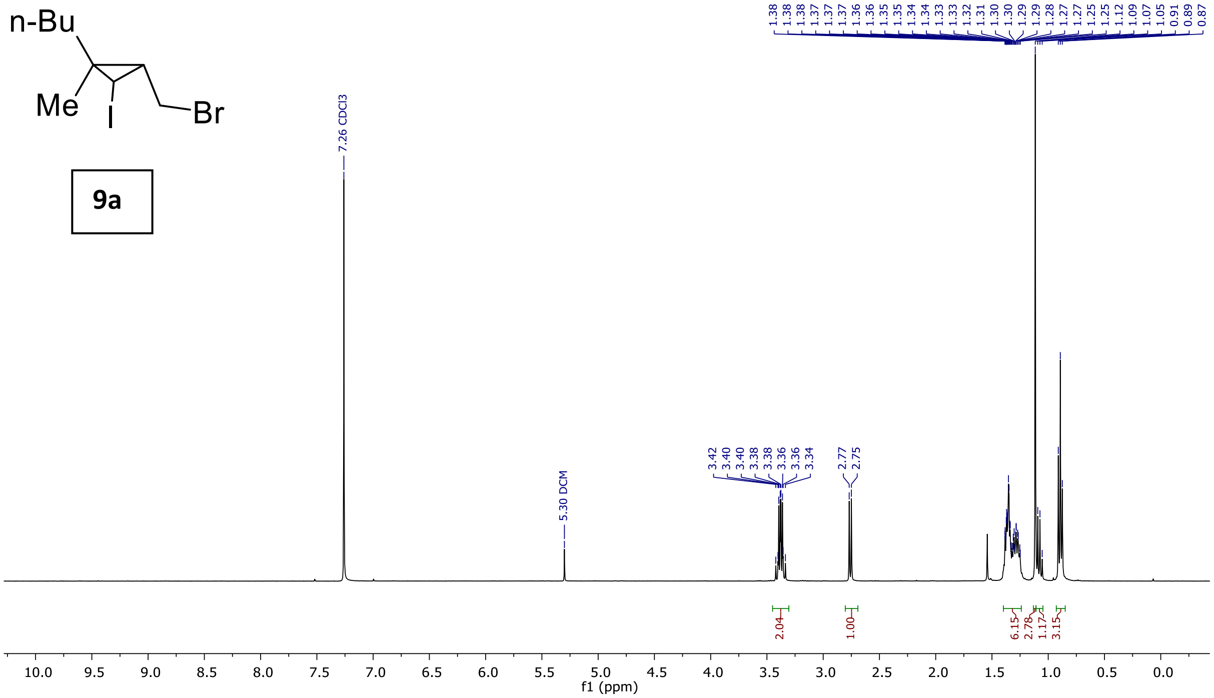
n-Bu
Me
I
Br
9a
1.38 1.38 1.38 1.37 1.37 1.37 1.36 1.36 1.35 1.35 1.34 1.34 1.33 1.33 1.32 1.31 1.30 1.30 1.29 1.29 1.28 1.27 1.27 1.25 1.25 1.12 1.09 1.07 1.05 0.91 0.89 0.87
7.26 CDCl3
5.30 DCM
3.42 3.40 3.40 3.38 3.38 3.36 3.36 3.34
2.77 2.75
2.04
1.00
6.15 2.78 1.17 3.15
10.0 9.5 9.0 8.5 8.0 7.5 7.0 6.5 6.0 5.5 5.0 4.5 4.0 3.5 3.0 2.5 2.0 1.5 1.0 0.5 0.0
f1 (ppm)

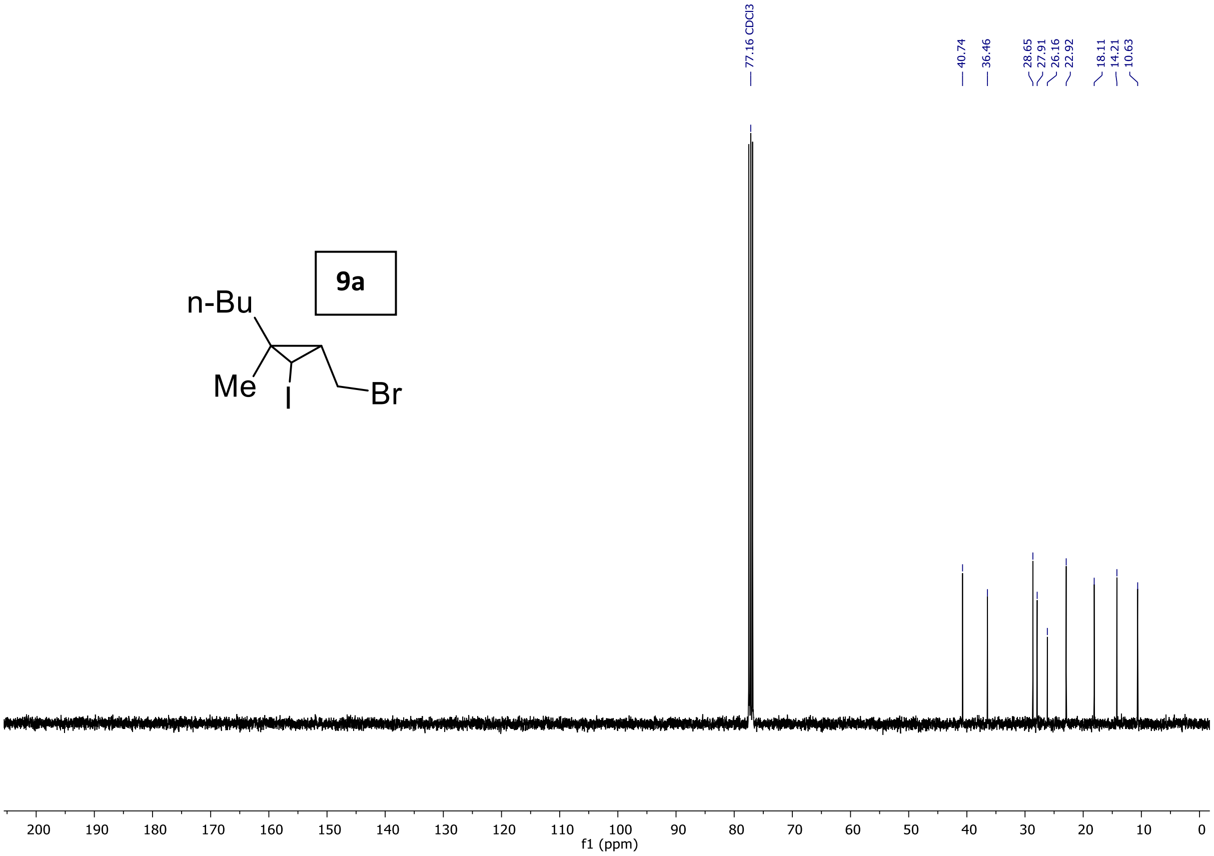
77.16 CDCl3
40.74
36.46
28.65 27.91 26.16 22.92
18.11 14.21 10.63
9a
n-Bu
Me
I
Br
200 190 180 170 160 150 140 130 120 110 100 90 80 70 60 50 40 30 20 10 0
f1 (ppm)

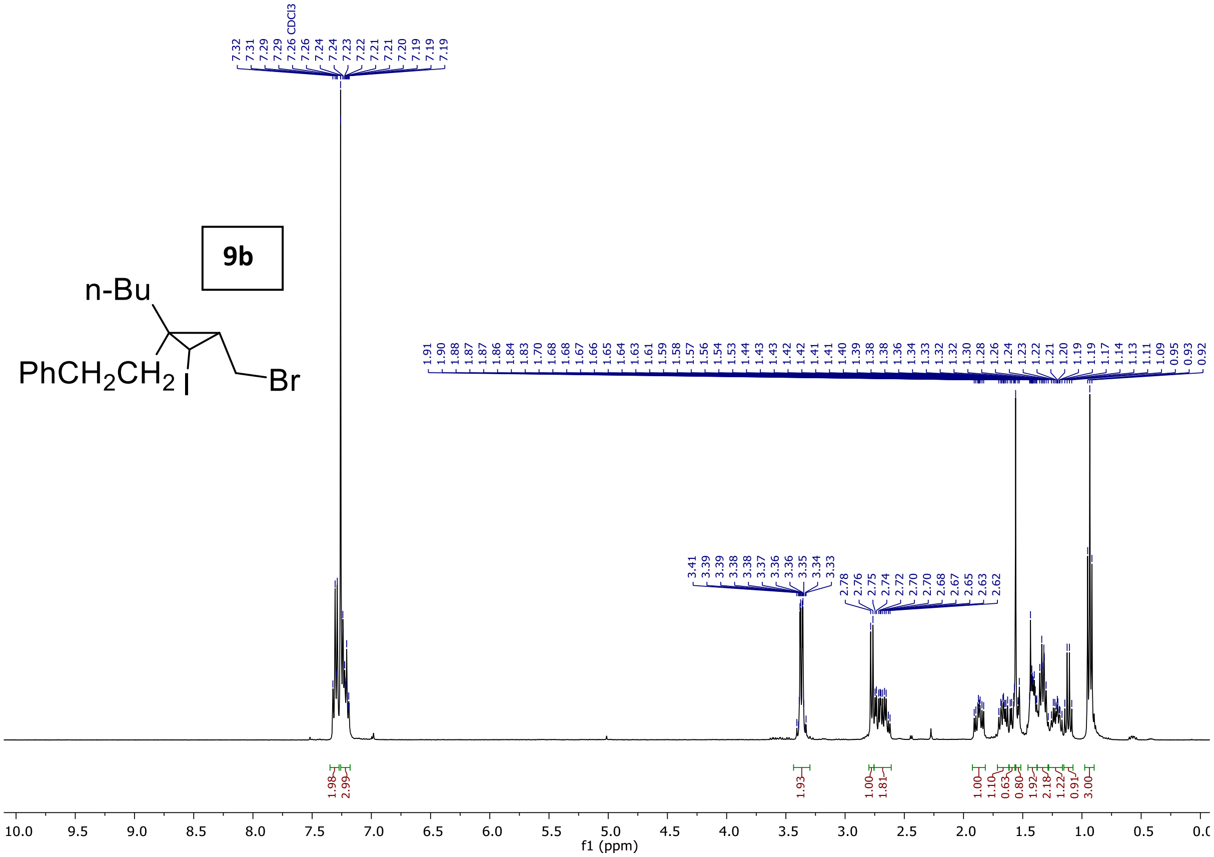
9b
n-Bu
PhCH2CH2
I
Br
f1 (ppm)

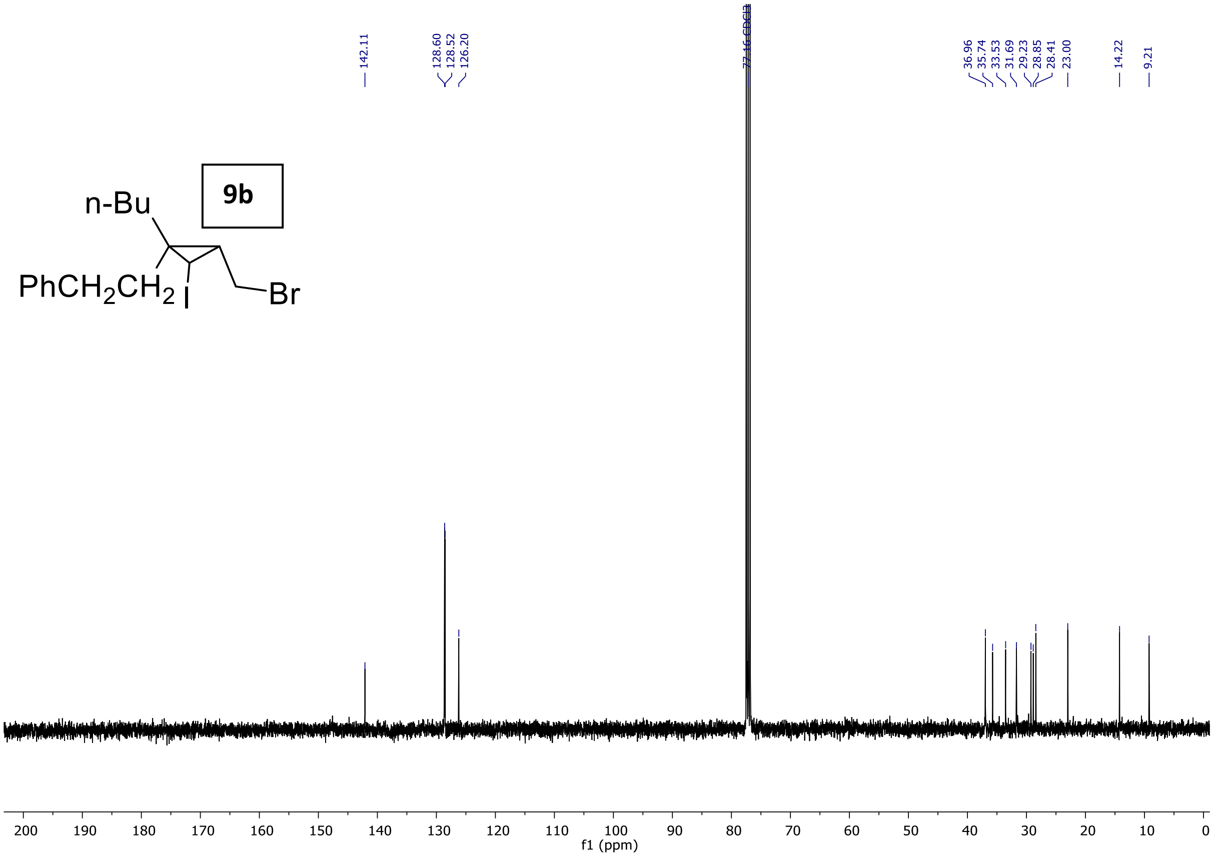
9b
n-Bu
PhCH2CH2
I
Br
142.11
128.60
128.52
126.20
77.16 CDCl3
36.96
35.74
33.53
31.69
29.23
28.85
28.41
23.00
14.22
9.21
f1 (ppm)

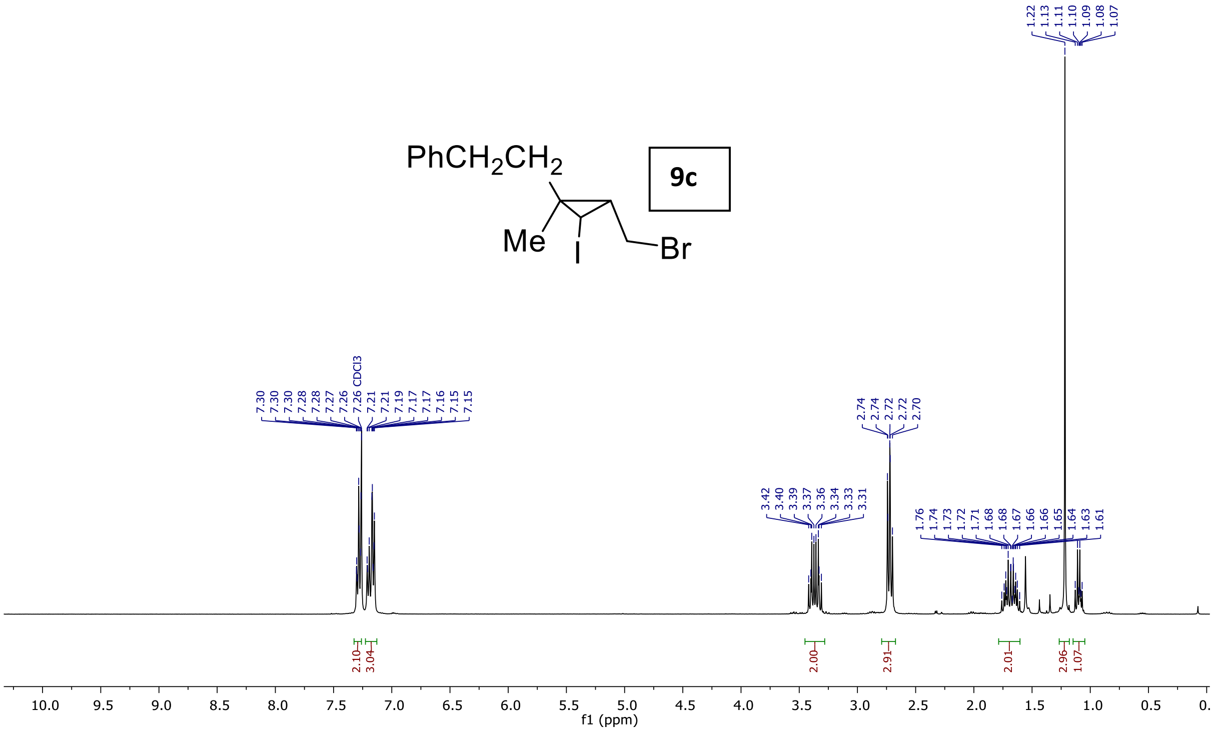
PhCH2CH2
Me
I
Br
9c
f1 (ppm)

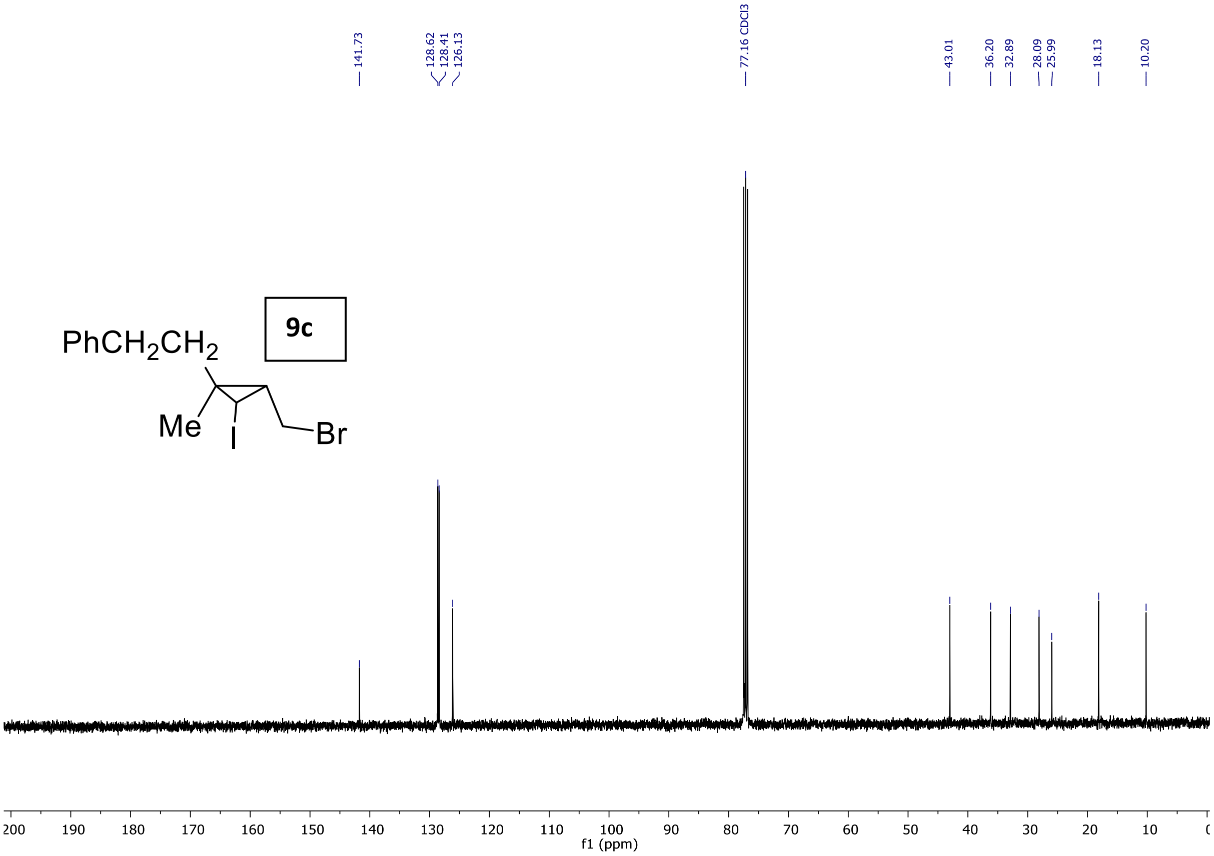
PhCH2CH2
Me
I
Br
9c
f1 (ppm)

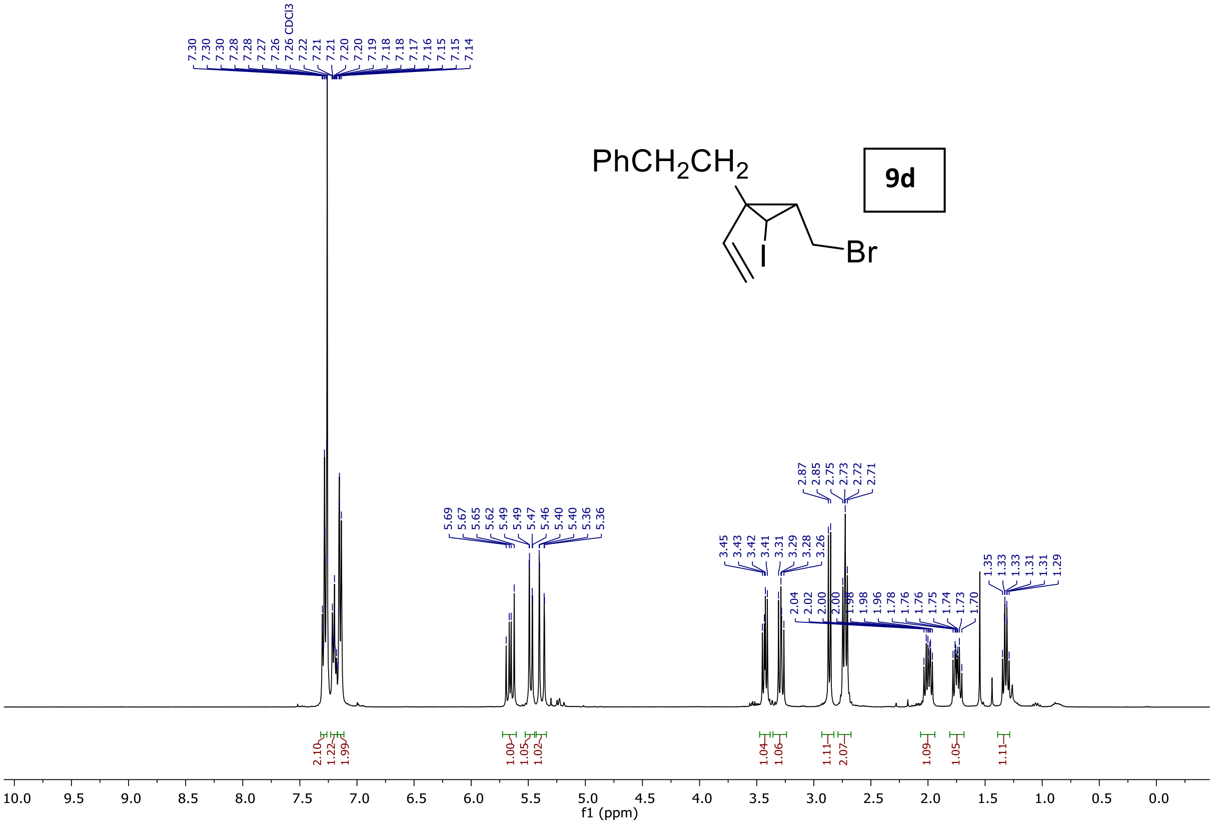
PhCH2CH2
9d
I
Br
7.30 7.30 7.30 7.28 7.28 7.27 7.26 CDCl3 7.22 7.21 7.21 7.20 7.20 7.19 7.18 7.18 7.17 7.16 7.15 7.15 7.14
5.69 5.67 5.65 5.62 5.49 5.49 5.47 5.46 5.40 5.40 5.36 5.36
3.45 3.43 3.42 3.41 3.31 3.29 3.28 3.26
2.87 2.85 2.75 2.73 2.72 2.71
2.04 2.02 2.00 2.00 1.98 1.98 1.96 1.78 1.76 1.76 1.75 1.74 1.73 1.70
1.35 1.33 1.33 1.31 1.31 1.29
2.10 1.22 1.99 1.00 1.05 1.02 1.04 1.06 1.11 2.07 1.09 1.05 1.11
f1 (ppm)

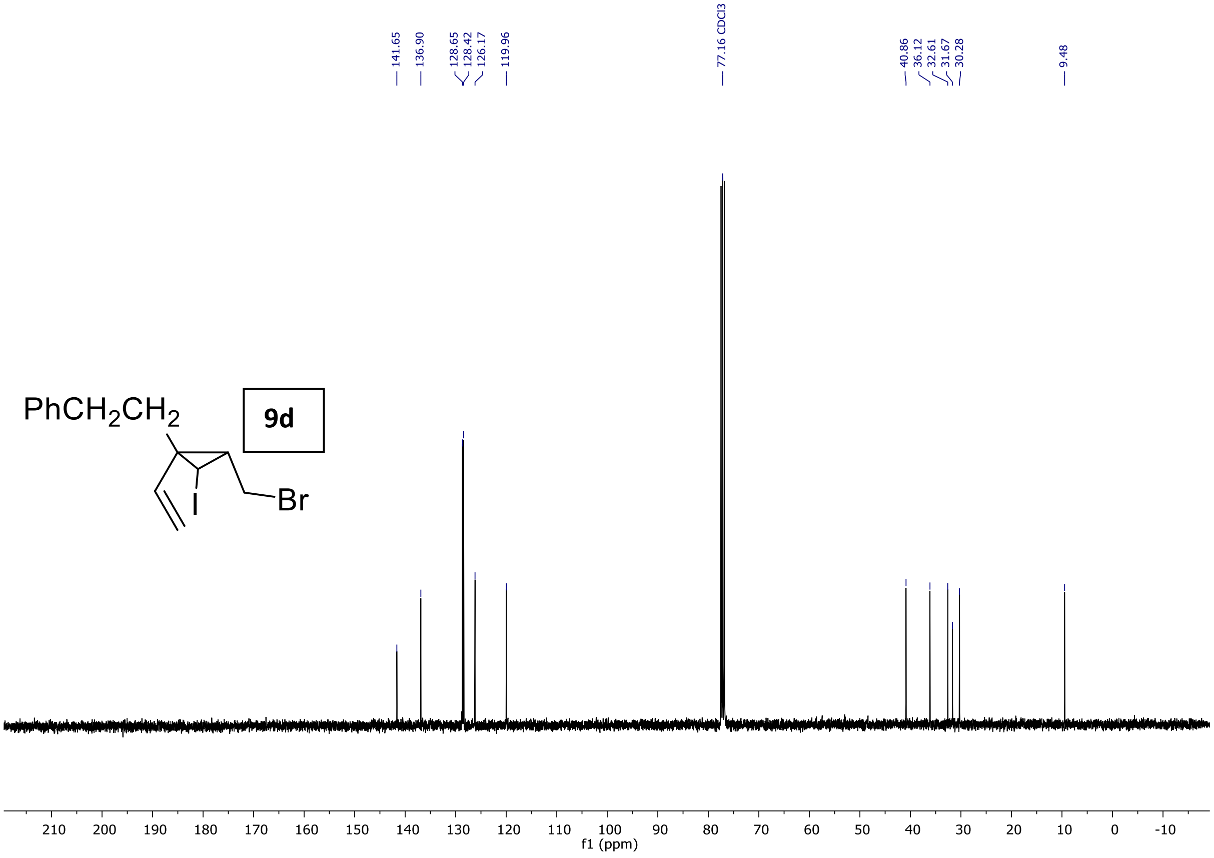
141.65 136.90 128.65 128.42 126.17 119.96 77.16 CDCl3 40.86 36.12 32.61 31.67 30.28 9.48
PhCH2CH2
9d
I
Br
f1 (ppm)

9e

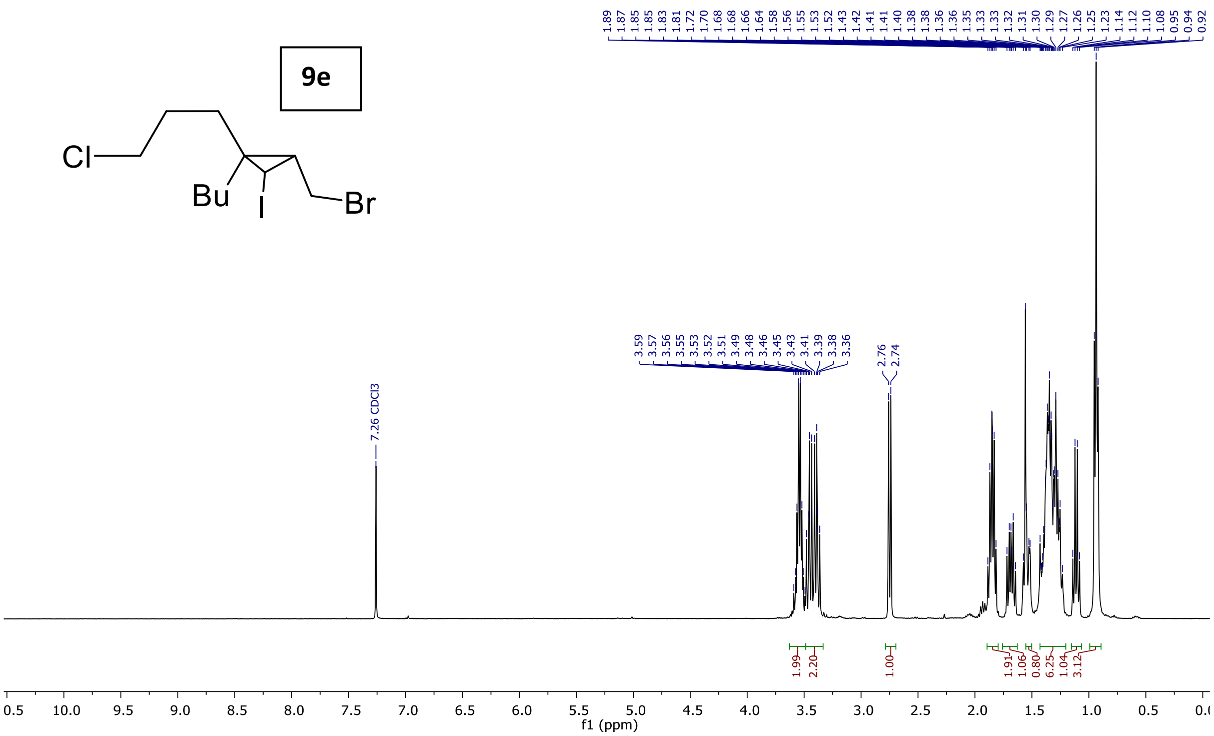

9e

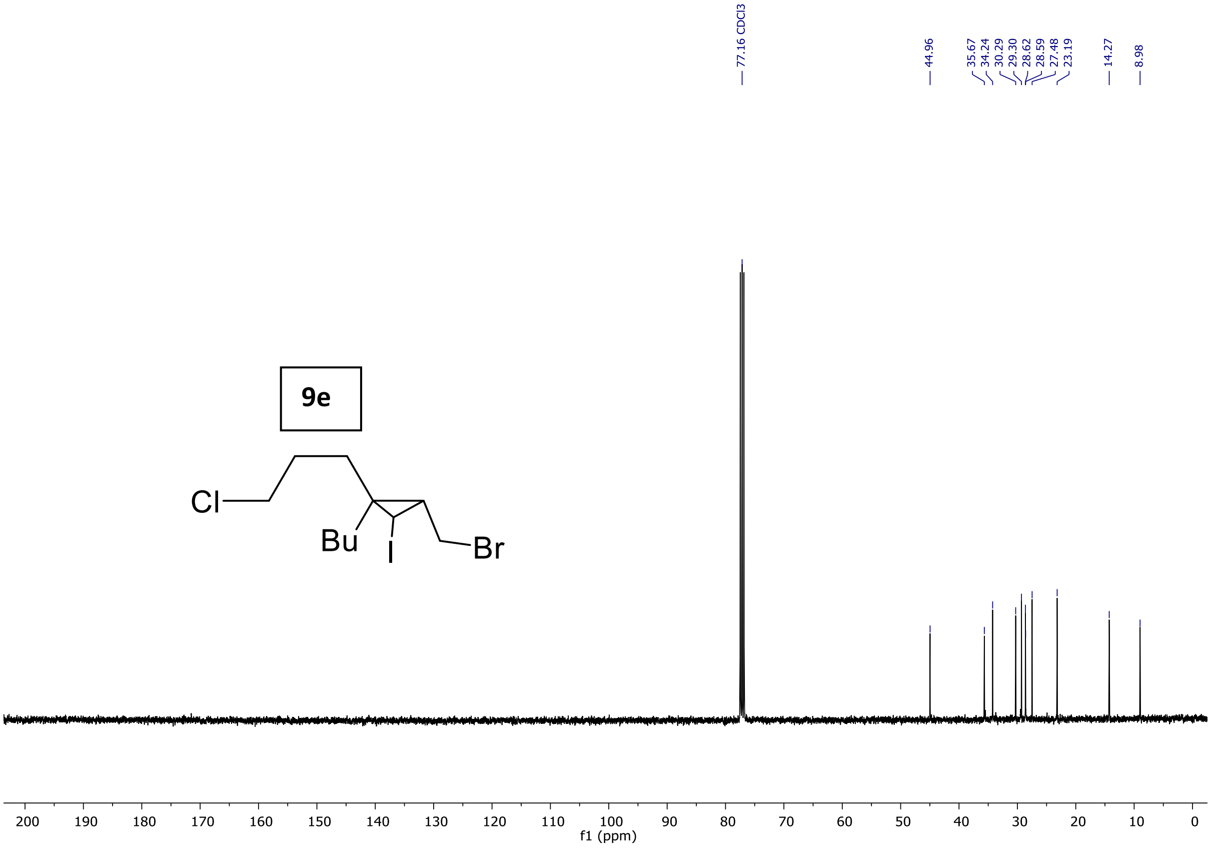

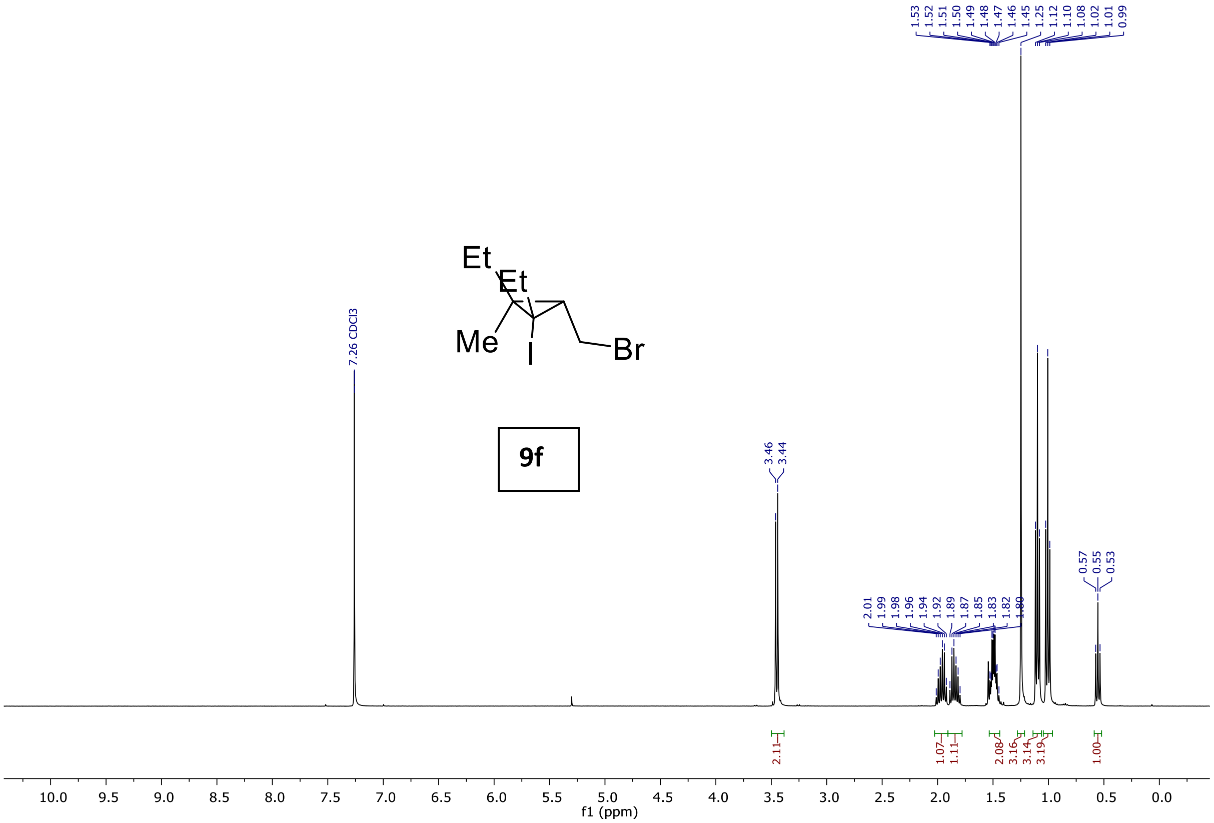

1.53
1.52
1.51
1.50
1.49
1.48
1.47
1.46
1.45
1.25
1.12
1.10
1.08
1.02
1.01
0.99
7.26 CDCl3
Et
Et
Me
I
Br
9f
3.46
3.44
2.01
1.99
1.98
1.96
1.94
1.92
1.89
1.87
1.85
1.83
1.82
1.80
0.57
0.55
0.53
2.11
1.07
1.11
2.08
3.16
3.14
3.19
1.00
10.0 9.5 9.0 8.5 8.0 7.5 7.0 6.5 6.0 5.5 5.0 4.5 4.0 3.5 3.0 2.5 2.0 1.5 1.0 0.5 0.0
f1 (ppm)

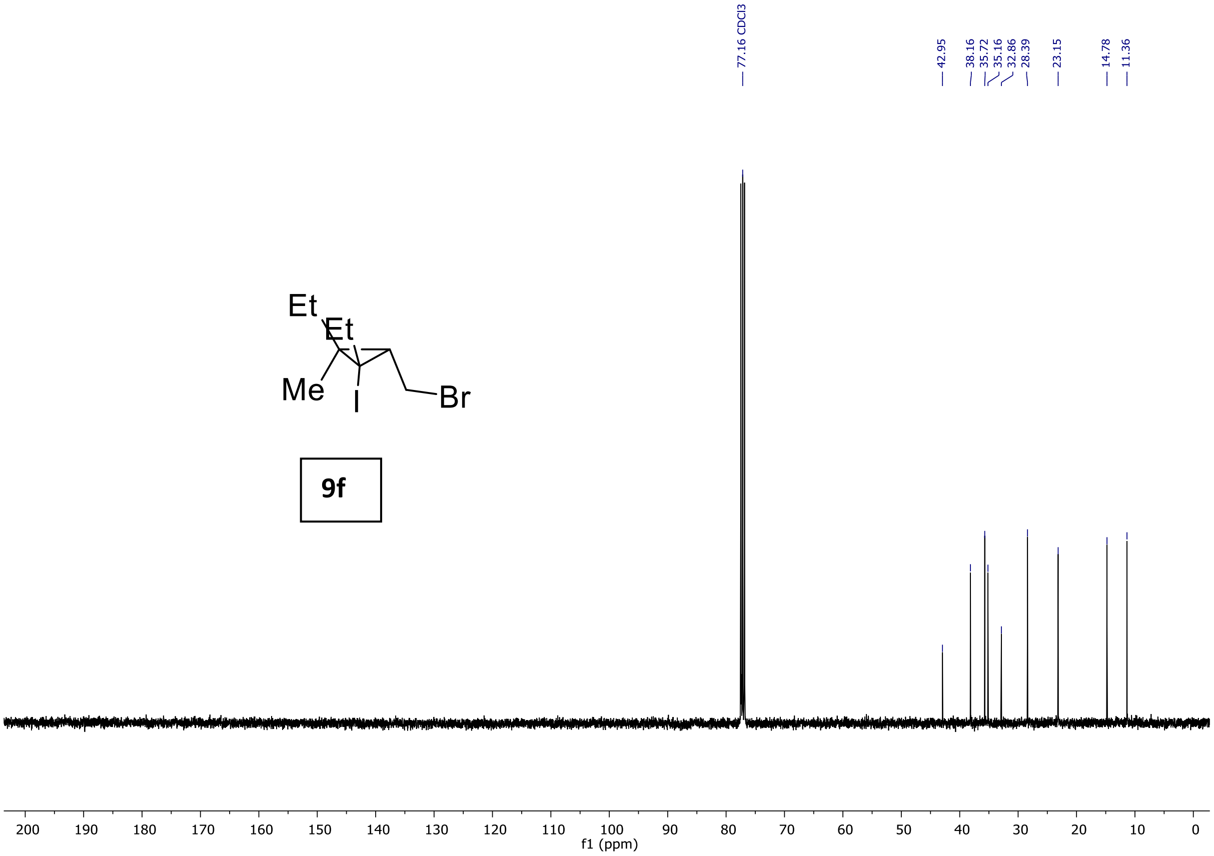

77.16 CDCl3
42.95
38.16
35.72
35.16
32.86
28.39
23.15
14.78
11.36
Et
Et
Me
I
Br
9f
200 190 180 170 160 150 140 130 120 110 100 90 80 70 60 50 40 30 20 10 0
f1 (ppm)

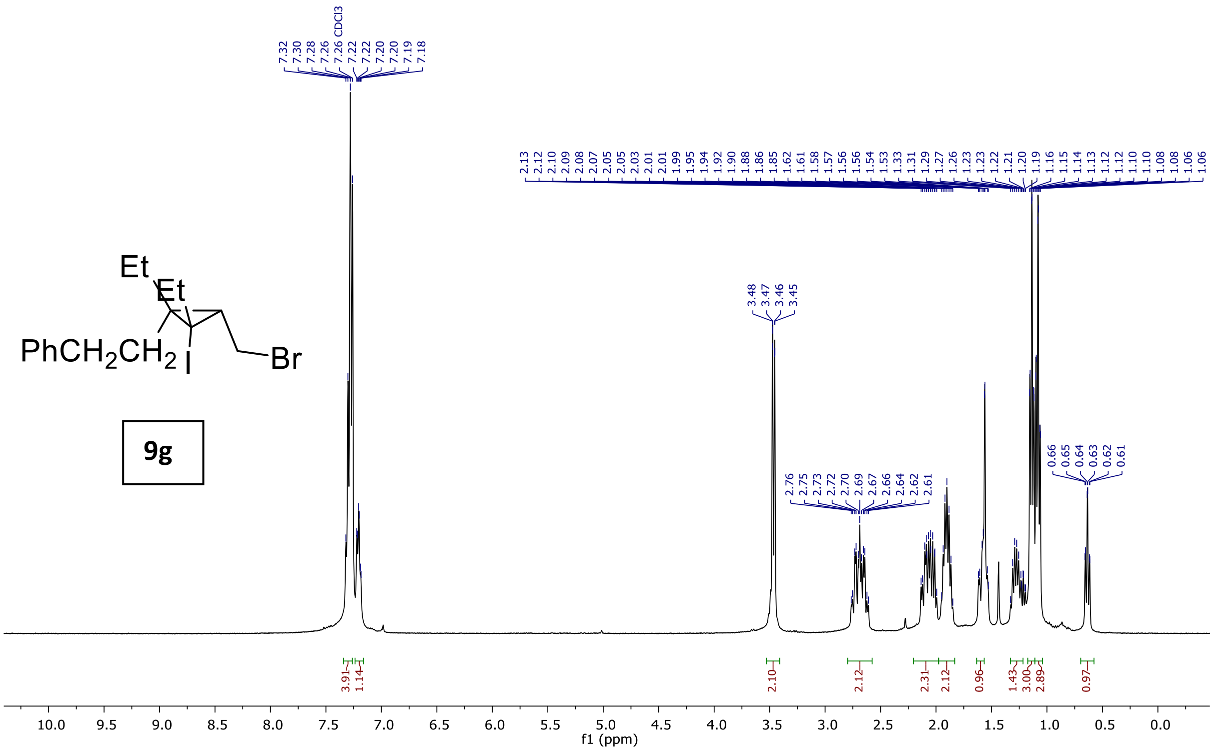

7.32
7.30
7.28
7.26
7.26 CDCl3
7.22
7.22
7.20
7.20
7.19
7.18
2.13
2.12
2.10
2.09
2.08
2.07
2.05
2.05
2.03
2.01
2.01
1.99
1.95
1.94
1.92
1.90
1.88
1.86
1.85
1.62
1.61
1.58
1.57
1.56
1.56
1.54
1.53
1.53
1.33
1.31
1.29
1.27
1.26
1.23
1.23
1.22
1.21
1.20
1.19
1.16
1.15
1.14
1.13
1.12
1.12
1.10
1.10
1.08
1.06
3.48
3.47
3.46
3.45
2.76
2.75
2.73
2.72
2.70
2.69
2.67
2.66
2.64
2.62
2.61
0.66
0.65
0.64
0.63
0.62
0.61
Et
Et
PhCH2CH2
I
Br
9g
3.91
1.14
2.10
2.12
2.31
2.12
0.96
1.43
3.00
2.89
0.97
10.0
9.5
9.0
8.5
8.0
7.5
7.0
6.5
6.0
5.5
5.0
4.5
4.0
3.5
3.0
2.5
2.0
1.5
1.0
0.5
0.0
f1 (ppm)

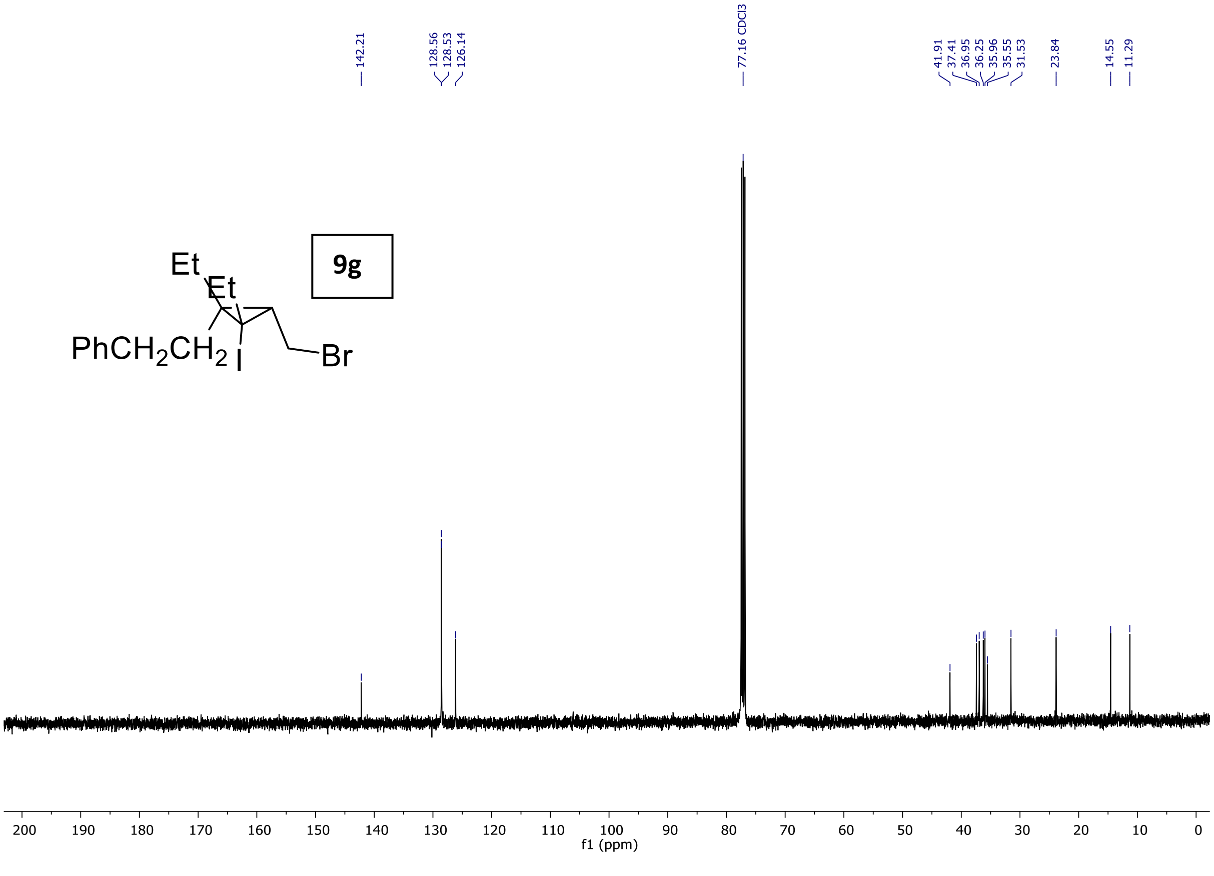

142.21
128.56
128.53
126.14
77.16 CDCl3
41.91
37.41
36.95
36.25
35.96
35.55
31.53
23.84
14.55
11.29
Et
Et
PhCH2CH2
I
Br
9g
200
190
180
170
160
150
140
130
120
110
100
90
80
70
60
50
40
30
20
10
0
f1 (ppm)

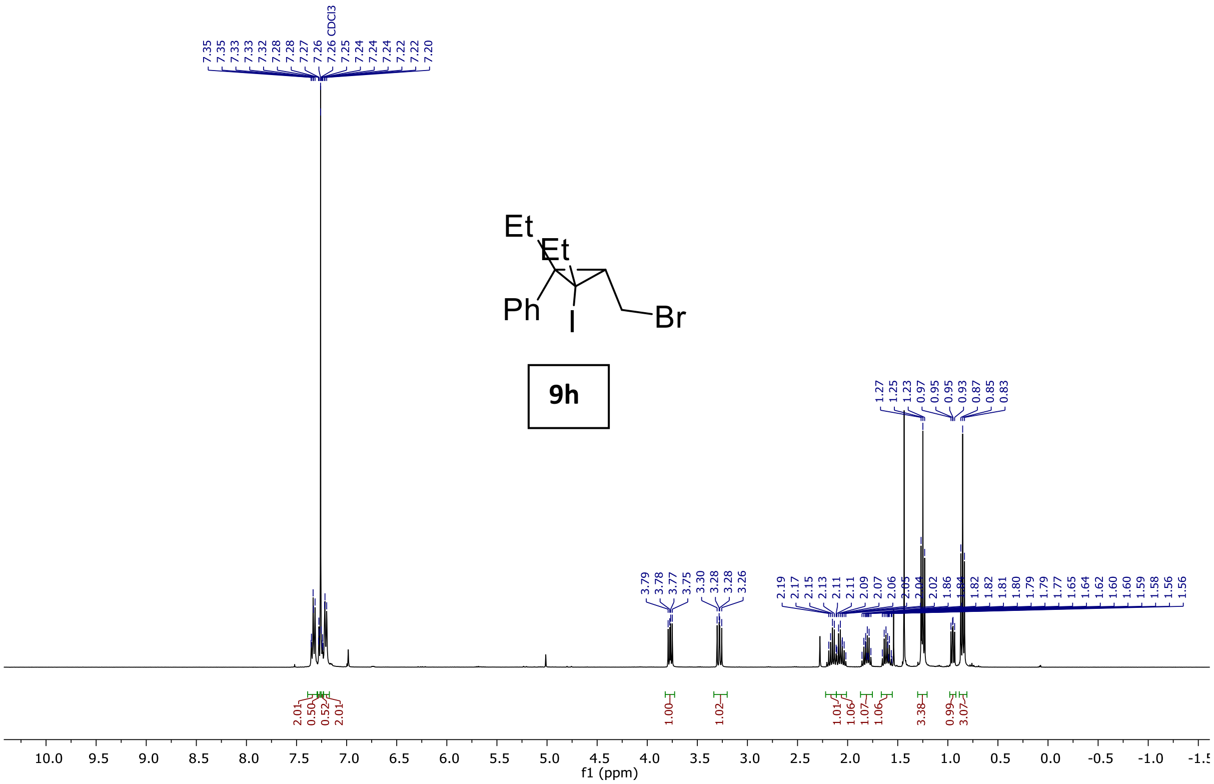
9h
7.35
7.35
7.33
7.33
7.32
7.28
7.28
7.27
7.26
7.26 CDCl3
7.25
7.24
7.24
7.24
7.22
7.20
3.79
3.78
3.77
3.75
3.30
3.28
3.28
3.26
1.27
1.25
1.23
0.97
0.95
0.95
0.93
0.87
0.85
0.83
f1 (ppm)

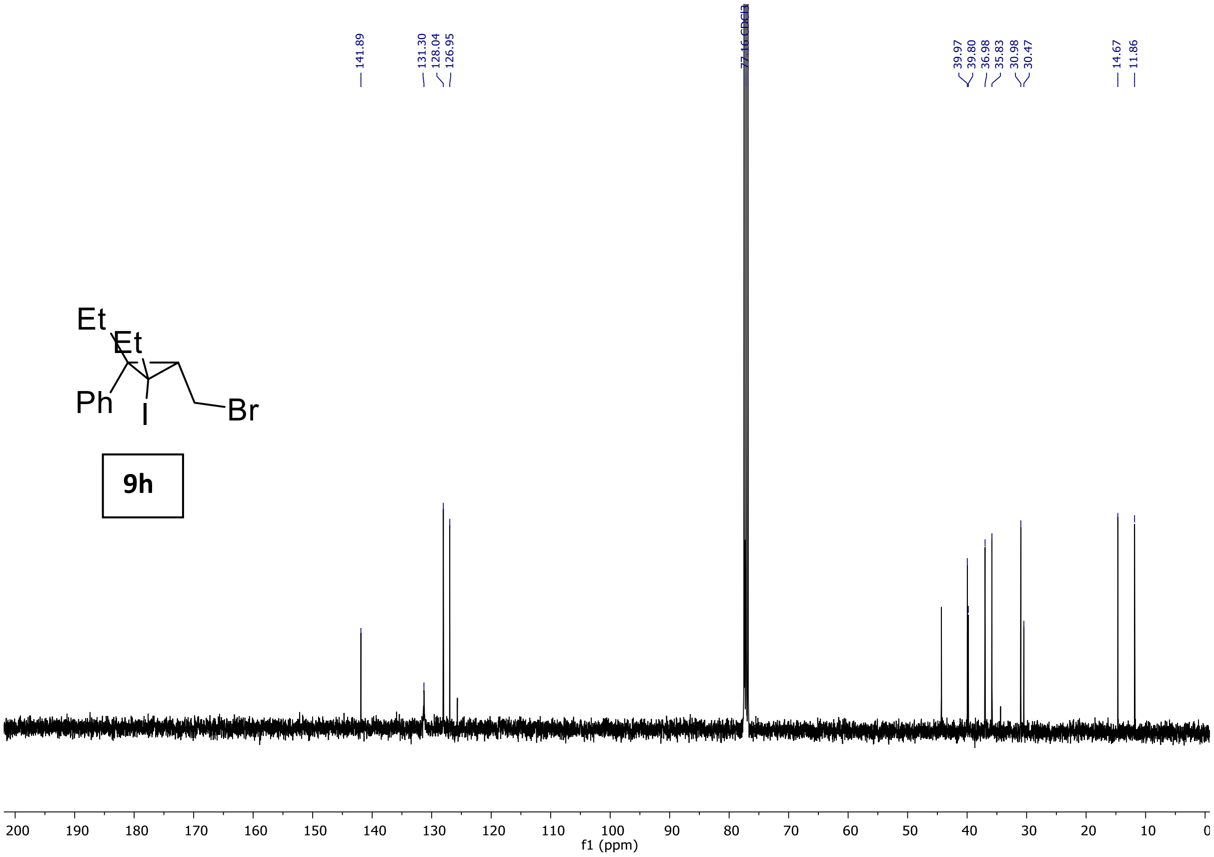
141.89
131.30
128.04
126.95
77.16 CDCl3
39.97
39.80
36.98
35.83
30.98
30.47
14.67
11.86
9h
f1 (ppm)

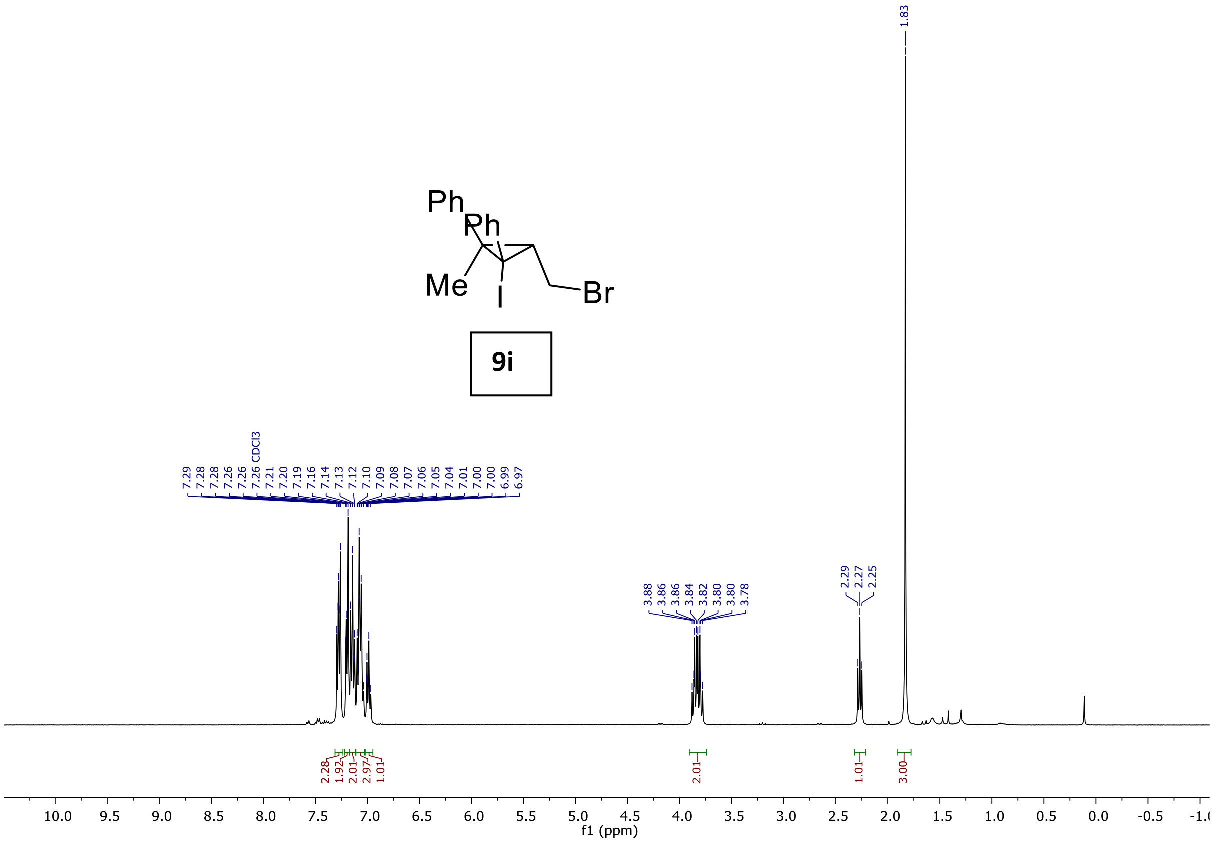
9i
f1 (ppm)

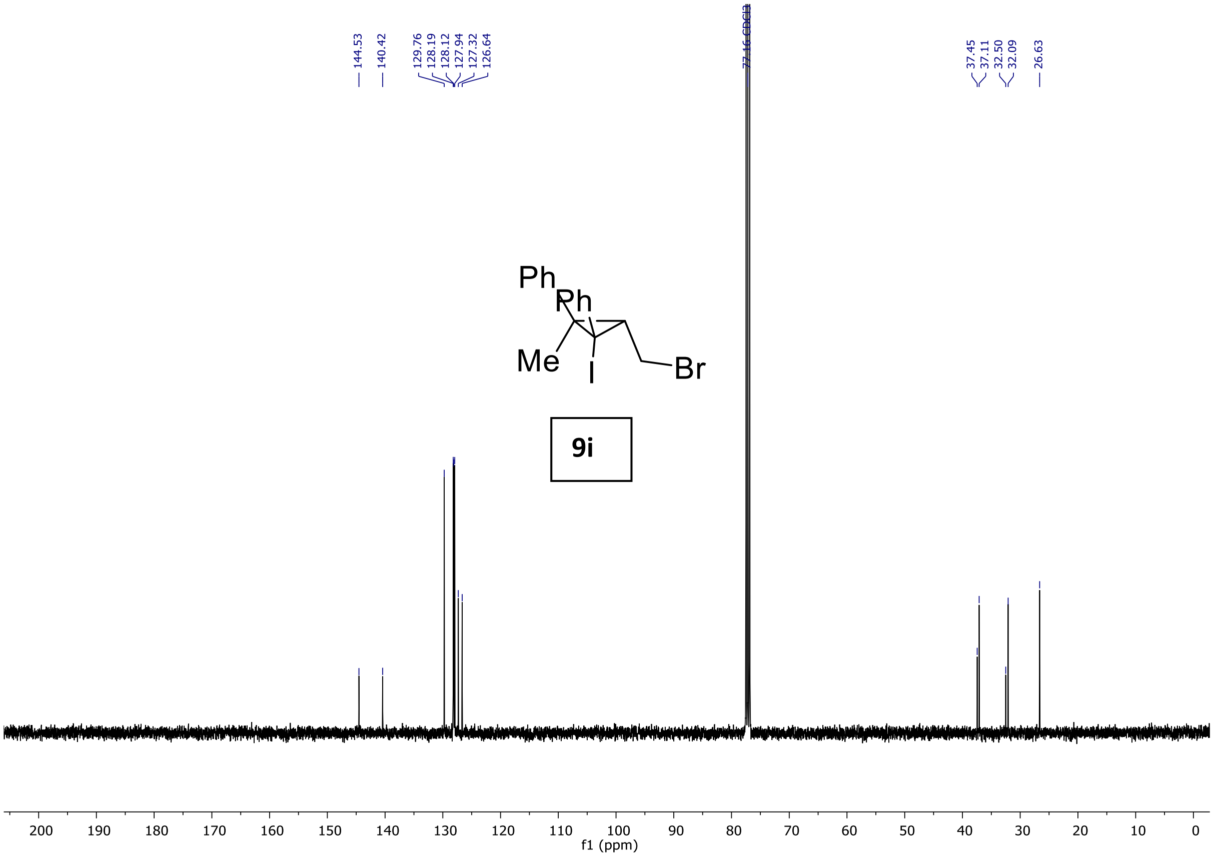
9i
f1 (ppm)

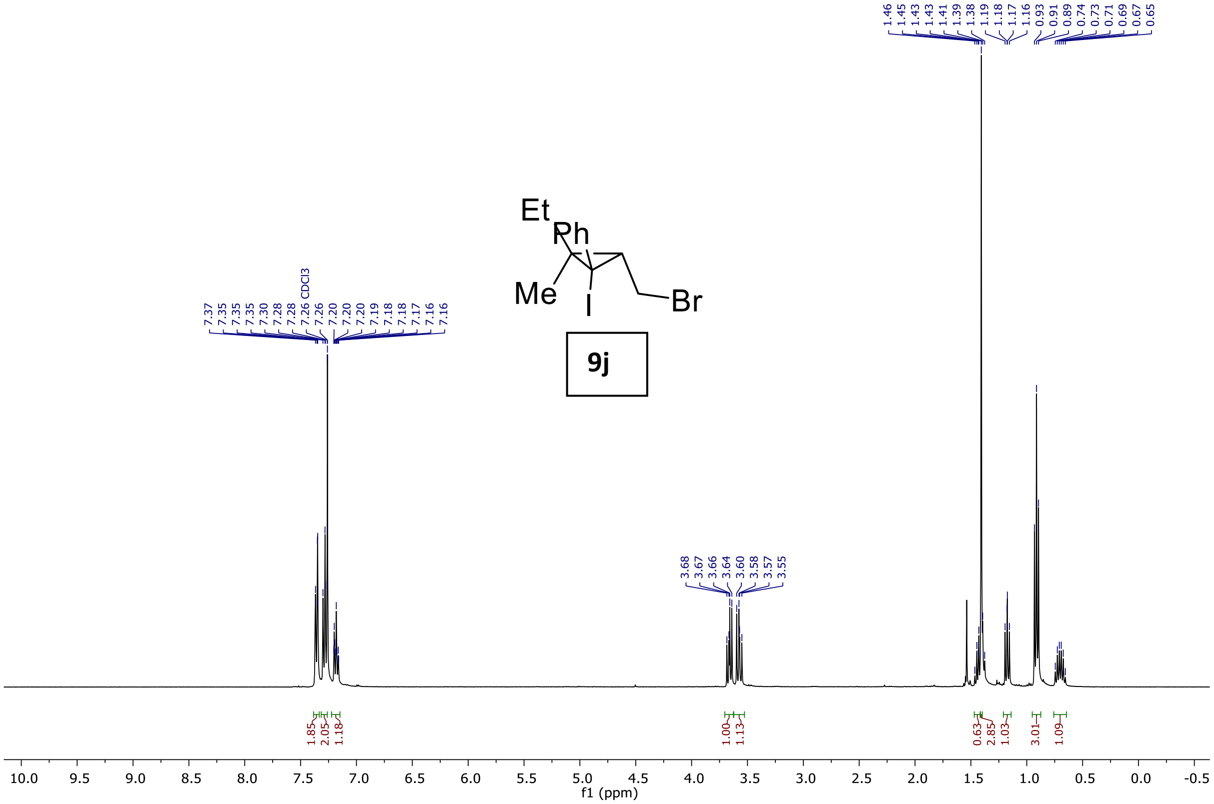
Et
Ph
Me
I
Br
9j
7.37 7.35 7.35 7.35 7.30 7.28 7.28 7.26 CDCl3 7.26 7.20 7.20 7.20 7.19 7.18 7.18 7.17 7.16 7.16
3.68 3.67 3.66 3.64 3.60 3.58 3.57 3.55
1.46 1.45 1.43 1.43 1.41 1.39 1.38 1.19 1.18 1.17 1.16 0.93 0.91 0.89 0.74 0.73 0.71 0.69 0.67 0.65
1.85 2.05 1.18 1.00 1.13 0.63 2.85 1.03 3.01 1.09
f1 (ppm)

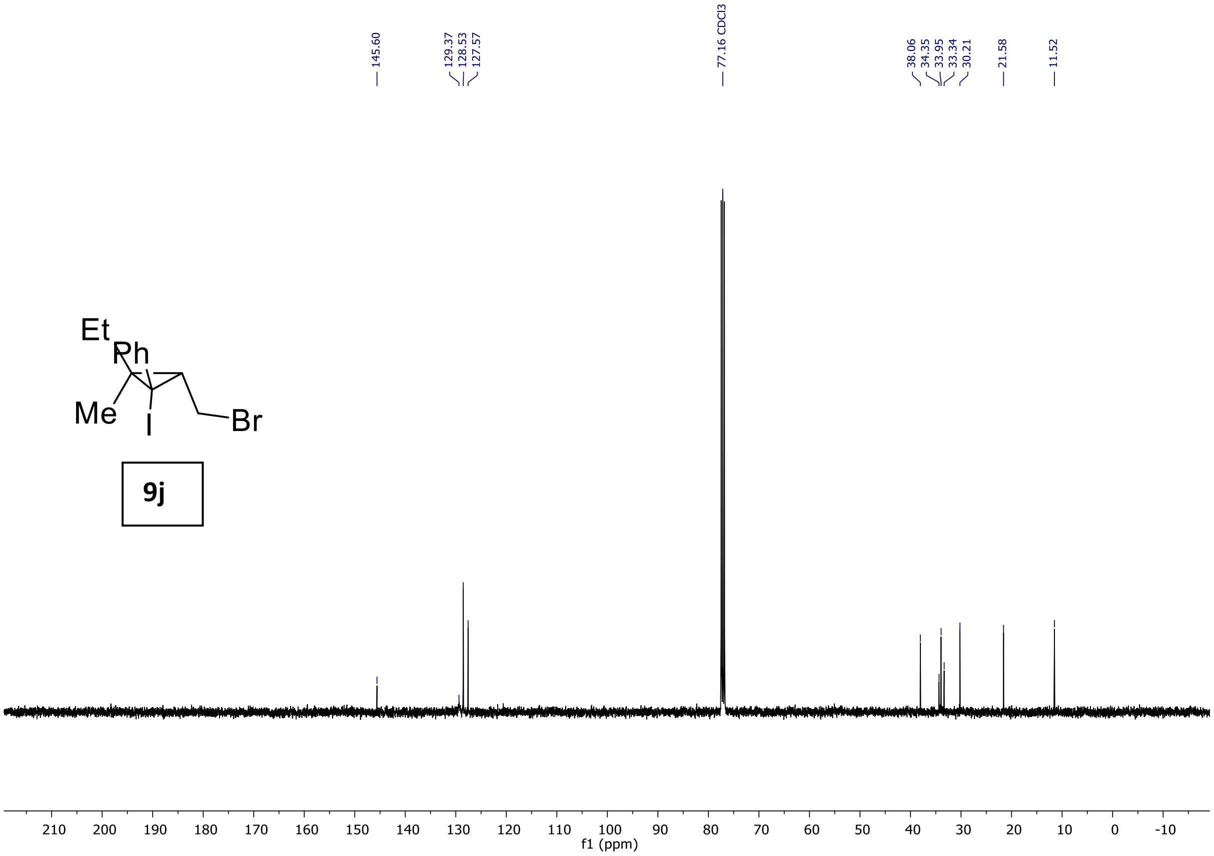
145.60
129.37 128.53 127.57
77.16 CDCl3
38.06 34.35 33.95 33.34 30.21
21.58
11.52
Et
Ph
Me
I
Br
9j
f1 (ppm)

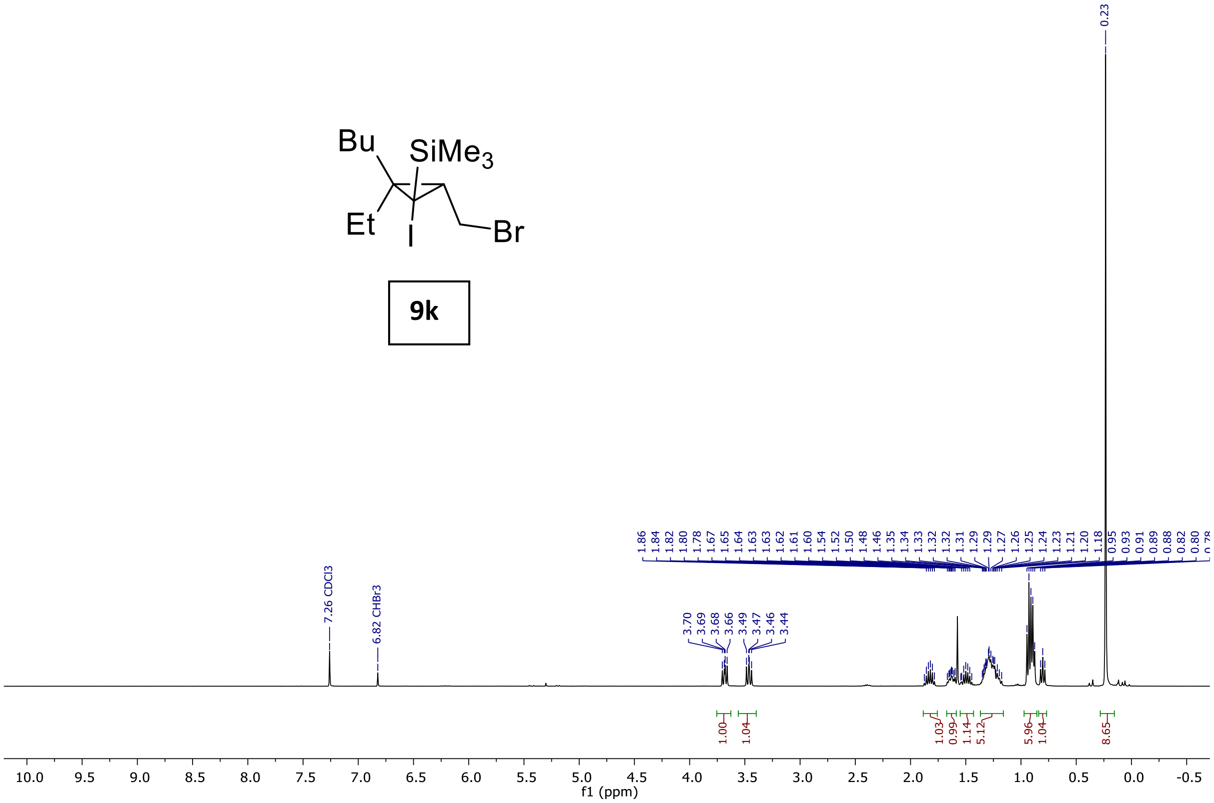
Bu
SiMe3
Et
I
Br
9k
7.26 CDCl3
6.82 CHBr3
3.70 3.69 3.68 3.66 3.49 3.47 3.46 3.44
1.86 1.84 1.82 1.80 1.78 1.67 1.65 1.64 1.63 1.63 1.62 1.61 1.60 1.54 1.52 1.50 1.48 1.46 1.35 1.34 1.33 1.32 1.32 1.31 1.29 1.29 1.27 1.26 1.25 1.24 1.23 1.21 1.20 1.18 0.95 0.93 0.91 0.89 0.88 0.82 0.80
0.23
1.00 1.04 1.03 0.99 1.14 5.12 5.96 1.04 8.65
f1 (ppm)

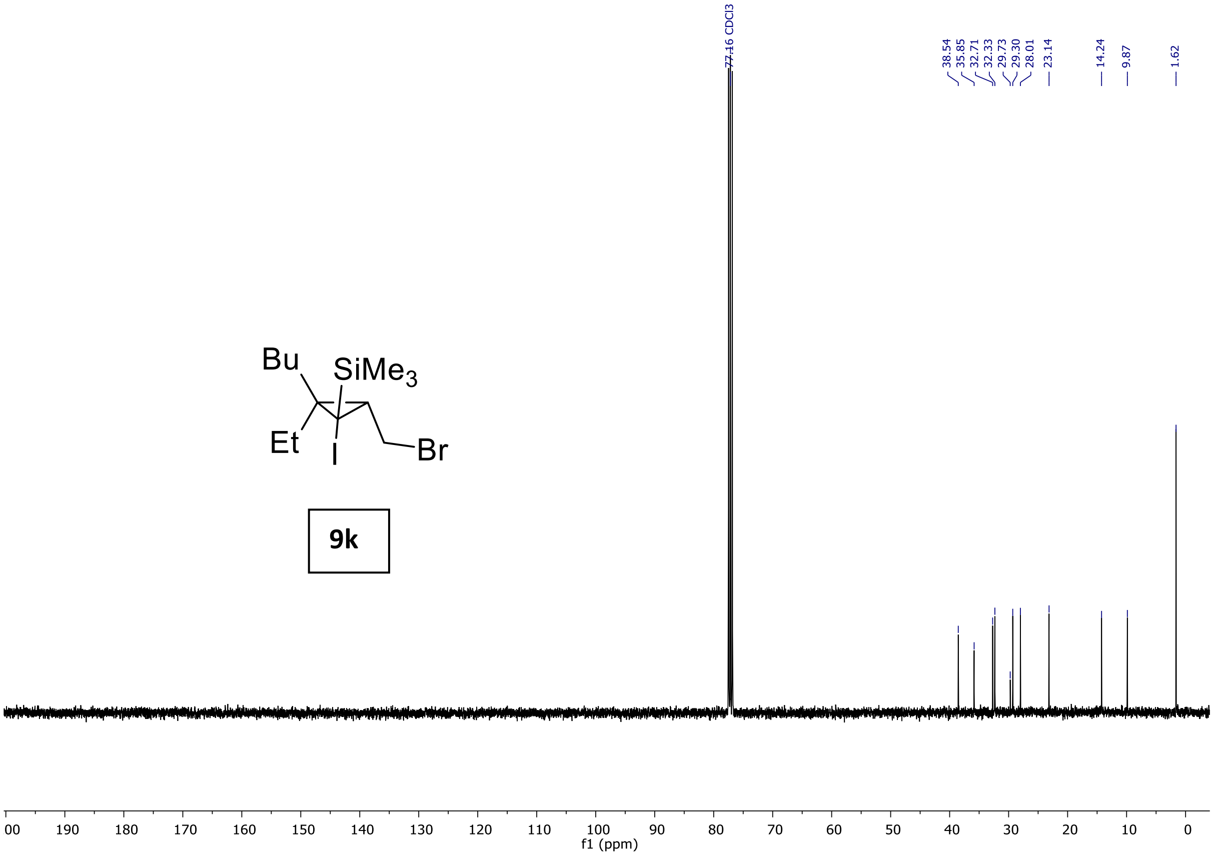
77.16 CDCl3
38.54 35.85 32.71 32.33 29.73 29.30 28.01 23.14 14.24 9.87 1.62
Bu
SiMe3
Et
I
Br
9k
f1 (ppm)

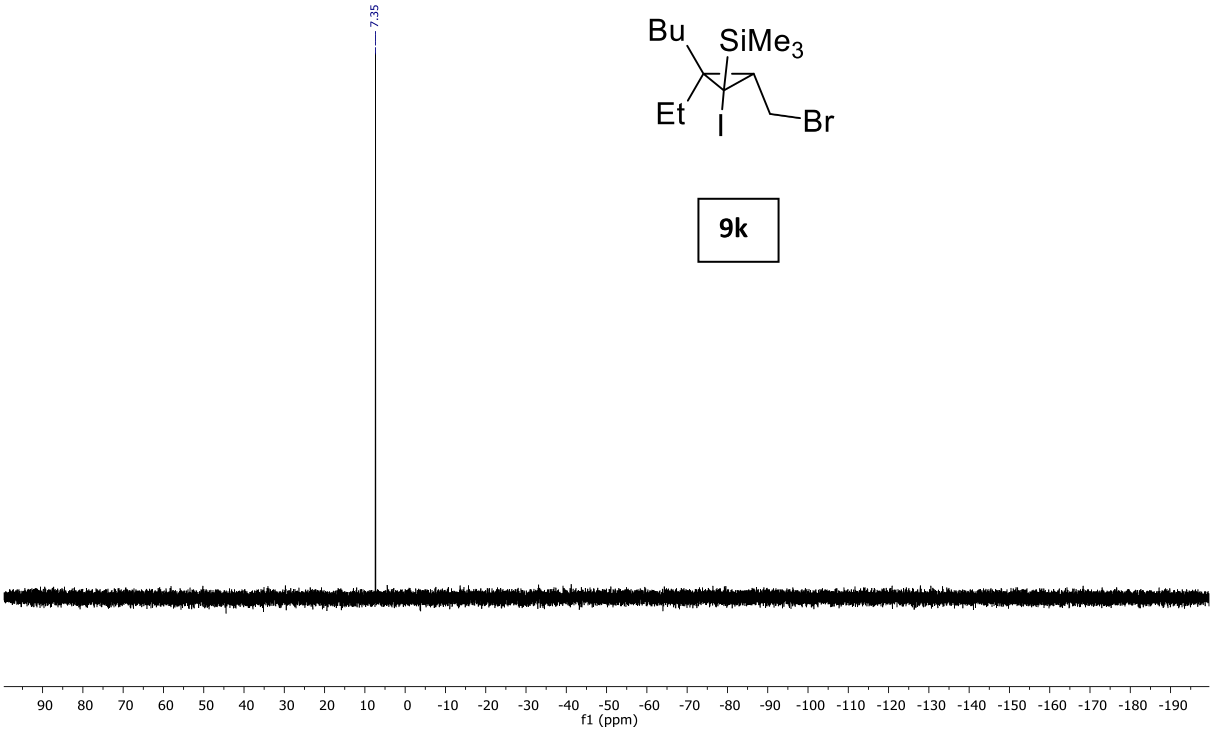

9k

7.35
90 80 70 60 50 40 30 20 10 0 -10 -20 -30 -40 -50 -60 -70 -80 -90 -100 -110 -120 -130 -140 -150 -160 -170 -180 -190
f1 (ppm)

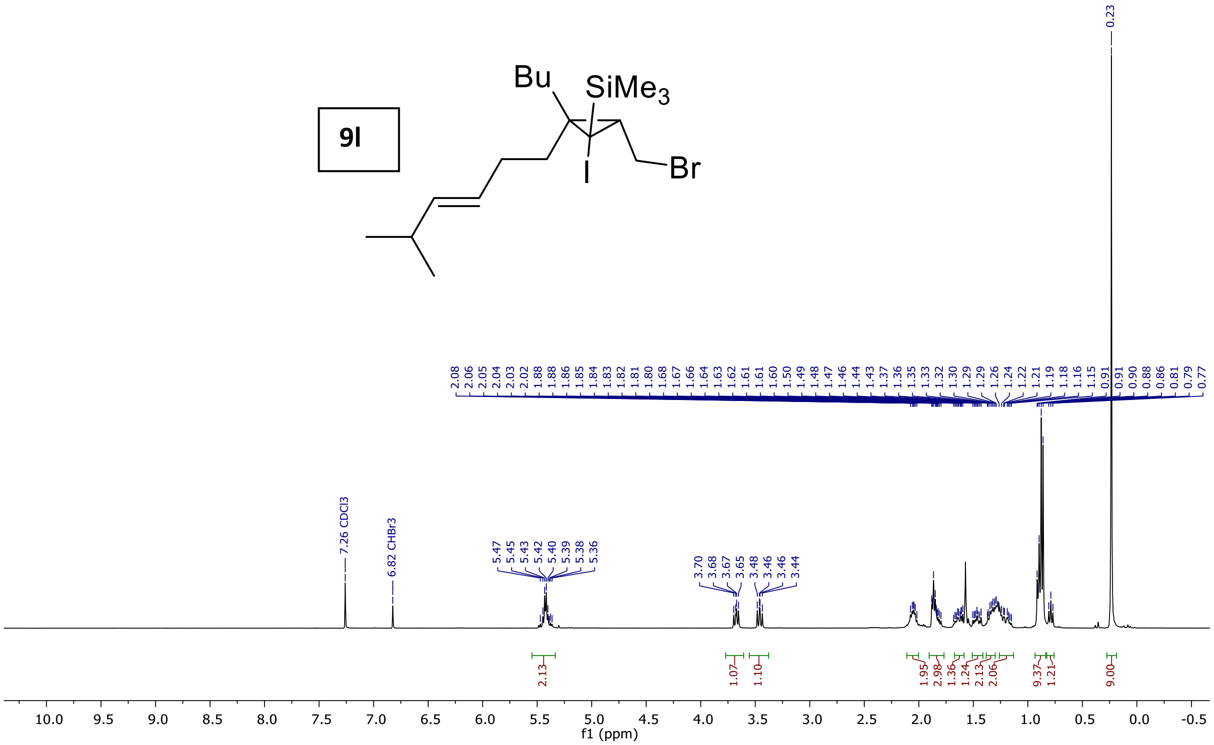
9l
Bu
SiMe3
I
Br
0.23
2.08 2.06 2.05 2.04 2.03 2.02 1.88 1.86 1.85 1.84 1.83 1.82 1.81 1.80 1.68 1.67 1.66 1.64 1.63 1.62 1.61 1.61 1.60 1.50 1.49 1.48 1.47 1.46 1.44 1.43 1.37 1.36 1.35 1.33 1.32 1.30 1.29 1.29 1.26 1.24 1.22 1.21 1.19 1.18 1.16 1.15 0.91 0.91 0.90 0.88 0.86 0.81 0.79 0.77
7.26 CDCl3
6.82 CHBr3
5.47 5.45 5.43 5.42 5.40 5.39 5.38 5.36
3.70 3.68 3.67 3.65 3.48 3.46 3.46 3.44
2.13 1.07 1.10 1.95 2.98 1.36 1.24 2.13 2.06 9.37 1.21 9.00
f1 (ppm)

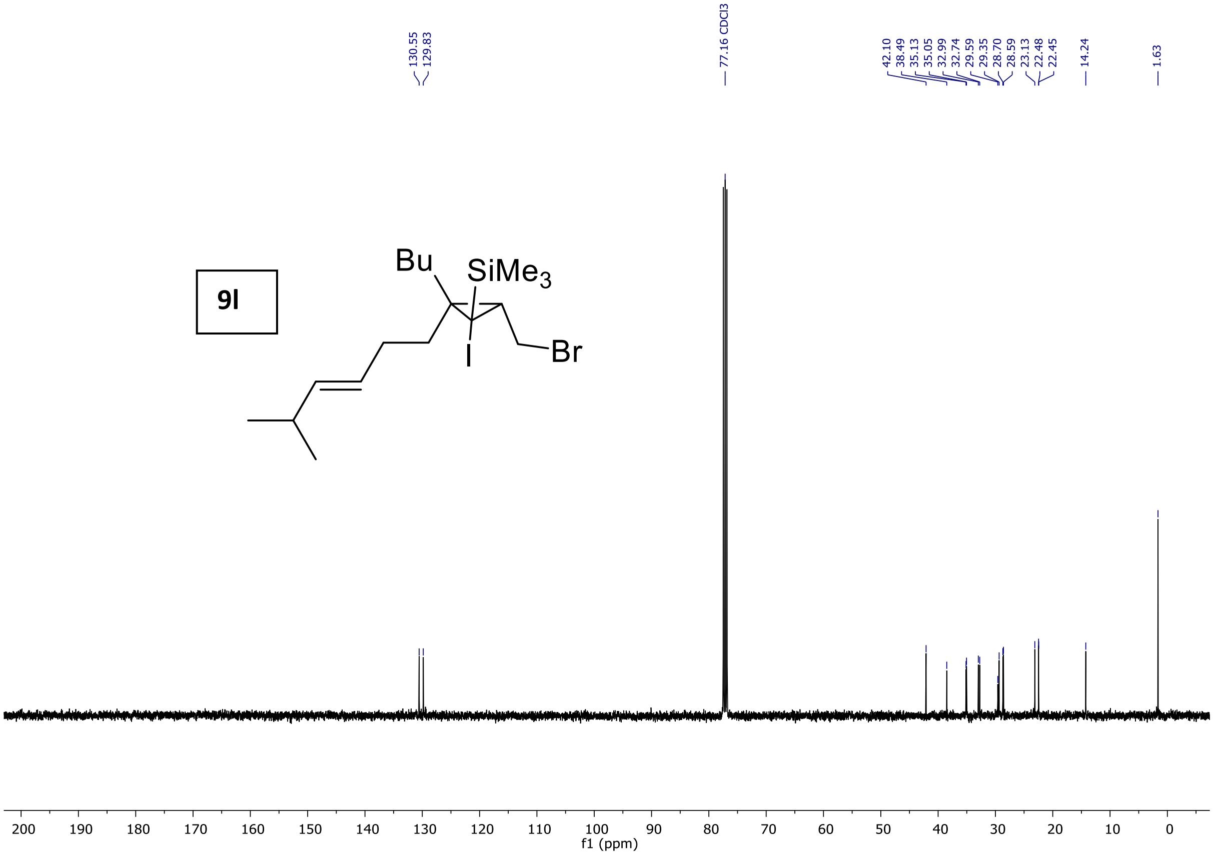
130.55 129.83
77.16 CDCl3
42.10 38.49 35.13 35.05 32.99 32.74 29.59 29.35 28.70 28.59 23.13 22.48 22.45 14.24 1.63
9l
Bu
SiMe3
I
Br
f1 (ppm)

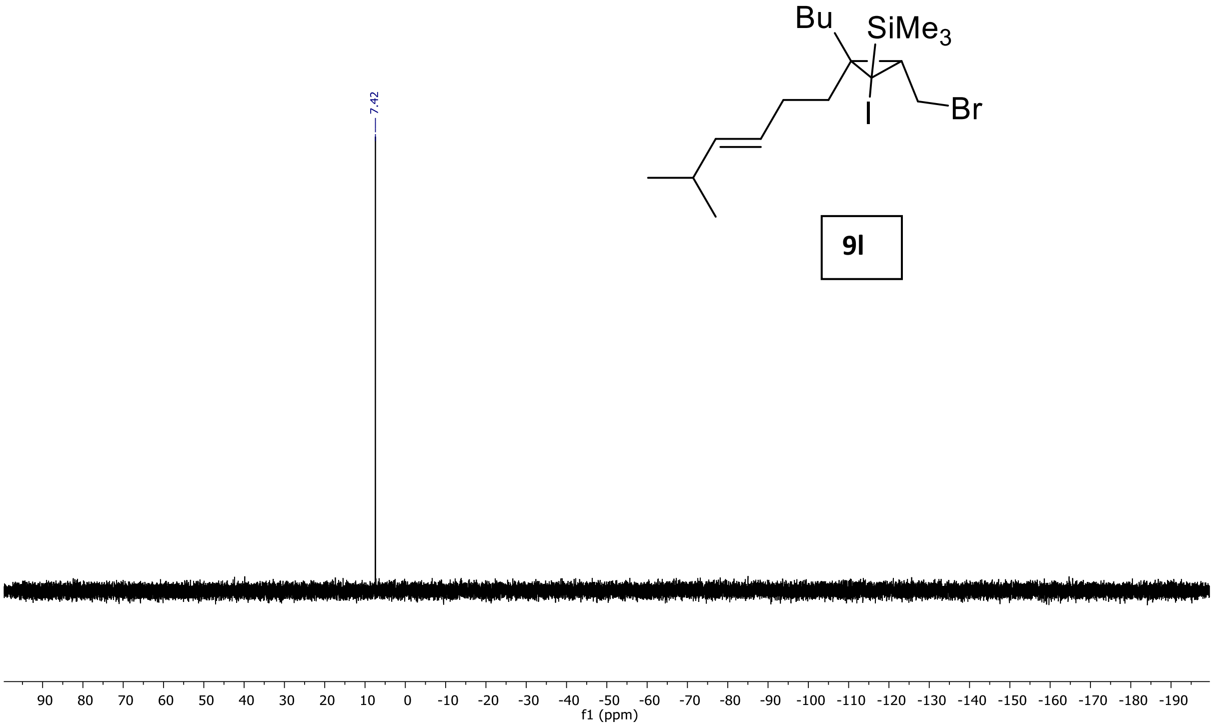
Bu
$SiMe_3$
I
Br
7.42
90 80 70 60 50 40 30 20 10 0 -10 -20 -30 -40 -50 -60 -70 -80 -90 -100 -110 -120 -130 -140 -150 -160 -170 -180 -190
f1 (ppm)

9l

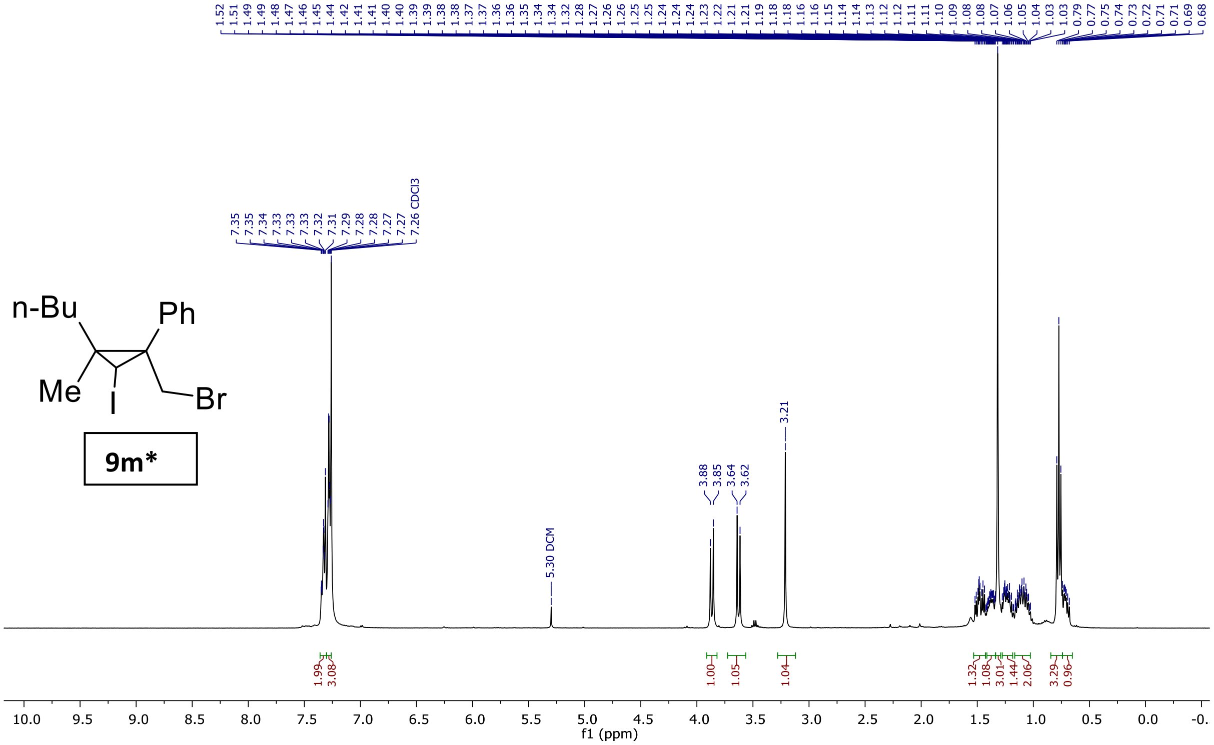
n-Bu
Ph
Me
I
Br
9m*
5.30 DCM
3.21
f1 (ppm)

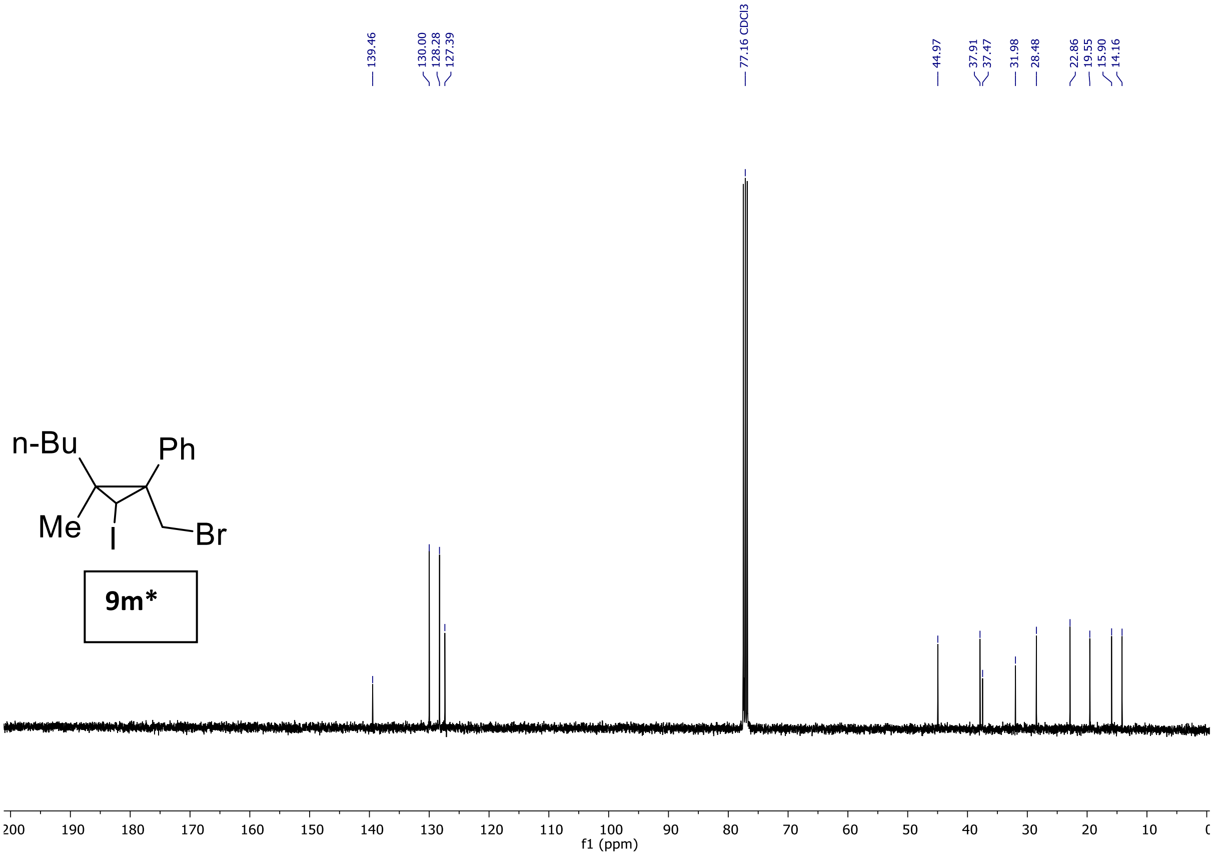
n-Bu
Ph
Me
I
Br
9m*
139.46
130.00
128.28
127.39
77.16 CDCl3
44.97
37.91
37.47
31.98
28.48
22.86
19.55
15.90
14.16
f1 (ppm)

HPLC chromatogram of racemic compound (9m)

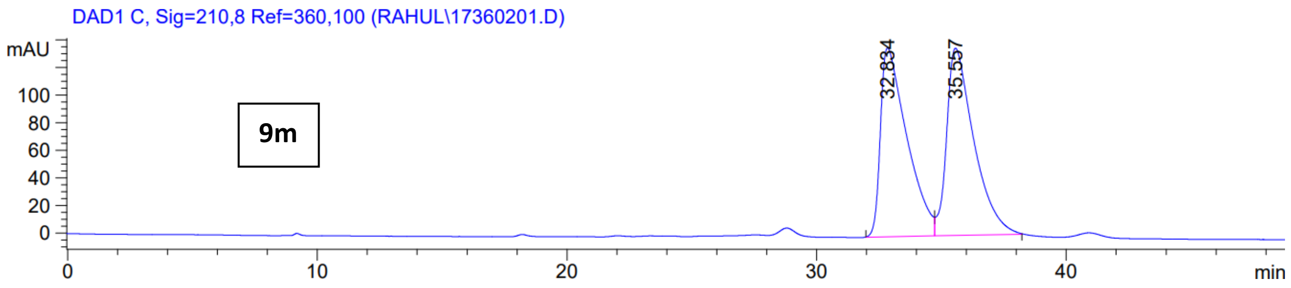

Signal 3: DAD1 C, Sig=210,8 Ref=360,100

Peak #	RetTime [min]	Type	Width [min]	Area [mAU*s]	Height [mAU]	Area %
1	32.834	BV	1.0266	1.00897e4	137.10295	49.3906
2	35.557	VB	1.0909	1.03387e4	136.00490	50.6094
Totals :				2.04285e4	273.10785	

CHIRACEL OD column, size: 1cmI.D x 25 cmL (n-Hexane, 1.5 mL/min, 5 °C)

HPLC chromatogram of enantioenriched compound (9m*)

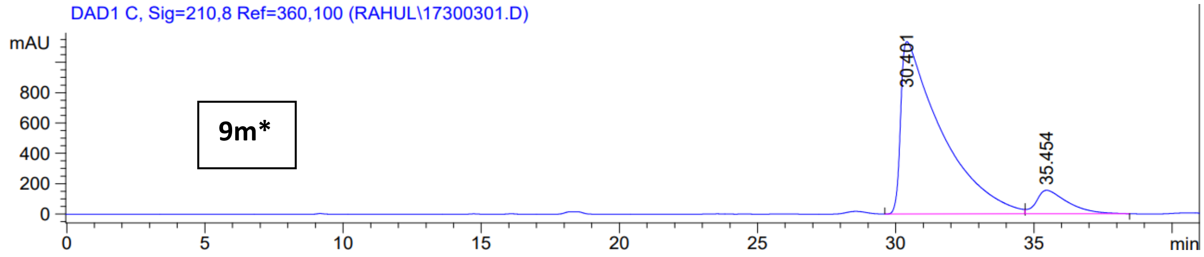

Signal 3: DAD1 C, Sig=210,8 Ref=360,100

Peak #	RetTime [min]	Type	Width [min]	Area [mAU*s]	Height [mAU]	Area %
1	30.401	VV	1.3478	1.16868e5	1136.44775	90.4363
2	35.454	VB	1.1204	1.23589e4	155.63481	9.5637
Totals :				1.29227e5	1292.08257	

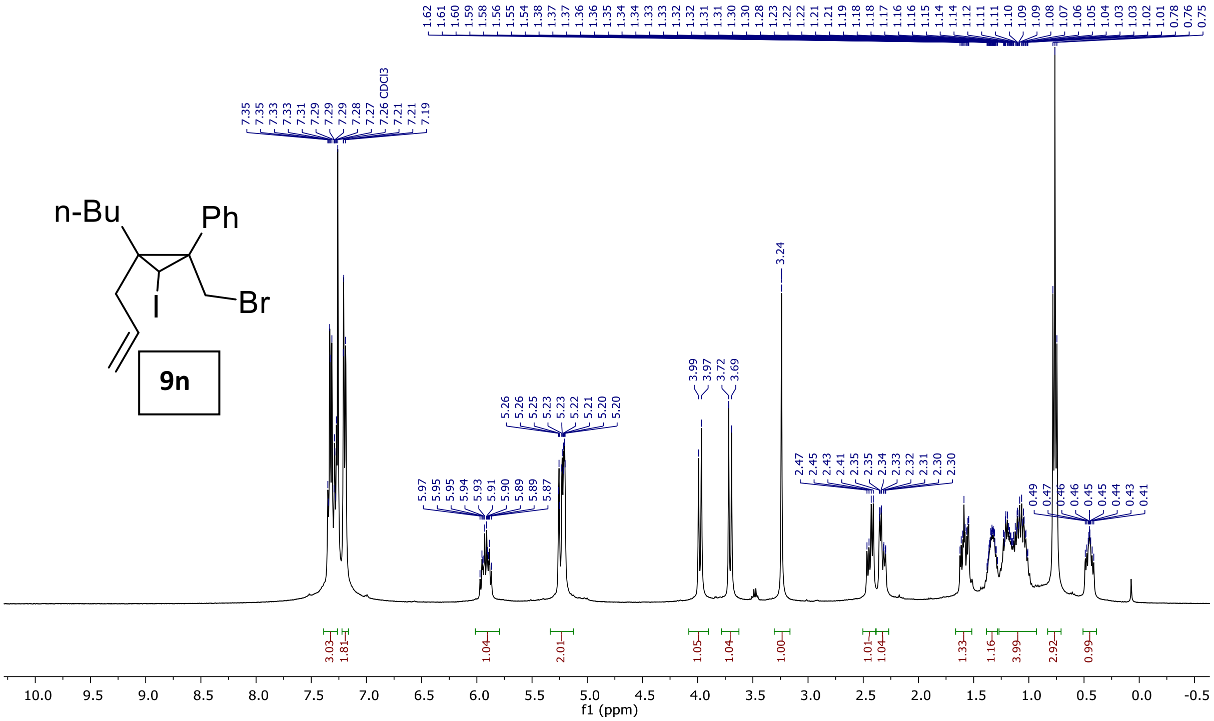
n-Bu
Ph
I
Br
9n
3.24
f1 (ppm)

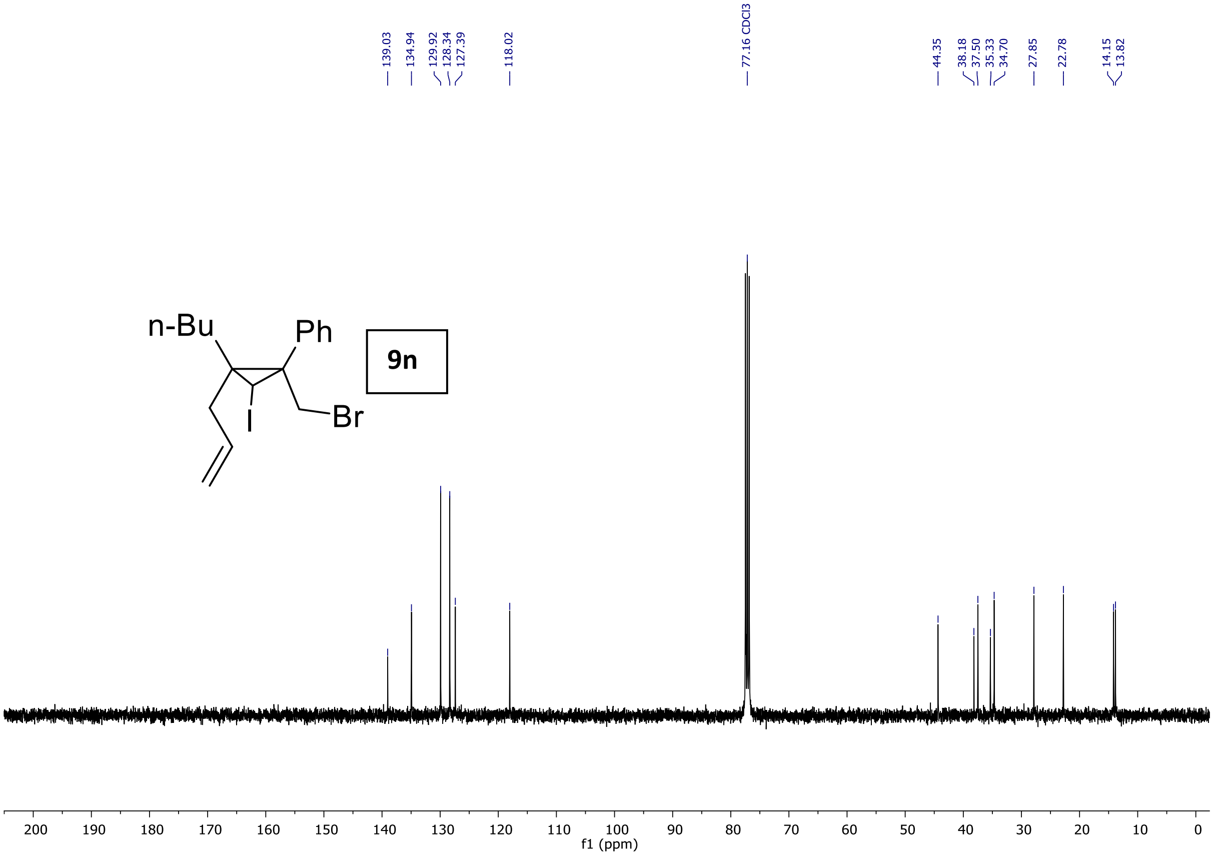
139.03
134.94
129.92
128.34
127.39
118.02
77.16 CDCl3
44.35
38.18
37.50
35.33
34.70
27.85
22.78
14.15
13.82
n-Bu
Ph
I
Br
9n
f1 (ppm)

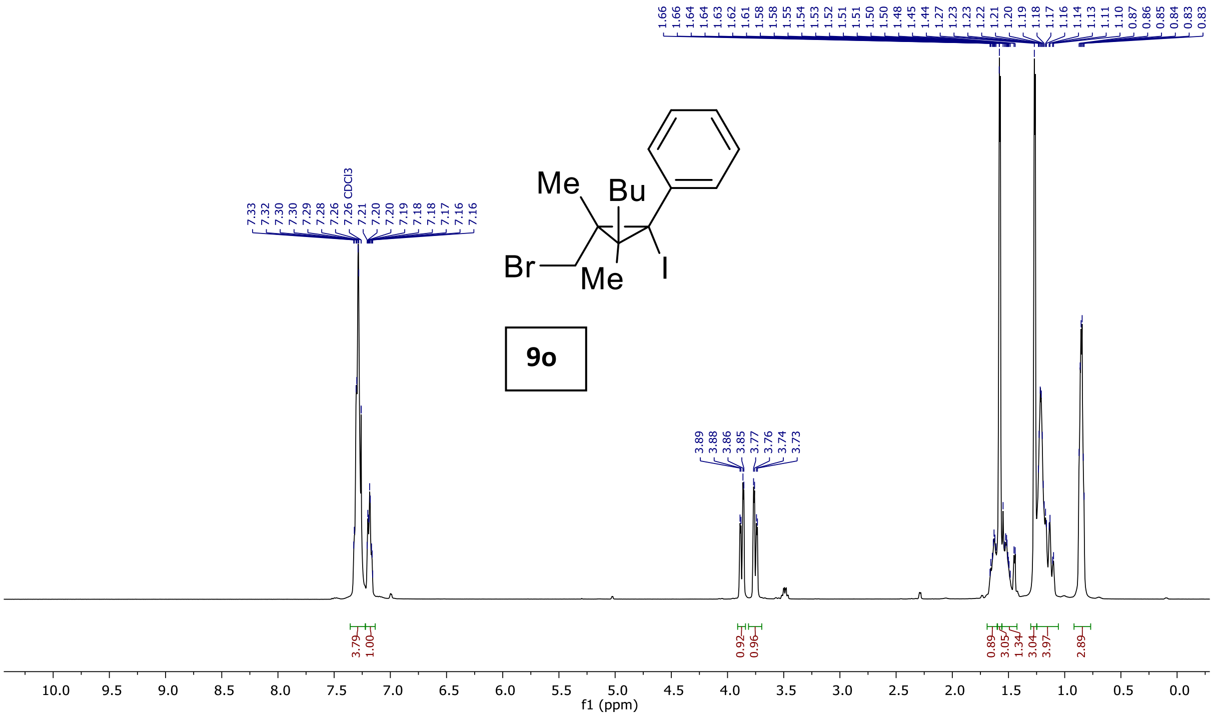
7.33 7.32 7.30 7.30 7.29 7.28 7.26 7.26 CDCl3 7.21 7.20 7.20 7.19 7.18 7.18 7.17 7.16 7.16
3.89 3.88 3.86 3.85 3.77 3.76 3.74 3.73
1.66 1.66 1.64 1.63 1.62 1.61 1.58 1.58 1.55 1.54 1.53 1.52 1.51 1.51 1.50 1.50 1.48 1.45 1.44 1.27 1.23 1.23 1.22 1.21 1.20 1.19 1.18 1.17 1.16 1.14 1.13 1.11 1.10 0.87 0.86 0.85 0.84 0.83 0.83
Me
Bu
Br
Me
I
9o
3.79 1.00
0.92 0.96
0.89 3.05 1.34 3.04 3.97 2.89
f1 (ppm)

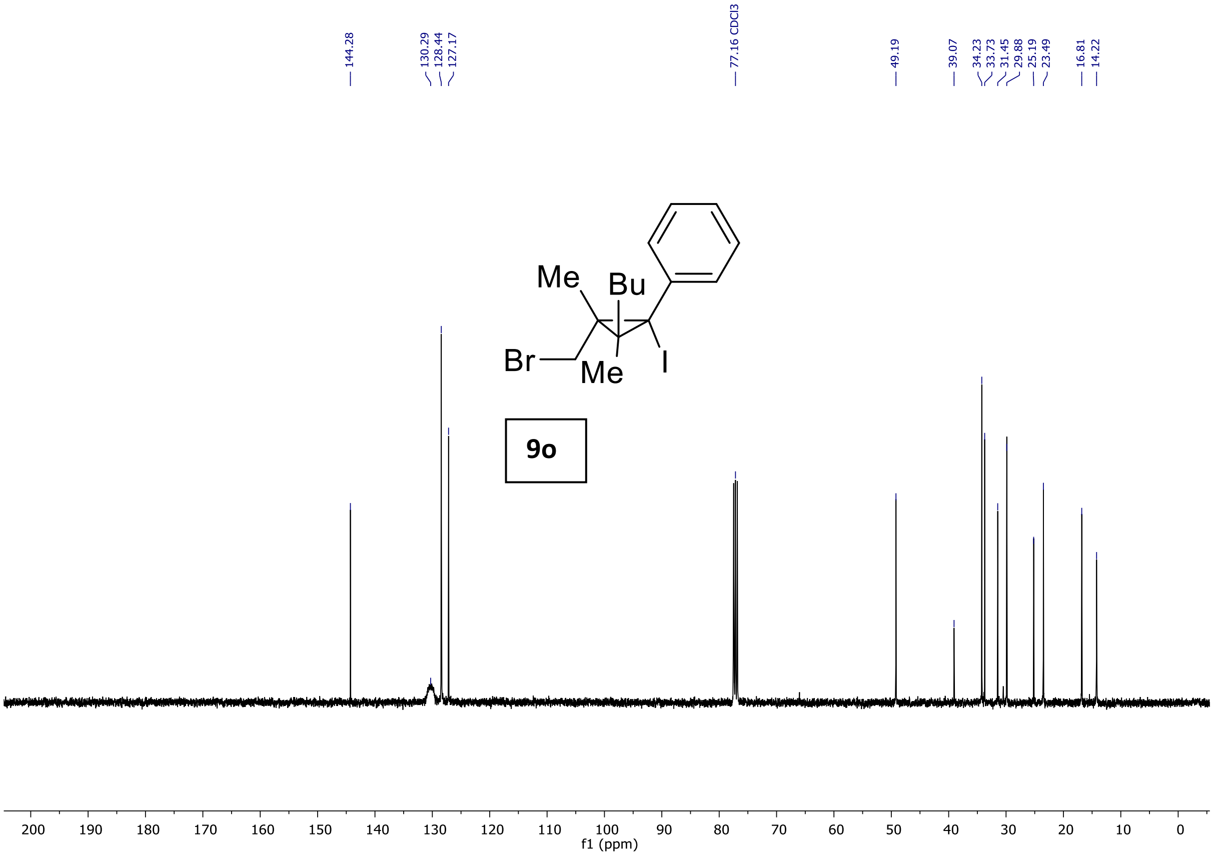
144.28
130.29 128.44 127.17
77.16 CDCl3
49.19
39.07 34.23 33.73 31.45 29.88 25.19 23.49
16.81 14.22
Me
Bu
Br
Me
I
9o
f1 (ppm)

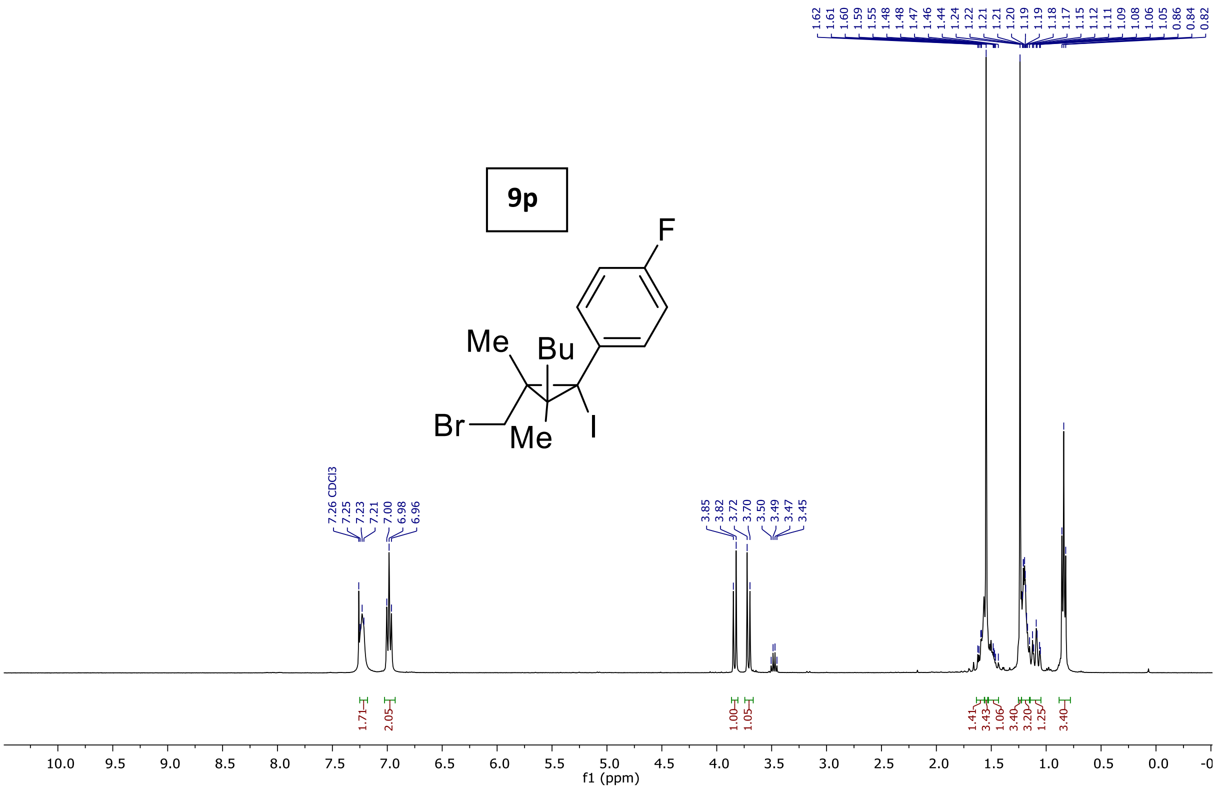

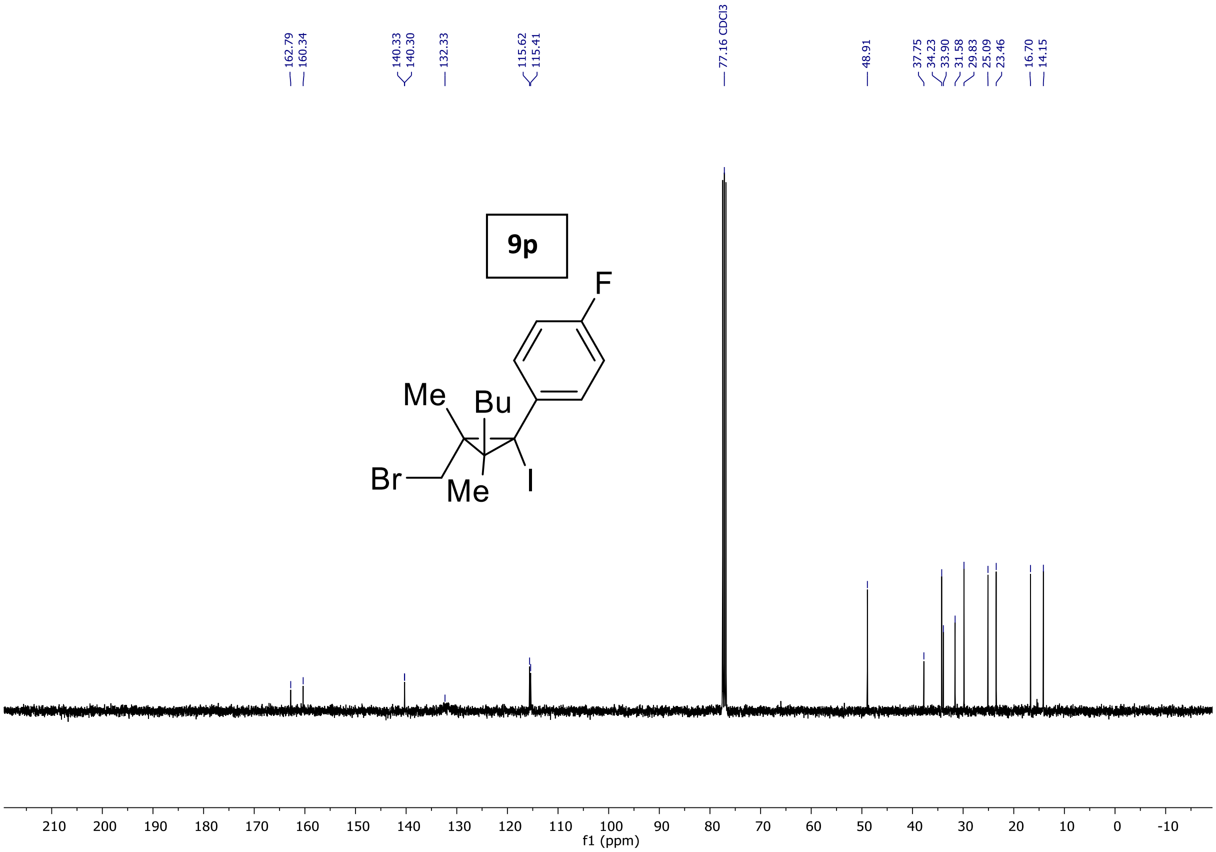

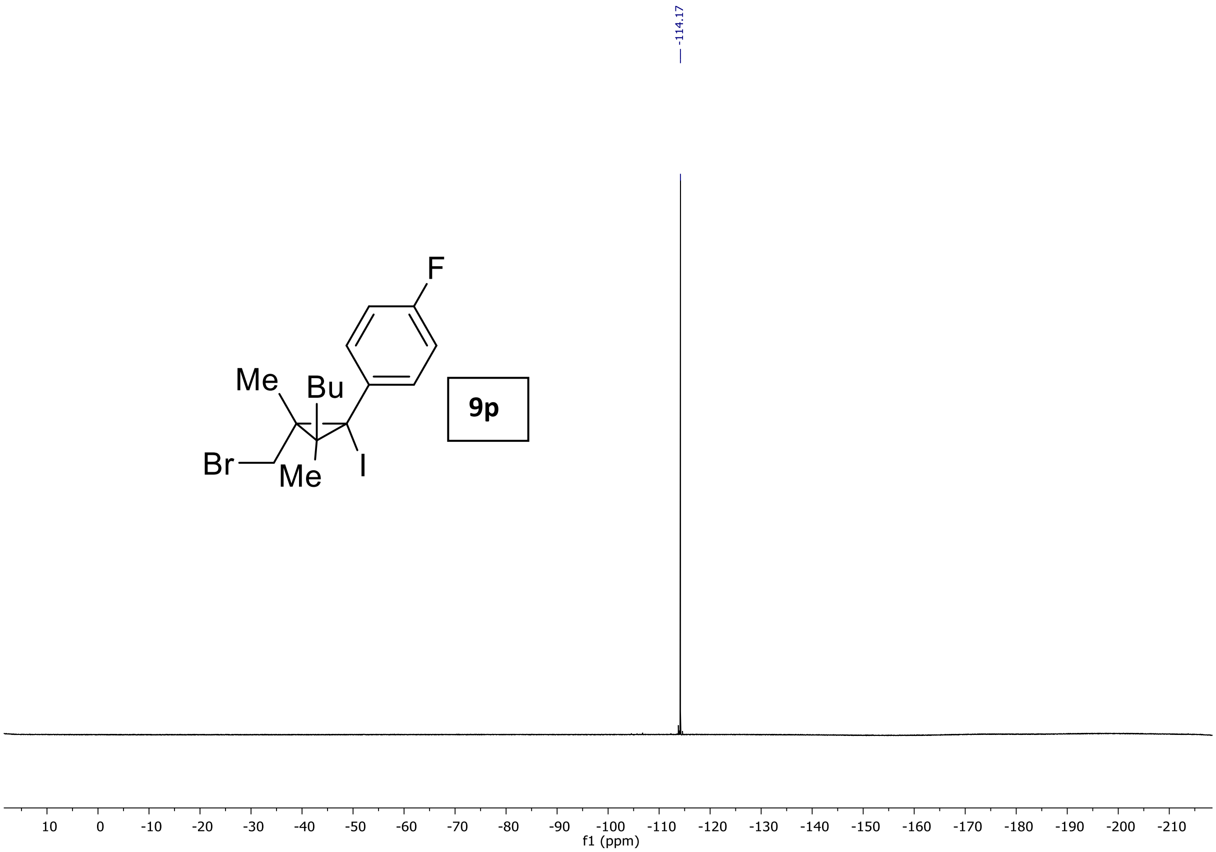

-114.17
F
Me
Bu
Br
Me
I
9p
10 0 -10 -20 -30 -40 -50 -60 -70 -80 -90 -100 -110 -120 -130 -140 -150 -160 -170 -180 -190 -200 -210
f1 (ppm)

9q

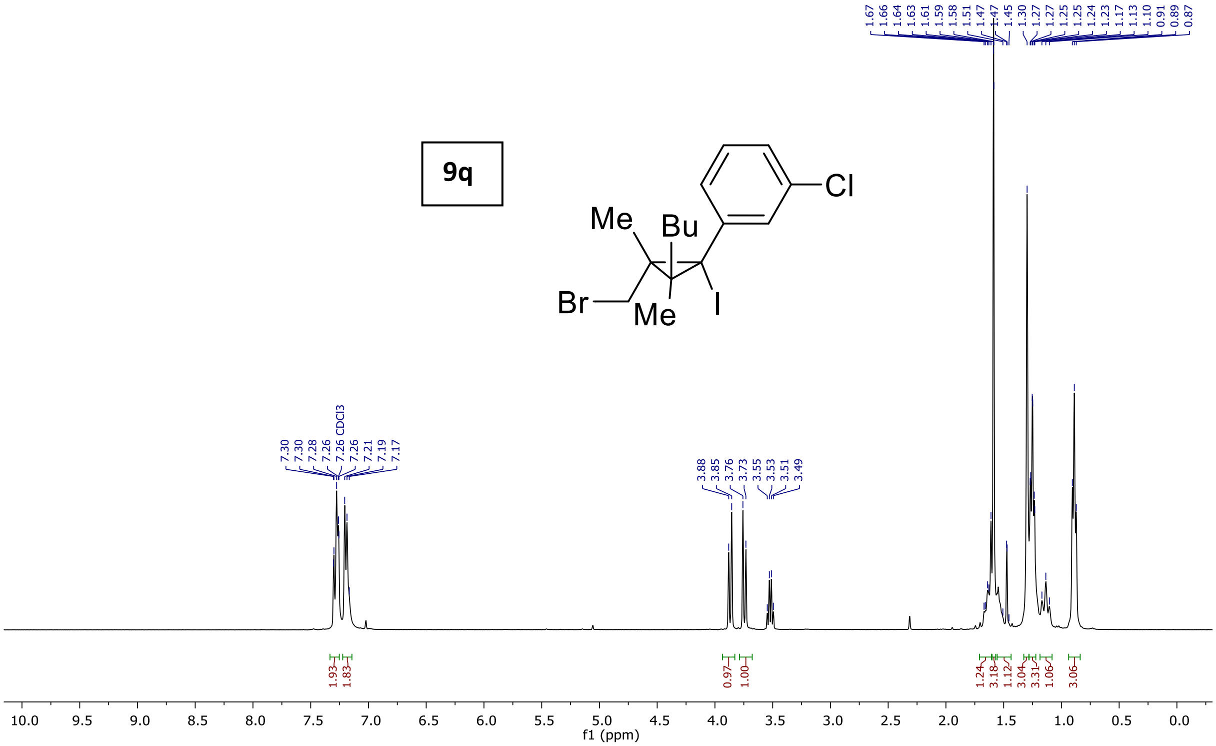
Me
Bu
Cl
Br
Me
I
7.30
7.30
7.28
7.26
7.26 CDCl3
7.26
7.21
7.19
7.17
3.88
3.85
3.76
3.73
3.55
3.53
3.51
3.49
1.67
1.66
1.64
1.63
1.61
1.59
1.58
1.51
1.47
1.47
1.45
1.30
1.27
1.27
1.25
1.25
1.24
1.23
1.17
1.13
1.10
0.91
0.89
0.87
1.93
1.83
0.97
1.00
1.24
3.18
1.12
3.04
3.31
1.06
3.06
f1 (ppm)

9q

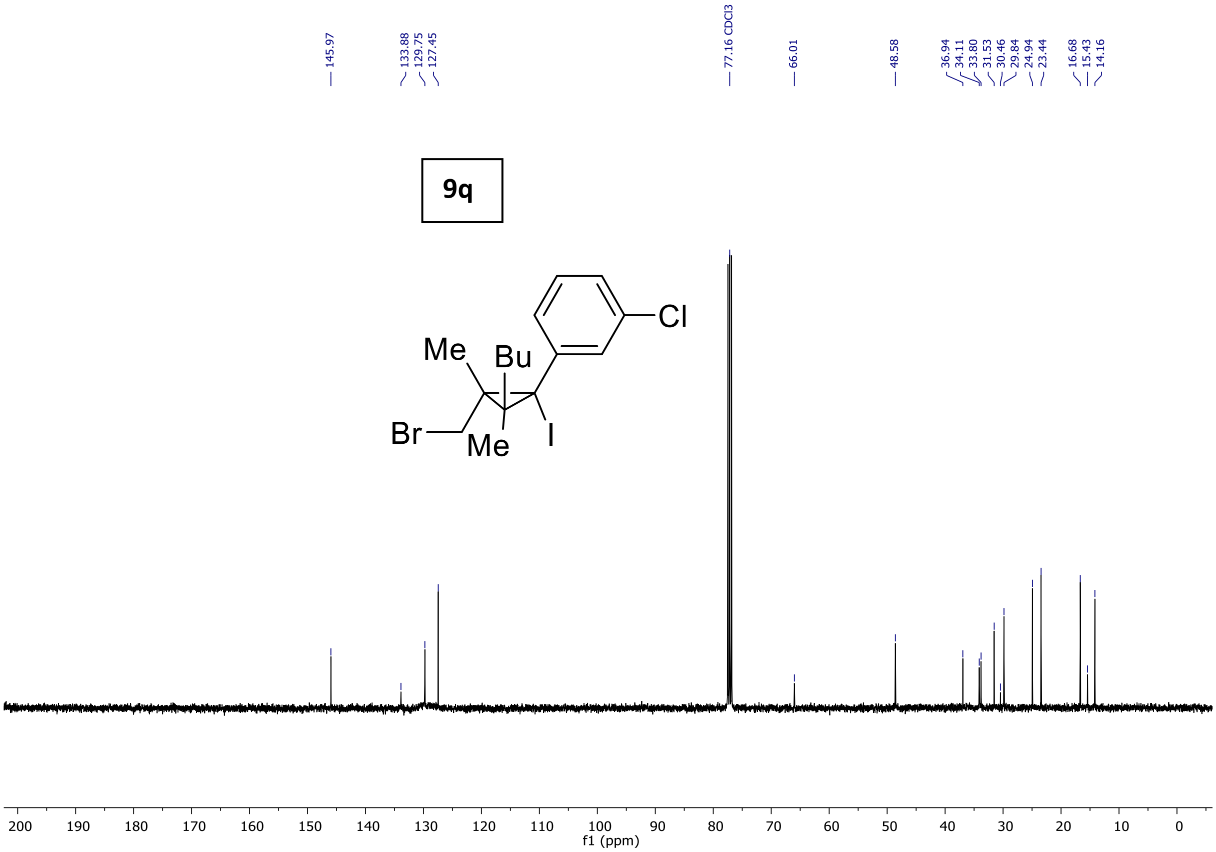
Me
Bu
Cl
Br
Me
I
145.97
133.88
129.75
127.45
77.16 CDCl3
66.01
48.58
36.94
34.11
33.80
31.53
30.46
29.84
24.94
23.44
16.68
15.43
14.16
f1 (ppm)

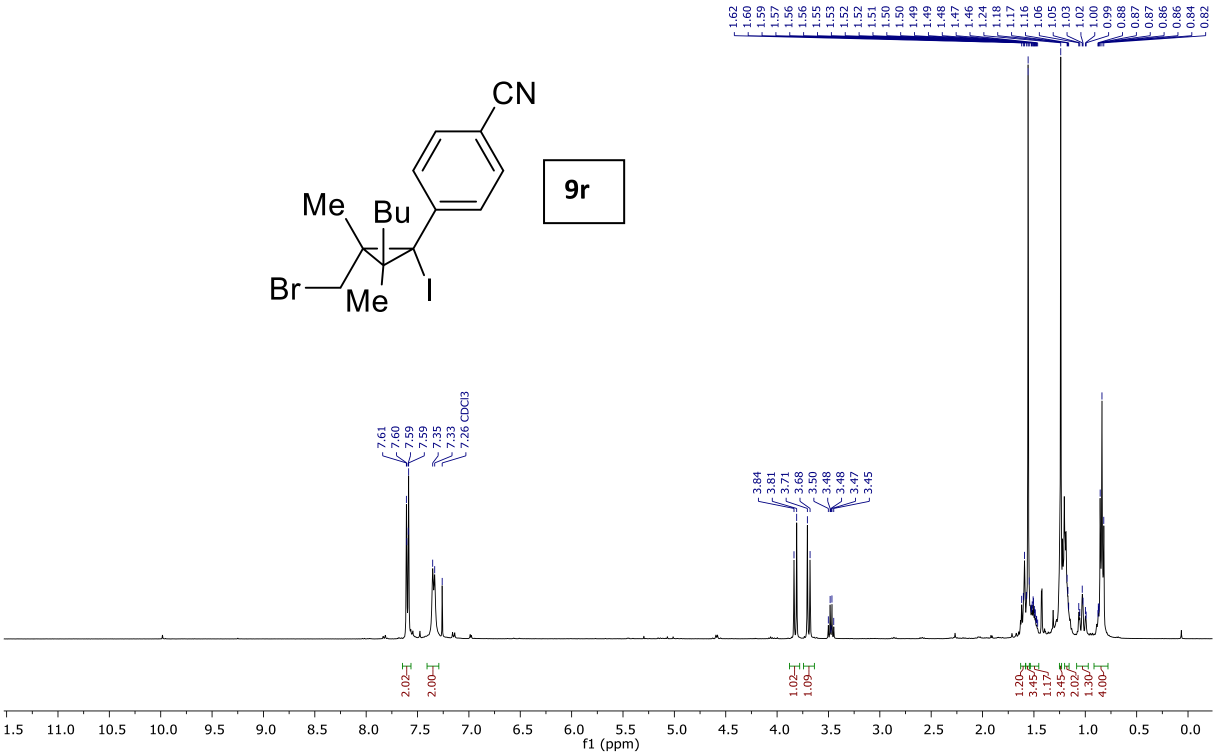
CN
9r
Me
Bu
Br
Me
I
1.62 1.60 1.59 1.57 1.56 1.56 1.55 1.53 1.52 1.52 1.51 1.50 1.50 1.49 1.49 1.48 1.47 1.46 1.24 1.18 1.17 1.16 1.06 1.05 1.03 1.02 1.00 0.99 0.88 0.87 0.87 0.86 0.86 0.84 0.82
7.61 7.60 7.59 7.59 7.35 7.33 7.26 CDCl3
3.84 3.81 3.71 3.68 3.50 3.48 3.48 3.47 3.45
2.02 2.00
1.02 1.09
1.20 3.45 1.17 3.45 2.02 1.30 4.00
1.5 11.0 10.5 10.0 9.5 9.0 8.5 8.0 7.5 7.0 6.5 6.0 5.5 5.0 4.5 4.0 3.5 3.0 2.5 2.0 1.5 1.0 0.5 0.0
f1 (ppm)

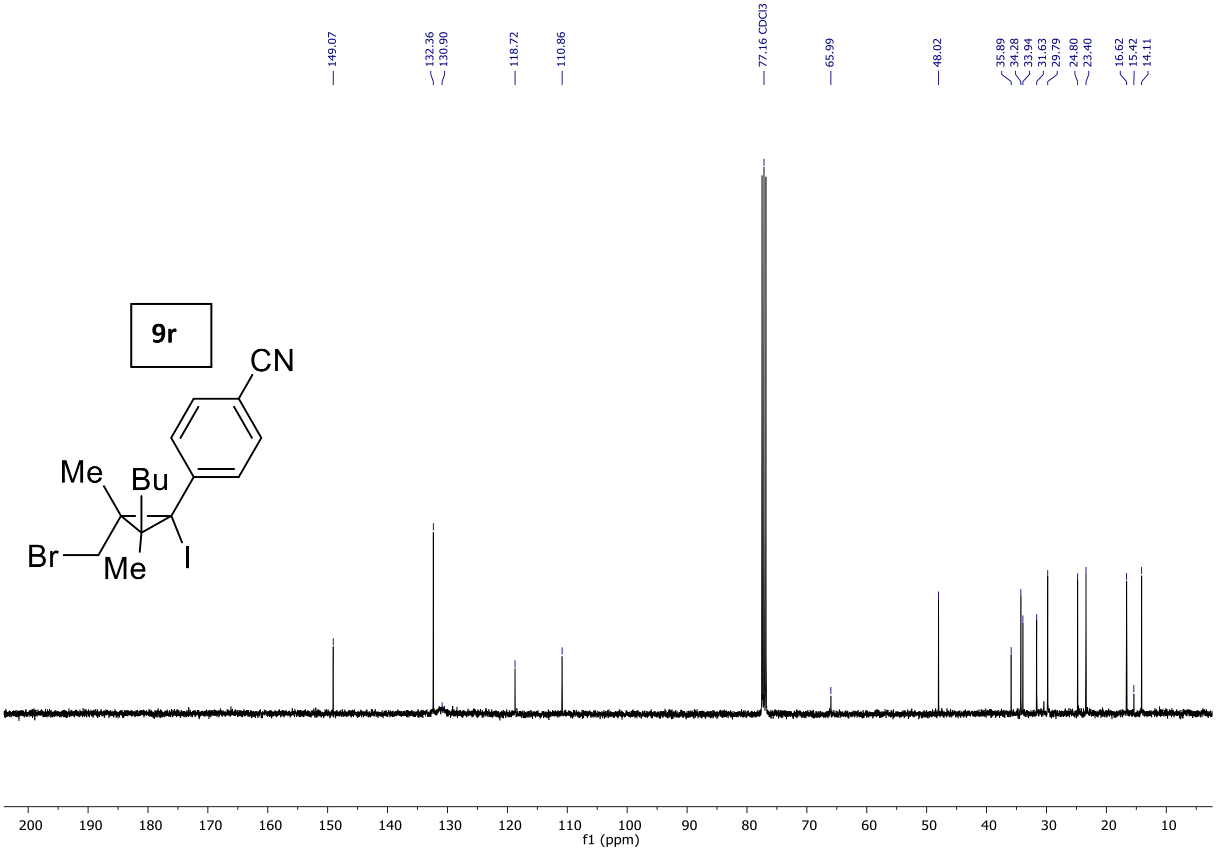
149.07
132.36 130.90
118.72
110.86
77.16 CDCl3
65.99
48.02
35.89 34.28 33.94 31.63 29.79 24.80 23.40
16.62 15.42 14.11
9r
CN
Me
Bu
Br
Me
I
200 190 180 170 160 150 140 130 120 110 100 90 80 70 60 50 40 30 20 10
f1 (ppm)

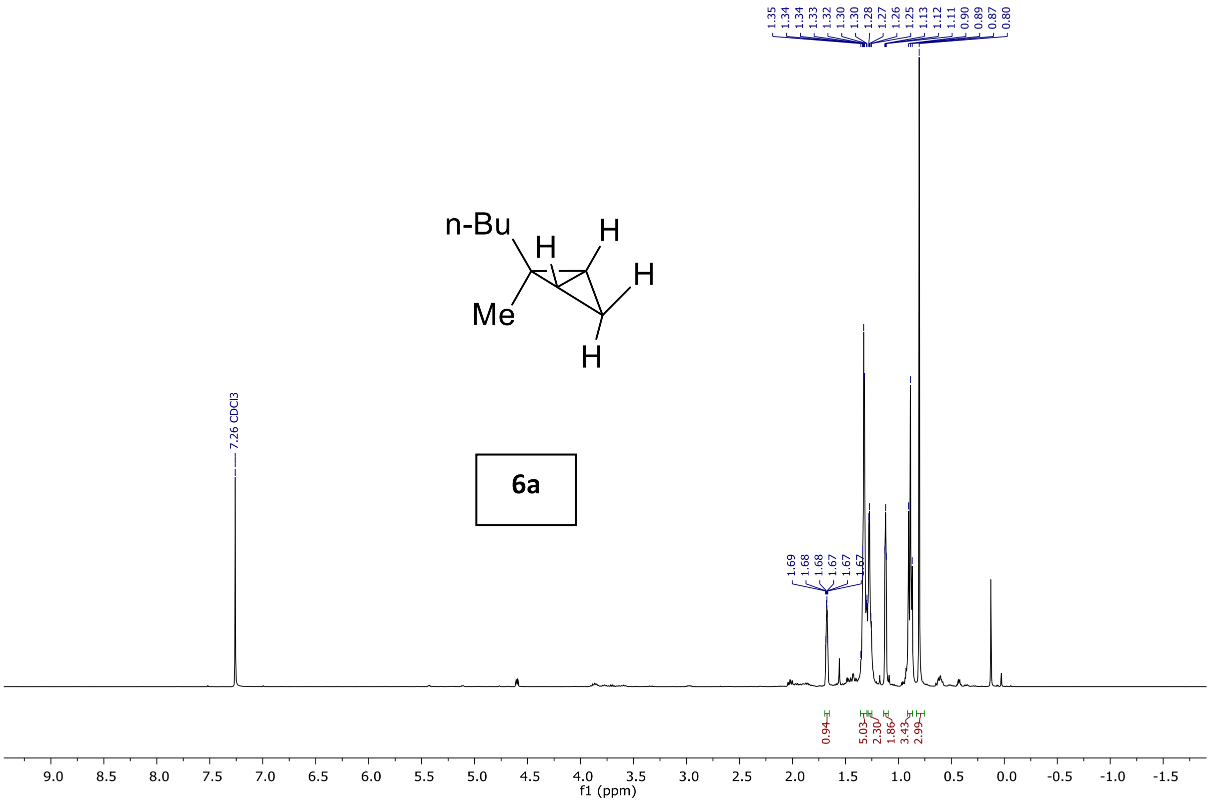
1.35
1.34
1.34
1.33
1.32
1.30
1.30
1.28
1.27
1.26
1.25
1.13
1.12
1.11
0.90
0.89
0.87
0.80
n-Bu
H
H
H
Me
H
7.26 CDCl3
6a
1.69
1.68
1.68
1.67
1.67
1.67
0.94
5.03
2.30
1.86
3.43
2.99
9.0 8.5 8.0 7.5 7.0 6.5 6.0 5.5 5.0 4.5 4.0 3.5 3.0 2.5 2.0 1.5 1.0 0.5 0.0 -0.5 -1.0 -1.5
f1 (ppm)

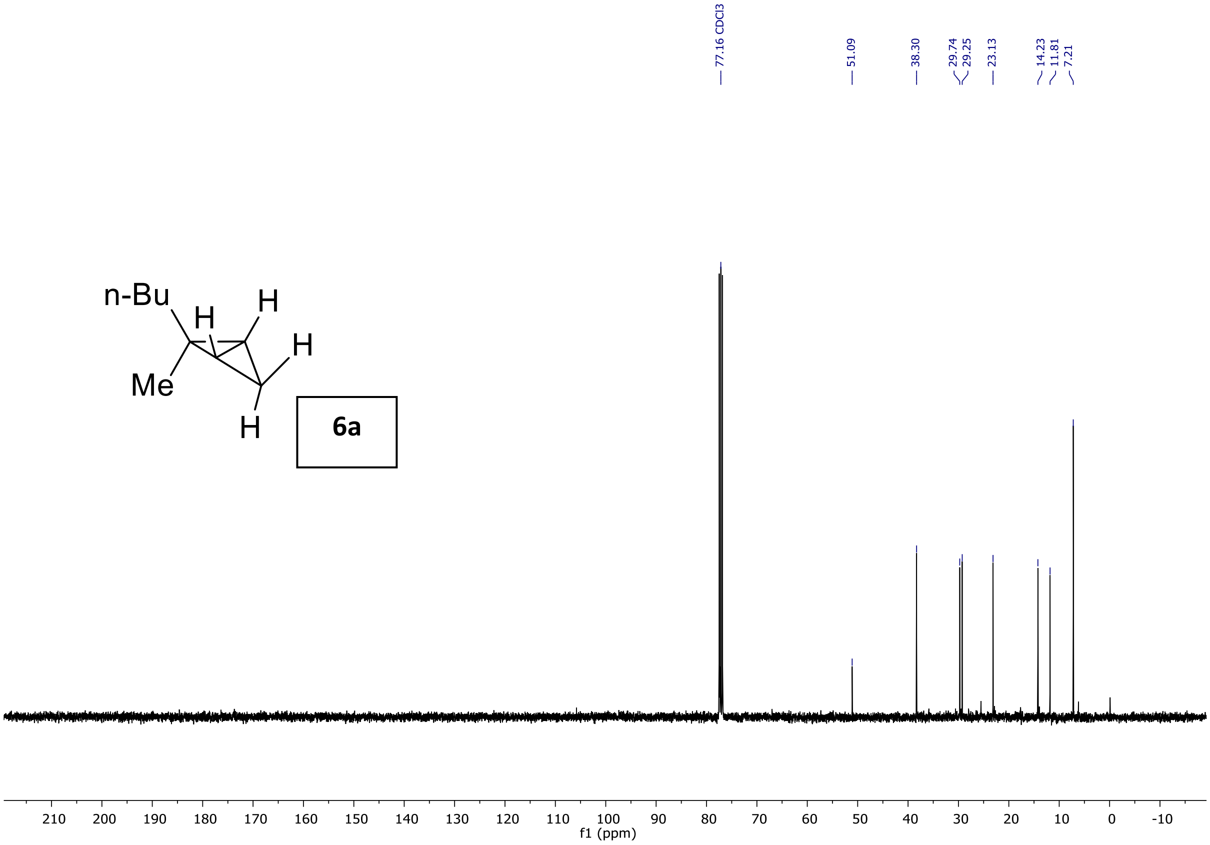
77.16 CDCl3
51.09
38.30
29.74
29.25
23.13
14.23
11.81
7.21
n-Bu
H
H
H
Me
H
6a
210 200 190 180 170 160 150 140 130 120 110 100 90 80 70 60 50 40 30 20 10 0 -10
f1 (ppm)

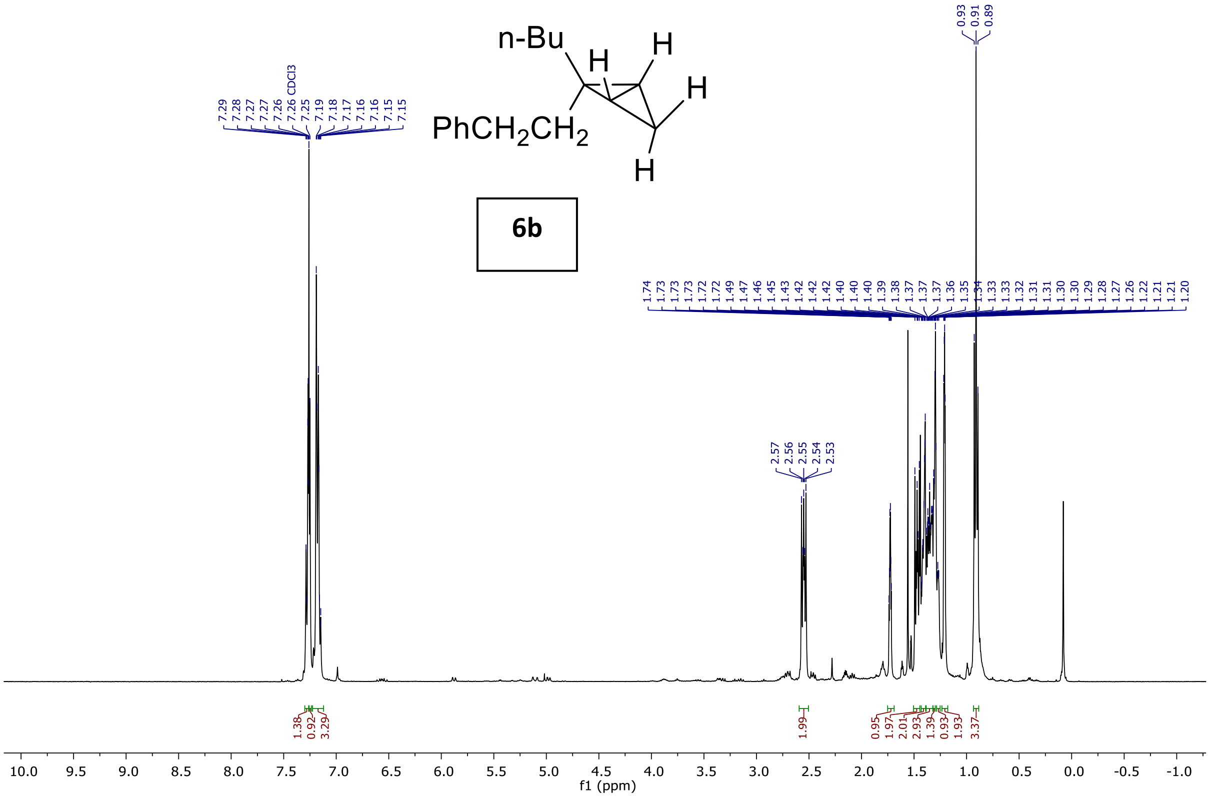
n-Bu
PhCH2CH2
6b
f1 (ppm)

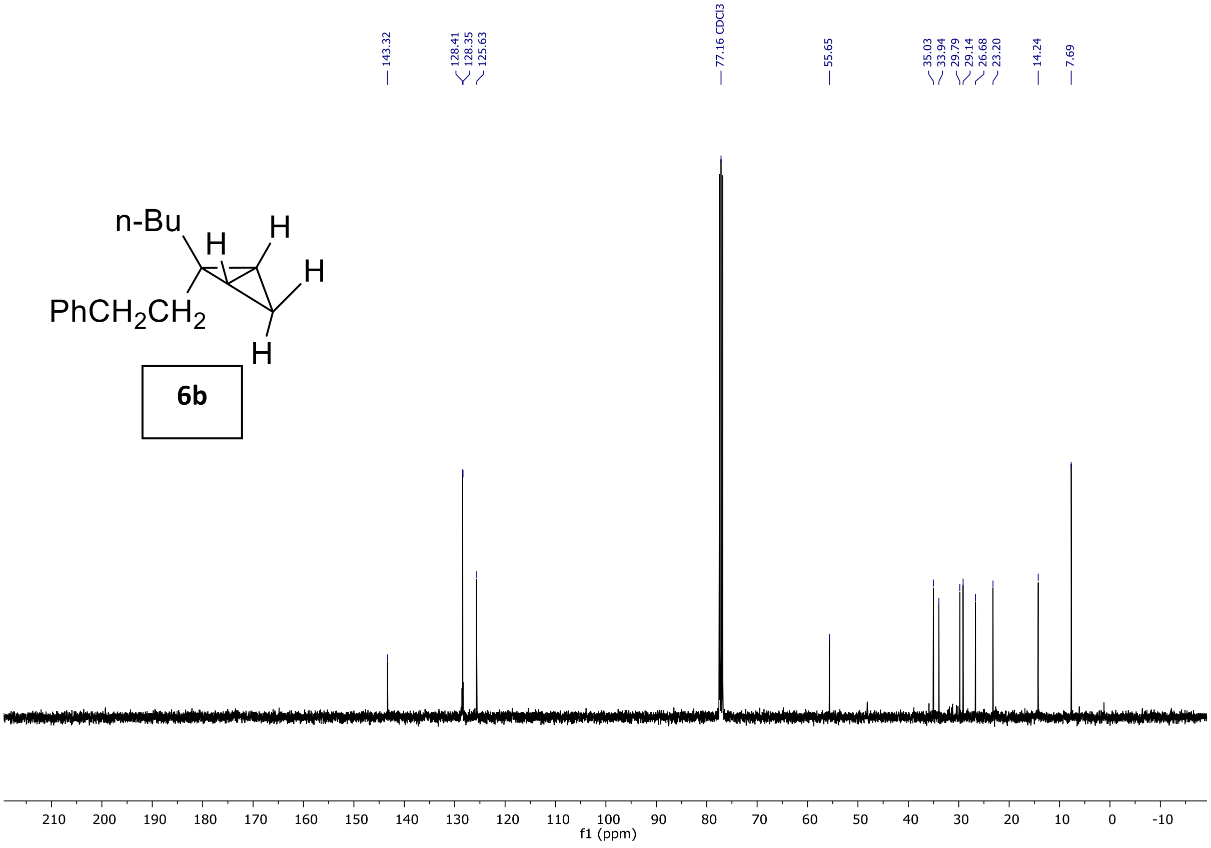
n-Bu
PhCH2CH2
6b
143.32
128.41
128.35
125.63
77.16 CDCl3
55.65
35.03
33.94
29.79
29.14
26.68
23.20
14.24
7.69
f1 (ppm)

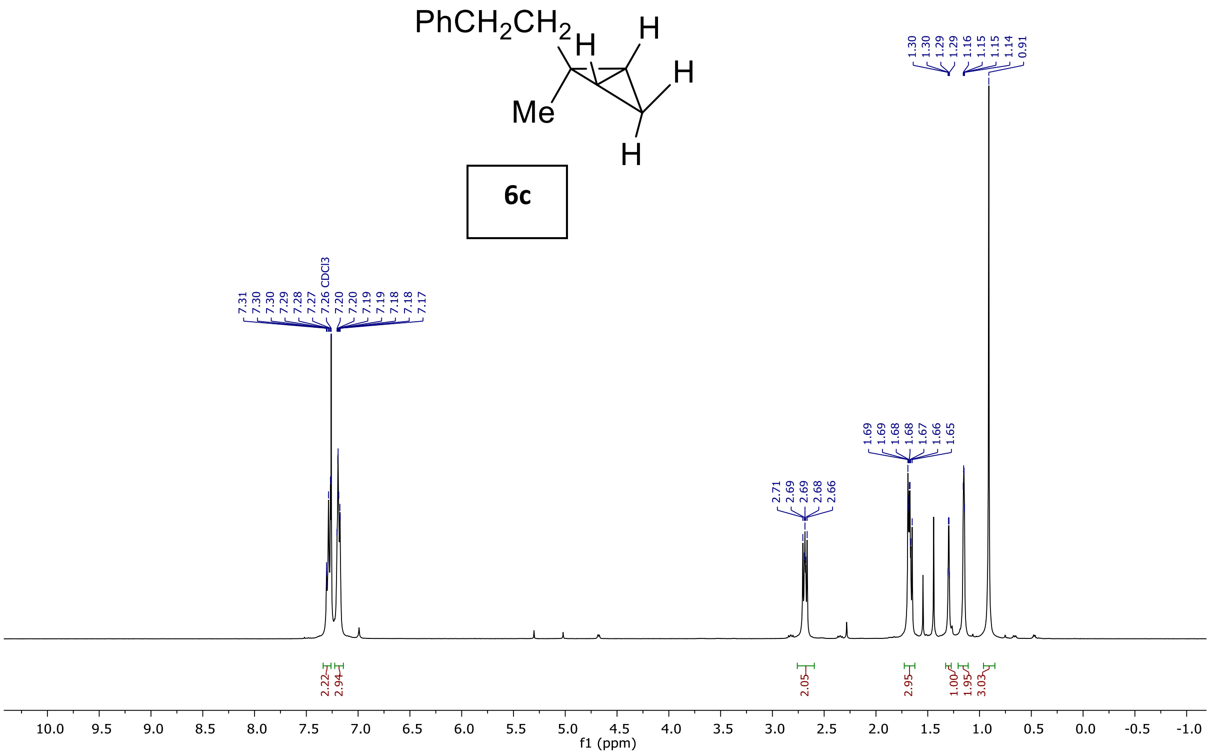
PhCH2CH2
H
H
H
Me
H
6c
7.31
7.30
7.30
7.29
7.28
7.27
7.26 CDCl3
7.20
7.20
7.20
7.19
7.19
7.18
7.18
7.17
2.71
2.69
2.69
2.68
2.66
1.69
1.69
1.68
1.68
1.67
1.66
1.65
1.30
1.30
1.29
1.29
1.16
1.15
1.15
1.14
0.91
2.22
2.94
2.05
2.95
1.00
1.95
3.03
f1 (ppm)

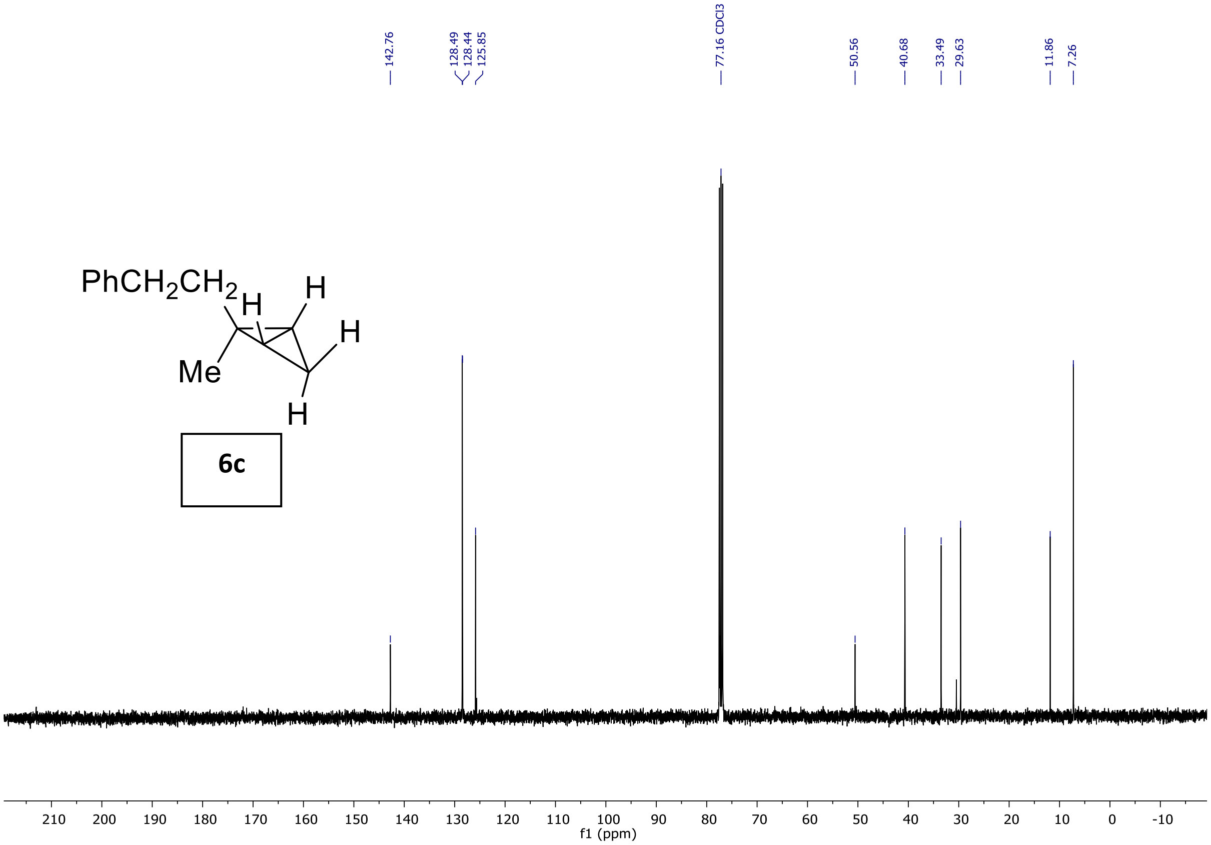
142.76
128.49
128.44
125.85
77.16 CDCl3
50.56
40.68
33.49
29.63
11.86
7.26
PhCH2CH2
H
H
H
Me
H
6c
f1 (ppm)

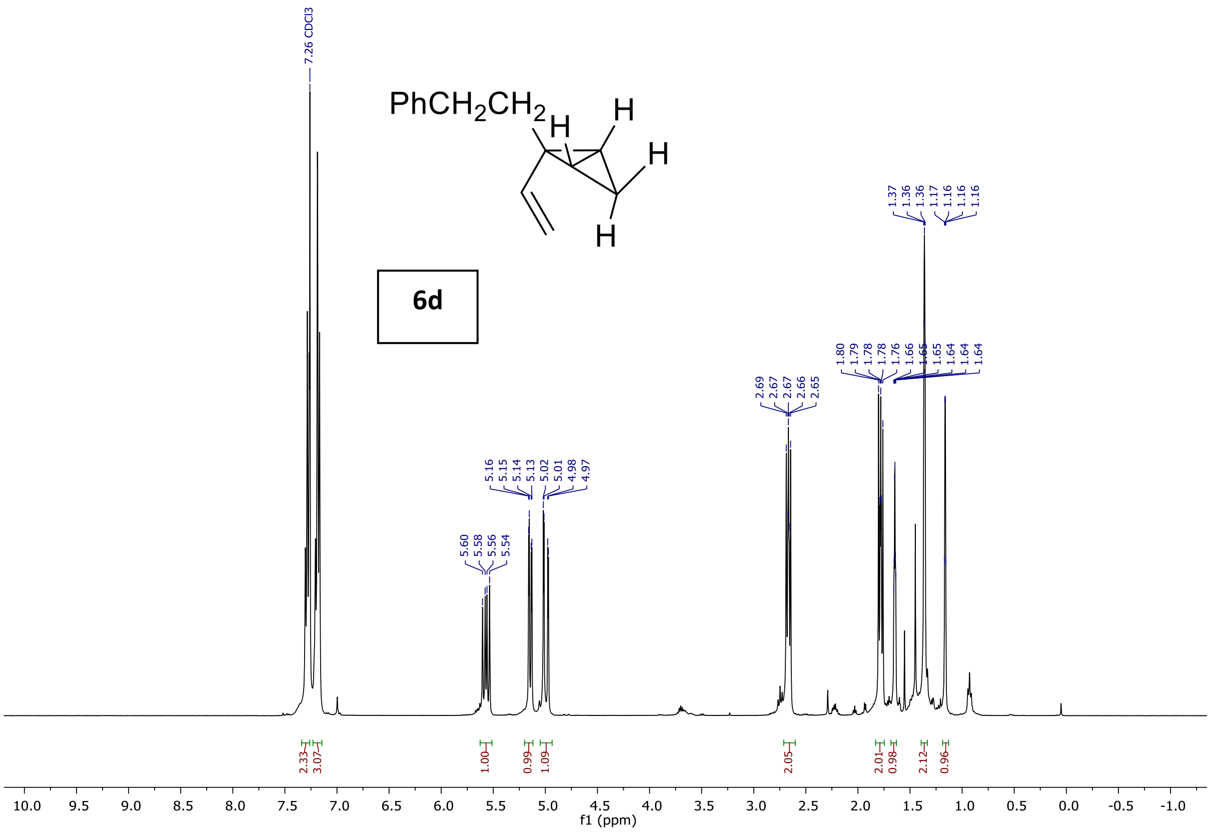
7.26 CDCl3
PhCH2CH2
H
H
H
H
6d
5.60
5.58
5.56
5.54
5.16
5.15
5.14
5.13
5.02
5.01
4.98
4.97
2.69
2.67
2.67
2.66
2.65
1.80
1.79
1.78
1.78
1.76
1.66
1.65
1.65
1.64
1.64
1.64
1.37
1.36
1.36
1.17
1.16
1.16
1.16
2.33
3.07
1.00
0.99
1.09
2.05
2.01
0.98
2.12
0.96
10.0
9.5
9.0
8.5
8.0
7.5
7.0
6.5
6.0
5.5
5.0
4.5
4.0
3.5
3.0
2.5
2.0
1.5
1.0
0.5
0.0
-0.5
-1.0
f1 (ppm)

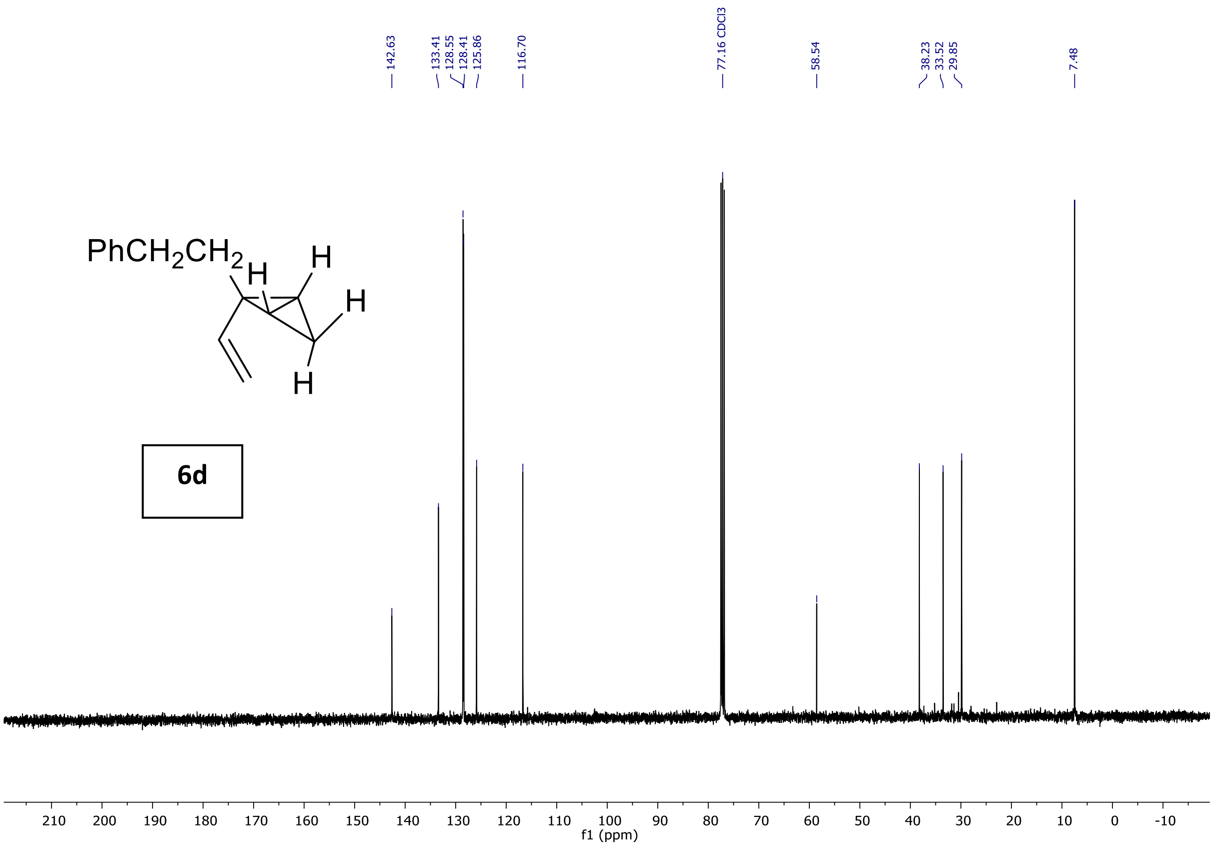
142.63
133.41
128.55
128.41
125.86
116.70
77.16 CDCl3
58.54
38.23
33.52
29.85
7.48
PhCH2CH2
H
H
H
H
6d
210
200
190
180
170
160
150
140
130
120
110
100
90
80
70
60
50
40
30
20
10
0
-10
f1 (ppm)

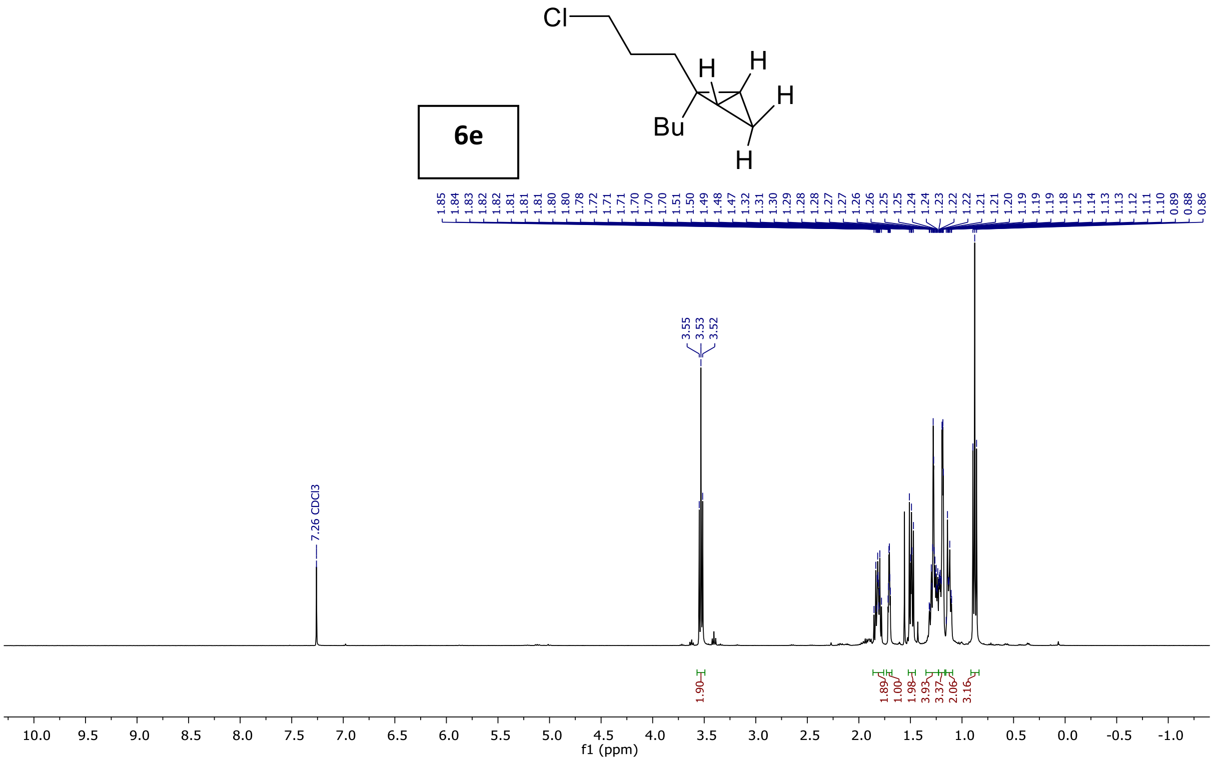
6e
Cl
H
H
H
Bu
H
7.26 CDCl3
3.55
3.53
3.52
1.90
1.89
1.00
1.98
3.93
3.37
2.06
3.16
f1 (ppm)

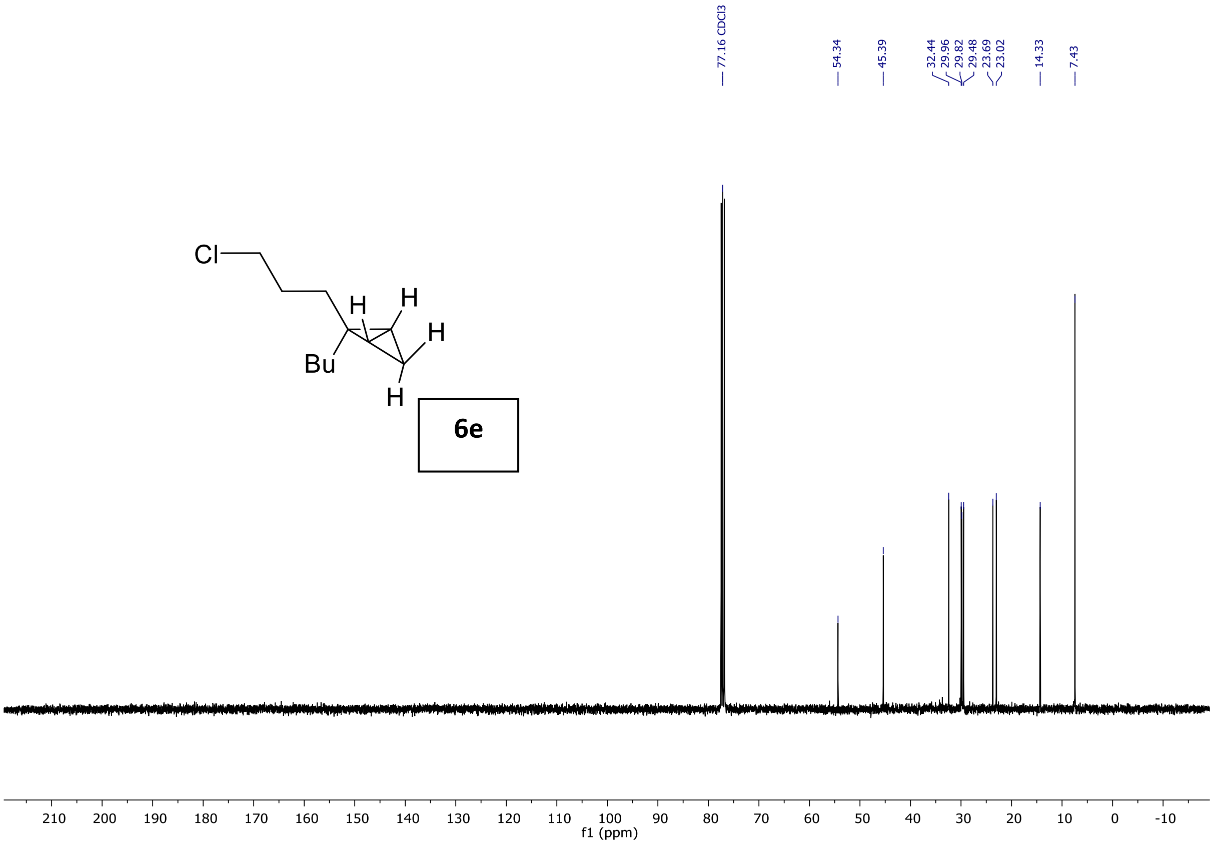
77.16 CDCl3
54.34
45.39
32.44
29.96
29.82
29.48
23.69
23.02
14.33
7.43
Cl
H
H
H
Bu
H
6e
f1 (ppm)

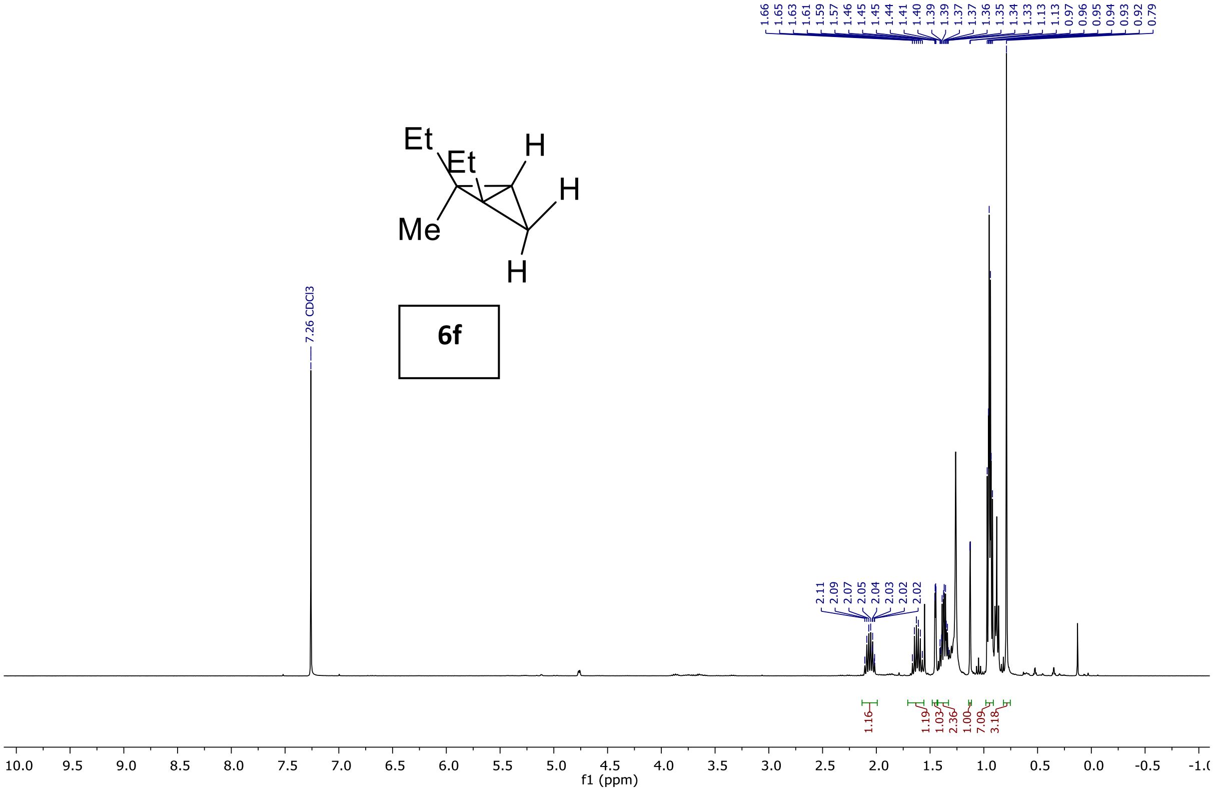

1.66
1.65
1.63
1.61
1.59
1.57
1.46
1.45
1.45
1.44
1.41
1.40
1.39
1.39
1.37
1.37
1.36
1.35
1.34
1.33
1.13
1.13
0.97
0.96
0.95
0.94
0.93
0.92
0.79
Et
Et
H
H
Me
H
6f
7.26 CDCl3
2.11
2.09
2.07
2.05
2.04
2.03
2.02
2.02
1.16
1.19
1.03
2.36
1.00
7.09
3.18
10.0 9.5 9.0 8.5 8.0 7.5 7.0 6.5 6.0 5.5 5.0 4.5 4.0 3.5 3.0 2.5 2.0 1.5 1.0 0.5 0.0 -0.5
f1 (ppm)

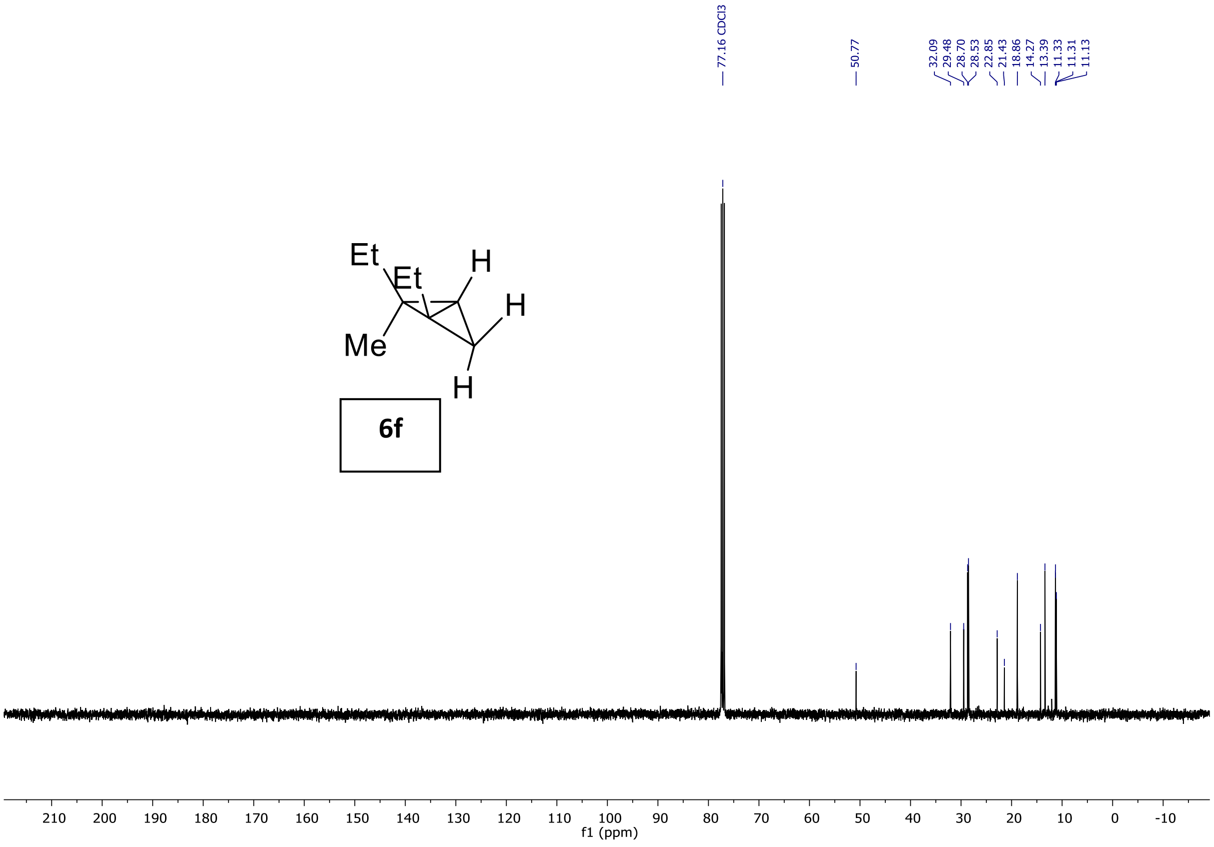

77.16 CDCl3
50.77
32.09
29.48
28.70
28.53
22.85
21.43
18.86
14.27
13.39
11.33
11.31
11.13
Et
Et
H
H
Me
H
6f
210 200 190 180 170 160 150 140 130 120 110 100 90 80 70 60 50 40 30 20 10 0 -10
f1 (ppm)

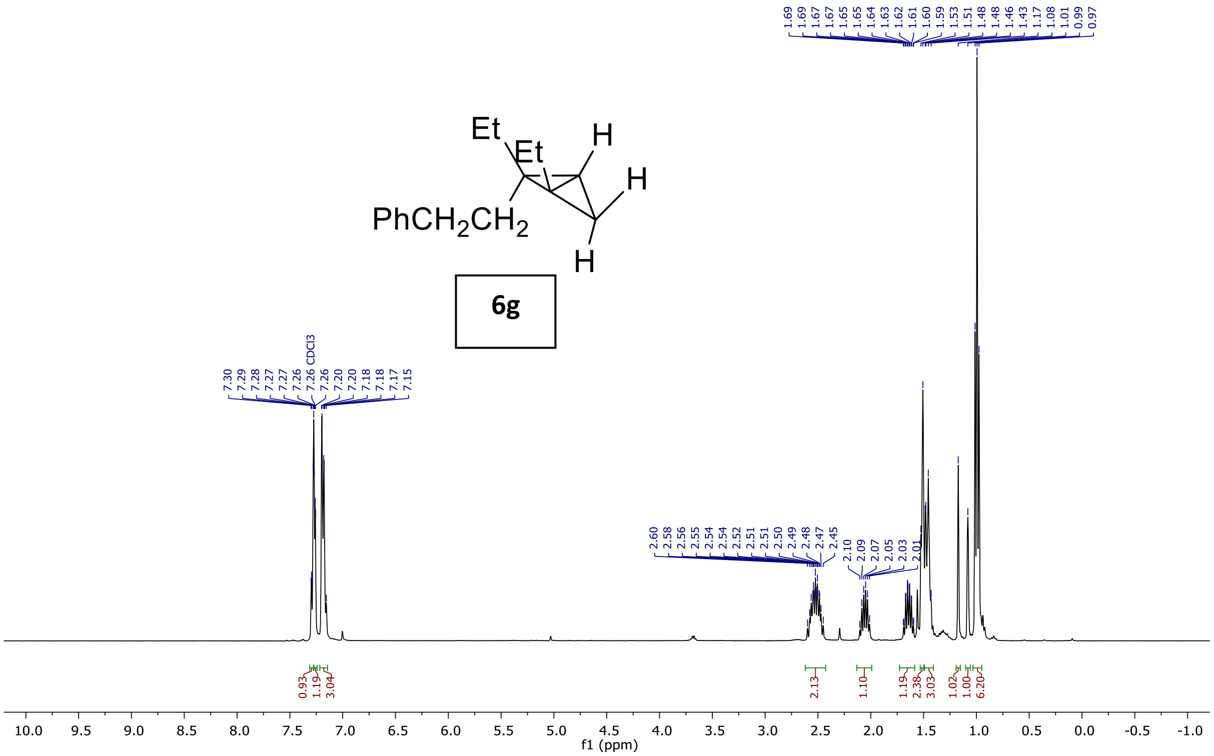
Et
Et
H
H
PhCH2CH2
H
6g
f1 (ppm)

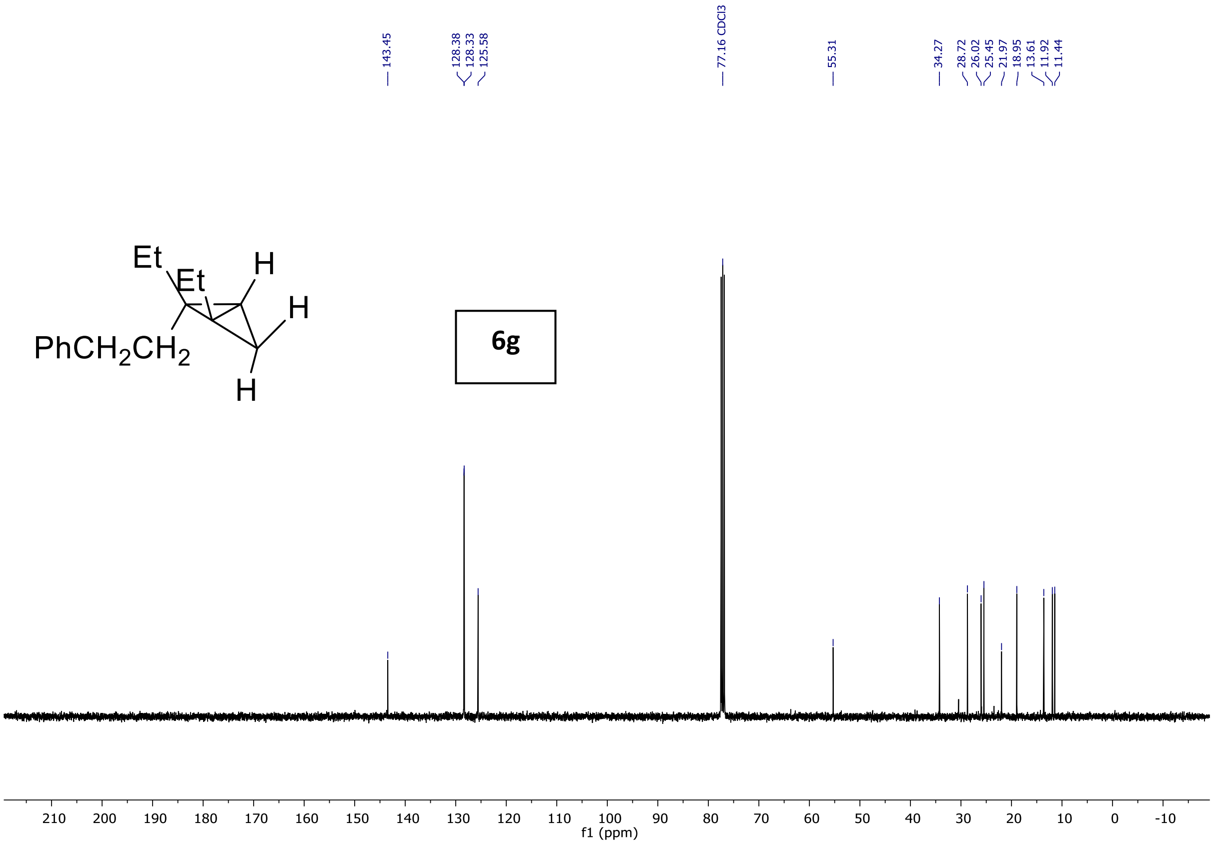
Et
Et
H
H
PhCH2CH2
H
6g
f1 (ppm)

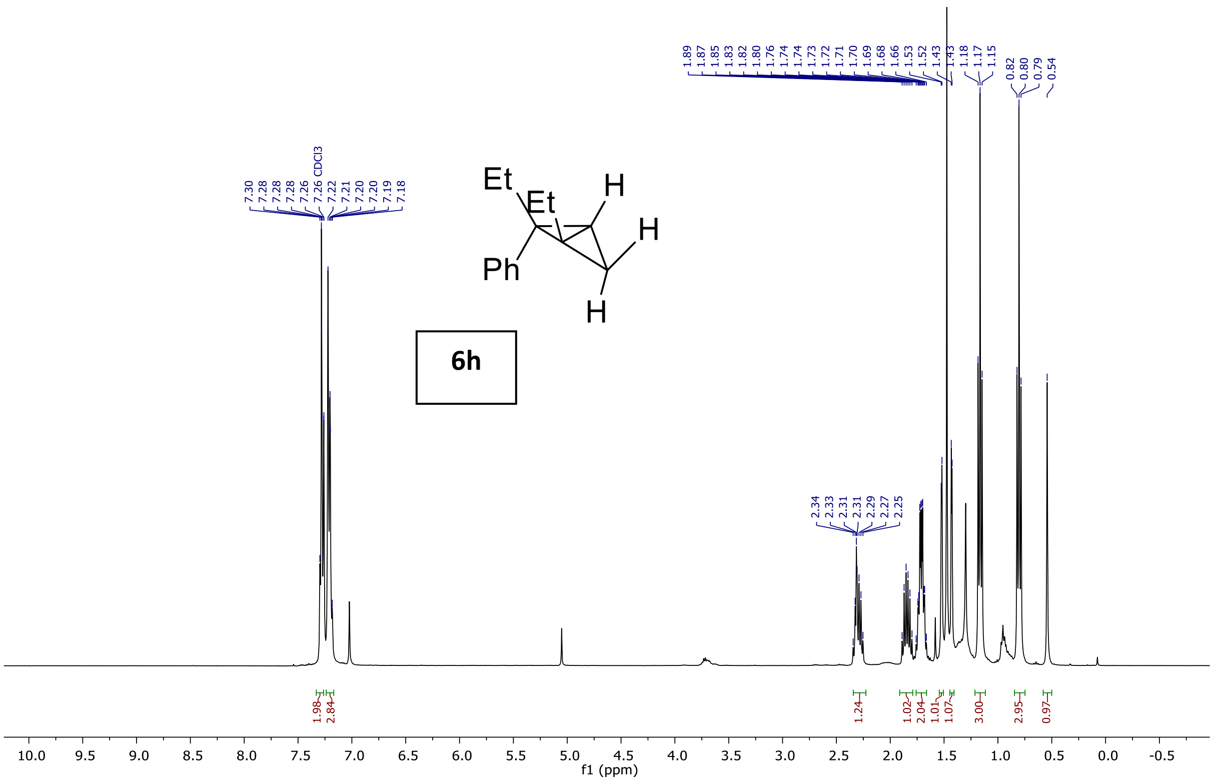

7.30
7.28
7.28
7.28
7.26
7.26 CDCl3
7.22
7.21
7.20
7.20
7.19
7.18
Et
Et
H
H
Ph
H
6h
1.89
1.87
1.85
1.83
1.82
1.80
1.76
1.74
1.74
1.73
1.72
1.71
1.70
1.69
1.68
1.66
1.53
1.52
1.43
1.43
1.18
1.17
1.15
0.82
0.80
0.79
0.54
2.34
2.33
2.31
2.31
2.29
2.27
2.25
1.98
2.84
1.24
1.02
2.04
1.01
1.07
3.00
2.95
0.97
10.0
9.5
9.0
8.5
8.0
7.5
7.0
6.5
6.0
5.5
5.0
4.5
4.0
3.5
3.0
2.5
2.0
1.5
1.0
0.5
0.0
-0.5
f1 (ppm)

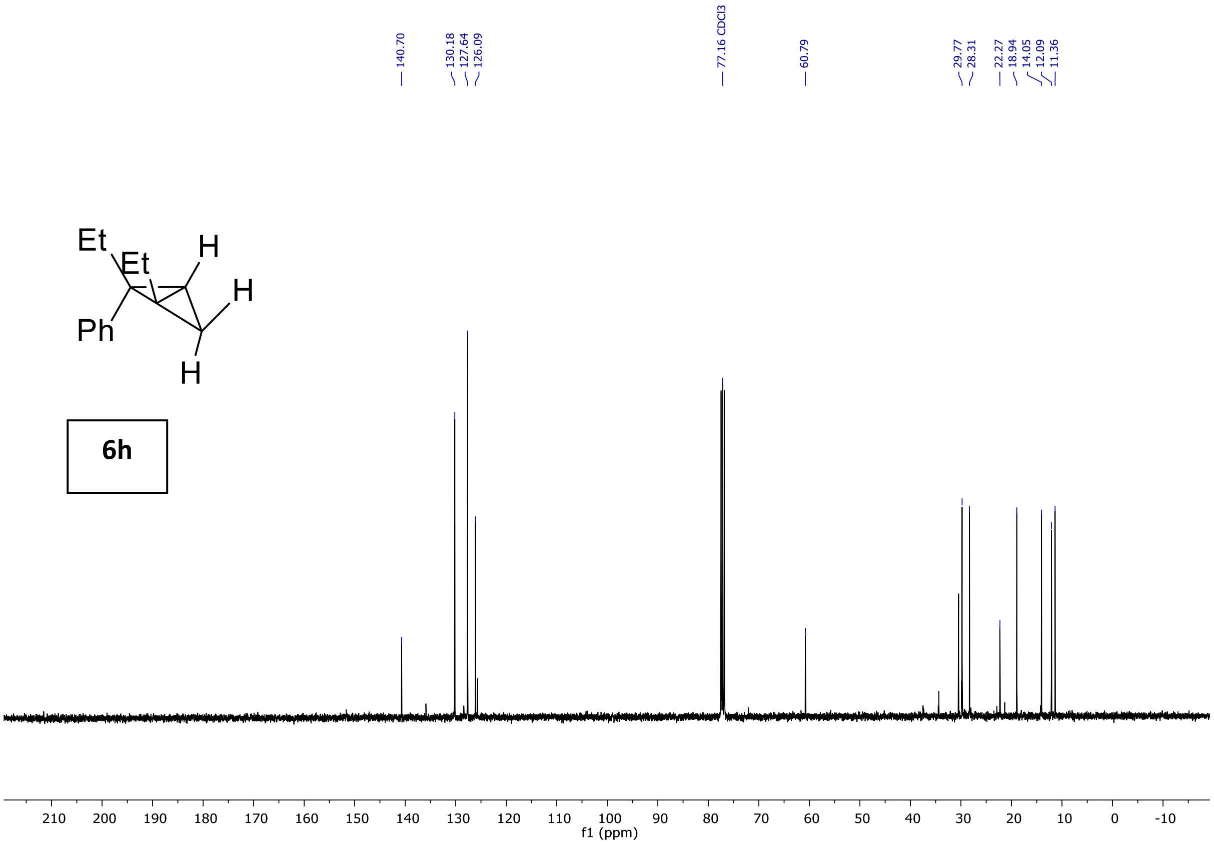

140.70
130.18
127.64
126.09
77.16 CDCl3
60.79
29.77
28.31
22.27
18.94
14.05
12.09
11.36
Et
Et
H
H
Ph
H
6h
210
200
190
180
170
160
150
140
130
120
110
100
90
80
70
60
50
40
30
20
10
0
-10
f1 (ppm)

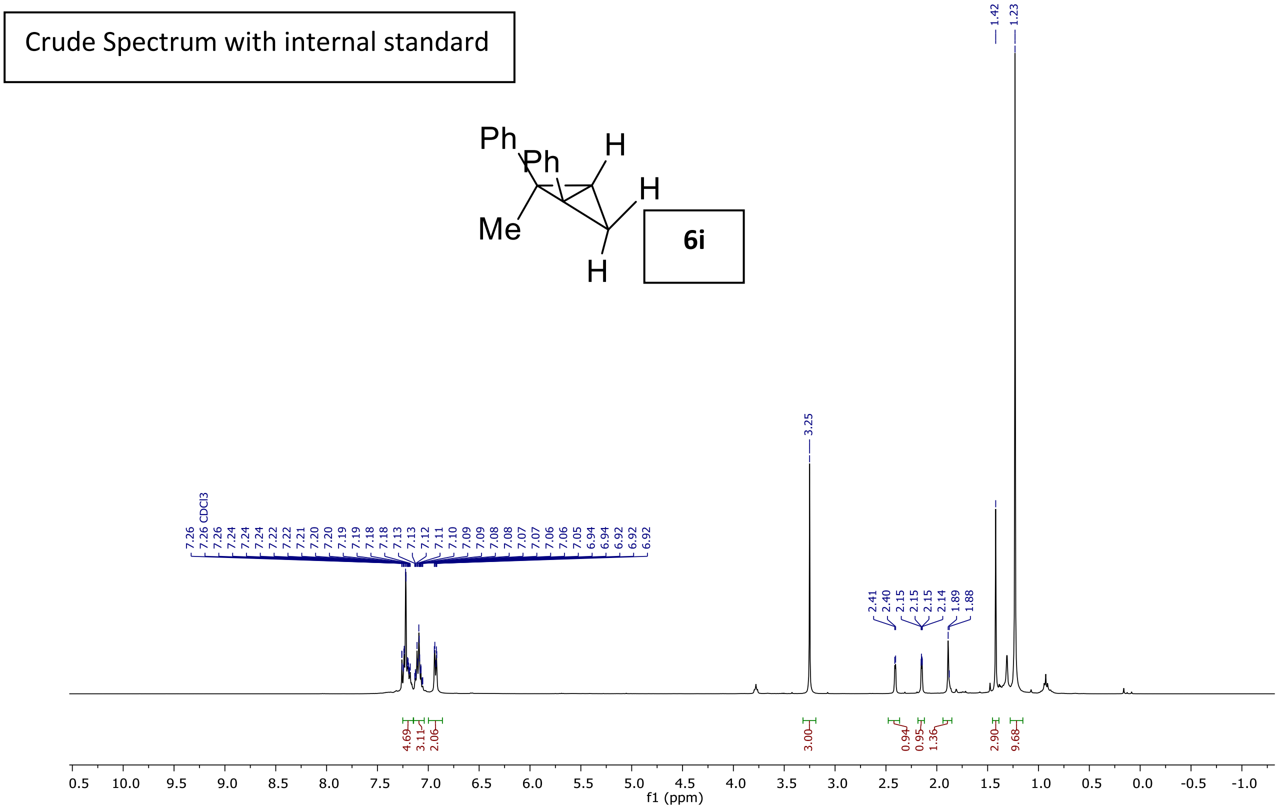

Crude Spectrum with internal standard
Ph
Ph
Me
H
H
H
6i
7.26
7.26 CDCl3
7.26
7.24
7.24
7.24
7.22
7.22
7.21
7.20
7.20
7.19
7.19
7.18
7.18
7.13
7.13
7.12
7.11
7.10
7.09
7.09
7.08
7.08
7.07
7.07
7.06
7.06
7.05
6.94
6.94
6.92
6.92
3.25
2.41
2.40
2.15
2.15
2.15
2.14
1.89
1.88
1.42
1.23
4.69
3.11
2.06
3.00
0.94
0.95
1.36
2.90
9.68
f1 (ppm)

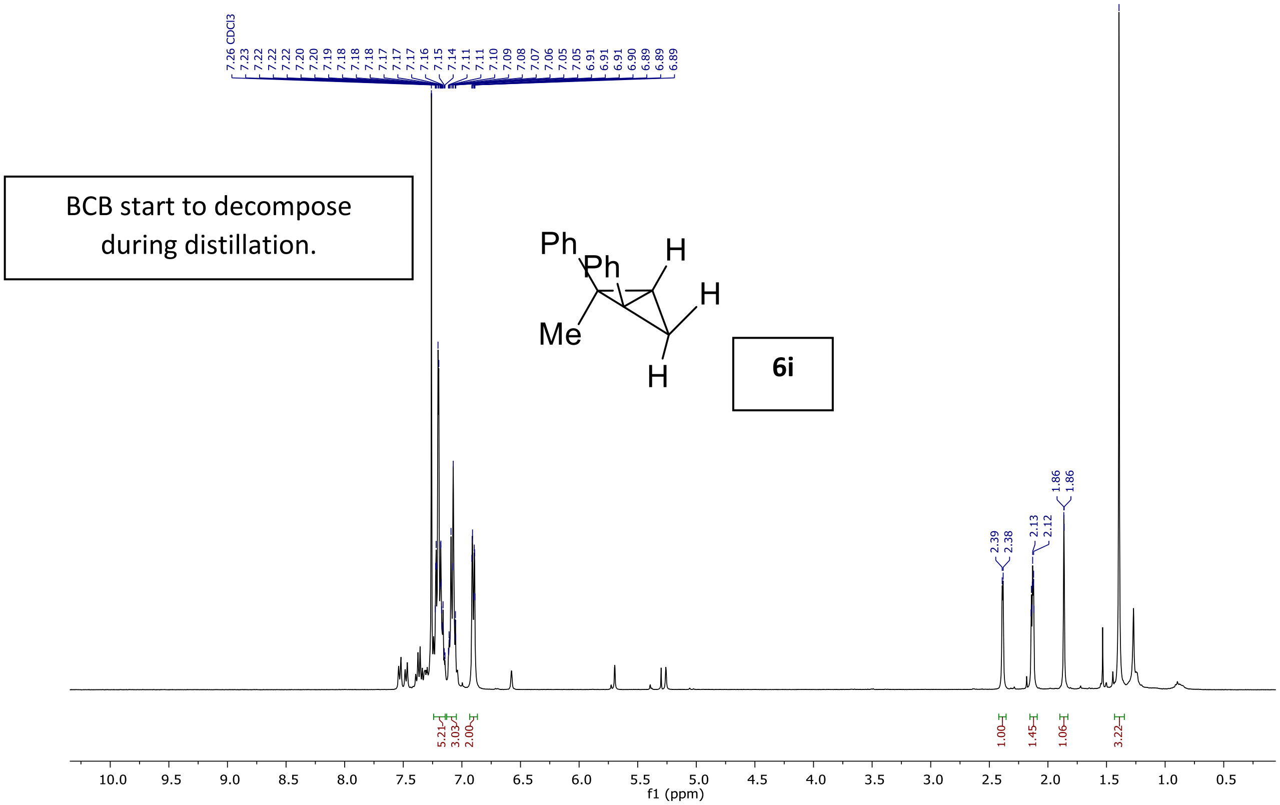
BCB start to decompose
during distillation.
6i
f1 (ppm)

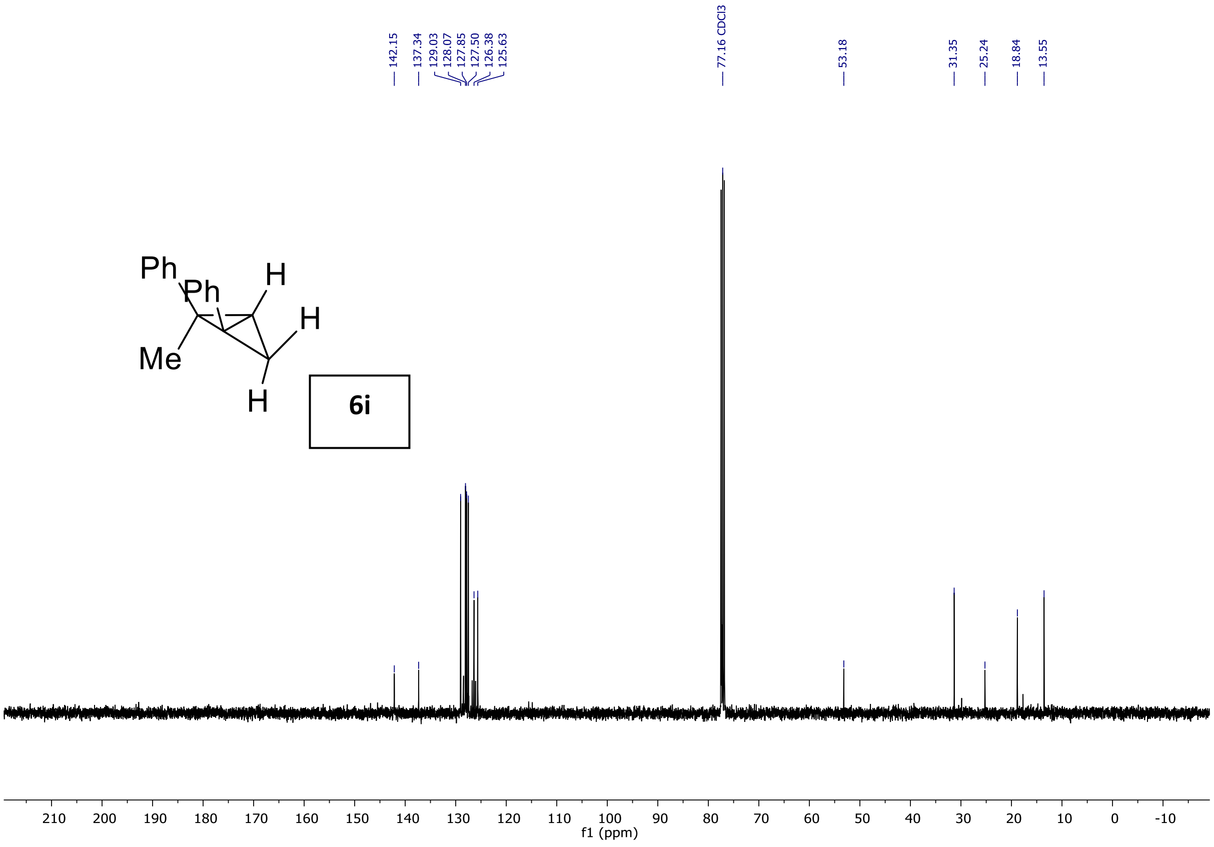
6i
f1 (ppm)

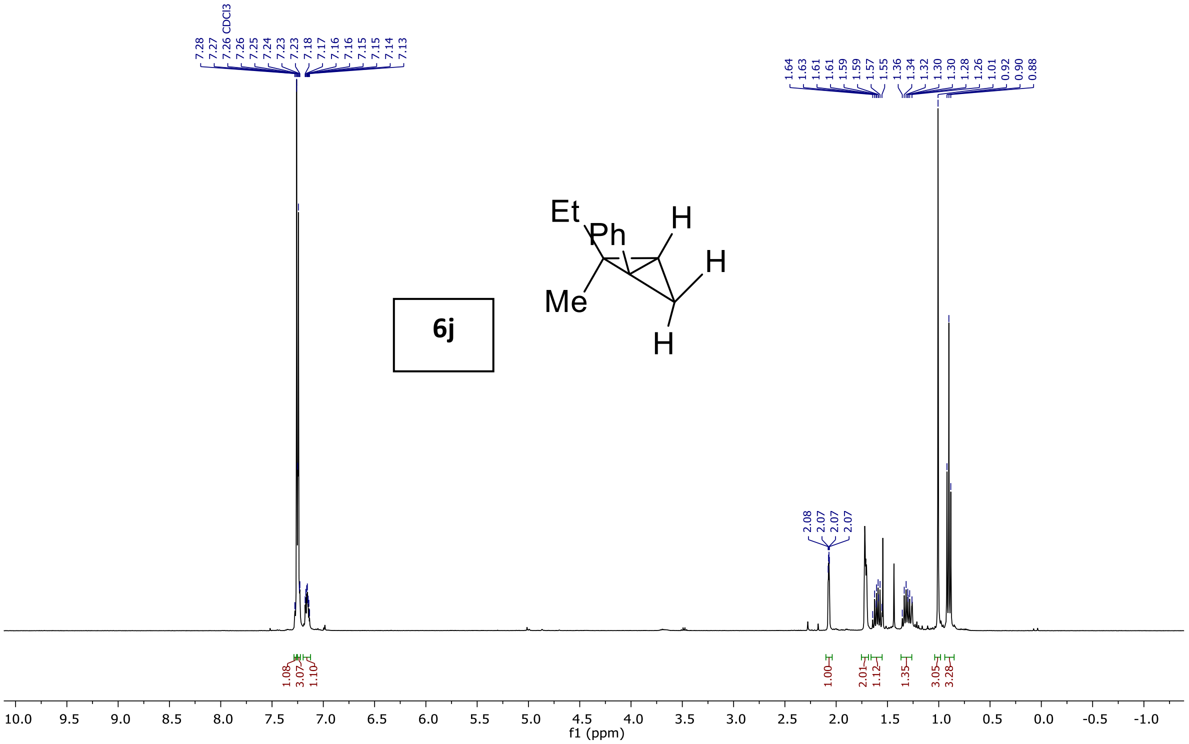

7.28
7.27
7.26 CDCl3
7.26
7.25
7.24
7.23
7.23
7.18
7.17
7.16
7.16
7.15
7.15
7.14
7.13
1.64
1.63
1.61
1.61
1.59
1.59
1.57
1.55
1.36
1.34
1.32
1.30
1.30
1.28
1.26
1.01
0.92
0.90
0.88
Et
Ph
H
H
Me
H
6j
2.08
2.07
2.07
2.07
1.08
3.07
1.10
1.00
2.01
1.12
1.35
3.05
3.28
f1 (ppm)

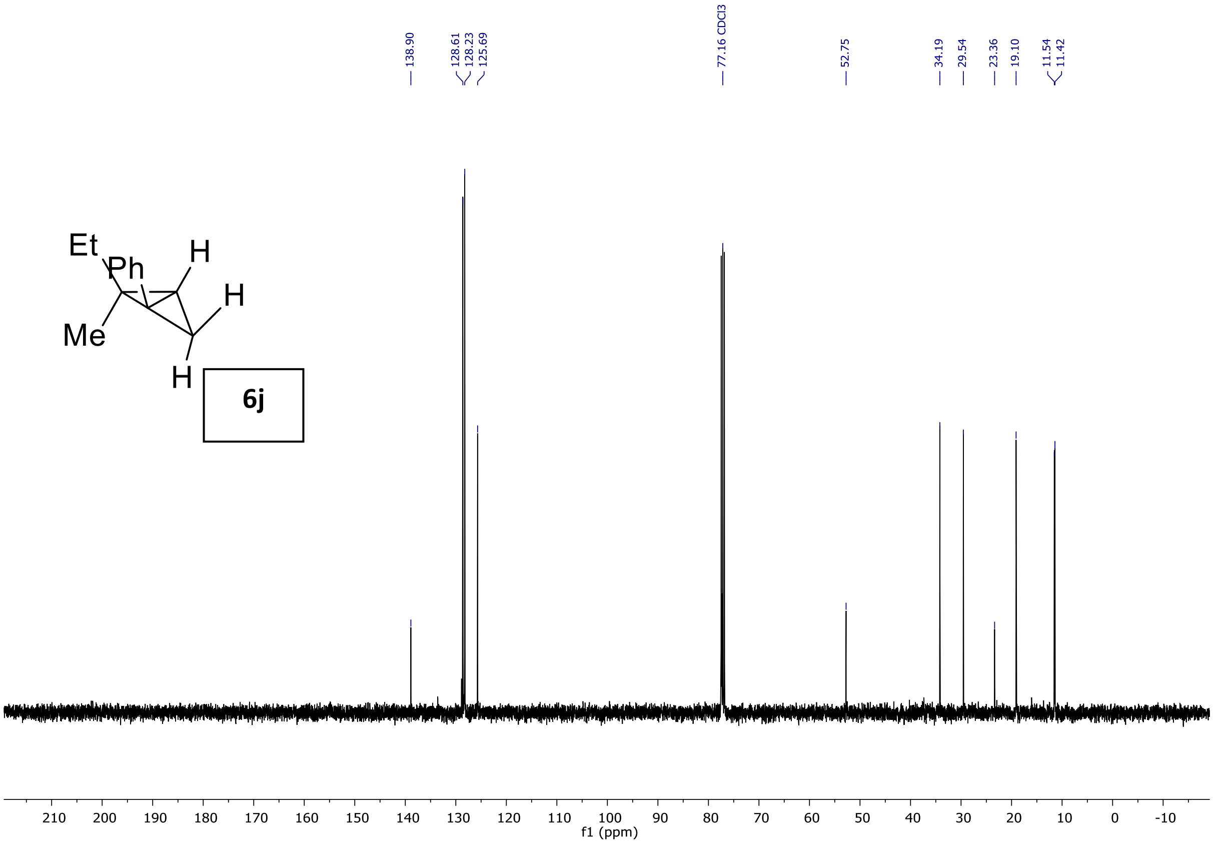

138.90
128.61
128.23
125.69
77.16 CDCl3
52.75
34.19
29.54
23.36
19.10
11.54
11.42
Et
Ph
H
H
Me
H
6j
f1 (ppm)

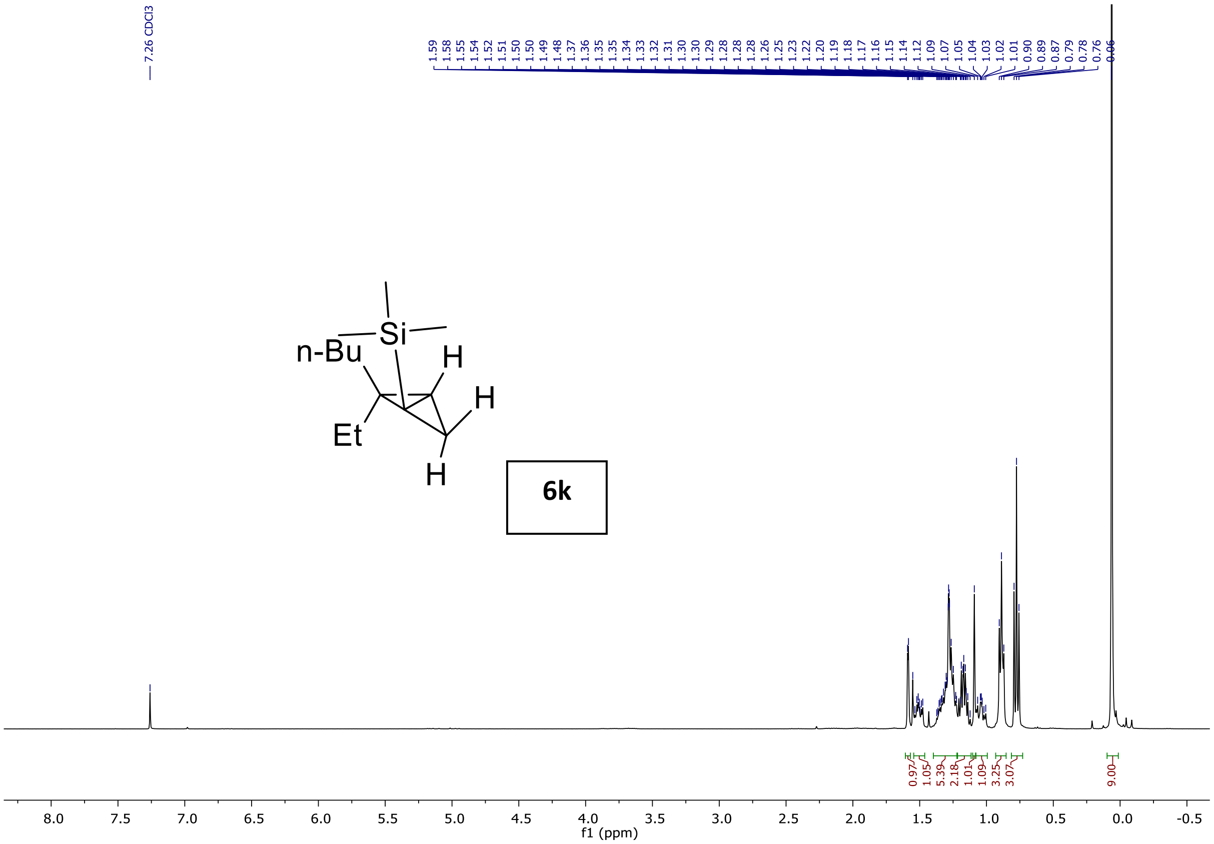

7.26 CDCl3
1.59 1.58 1.55 1.54 1.52 1.51 1.50 1.50 1.49 1.48 1.37 1.36 1.35 1.35 1.34 1.33 1.32 1.31 1.30 1.30 1.29 1.28 1.28 1.28 1.26 1.25 1.23 1.22 1.20 1.19 1.18 1.17 1.16 1.15 1.14 1.12 1.09 1.07 1.05 1.04 1.03 1.02 1.01 0.90 0.89 0.87 0.79 0.78 0.76 0.06
n-Bu
Si
Et
H
H
H
6k
0.97
1.05
5.39
2.18
1.01
1.09
3.25
3.07
9.00
8.0 7.5 7.0 6.5 6.0 5.5 5.0 4.5 4.0 3.5 3.0 2.5 2.0 1.5 1.0 0.5 0.0 -0.5
f1 (ppm)

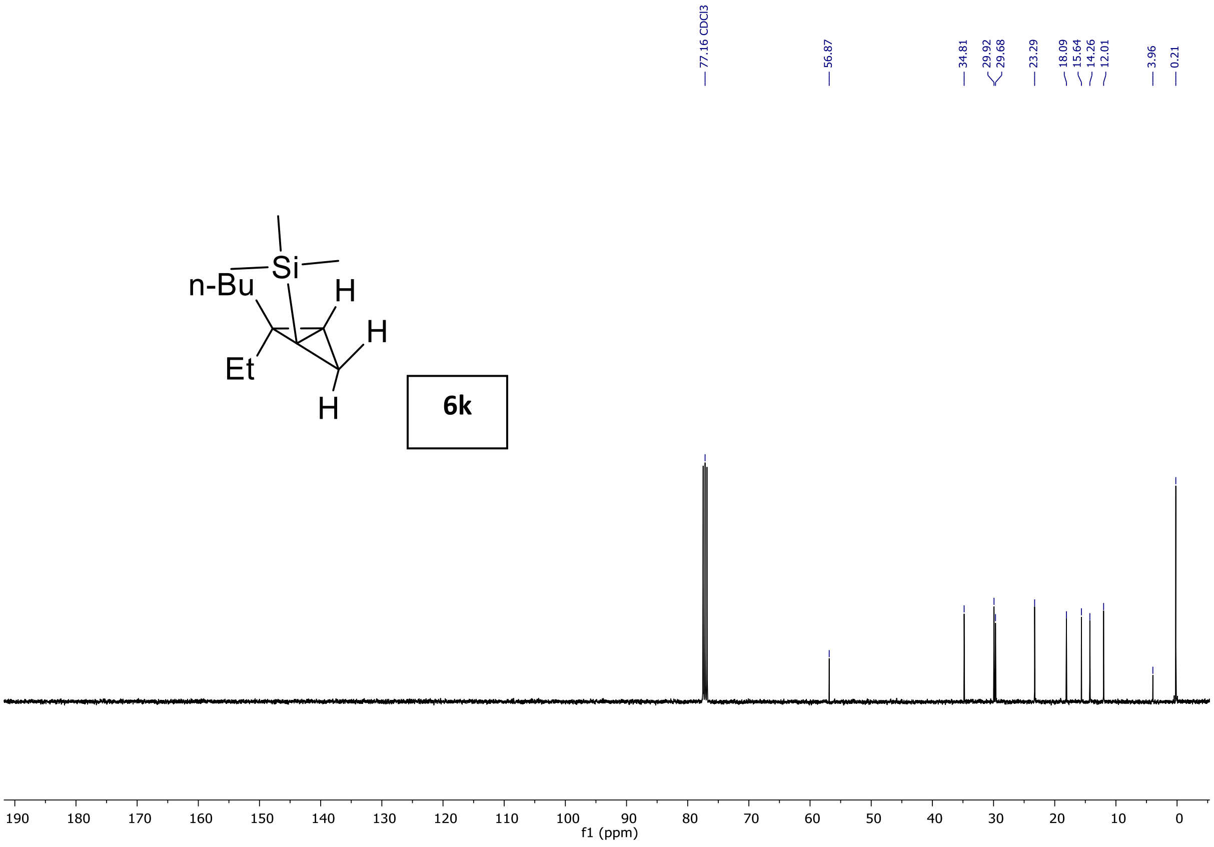

77.16 CDCl3
56.87
34.81
29.92
29.68
23.29
18.09
15.64
14.26
12.01
3.96
0.21
n-Bu
Si
Et
H
H
H
6k
190 180 170 160 150 140 130 120 110 100 90 80 70 60 50 40 30 20 10 0
f1 (ppm)

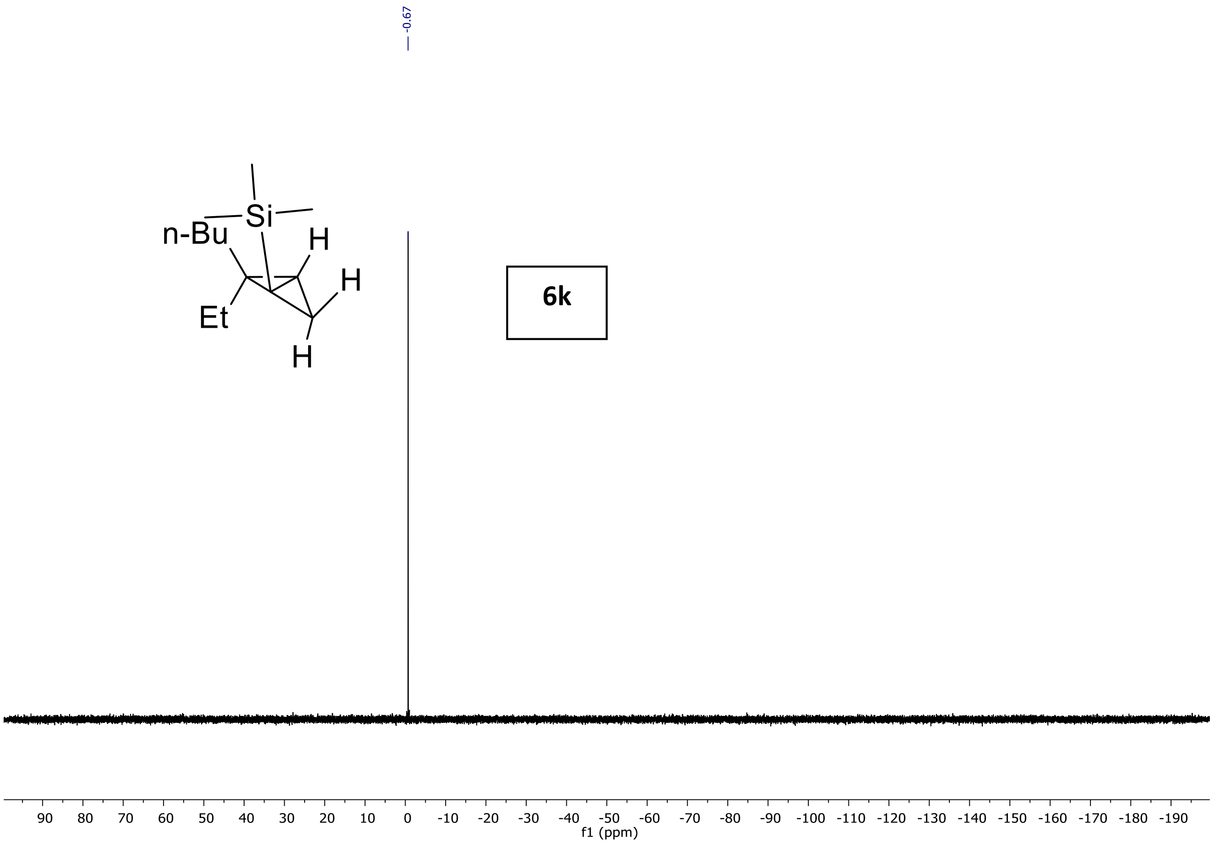

-0.67
n-Bu
Si
H
H
Et
H
6k
90 80 70 60 50 40 30 20 10 0 -10 -20 -30 -40 -50 -60 -70 -80 -90 -100 -110 -120 -130 -140 -150 -160 -170 -180 -190
f1 (ppm)

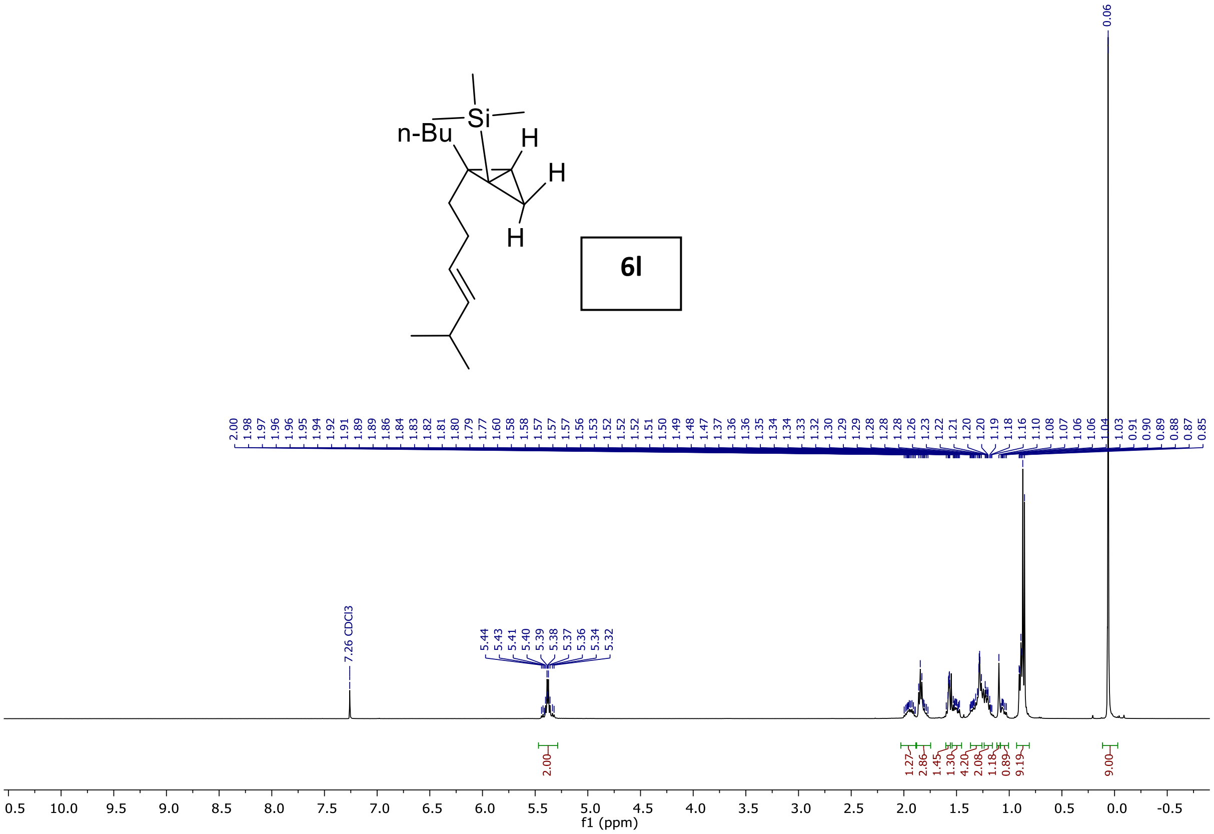
n-Bu
Si
H
H
H
6l
0.06
2.00 1.98 1.97 1.96 1.96 1.95 1.94 1.92 1.91 1.89 1.89 1.86 1.84 1.83 1.82 1.81 1.80 1.79 1.77 1.60 1.58 1.58 1.57 1.57 1.57 1.56 1.53 1.52 1.52 1.52 1.51 1.50 1.49 1.48 1.47 1.37 1.36 1.36 1.35 1.34 1.34 1.33 1.32 1.30 1.29 1.29 1.28 1.28 1.28 1.26 1.23 1.22 1.21 1.20 1.20 1.19 1.18 1.16 1.10 1.08 1.07 1.06 1.06 1.04 1.03 0.91 0.90 0.89 0.88 0.87 0.85
7.26 CDCl3
5.44 5.43 5.41 5.40 5.39 5.38 5.37 5.36 5.34 5.32
2.00
1.27 2.86 1.45 1.30 4.20 2.08 1.18 0.89 9.19
9.00
f1 (ppm)

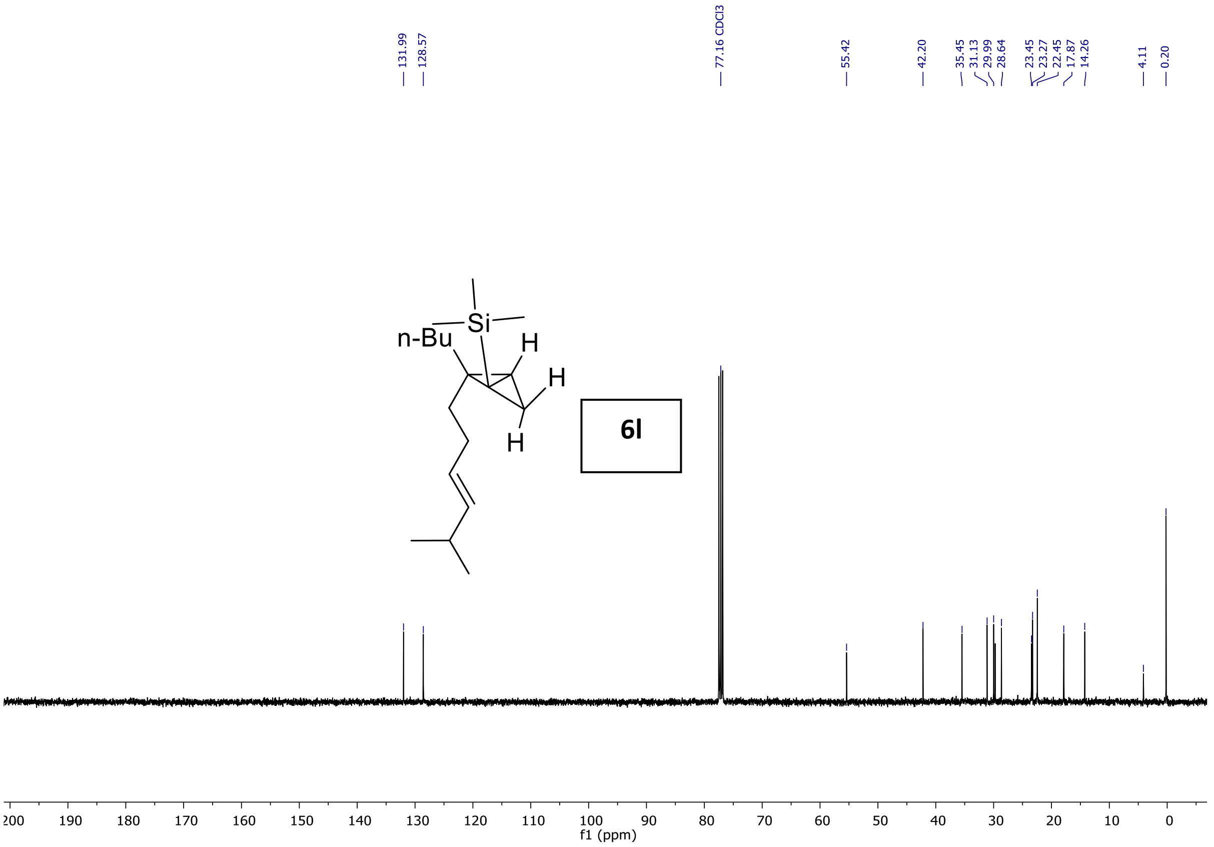
131.99
128.57
77.16 CDCl3
55.42
42.20
35.45 31.13 29.99 28.64
23.45 23.27 22.45 17.87 14.26
4.11
0.20
n-Bu
Si
H
H
H
6l
f1 (ppm)

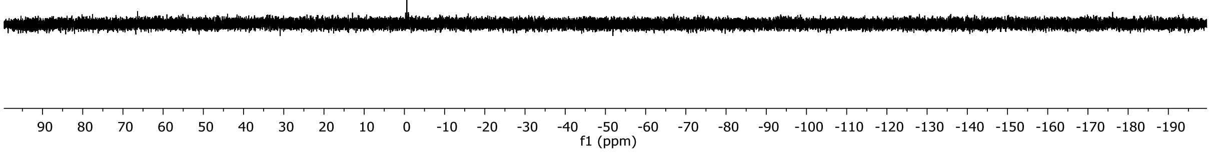

-0.61
90 80 70 60 50 40 30 20 10 0 -10 -20 -30 -40 -50 -60 -70 -80 -90 -100 -110 -120 -130 -140 -150 -160 -170 -180 -190
f1 (ppm)

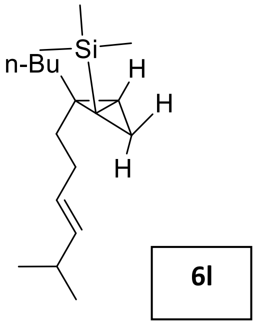

n-Bu
Si
H
H
H

6l

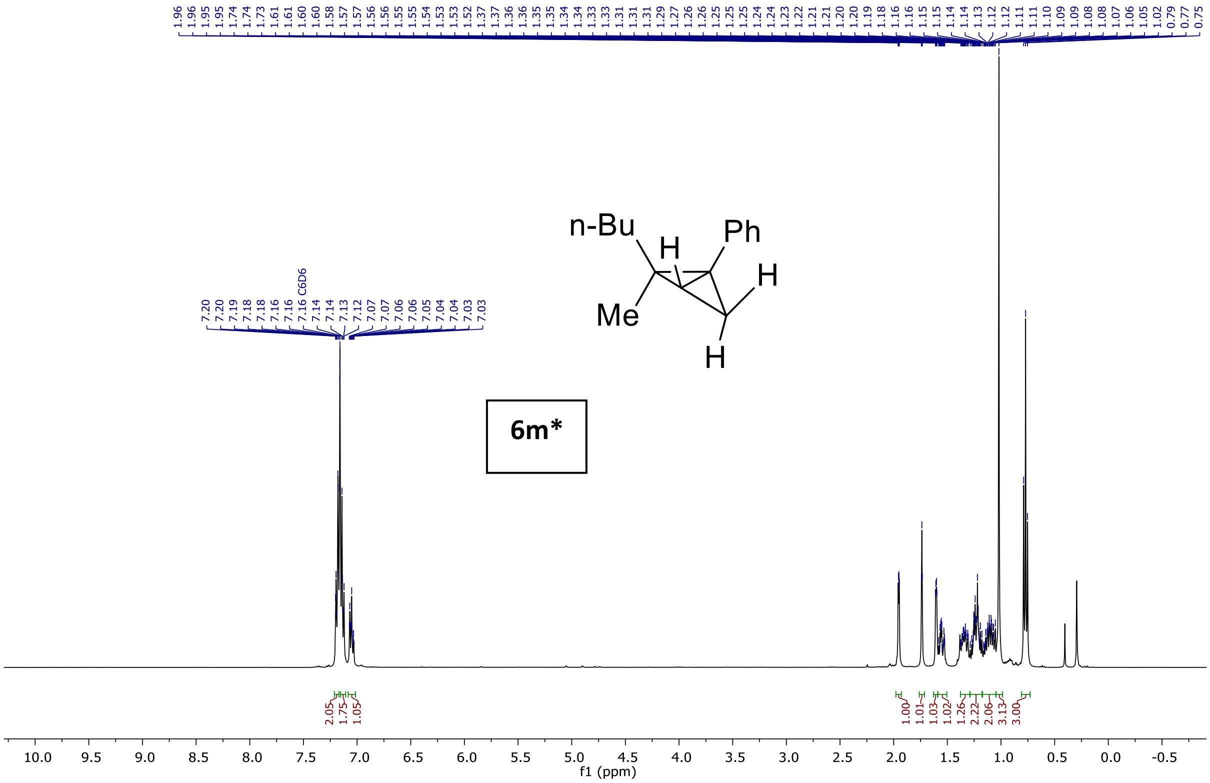
1.96 1.96 1.95 1.95 1.74 1.74 1.73 1.61 1.61 1.60 1.60 1.58 1.57 1.57 1.56 1.56 1.55 1.55 1.54 1.53 1.53 1.52 1.37 1.37 1.36 1.36 1.35 1.35 1.34 1.34 1.33 1.33 1.31 1.31 1.29 1.27 1.26 1.26 1.25 1.25 1.25 1.24 1.24 1.23 1.22 1.21 1.21 1.20 1.20 1.19 1.18 1.16 1.16 1.15 1.15 1.14 1.14 1.13 1.13 1.12 1.12 1.11 1.11 1.10 1.09 1.09 1.08 1.08 1.07 1.06 1.05 1.02 0.79 0.75
7.20 7.20 7.19 7.18 7.18 7.16 7.16 7.16 C6D6 7.14 7.14 7.13 7.12 7.07 7.07 7.06 7.06 7.05 7.04 7.04 7.03 7.03
n-Bu
H
Ph
H
Me
H
6m*
2.05 1.75 1.05
1.00 1.01 1.03 1.02 1.26 2.22 2.06 3.13 3.00
10.0 9.5 9.0 8.5 8.0 7.5 7.0 6.5 6.0 5.5 5.0 4.5 4.0 3.5 3.0 2.5 2.0 1.5 1.0 0.5 0.0 -0.5
f1 (ppm)

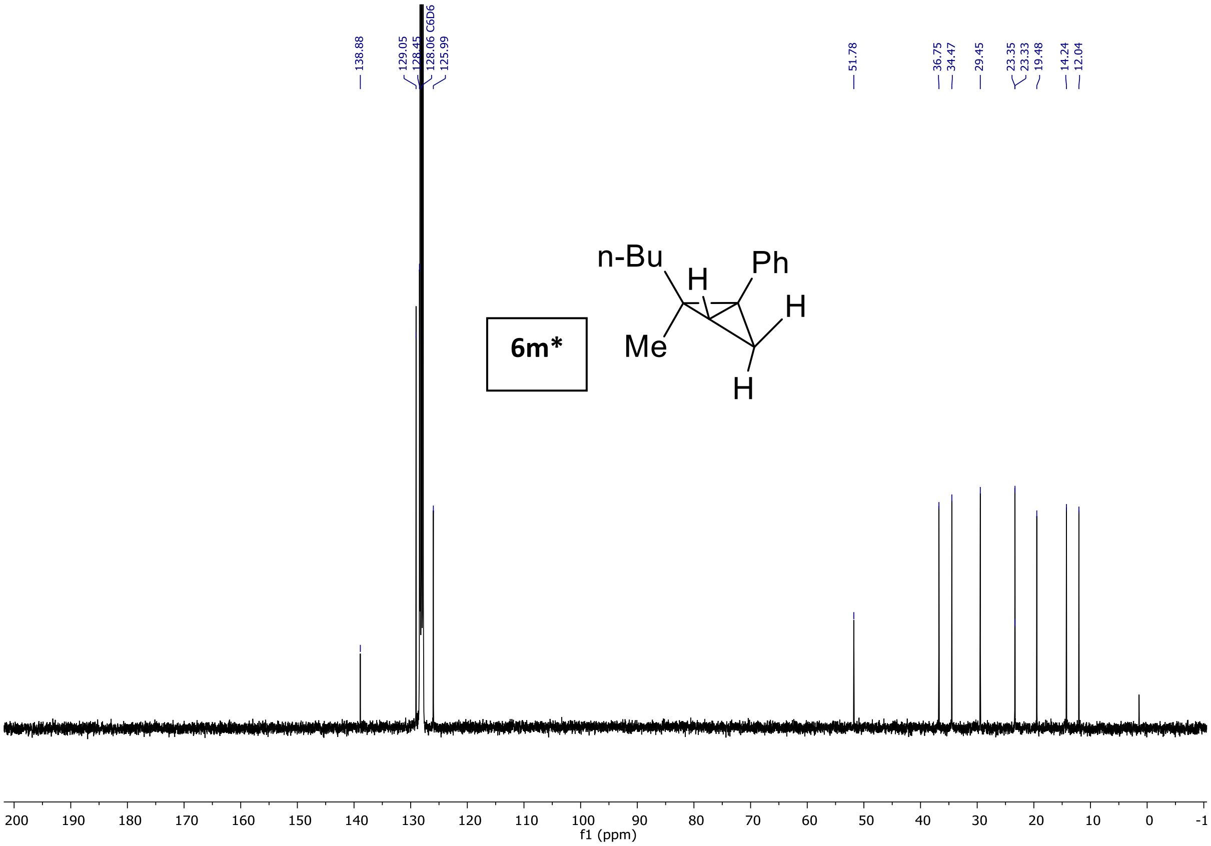
138.88
129.05 128.45 128.06 C6D6 125.99
51.78
36.75 34.47 29.45 23.35 23.33 19.48 14.24 12.04
n-Bu
H
Ph
H
Me
H
6m*
200 190 180 170 160 150 140 130 120 110 100 90 80 70 60 50 40 30 20 10 0 -1
f1 (ppm)

HPLC chromatogram of racemic compound (6m)

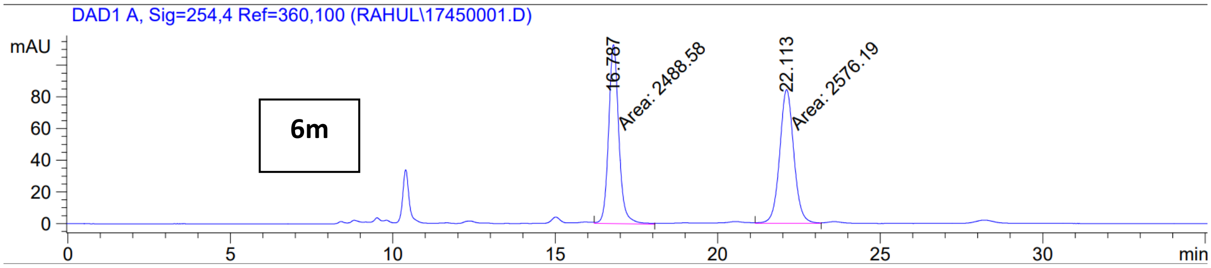

Signal 1: DAD1 A, Sig=254,4 Ref=360,100

Peak #	RetTime [min]	Type	Width [min]	Area [mAU*s]	Height [mAU]	Area %
1	16.787	MM	0.3664	2488.57886	113.19654	49.1351
2	22.113	MM	0.5070	2576.18677	84.68488	50.8649

Totals : 5064.76563 197.88142

CHIRACEL OD column, 1cmI.D x 25 cmL, (n-Hexane, 1.5 mL/min, 20 °C, 254.4 nm)

HPLC chromatogram of enantioenriched sample (6m*)

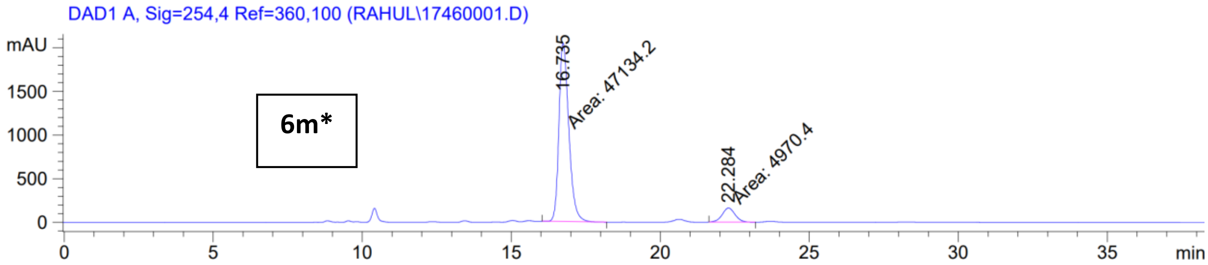

Signal 1: DAD1 A, Sig=254,4 Ref=360,100

Peak #	RetTime [min]	Type	Width [min]	Area [mAU*s]	Height [mAU]	Area %
1	16.735	MM	0.3844	4.71342e4	2043.61011	90.4607
2	22.284	MM	0.5077	4970.39844	163.18129	9.5393

Totals : 5.21046e4 2206.79140

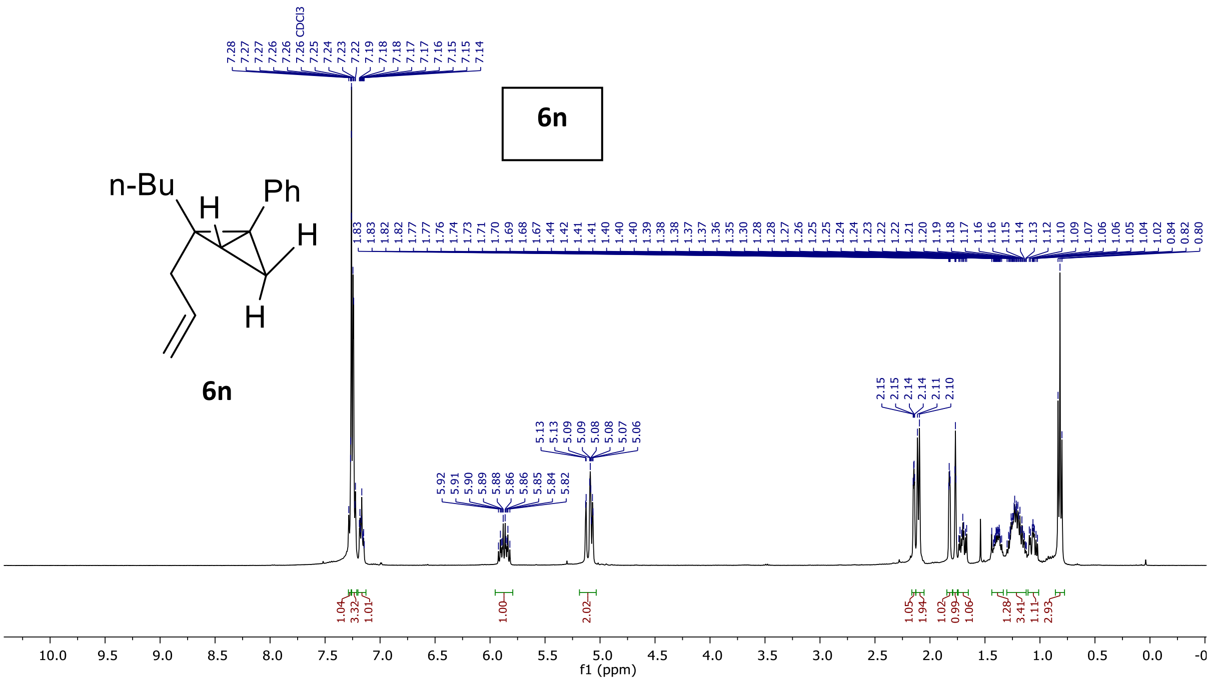
6n
n-Bu
Ph
H
H
H
6n
f1 (ppm)

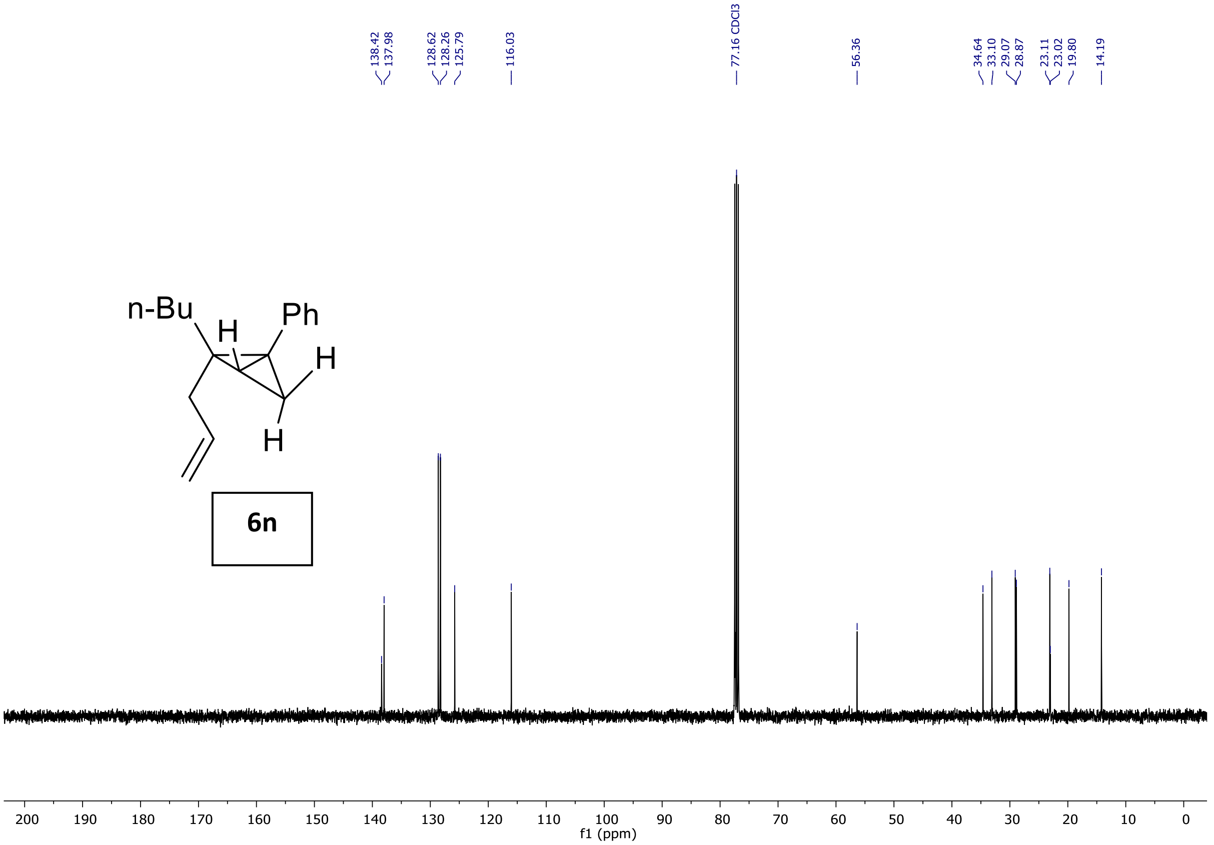
138.42
137.98
128.62
128.26
125.79
116.03
77.16 CDCl3
56.36
34.64
33.10
29.07
28.87
23.11
23.02
19.80
14.19
n-Bu
Ph
H
H
H
6n
f1 (ppm)

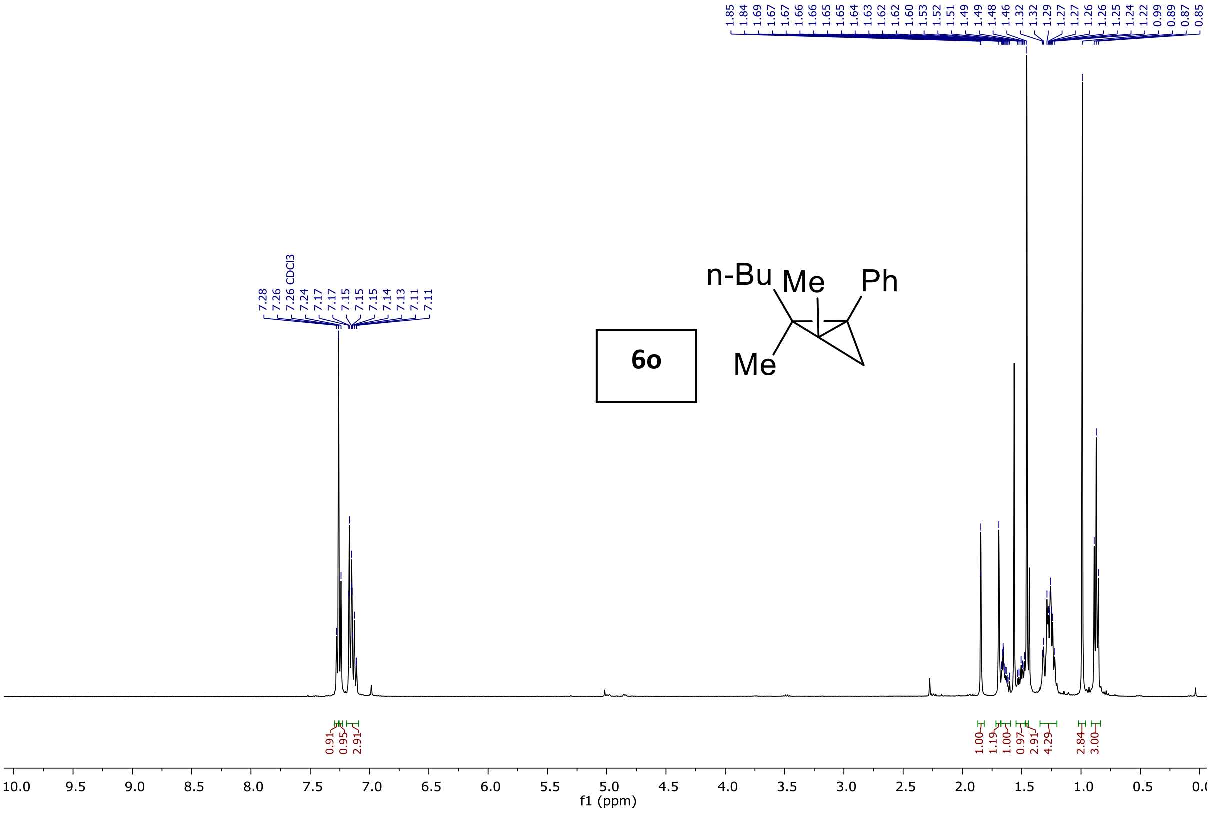
6o
n-Bu
Me
Ph
Me
f1 (ppm)

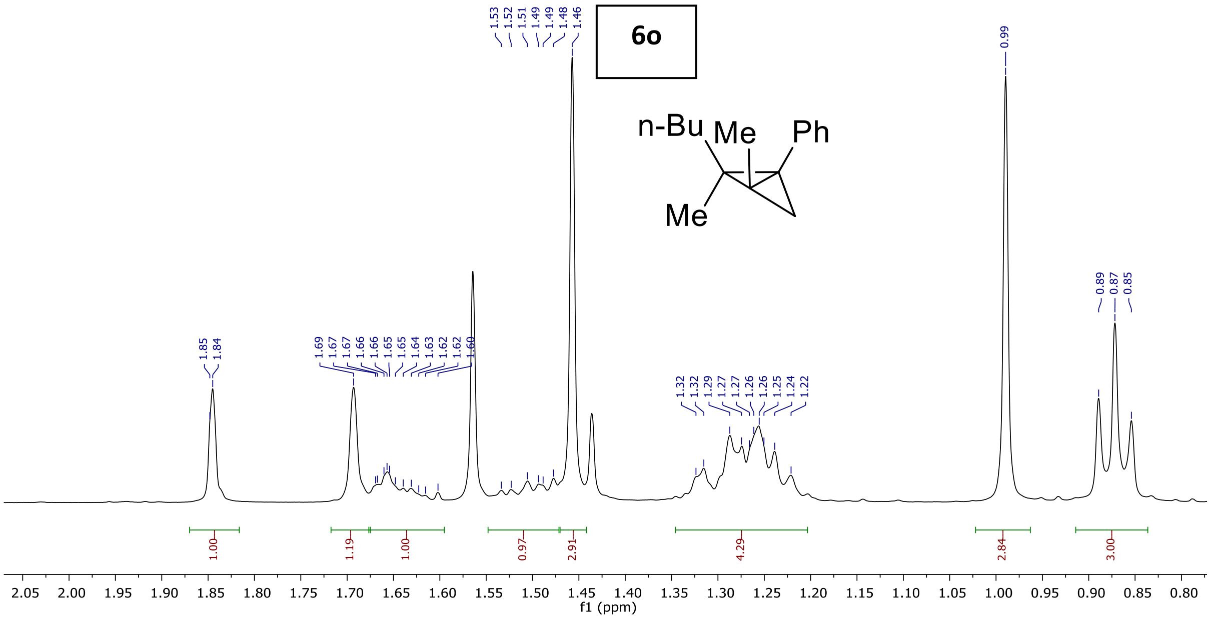
6o
n-Bu
Me
Ph
Me
f1 (ppm)

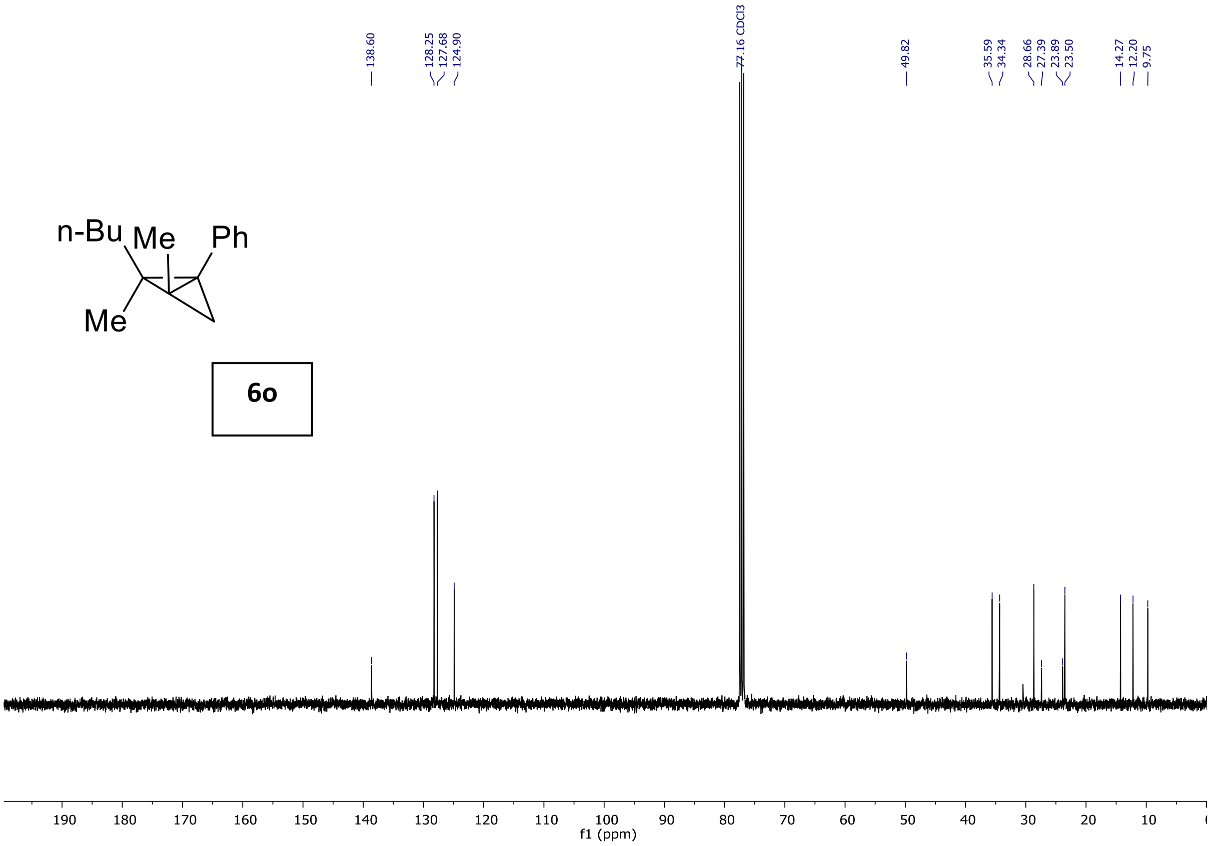
n-Bu
Me
Ph
Me
138.60
128.25
127.68
124.90
77.16 CDCl3
49.82
35.59
34.34
28.66
27.39
23.89
23.50
14.27
12.20
9.75
190 180 170 160 150 140 130 120 110 100 90 80 70 60 50 40 30 20 10
f1 (ppm)

6o

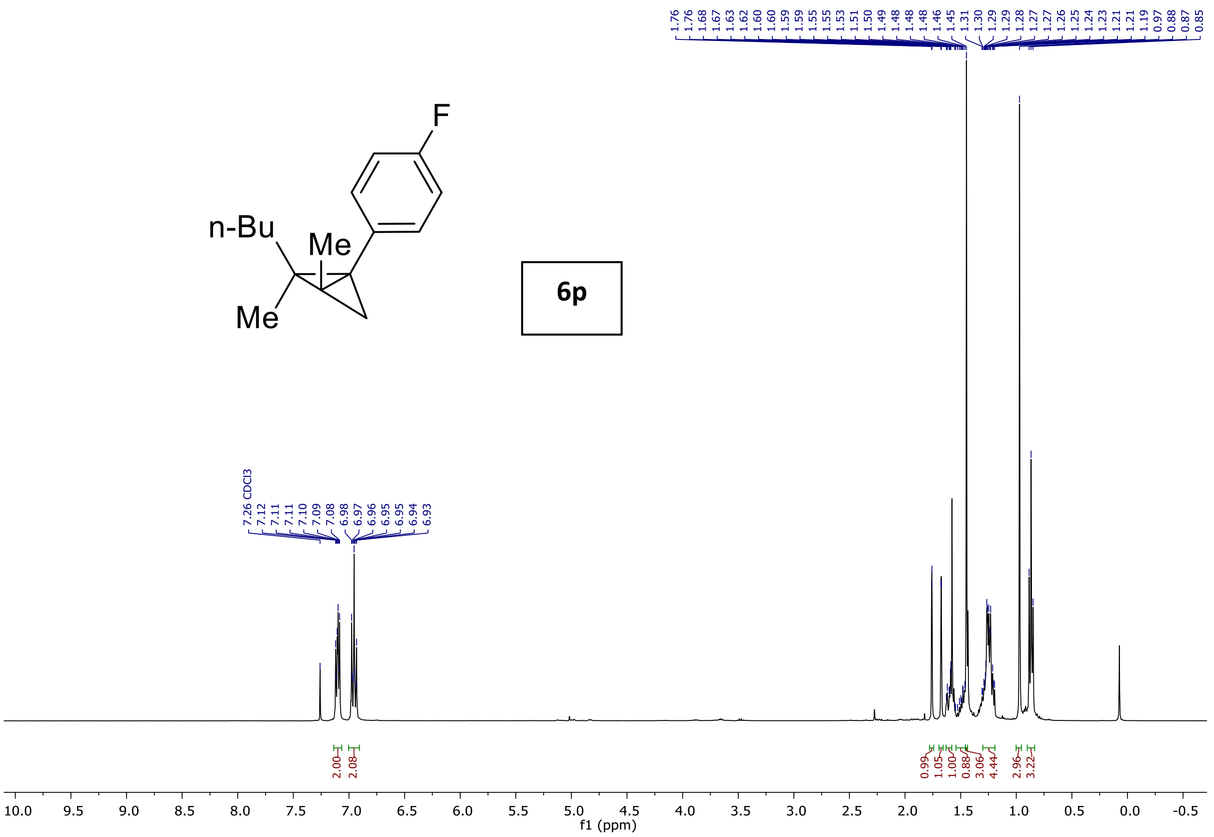
6p
n-Bu
Me
Me
F
f1 (ppm)

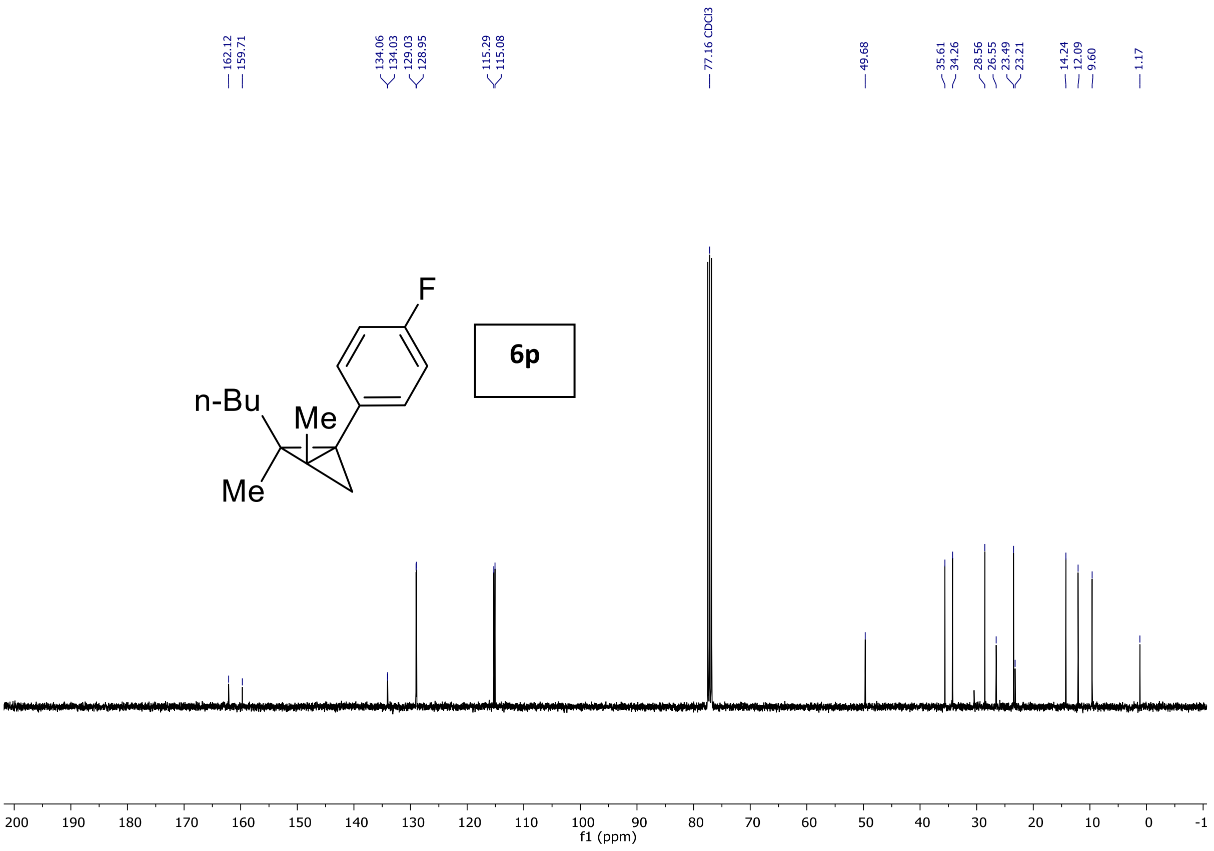
6p
n-Bu
Me
Me
F
f1 (ppm)

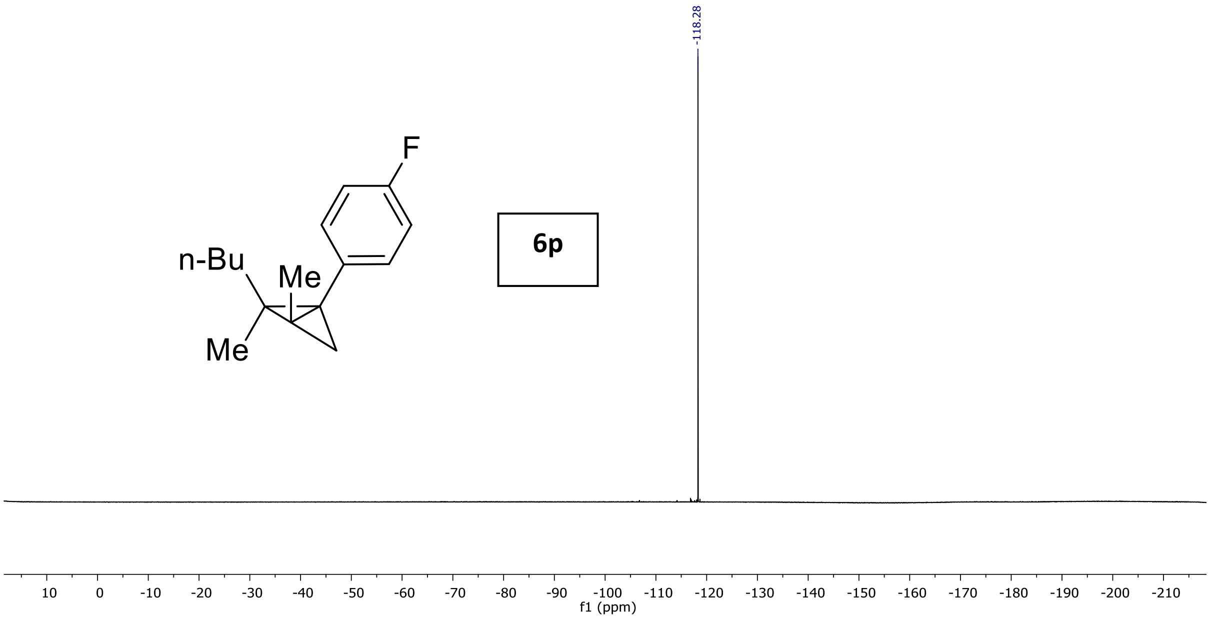
-118.28
F
n-Bu
Me
Me
6p
10 0 -10 -20 -30 -40 -50 -60 -70 -80 -90 -100 -110 -120 -130 -140 -150 -160 -170 -180 -190 -200 -210
f1 (ppm)

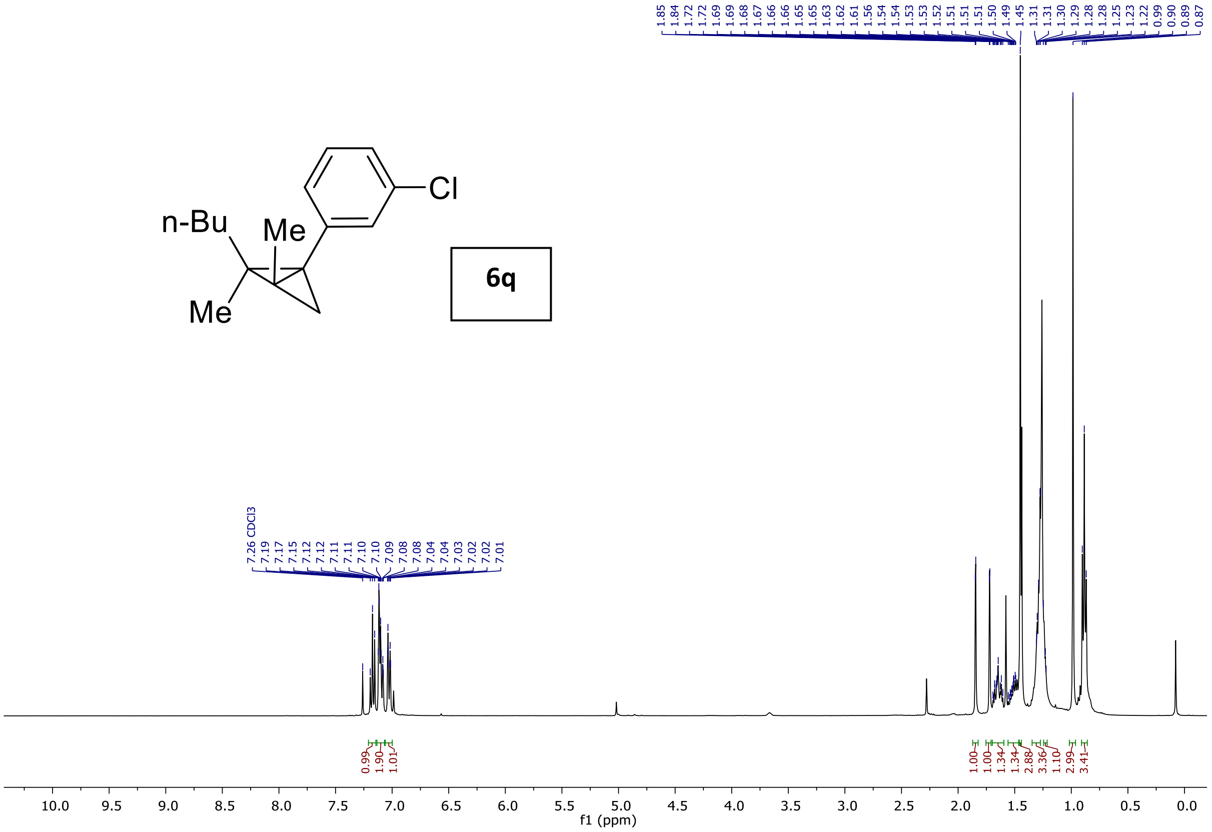

6q
n-Bu
Me
Me
Cl
f1 (ppm)

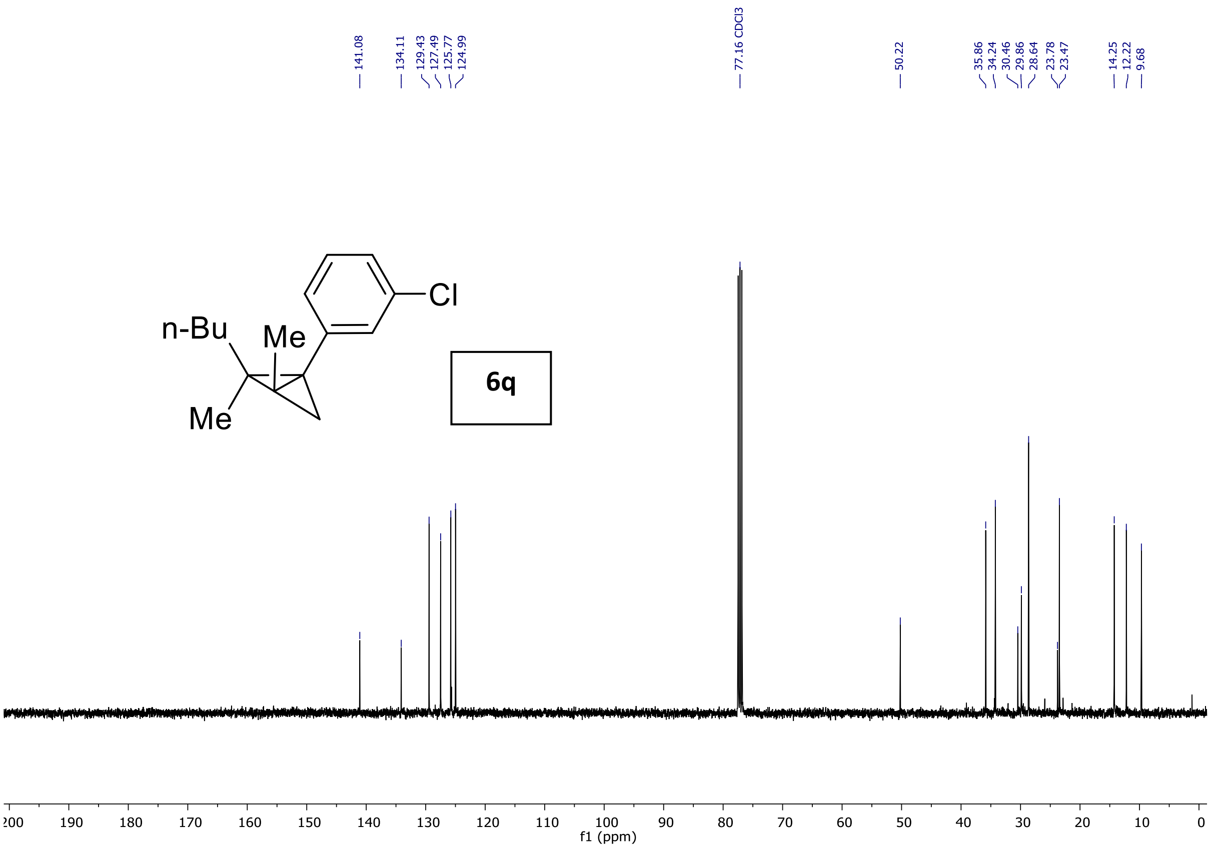

6q
n-Bu
Me
Me
Cl
141.08
134.11
129.43
127.49
125.77
124.99
77.16 CDCl3
50.22
35.86
34.24
30.46
29.86
28.64
23.78
23.47
14.25
12.22
9.68
f1 (ppm)

Crude spectra with internal standard

NMR yield with internal standard (tert-butyl methyl ether)

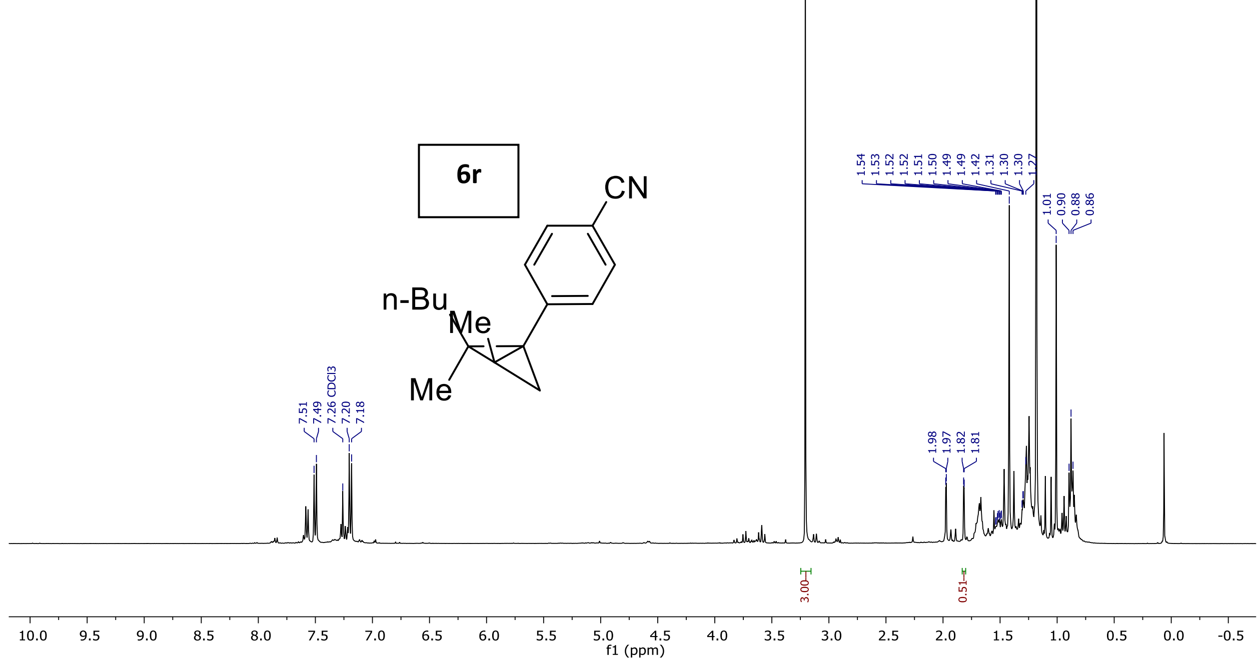

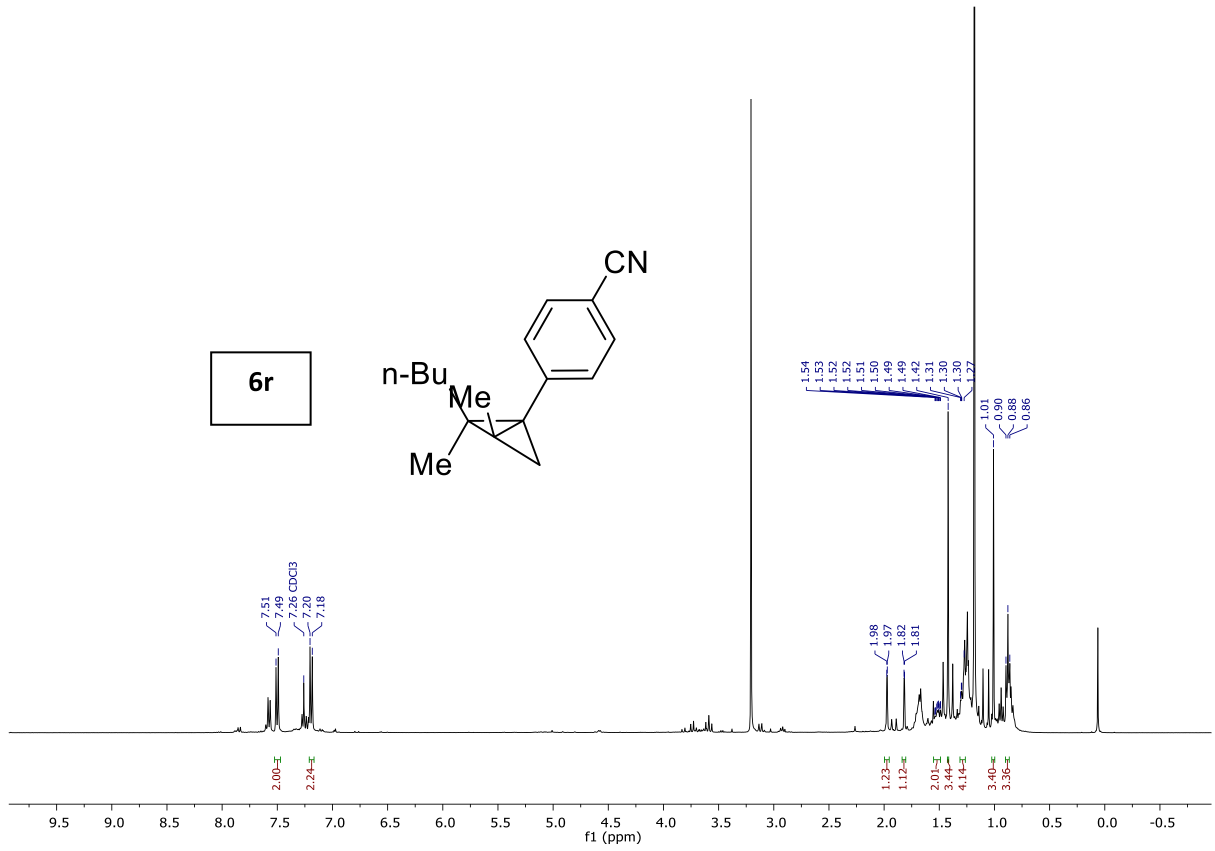

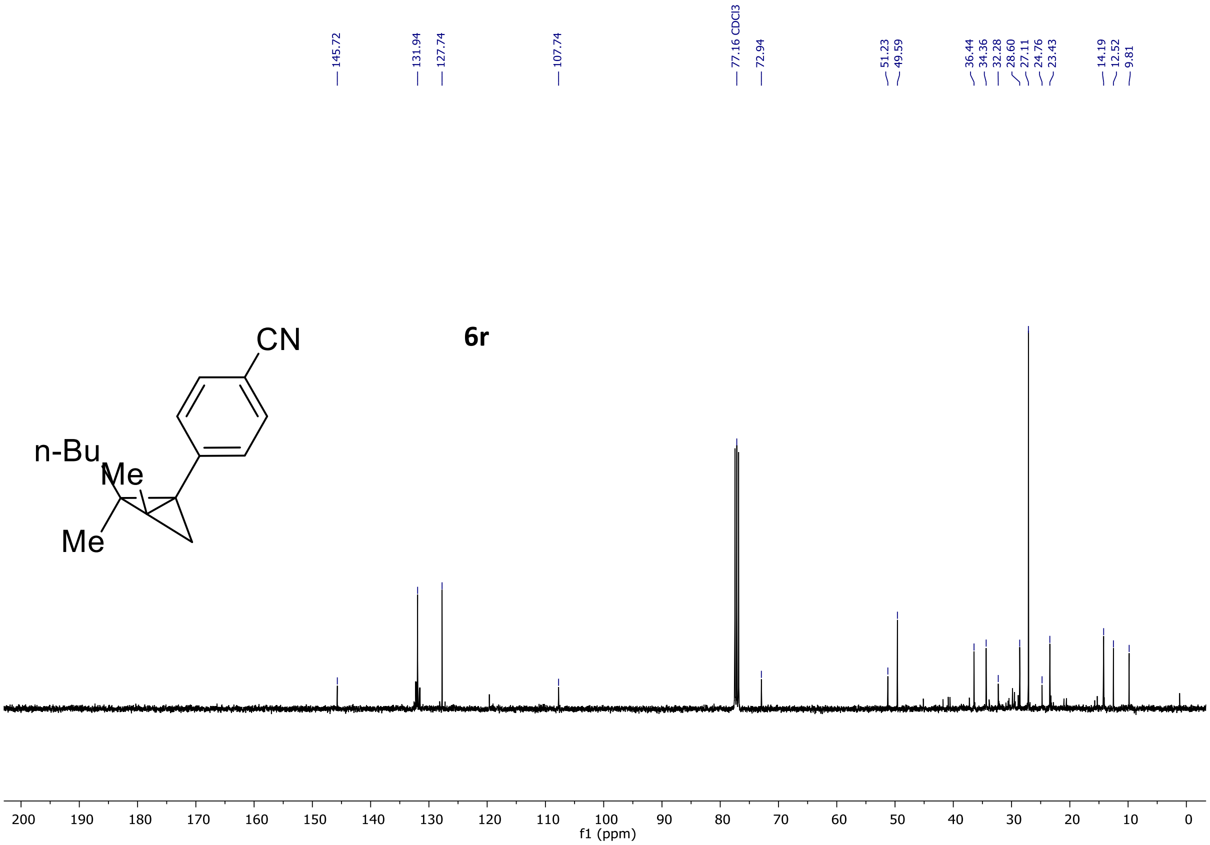
CN
n-Bu
Me
Me
145.72
131.94
127.74
107.74
77.16 CDCl3
72.94
51.23
49.59
36.44
34.36
32.28
28.60
27.11
24.76
23.43
14.19
12.52
9.81
200 190 180 170 160 150 140 130 120 110 100 90 80 70 60 50 40 30 20 10 0
f1 (ppm)

6r

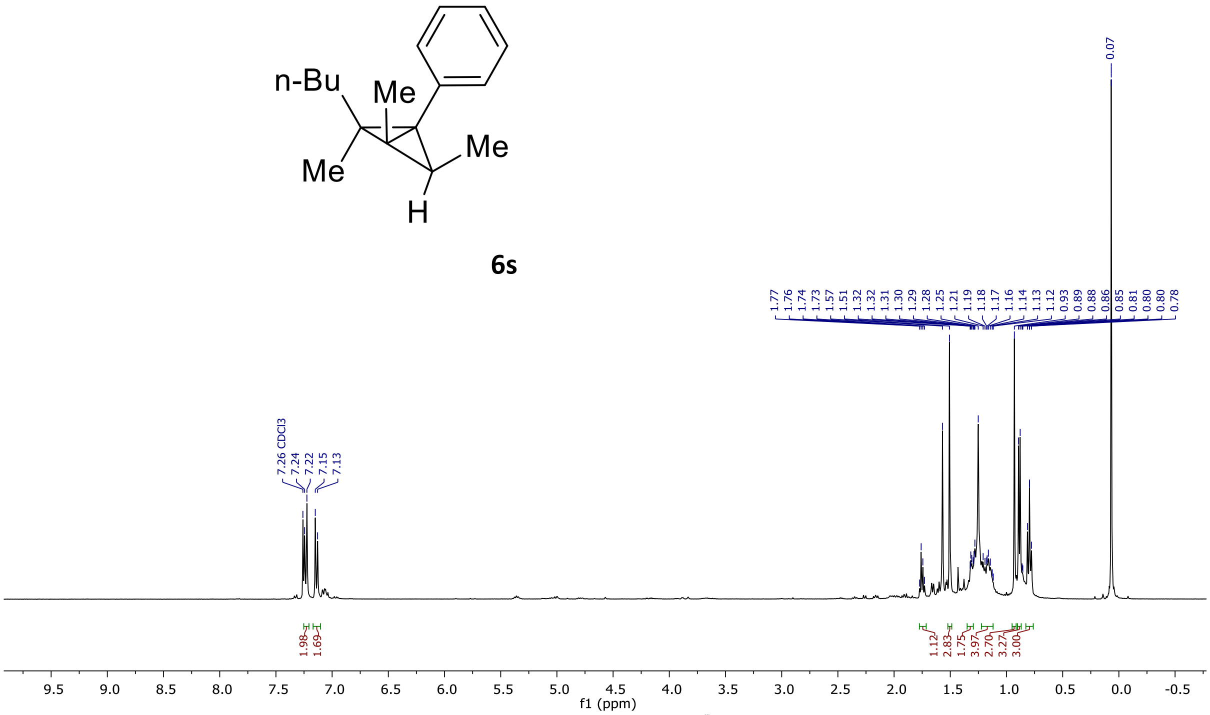
n-Bu
Me
Me
Me
H
6s
7.26 CDCl3
7.24
7.22
7.15
7.13
1.77
1.76
1.74
1.73
1.57
1.51
1.32
1.32
1.31
1.30
1.29
1.28
1.25
1.21
1.19
1.18
1.17
1.16
1.14
1.13
1.12
0.93
0.89
0.88
0.86
0.85
0.81
0.80
0.80
0.78
0.07
1.98
1.69
1.12
2.83
1.75
3.97
2.70
3.27
3.00
f1 (ppm)

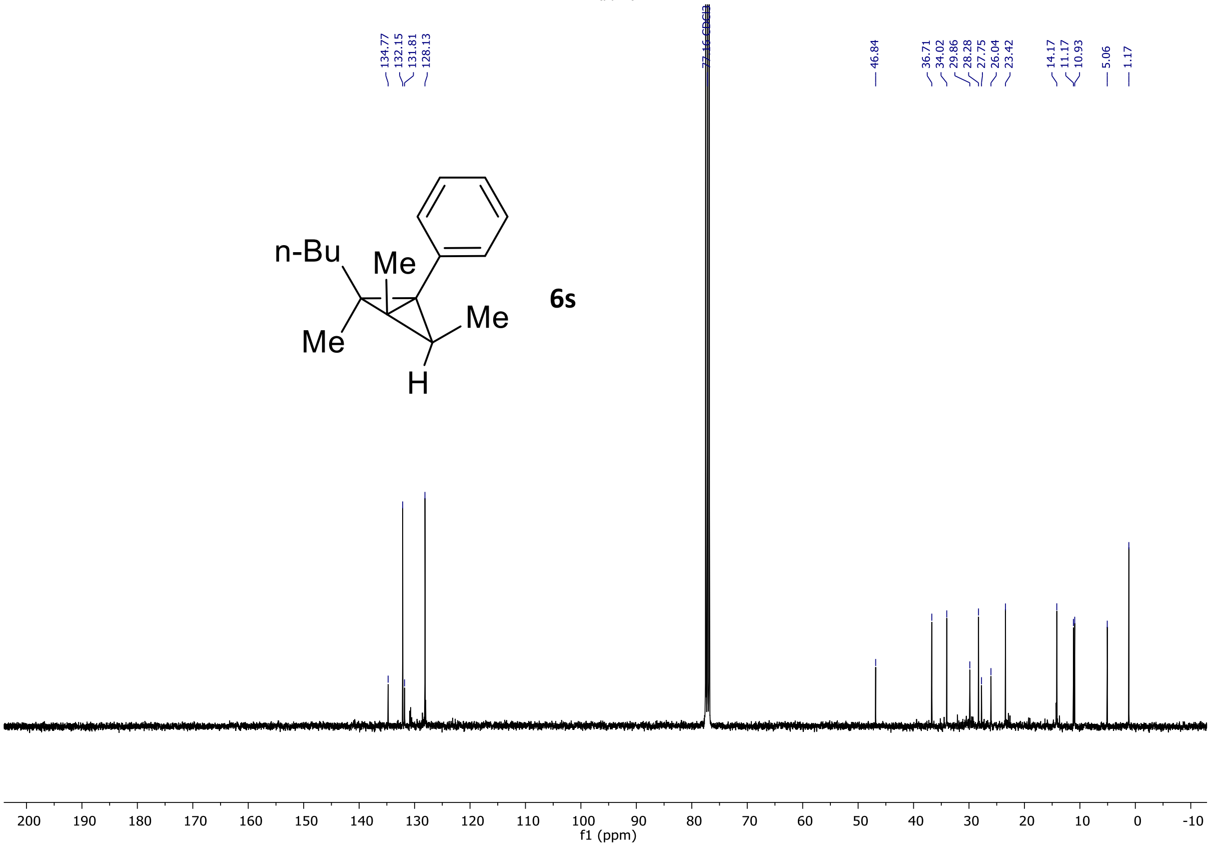
134.77
132.15
131.81
128.13
77.16 CDCl3
46.84
36.71
34.02
29.86
28.28
27.75
26.04
23.42
14.17
11.17
10.93
5.06
1.17
n-Bu
Me
Me
Me
H
6s
f1 (ppm)

2D NOESY NMR of compound **6s**.

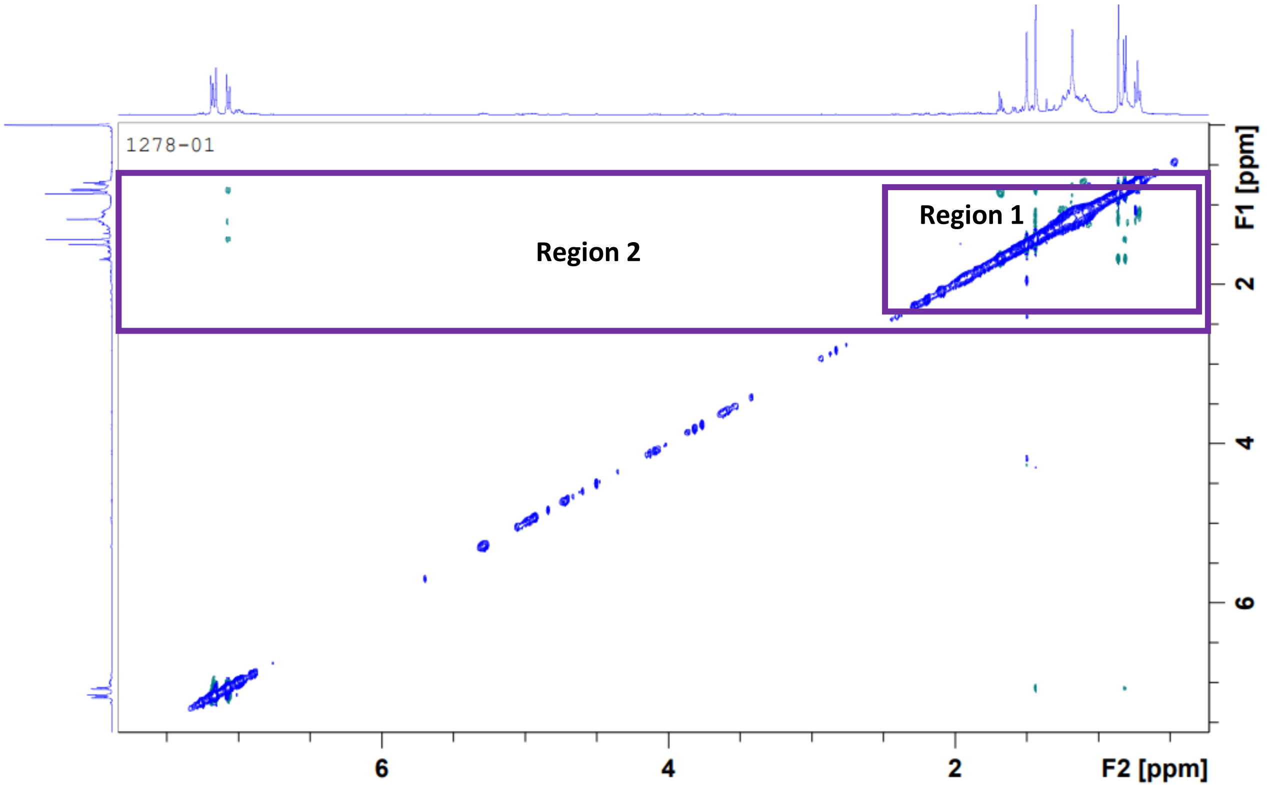

Region 1

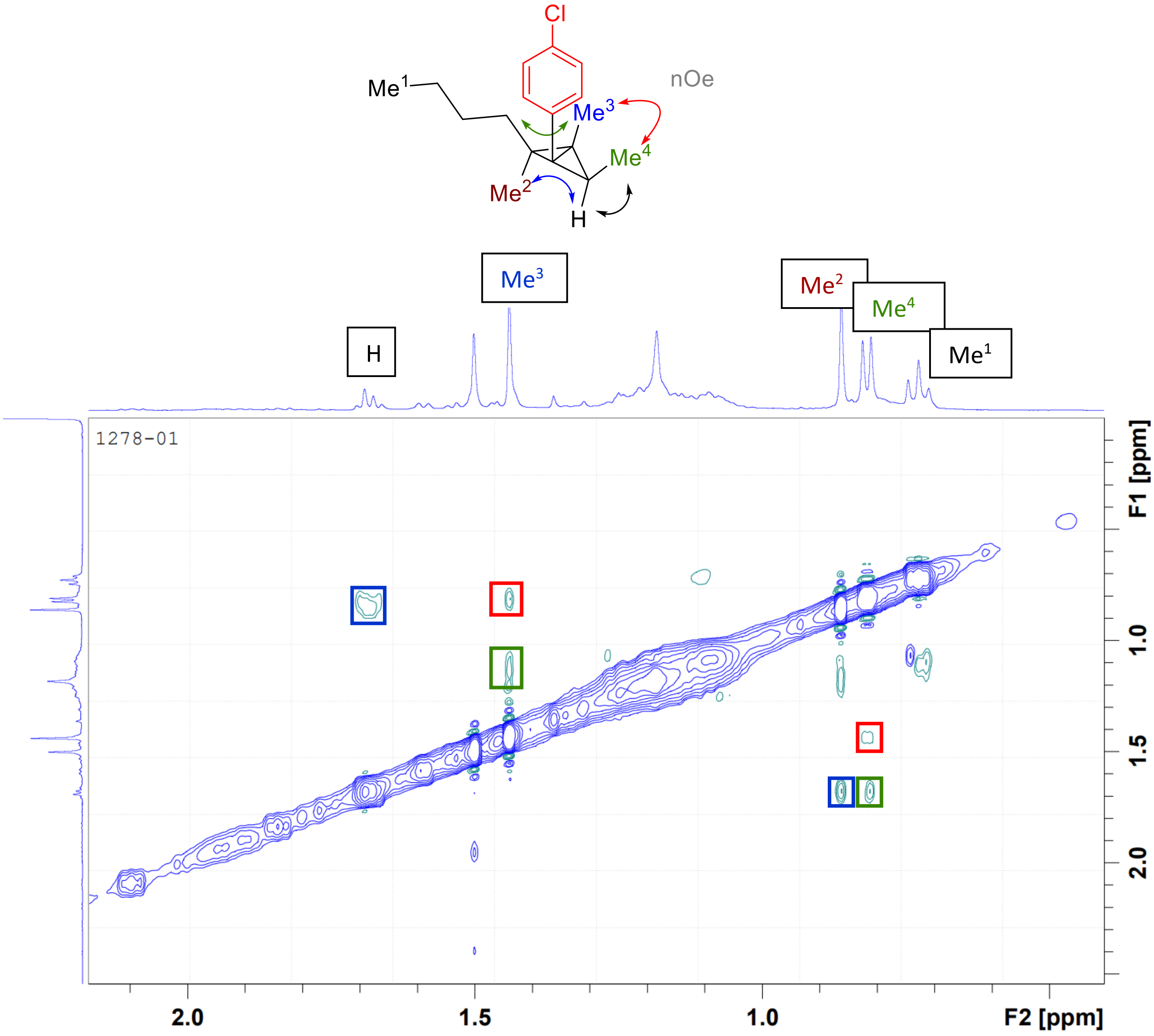
Cl
Me1
Me3
Me4
Me2
H
nOe
Me3
H
Me2
Me4
Me1
1278-01
F1 [ppm]
1.0
1.5
2.0
2.0
1.5
1.0
F2 [ppm]

Region 2

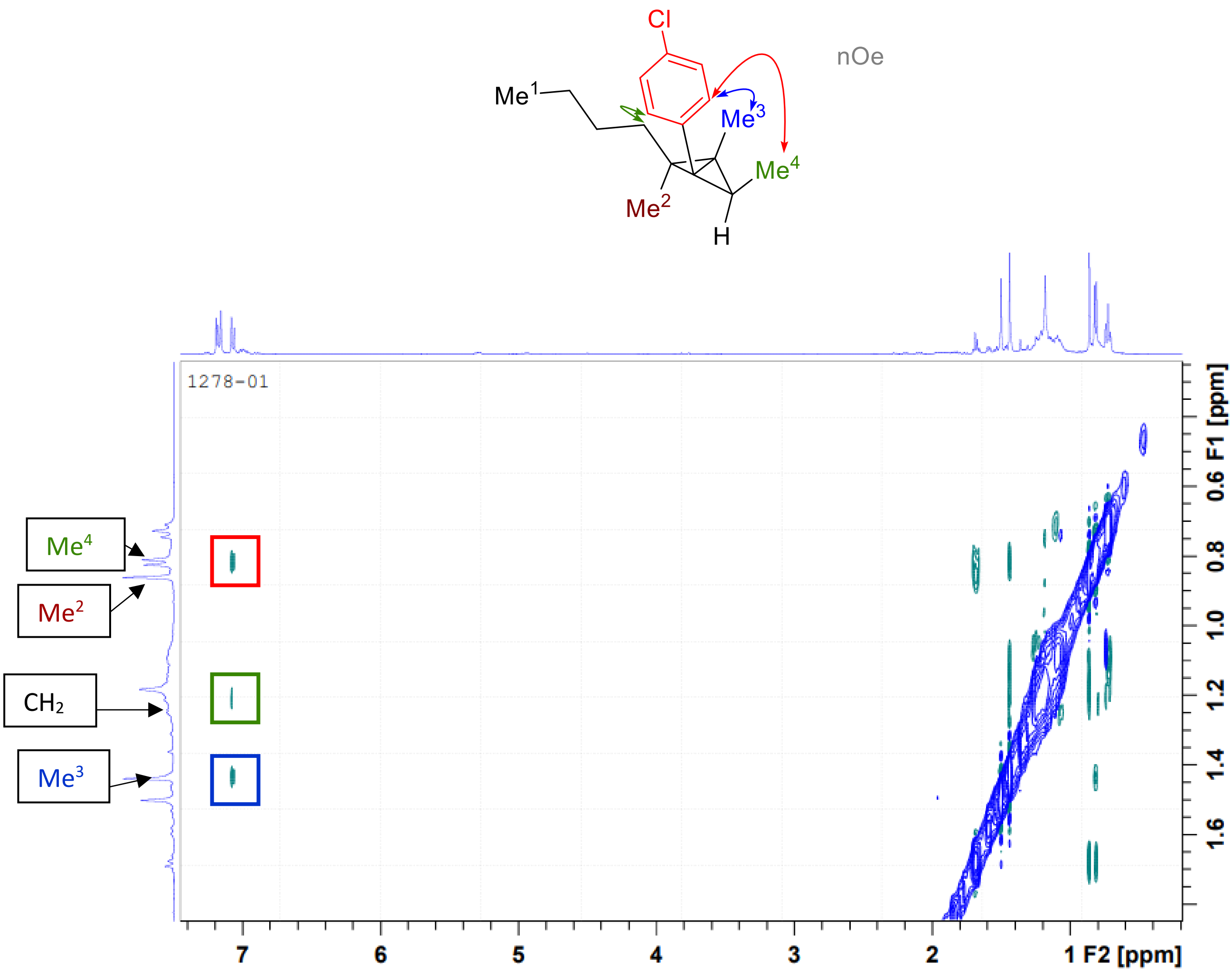

Supplement: Supplementary file 1 — ja4c04438_si_001.pdf [file ja4c04438_si_001.pdf]
